# Supplementary material for: Distinct core minor pilin complexes prime specialized type IV filaments in cyanobacteria
Source: PLoS Genet. 2026 Oct 26;22(10):e1012337. doi: 10.1371/journal.pgen.1012337 (PMC13759059; doi:10.1371/journal.pgen.1012337)
Supplement: Supplementary file 1 [file pgen.1012337.s012.pdf]

Fig. 3A cellular extracts membrane A

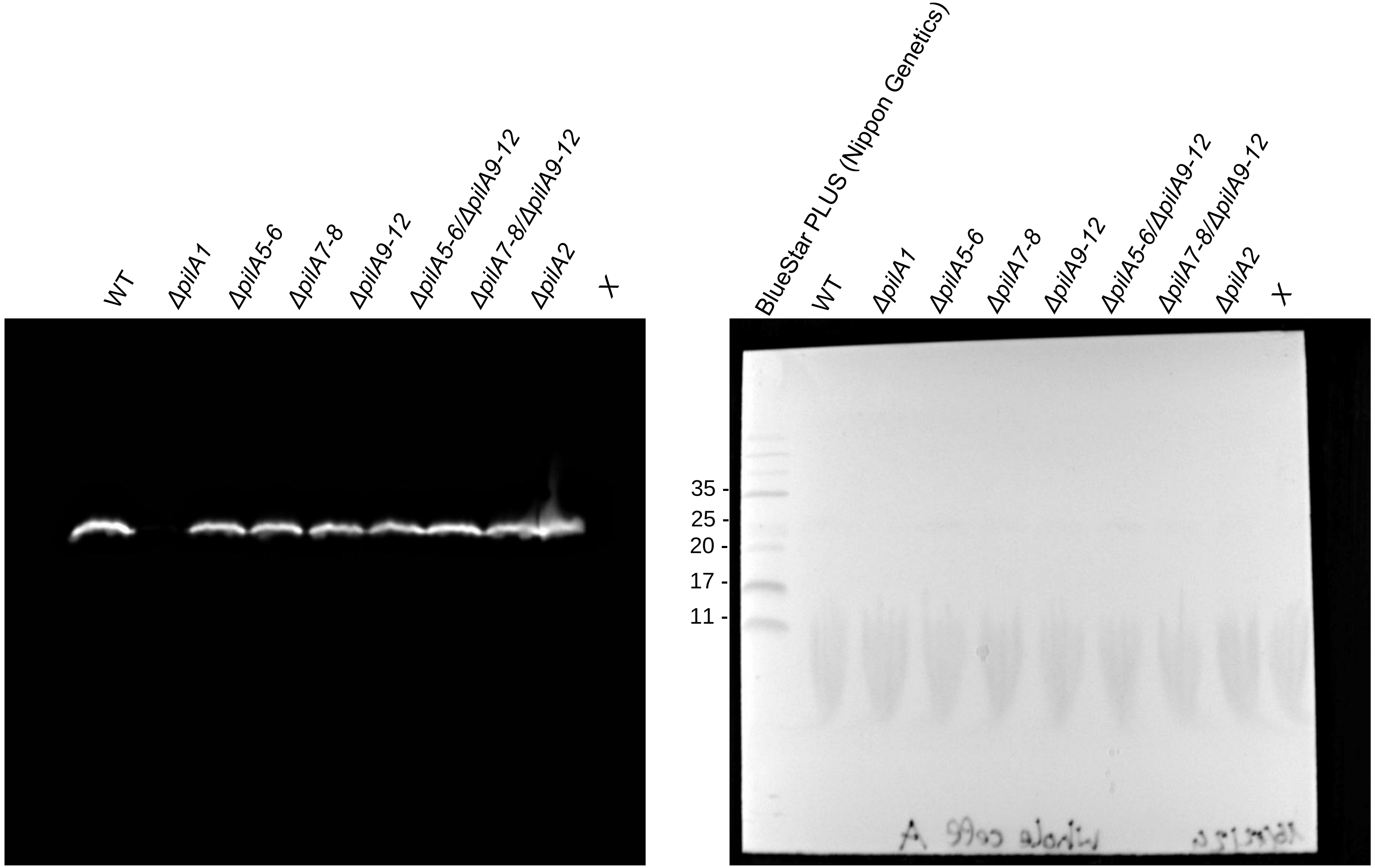

Samples (7.5 µl) were resolved on a 15% SDS-PAGE gel and transferred to a nitrocellulose membrane. The blot was probed with primary anti-PilA1 (1:10,000) and secondary anti-rabbit HRP (1:20,000) antibodies, then visualized using Pierce ECL Plus Western Blotting Substrate.

Fig. 3A cellular extracts membrane B

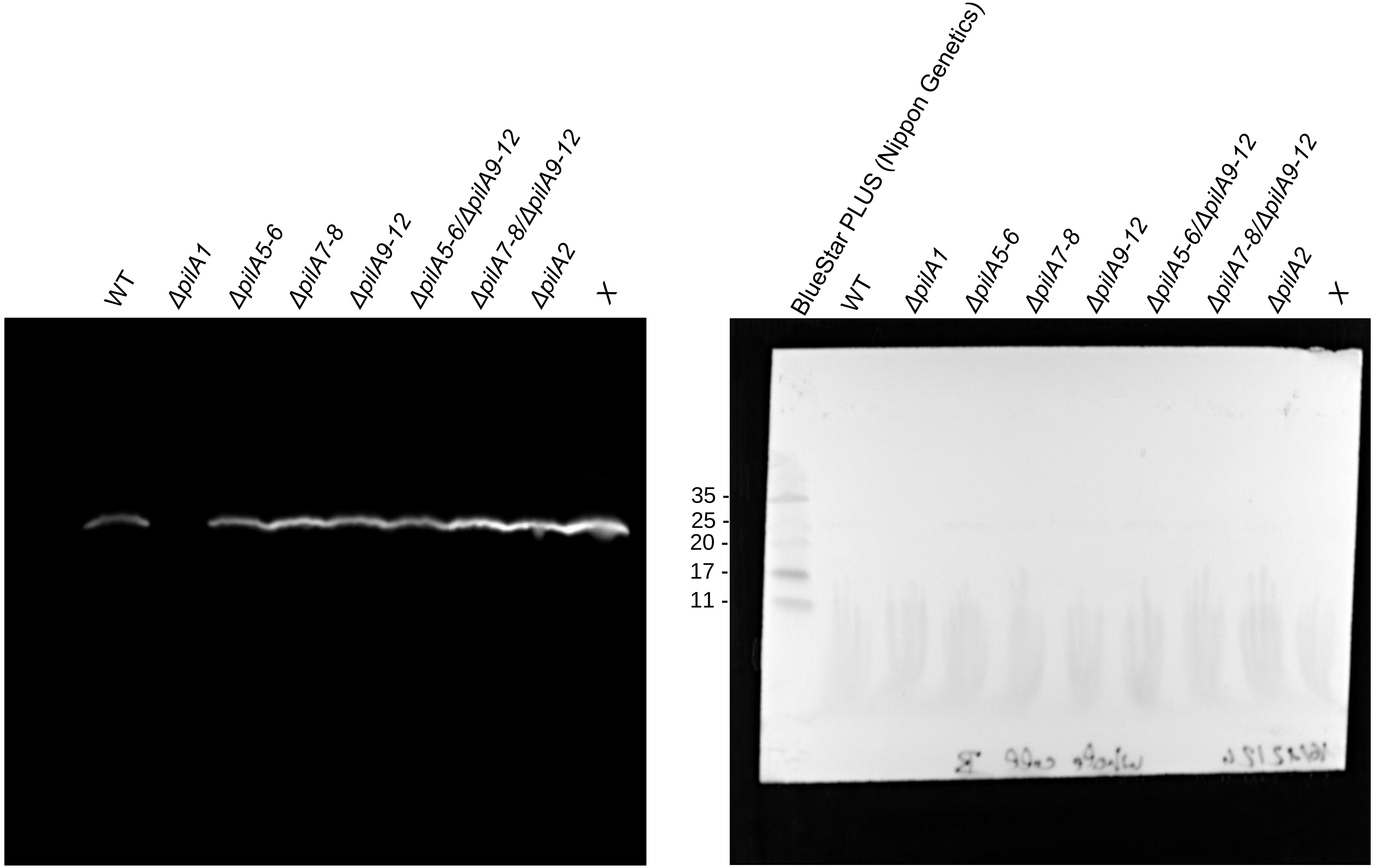

Samples (7.5 µl) were resolved on a 15% SDS-PAGE gel and transferred to a nitrocellulose membrane. The blot was probed with primary anti-PilA1 (1:10,000) and secondary anti-rabbit HRP (1:20,000) antibodies, then visualized using Pierce ECL Plus Western Blotting Substrate.

Fig. 3A cellular extracts membrane C

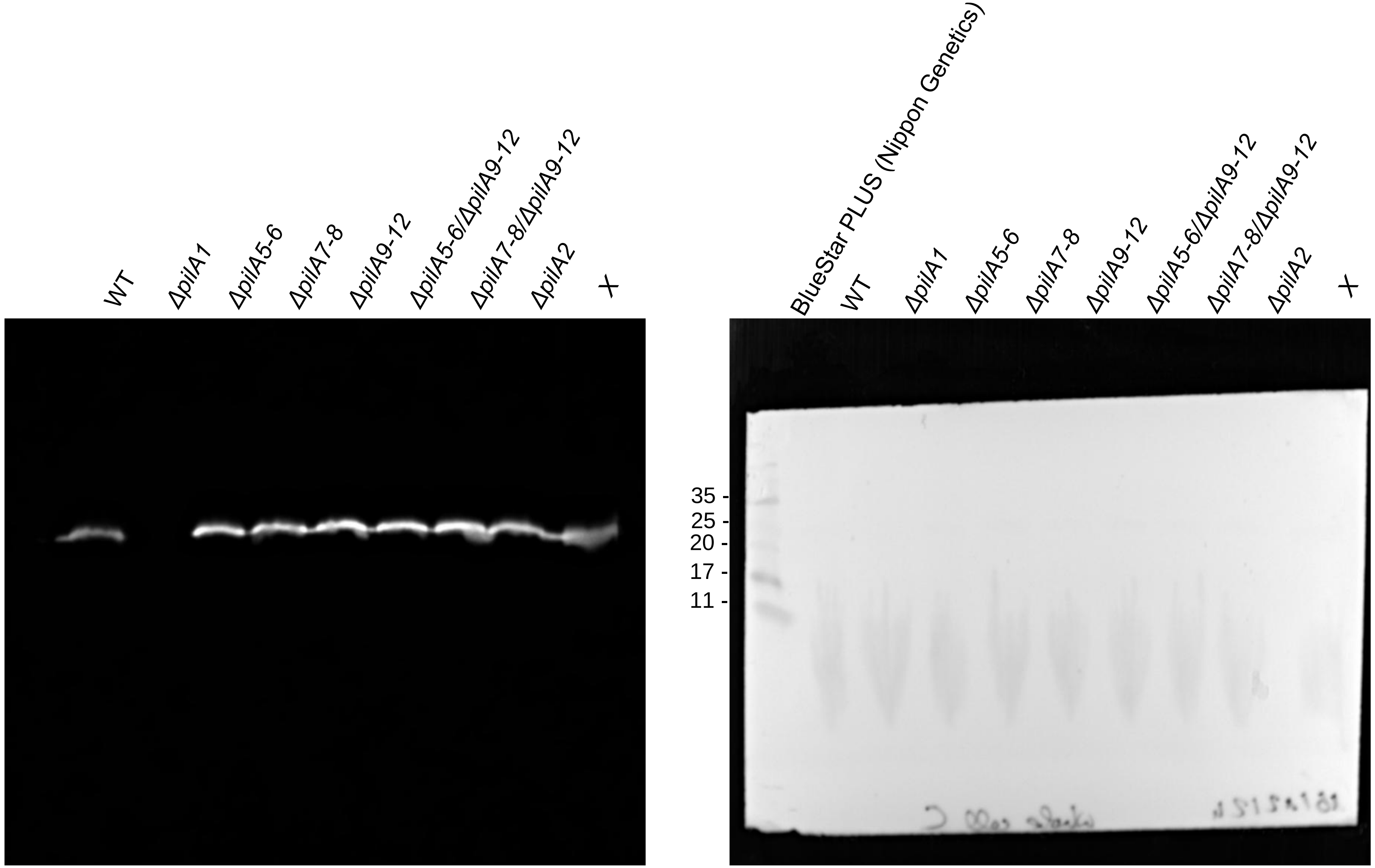

Samples (7.5 µl) were resolved on a 15% SDS-PAGE gel and transferred to a nitrocellulose membrane. The blot was probed with primary anti-PilA1 (1:10,000) and secondary anti-rabbit HRP (1:20,000) antibodies, then visualized using Pierce ECL Plus Western Blotting Substrate.

Fig. 3A sheared pili fraction membrane A

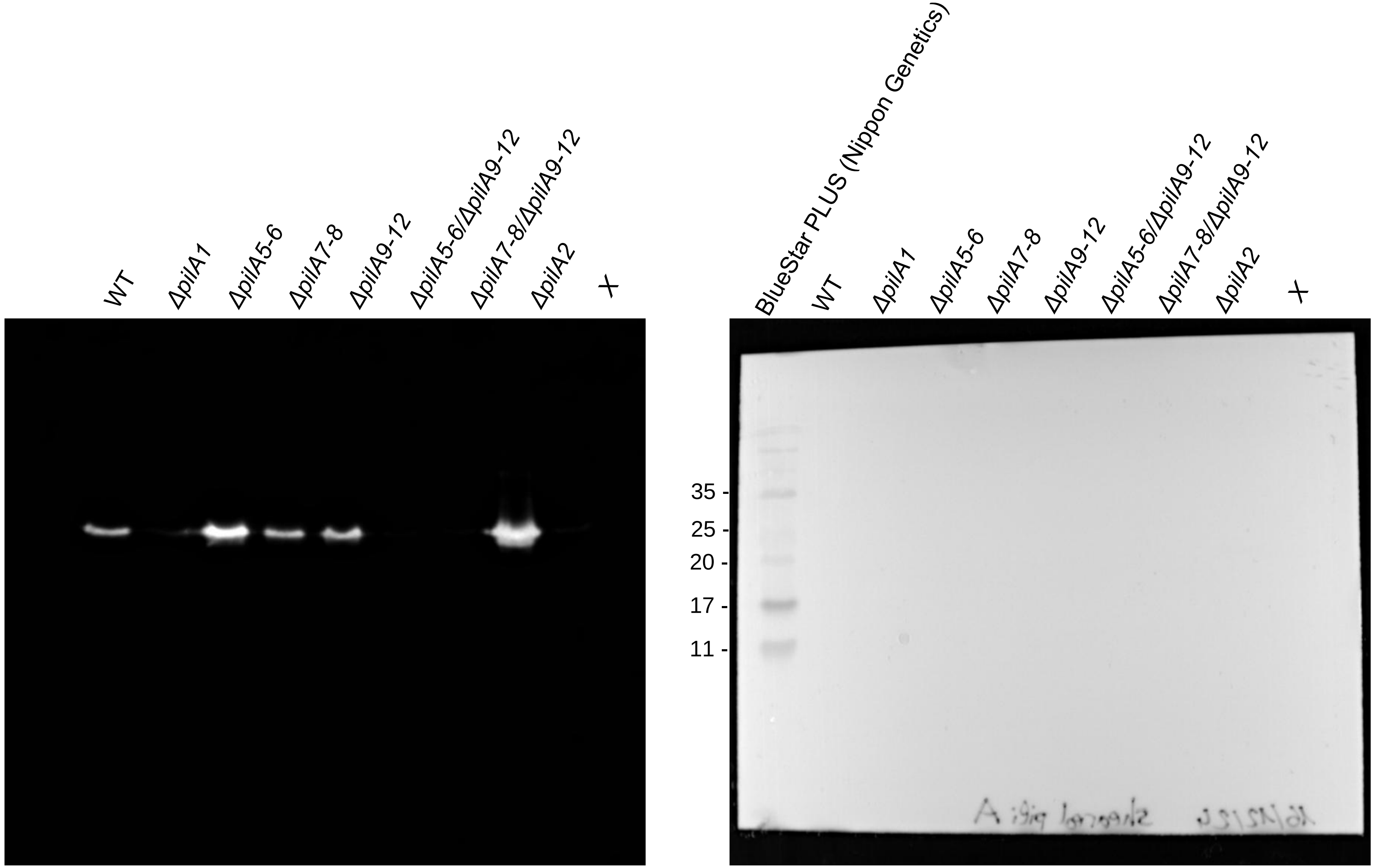

Samples (7.5 µl) were resolved on a 15% SDS-PAGE gel and transferred to a nitrocellulose membrane. The blot was probed with primary anti-PilA1 (1:10,000) and secondary anti-rabbit HRP (1:20,000) antibodies, then visualized using Pierce ECL Plus Western Blotting Substrate.

Fig. 3A sheared pili fraction membrane B

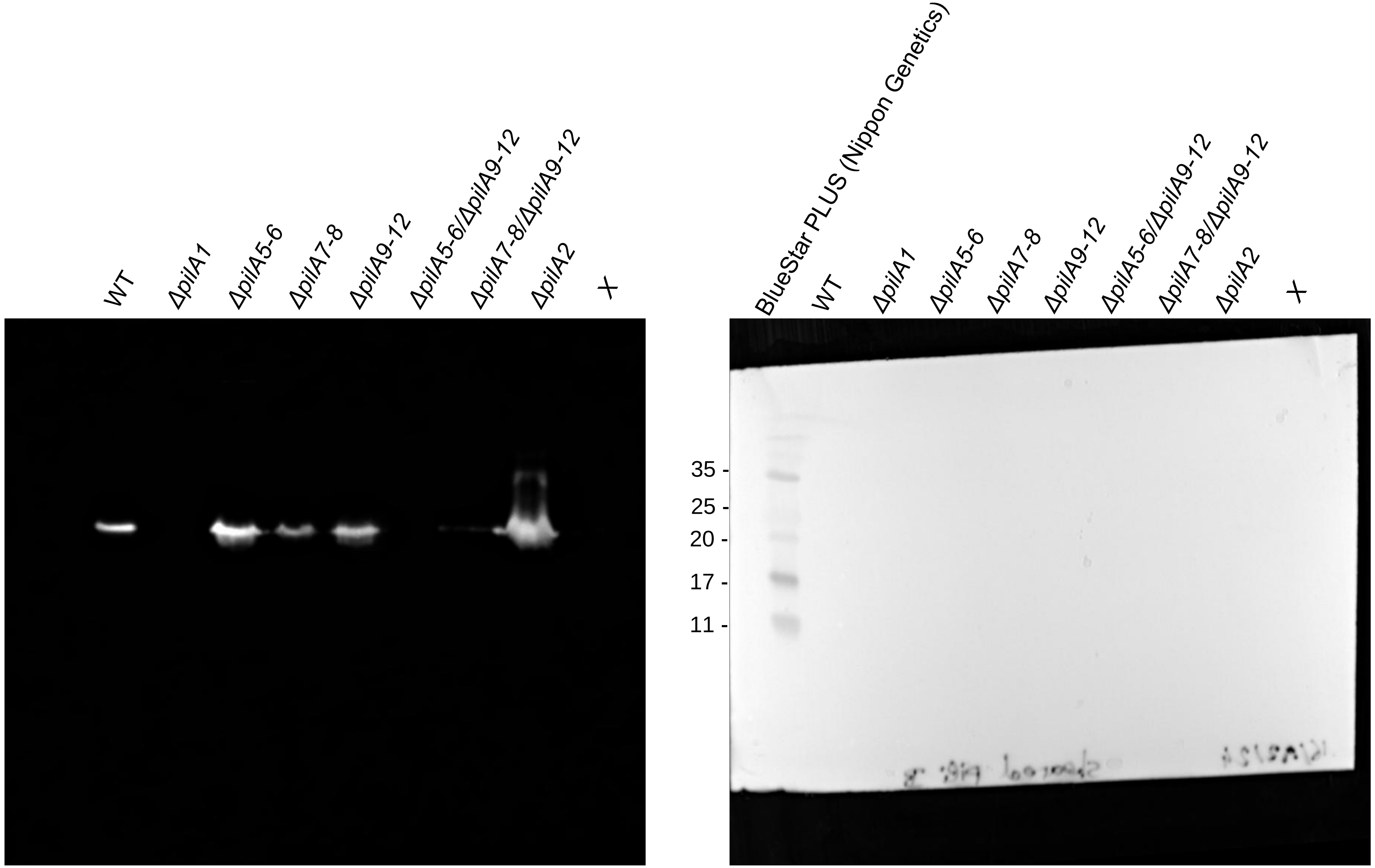

Samples (7.5 µl) were resolved on a 15% SDS-PAGE gel and transferred to a nitrocellulose membrane. The blot was probed with primary anti-PilA1 (1:10,000) and secondary anti-rabbit HRP (1:20,000) antibodies, then visualized using Pierce ECL Plus Western Blotting Substrate.

Fig. 3A sheared pili fraction membrane C

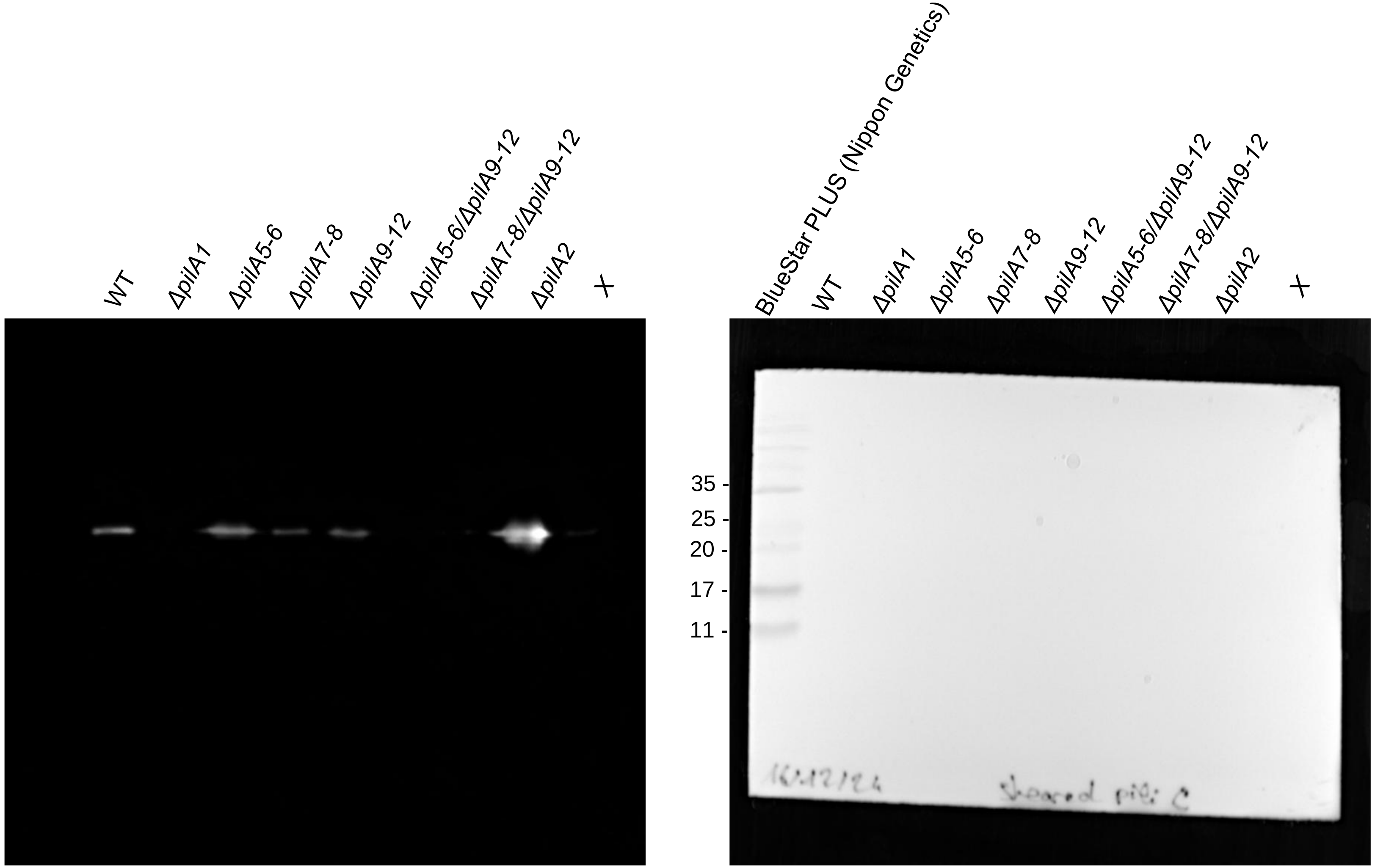

Samples (7.5 µl) were resolved on a 15% SDS-PAGE gel and transferred to a nitrocellulose membrane. The blot was probed with primary anti-PilA1 (1:10,000) and secondary anti-rabbit HRP (1:20,000) antibodies, then visualized using Pierce ECL Plus Western Blotting Substrate.

Fig. S8A cPCR of *pilA1* cysteine mutants

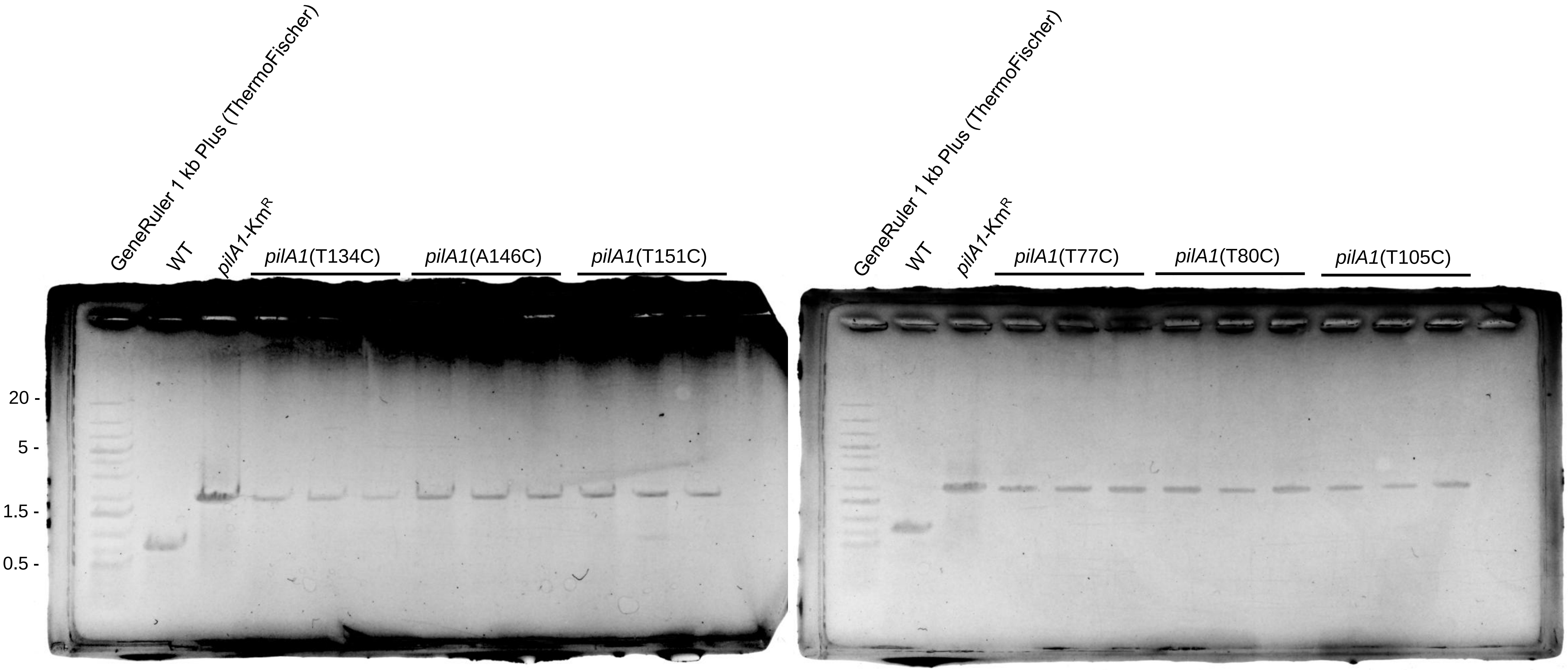

Colony PCR of *pilA1* mutant strains was performed using primers #244_PilA1_KO_fwd (5'-CGATAGAAAATTGGGCTTTC-3') and #245_PilA1_KO_rev (5'-GGTTATTAATGCTAGAAGAAC-3'). Products were resolved on a 0.8% agarose gel. The pUC-pilA1::Km plasmid served as a positive control.

Fig. S8B Western blot of sheared pili fractions of *pilA1* cysteine mutants

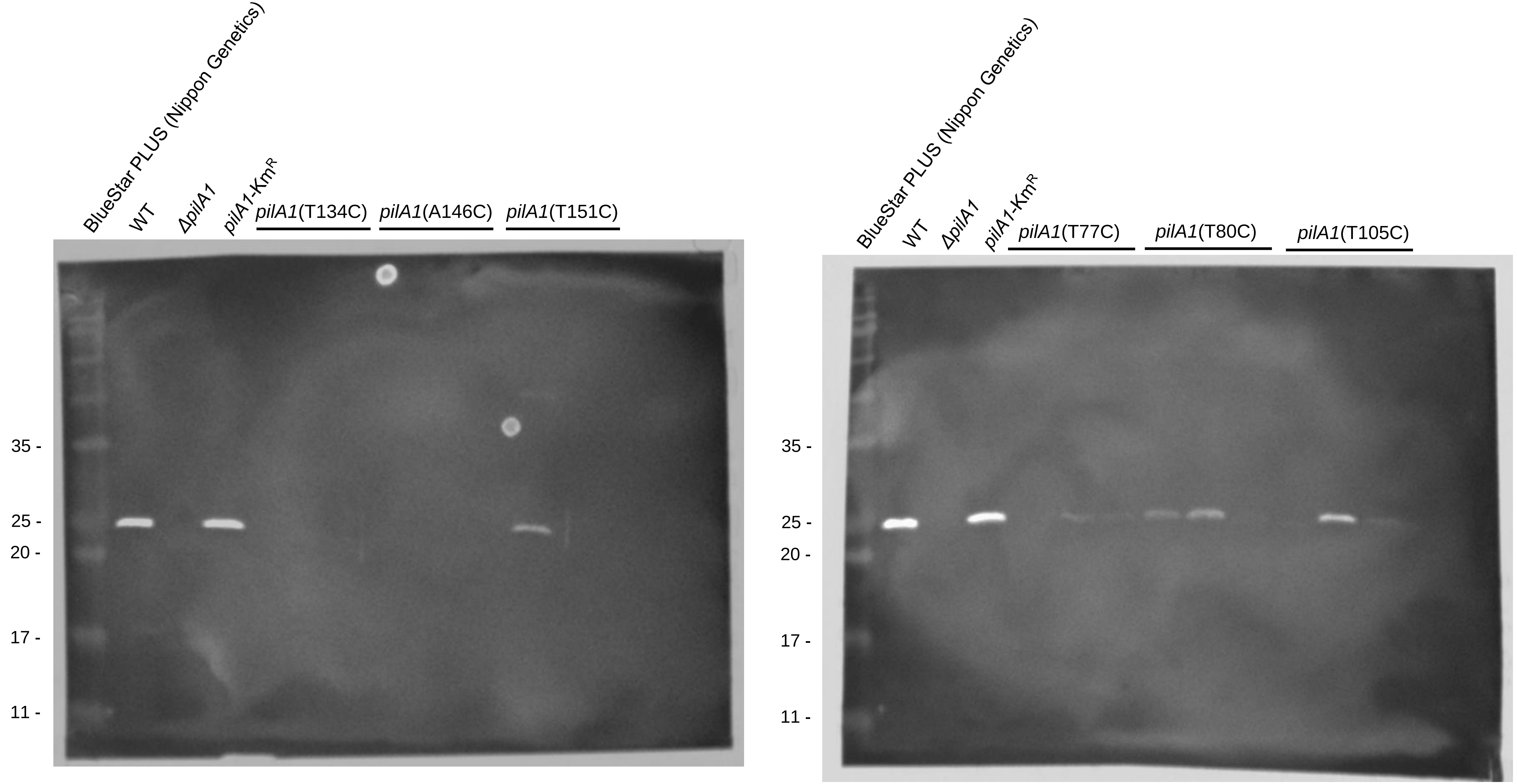

Samples (5 µl) were resolved on a 15% SDS-PAGE gel and transferred to a nitrocellulose membrane. The blot was probed with primary anti-PilA1 (1:25,000) and secondary anti-rabbit HRP (1:50,000) antibodies, then visualized using Pierce Pierce ECL Plus Western Blotting Substrate Western Blotting Substrate
