# Supplementary material for: Distinct core minor pilin complexes prime specialized type IV filaments in cyanobacteria
Source: PLoS Genet. 2026 Oct 26;22(10):e1012337. doi: 10.1371/journal.pgen.1012337 (PMC13759059; doi:10.1371/journal.pgen.1012337)
Supplement: Supplementary file 2 [file pgen.1012337.s014.pdf]

Acaryochloris marina (strain MBIC 11017)

GCA_000018105.1

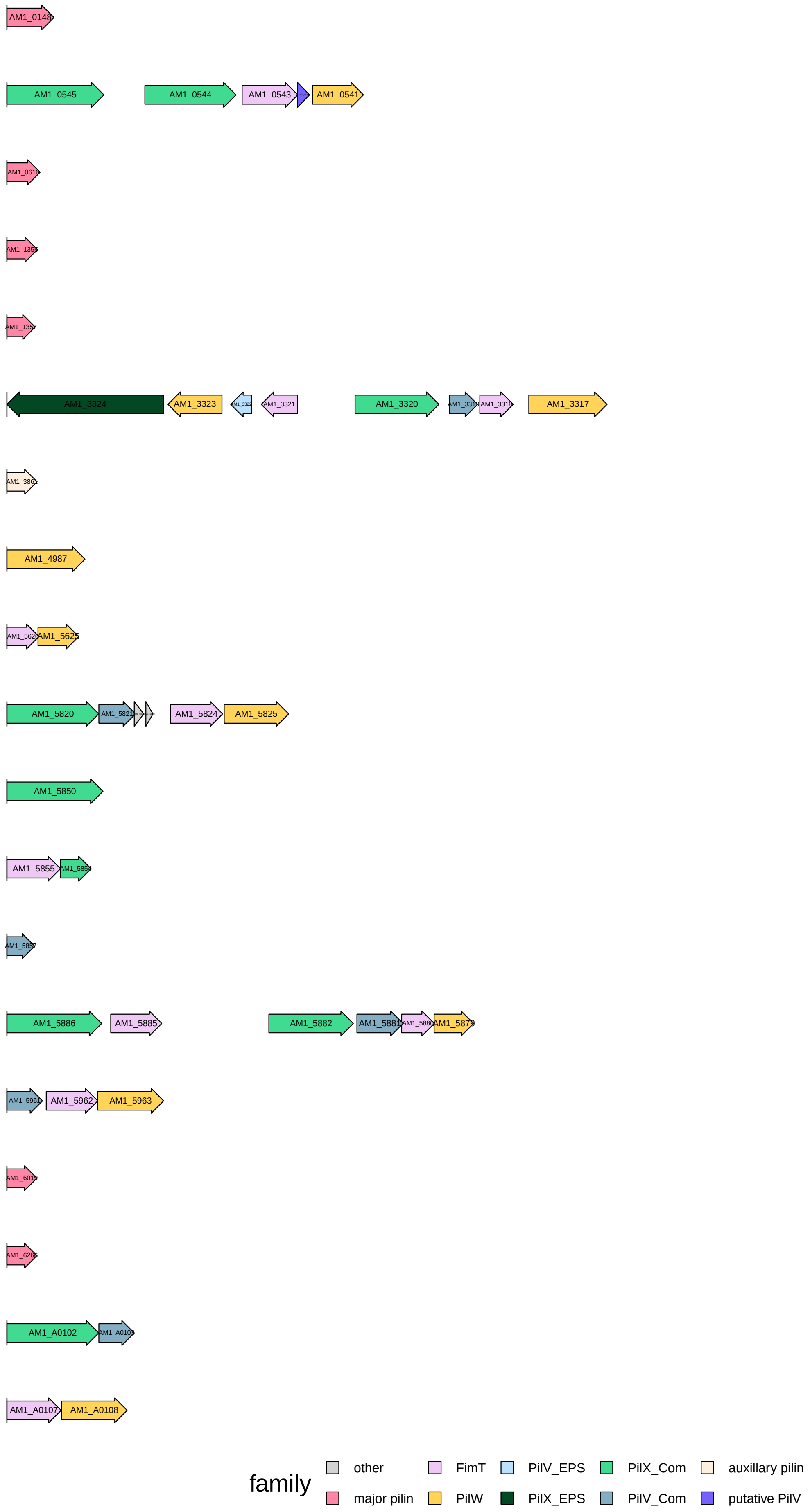

locus	gene	family	start	end
177	AM1_0148	major pilin	147100	147852
180	AM1_0541	PilW	522709	523521
180	AM1_0542	putative PilV	523570	523761
180	AM1_0543	FimT	523758	524648
180	AM1_0544	PilX_Com	524745	526202
180	AM1_0545	PilX_Com	526856	528406
182	AM1_0616	major pilin	605018	605548
185	AM1_1355	major pilin	1332259	1332747
186	AM1_1357	major pilin	1333817	1334269
195	AM1_3317	PilW	3348754	3350004
195	AM1_3318	FimT	3350254	3350787
195	AM1_3319	PilV_Com	3350823	3351272
195	AM1_3320	PilX_Com	3351441	3352781
195	AM1_3321	FimT	3353703	3354281
195	AM1_3322	PilV_EPS	3354433	3354768
195	AM1_3323	PilW	3354908	3355771
195	AM1_3324	PilX_EPS	3355840	3358344
196	AM1_3861	auxillary pilin	3912558	3913040
202	AM1_4987	PilW	5033573	5034820
203	AM1_5625	PilW	5685891	5686541
203	AM1_5626	FimT	5686526	5687038
205	AM1_5820	PilX_Com	5898718	5900184
205	AM1_5821	PilV_Com	5900187	5900774
205	AM1_5822	other	5900752	5900904
205	AM1_5823	other	5900938	5901051
205	AM1_5824	FimT	5901332	5902165
205	AM1_5825	PilW	5902189	5903220
206	AM1_5850	PilX_Com	5917715	5919250
207	AM1_5854	PilX_Com	5920057	5920548
207	AM1_5855	FimT	5920545	5921402
208	AM1_5857	PilV_Com	5921909	5922352
209	AM1_5879	PilW	5938062	5938694
209	AM1_5880	FimT	5938691	5939212
209	AM1_5881	PilV_Com	5939190	5939927
209	AM1_5882	PilX_Com	5939985	5941334
209	AM1_5885	FimT	5943046	5943861
209	AM1_5886	PilX_Com	5944006	5945520
210	AM1_5961	PilV_Com	6024260	6024829
210	AM1_5962	FimT	6024888	6025712
210	AM1_5963	PilW	6025709	6026764
211	AM1_6019	major pilin	6103438	6103917
212	AM1_6266	major pilin	6342544	6343023
213	AM1_A0102	PilX_Com	93491	94957
213	AM1_A0103	PilV_Com	94960	95526
214	AM1_A0107	FimT	96085	96951
214	AM1_A0108	PilW	96960	98006

Acaryochloris thomasi RCC1774

GCA_003231495.1

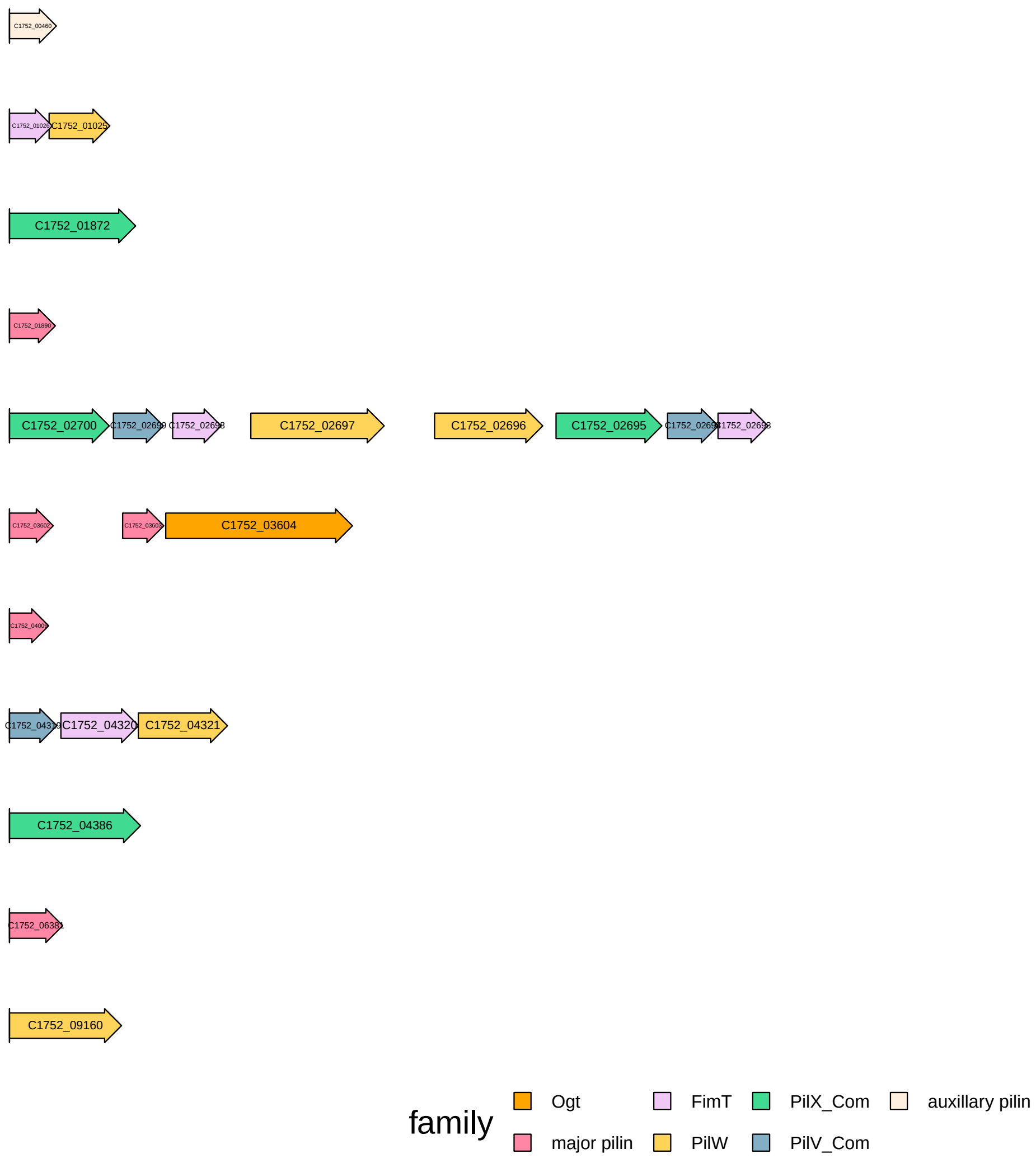

locus	gene	family	start	end
1358	C1752_00460	auxillary pilin	485325	485873
1362	C1752_01025	PilW	472498	473208
1362	C1752_01026	FimT	473171	473671
1364	C1752_01872	PilX_Com	418119	419594
1365	C1752_01890	major pilin	26332	26868
1367	C1752_02693	FimT	8634	9218
1367	C1752_02694	PilV_Com	9215	9808
1367	C1752_02695	PilX_Com	9874	11112
1367	C1752_02696	PilW	11267	12532
1367	C1752_02697	PilW	13121	14680
1367	C1752_02698	FimT	15028	15594
1367	C1752_02699	PilV_Com	15703	16287
1367	C1752_02700	PilX_Com	16336	17502
1369	C1752_03602	major pilin	40612	41124
1369	C1752_03603	major pilin	41935	42417
1369	C1752_03604	Ogt	42439	44622
1372	C1752_04009	major pilin	80559	81017
1374	C1752_04319	PilV_Com	45466	46020
1374	C1752_04320	FimT	46067	46975
1374	C1752_04321	PilW	46972	48015
1375	C1752_04386	PilX_Com	115836	117368
1380	C1752_06381	major pilin	65011	65634
1381	C1752_09160	PilW	39139	40449

Adonisia turfae CCMR0081

GCA_011009555.1

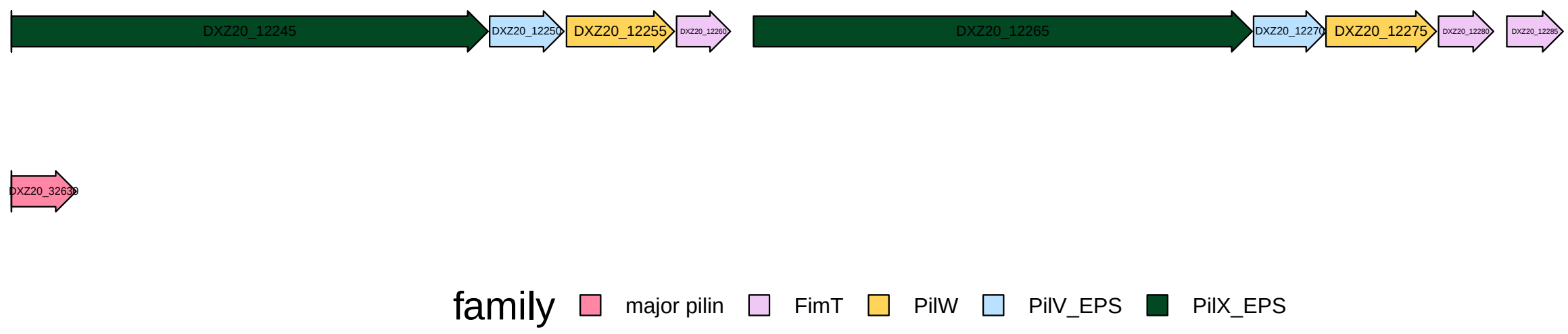

locus	gene	family	start	end
1573	DXZ20_12245	PilX_EPS	1855682	1860295
1573	DXZ20_12250	PilV_EPS	1860310	1861029
1573	DXZ20_12255	PilW	1861054	1862097
1573	DXZ20_12260	FimT	1862119	1862640
1573	DXZ20_12265	PilX_EPS	1862863	1867689
1573	DXZ20_12270	PilV_EPS	1867703	1868410
1573	DXZ20_12275	PilW	1868404	1869471
1573	DXZ20_12280	FimT	1869493	1870026
1573	DXZ20_12285	FimT	1870154	1870699
1582	DXZ20_32630	major pilin	6716350	6716976

Aliterella atlantica CENA595

GCA_000952155.1

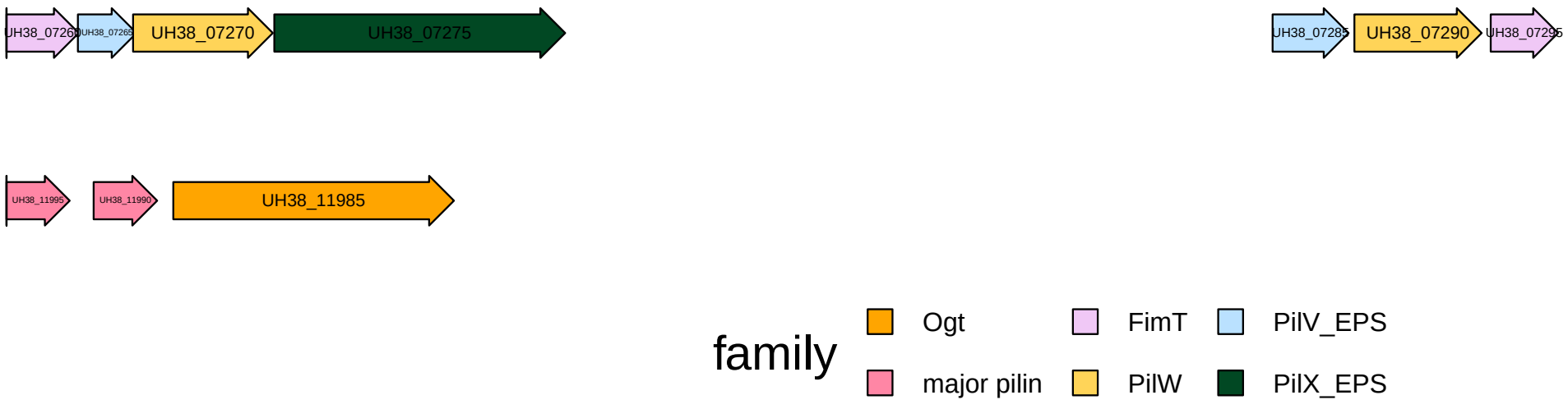

locus	gene	family	start	end
874	UH38_07260	FimT	17513	18088
874	UH38_07265	PilV_EPS	18081	18548
874	UH38_07270	PilW	18520	19632
874	UH38_07275	PilX_EPS	19644	21959
874	UH38_07285	PilV_EPS	27589	28194
874	UH38_07290	PilW	28240	29253
874	UH38_07295	FimT	29325	29861
879	UH38_11985	Ogt	62862	65096
879	UH38_11990	major pilin	65224	65730
879	UH38_11995	major pilin	65920	66423

Allocoleopsis franciscana PCC 7113

GCA_000317515.1

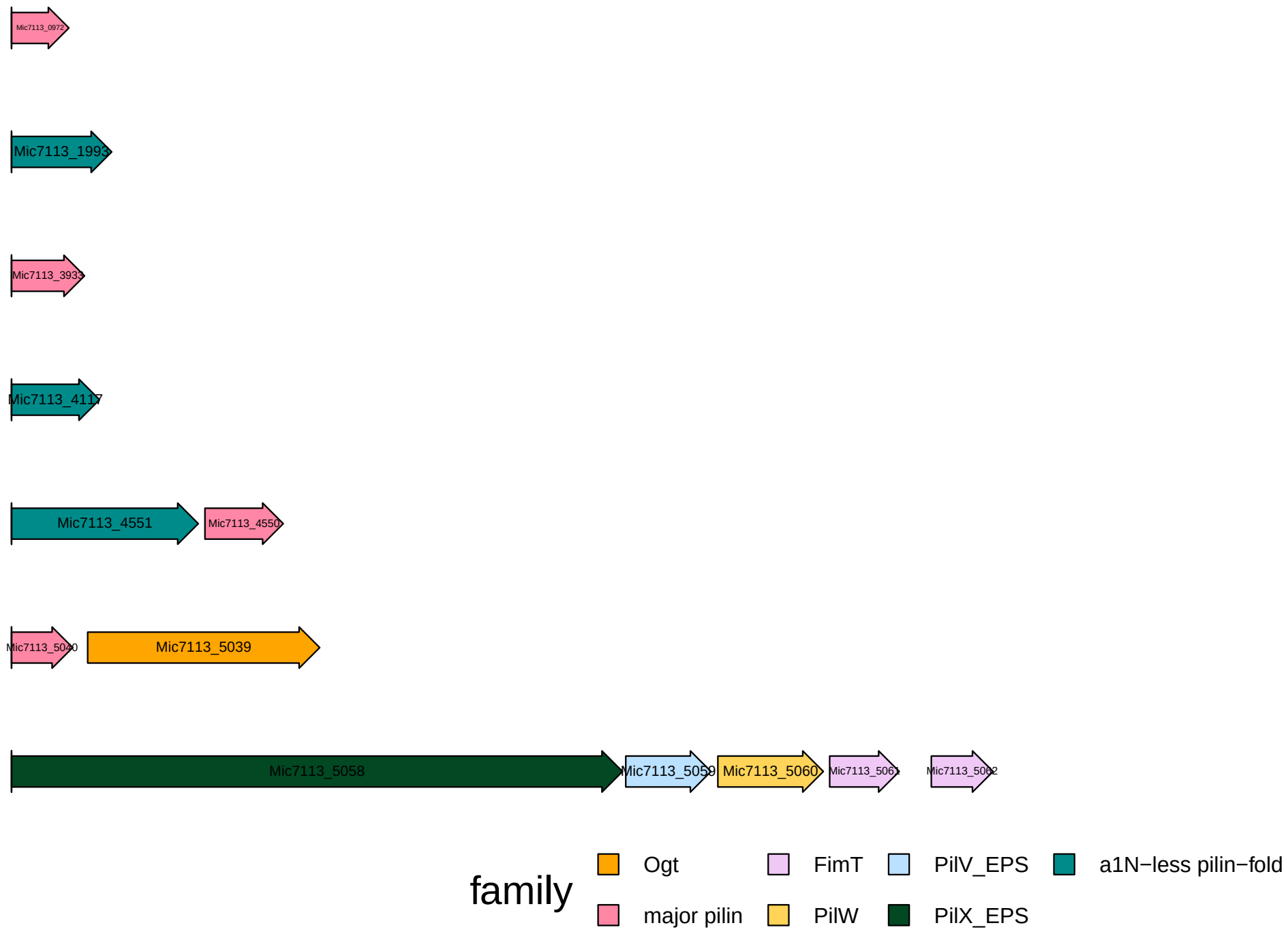

locus	gene	family	start	end
555	Mic7113_0972	major pilin	1125649	1126200
559	Mic7113_1993	a1N−less pilin−fold	2326855	2327817
563	Mic7113_3933	major pilin	4559682	4560383
564	Mic7113_4117	a1N−less pilin−fold	4808170	4809015
568	Mic7113_4550	major pilin	5326616	5327368
568	Mic7113_4551	a1N−less pilin−fold	5327432	5329225
572	Mic7113_5039	Ogt	5887000	5889228
572	Mic7113_5040	major pilin	5889372	5889959
573	Mic7113_5058	PilX_EPS	5915429	5921296
573	Mic7113_5059	PilV_EPS	5921327	5922145
573	Mic7113_5060	PilW	5922211	5923224
573	Mic7113_5061	FimT	5923285	5923953
573	Mic7113_5062	FimT	5924261	5924854

Anabaena cylindrica (strain ATCC 27899 / PCC 7122)

GCA_000317695.1

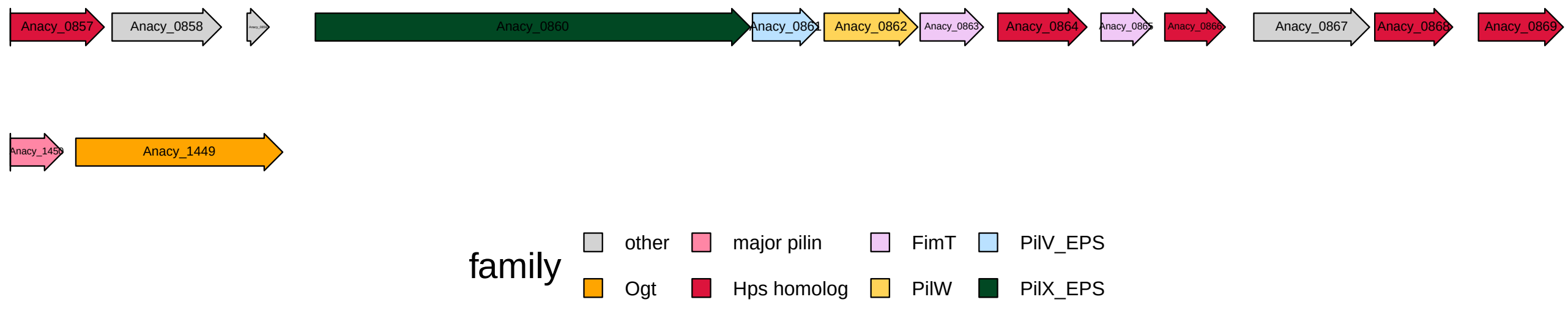

locus	gene	family	start	end
656	Anacy_0857	Hps homolog	1025414	1026418
656	Anacy_0858	other	1026502	1027671
656	Anacy_0859	other	1027946	1028182
656	Anacy_0860	PilX_EPS	1028676	1033328
656	Anacy_0861	PilV_EPS	1033352	1034059
656	Anacy_0862	PilW	1034119	1035120
656	Anacy_0863	FimT	1035145	1035822
656	Anacy_0864	Hps homolog	1035976	1036929
656	Anacy_0865	FimT	1037080	1037619
656	Anacy_0866	Hps homolog	1037762	1038409
656	Anacy_0867	other	1038714	1039952
656	Anacy_0868	Hps homolog	1040008	1040841
656	Anacy_0869	Hps homolog	1041117	1042025
658	Anacy_1449	Ogt	1698430	1700643
658	Anacy_1450	major pilin	1700780	1701343

Aphanothece hegewaldii CCALA 016

GCA_003007785.1

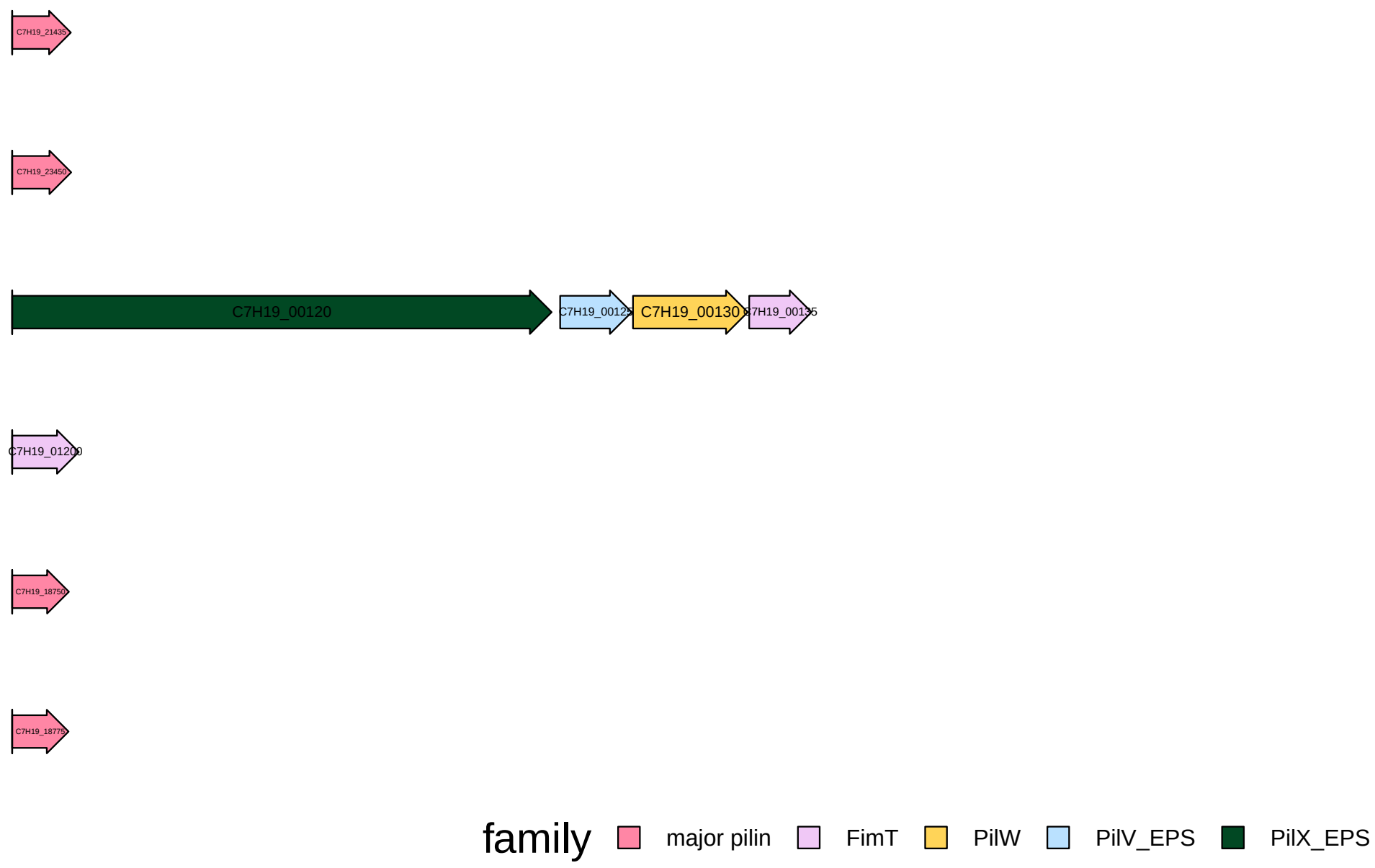

locus	gene	family	start	end
1300	C7H19_21435	major pilin	6976	7509
1309	C7H19_23450	major pilin	3134	3670
1314	C7H19_00120	PilX_EPS	24666	29567
1314	C7H19_00125	PilV_EPS	29644	30294
1314	C7H19_00130	PilW	30306	31349
1314	C7H19_00135	FimT	31362	31928
1317	C7H19_01200	FimT	258822	259427
1318	C7H19_18750	major pilin	12057	12572
1319	C7H19_18775	major pilin	15269	15781

Aphanothece sacrum FPU1

GCA_003864295.1

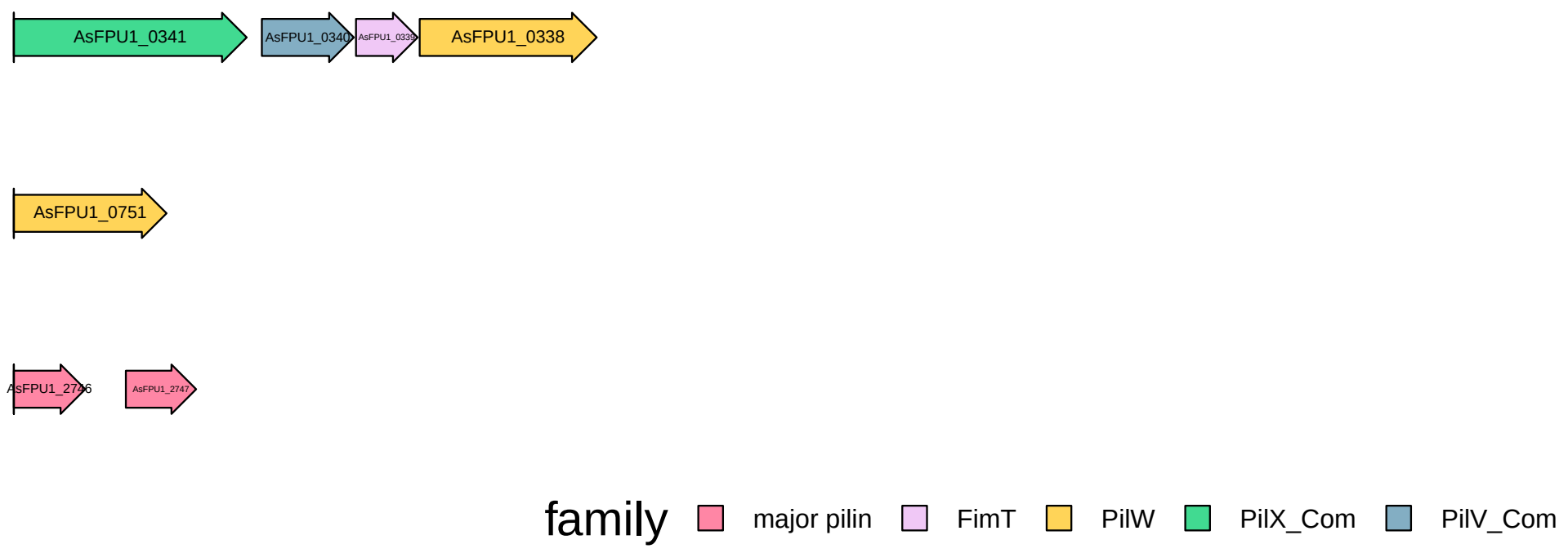

locus	gene	family	start	end
1422	AsFPU1_0338	PilW	387496	388914
1422	AsFPU1_0339	FimT	388930	389424
1422	AsFPU1_0340	PilV_Com	389441	390178
1422	AsFPU1_0341	PilX_Com	390299	392164
1425	AsFPU1_0751	PilW	828490	829713
1431	AsFPU1_2746	major pilin	704399	704971
1431	AsFPU1_2747	major pilin	705296	705859

Brasilonema sennae CENA114

GCA_006968745.1

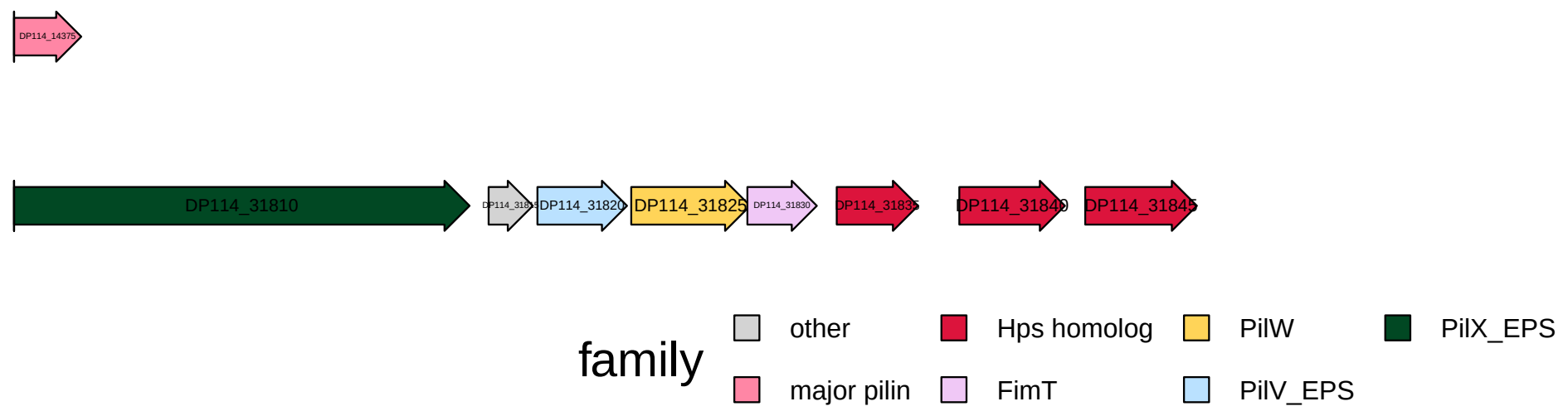

locus	gene	family	start	end
1528	DP114_14375	major pilin	3356157	3356687
1538	DP114_31810	PilX_EPS	7562173	7565766
1538	DP114_31815	other	7565916	7566265
1538	DP114_31820	PilV_EPS	7566301	7567008
1538	DP114_31825	PilW	7567041	7567979
1538	DP114_31830	FimT	7567957	7568505
1538	DP114_31835	Hps homolog	7568662	7569303
1538	DP114_31840	Hps homolog	7569629	7570462
1538	DP114_31845	Hps homolog	7570622	7571503

Calothrix elsteri CCALA 953

GCA_002289455.1

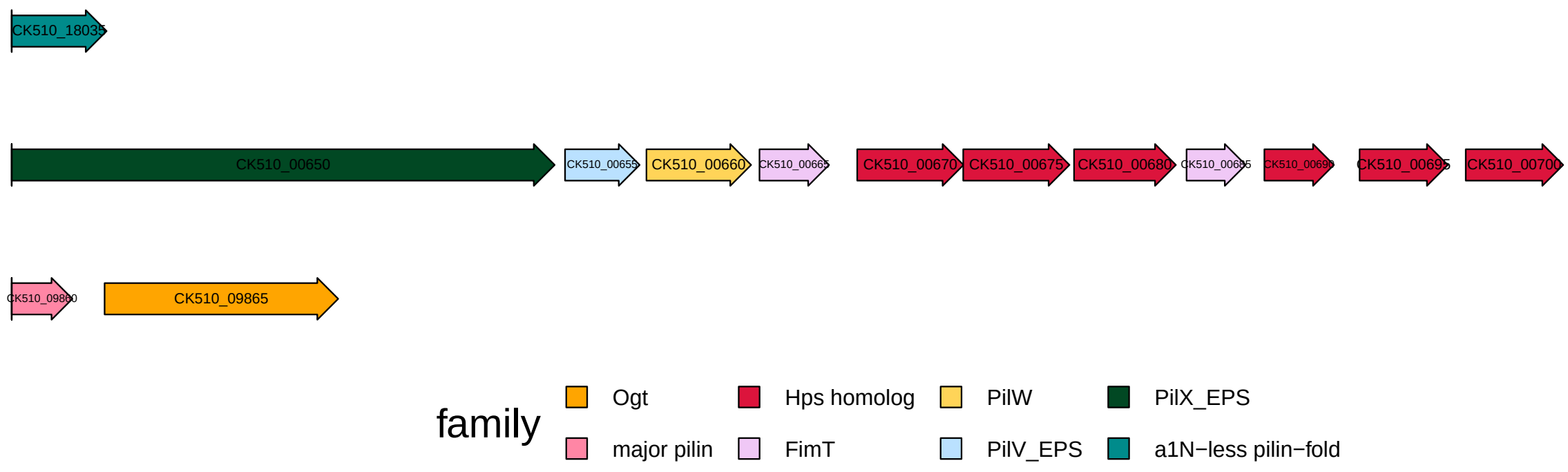

locus	gene	family	start	end
1083	CK510_18035	a1N−less pilin−fold	4454	5353
1084	CK510_00650	PilX_EPS	28541	33685
1084	CK510_00655	PilV_EPS	33783	34490
1084	CK510_00660	PilW	34554	35546
1084	CK510_00665	FimT	35625	36284
1084	CK510_00670	Hps homolog	36551	37558
1084	CK510_00675	Hps homolog	37555	38562
1084	CK510_00680	Hps homolog	38605	39570
1084	CK510_00685	FimT	39671	40231
1084	CK510_00690	Hps homolog	40408	41067
1084	CK510_00695	Hps homolog	41310	42146
1084	CK510_00700	Hps homolog	42316	43239
1095	CK510_09860	major pilin	4746	5321
1095	CK510_09865	Ogt	5626	7839

Candidatus Synechococcus spongiarum LMB bulk15N

GCA_002018015.1

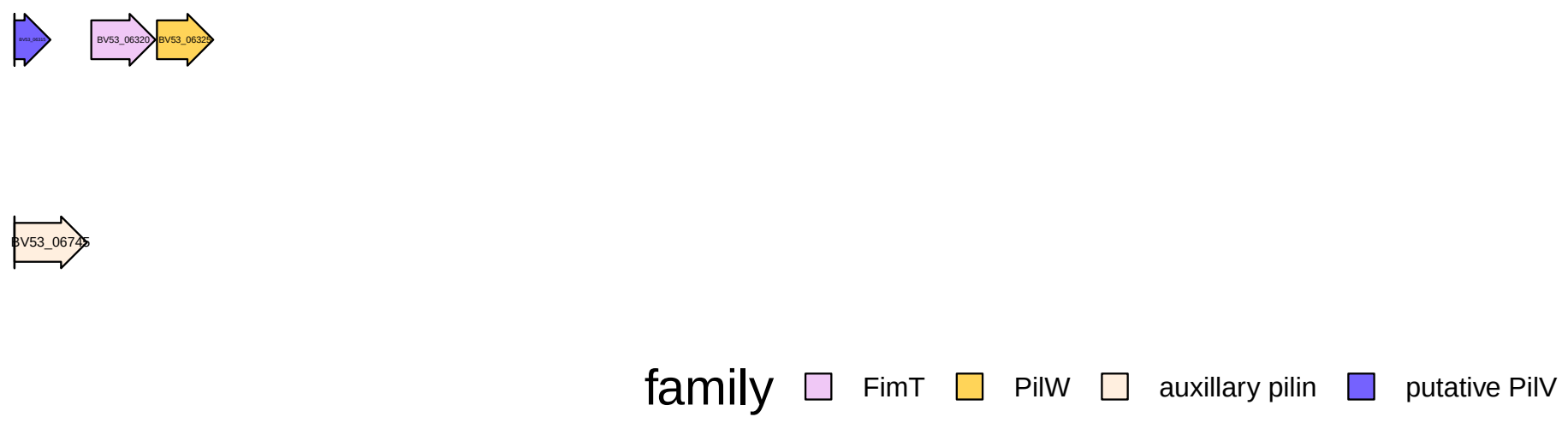

locus	gene	family	start	end
1054	BV53_06315	putative PilV	26355	26630
1054	BV53_06320	FimT	26942	27433
1054	BV53_06325	PilW	27444	27875
1055	BV53_06745	auxillary pilin	519	1073

Chamaesiphon minutus (strain ATCC 27169 / PCC 6605)

GCA_000317145.1

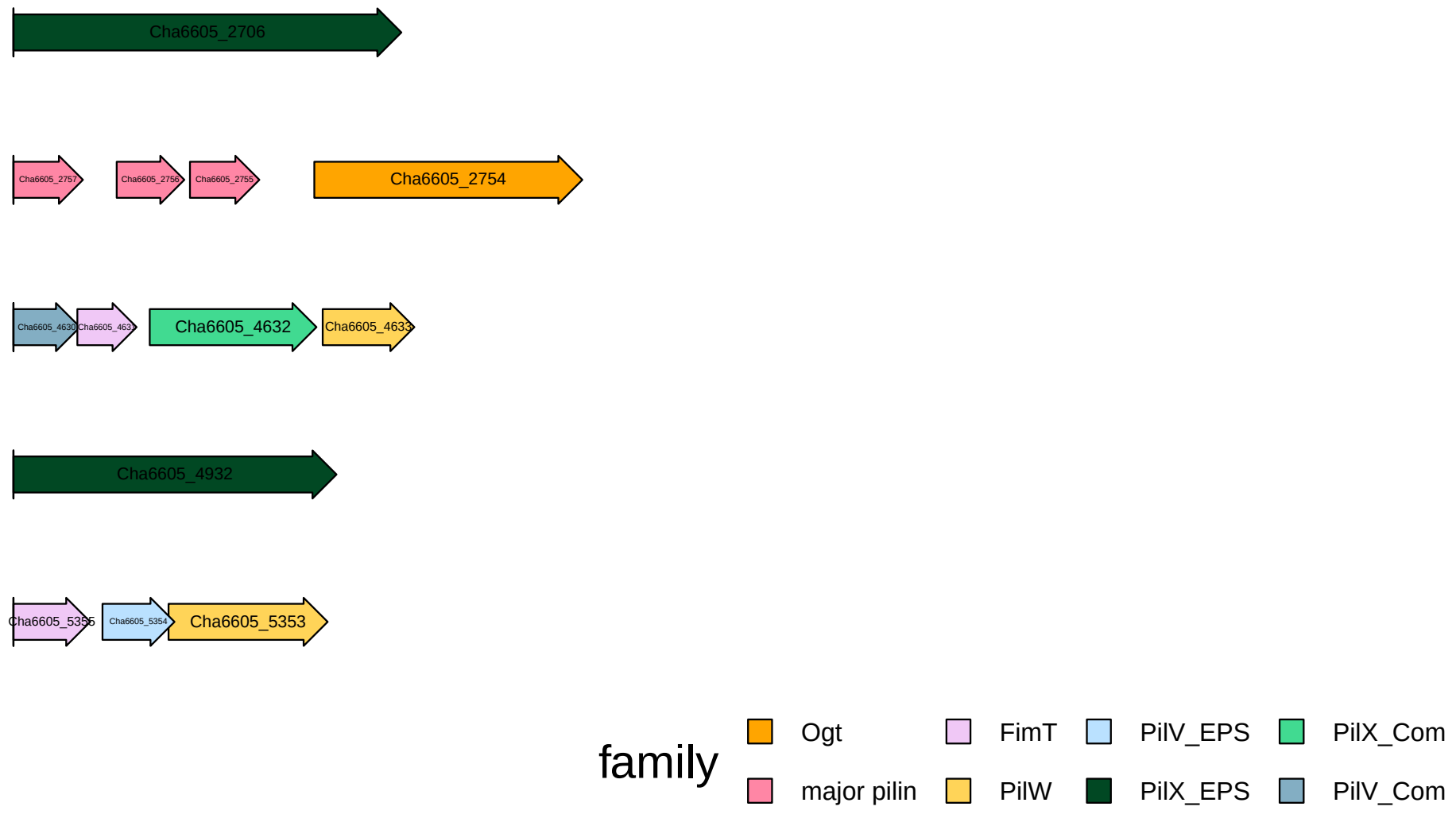

locus	gene	family	start	end
494	Cha6605_2706	PilX_EPS	2809046	2812240
495	Cha6605_2754	Ogt	2863179	2865386
495	Cha6605_2755	major pilin	2865838	2866410
495	Cha6605_2756	major pilin	2866456	2867013
495	Cha6605_2757	major pilin	2867291	2867863
502	Cha6605_4630	PilV_Com	4910054	4910602
502	Cha6605_4631	FimT	4910580	4911068
502	Cha6605_4632	PilX_Com	4911176	4912549
502	Cha6605_4633	PilW	4912600	4913355
505	Cha6605_4932	PilX_EPS	5225804	5228464
507	Cha6605_5353	PilW	5658401	5659711
507	Cha6605_5354	PilV_EPS	5659662	5660255
507	Cha6605_5355	FimT	5660356	5660988

Chamaesiphon polymorphus CCALA 037

GCA_003003845.1

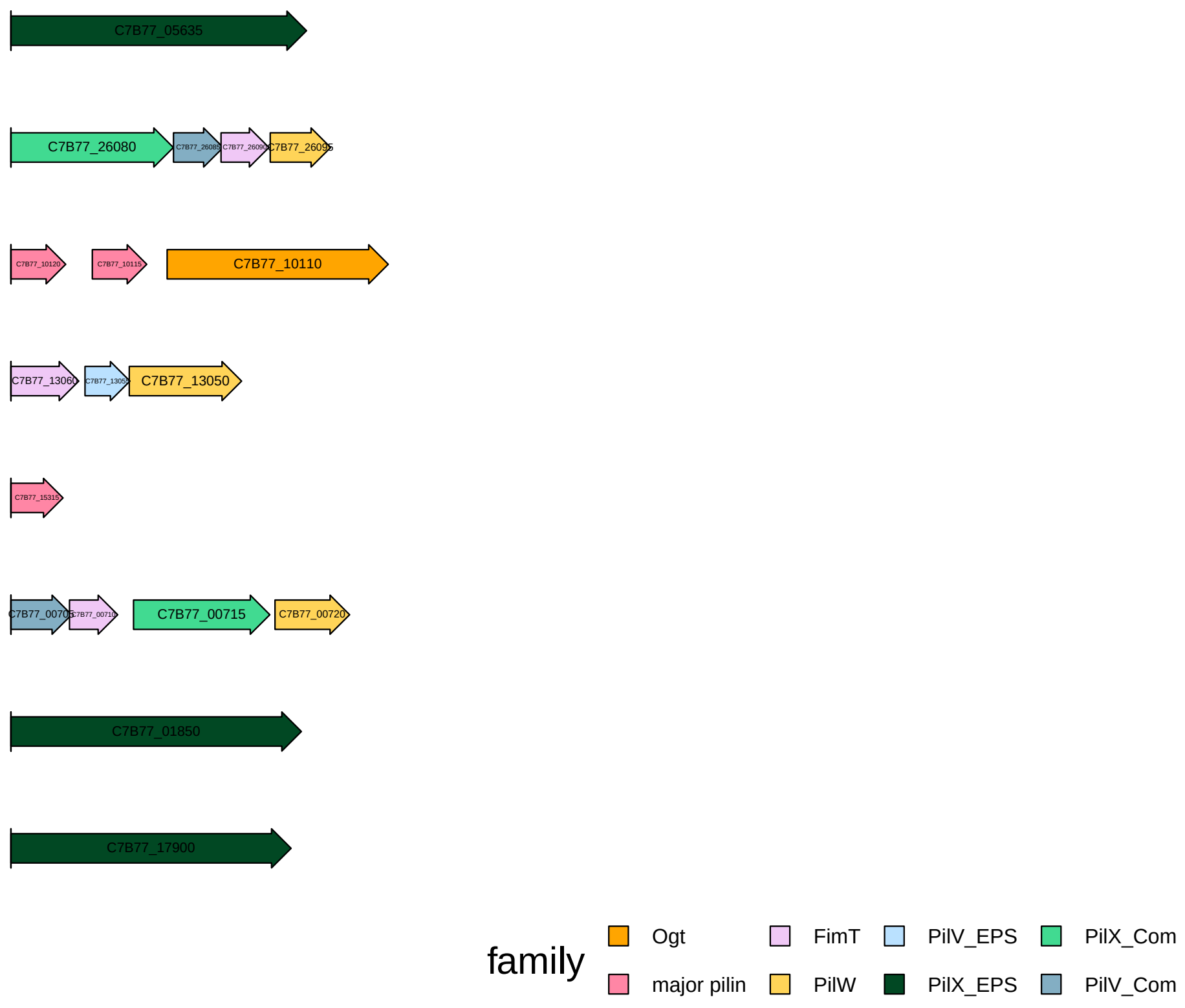

locus	gene	family	start	end
1273	C7B77_05635	PilX_EPS	7018	10008
1279	C7B77_26080	PilX_Com	254	1897
1279	C7B77_26085	PilV_Com	1898	2401
1279	C7B77_26090	FimT	2379	2864
1279	C7B77_26095	PilW	2877	3488
1280	C7B77_10110	Ogt	9196	11433
1280	C7B77_10115	major pilin	11639	12190
1280	C7B77_10120	major pilin	12459	13013
1284	C7B77_13050	PilW	4162	5298
1284	C7B77_13055	PilV_EPS	5291	5746
1284	C7B77_13060	FimT	5809	6495
1286	C7B77_15315	major pilin	4887	5414
1287	C7B77_00705	PilV_Com	8452	9066
1287	C7B77_00710	FimT	9044	9532
1287	C7B77_00715	PilX_Com	9692	11071
1287	C7B77_00720	PilW	11123	11878
1289	C7B77_01850	PilX_EPS	1	2939
1291	C7B77_17900	PilX_EPS	5274	8108

Chlorogloeopsis fritschii PCC 6912

GCA_003990575.1

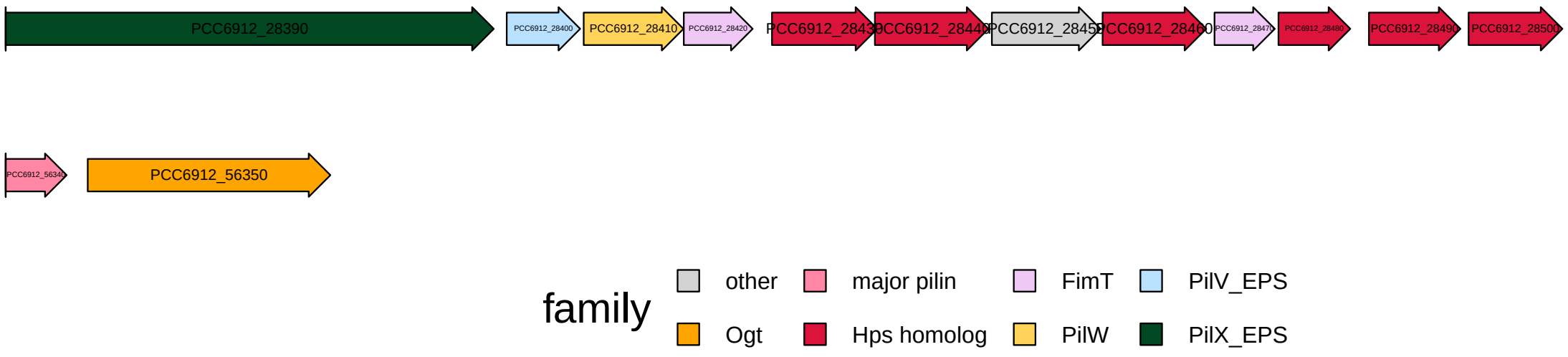

locus	gene	family	start	end
1447	PCC6912_28390	PilX_EPS	63109	67566
1447	PCC6912_28400	PilV_EPS	67684	68355
1447	PCC6912_28410	PilW	68387	69298
1447	PCC6912_28420	FimT	69301	69930
1447	PCC6912_28430	Hps homolog	70106	71068
1447	PCC6912_28440	Hps homolog	71046	72083
1447	PCC6912_28450	other	72114	73106
1447	PCC6912_28460	Hps homolog	73125	74072
1447	PCC6912_28470	FimT	74147	74698
1447	PCC6912_28480	Hps homolog	74731	75387
1447	PCC6912_28490	Hps homolog	75557	76393
1447	PCC6912_28500	Hps homolog	76467	77324
1453	PCC6912_56340	major pilin	7841	8398
1453	PCC6912_56350	Ogt	8590	10806

Chroococcidiopsis thermalis (strain PCC 7203)

GCA_000317125.1

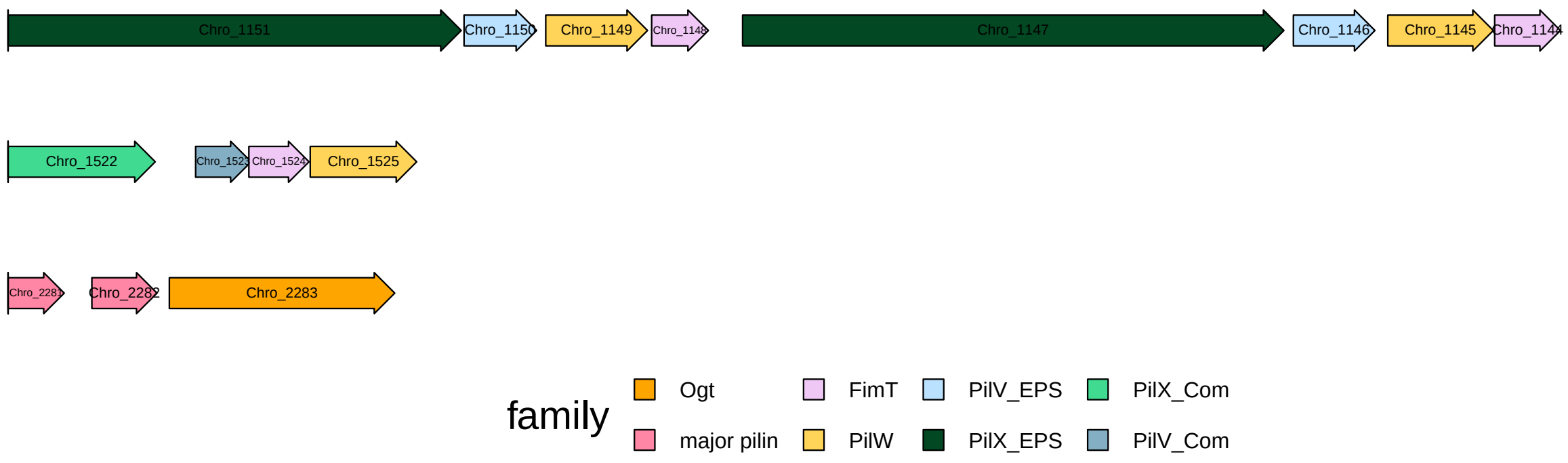

locus	gene	family	start	end
475	Chro_1144	FimT	1276778	1277413
475	Chro_1145	PilW	1277426	1278445
475	Chro_1146	PilV_EPS	1278569	1279357
475	Chro_1147	PilX_EPS	1279448	1284676
475	Chro_1148	FimT	1285006	1285554
475	Chro_1149	PilW	1285594	1286577
475	Chro_1150	PilV_EPS	1286665	1287366
475	Chro_1151	PilX_EPS	1287393	1291769
476	Chro_1522	PilX_Com	1698048	1699469
476	Chro_1523	PilV_Com	1699859	1700395
476	Chro_1524	FimT	1700373	1700954
476	Chro_1525	PilW	1700966	1701994
478	Chro_2281	major pilin	2554893	2555435
478	Chro_2282	major pilin	2555702	2556331
478	Chro_2283	Ogt	2556450	2558627

Chroogloeocystis siderophila 5.2 s.c.1

GCA_001904655.1

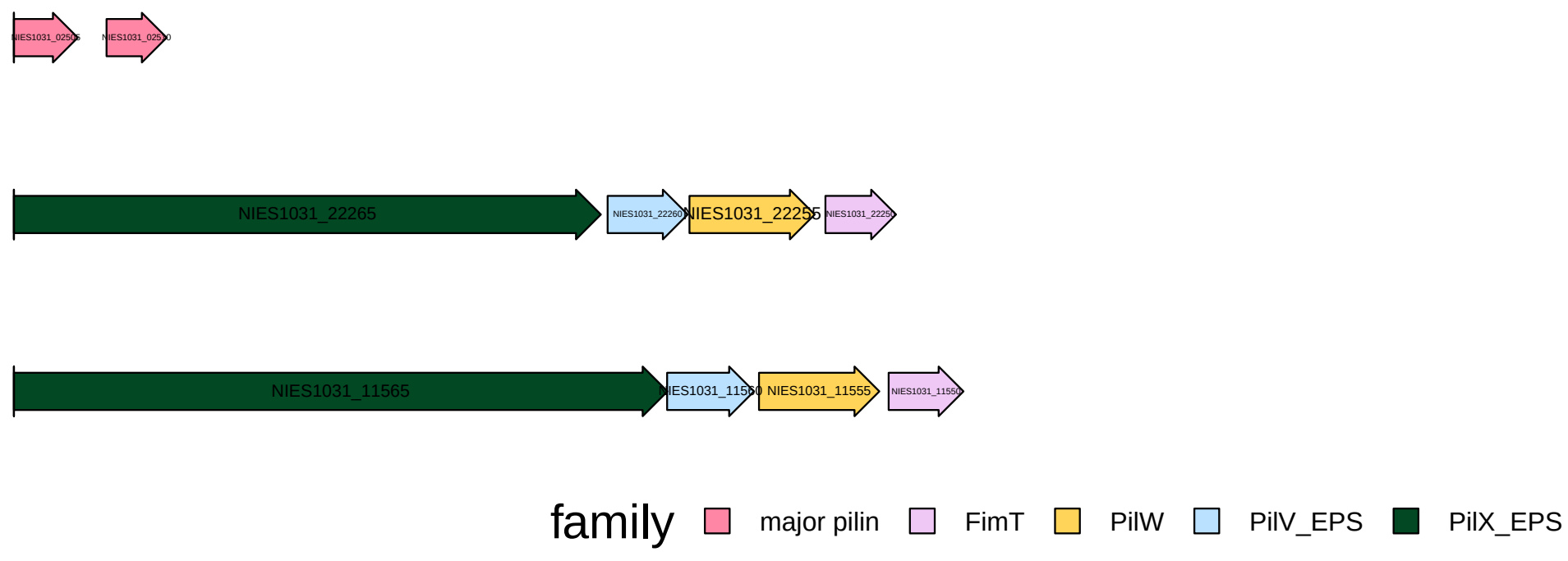

locus	gene	family	start	end
977	NIES1031_02505	major pilin	140622	141131
977	NIES1031_02510	major pilin	141360	141839
980	NIES1031_22250	FimT	994	1557
980	NIES1031_22255	PilW	1642	2640
980	NIES1031_22260	PilV_EPS	2654	3292
980	NIES1031_22265	PilX_EPS	3346	8022
986	NIES1031_11550	FimT	154400	154996
986	NIES1031_11555	PilW	155070	156029
986	NIES1031_11560	PilV_EPS	156069	156761
986	NIES1031_11565	PilX_EPS	156758	161965

Coleofasciculus chthonoplastes PCC 7420

GCA_000155555.1

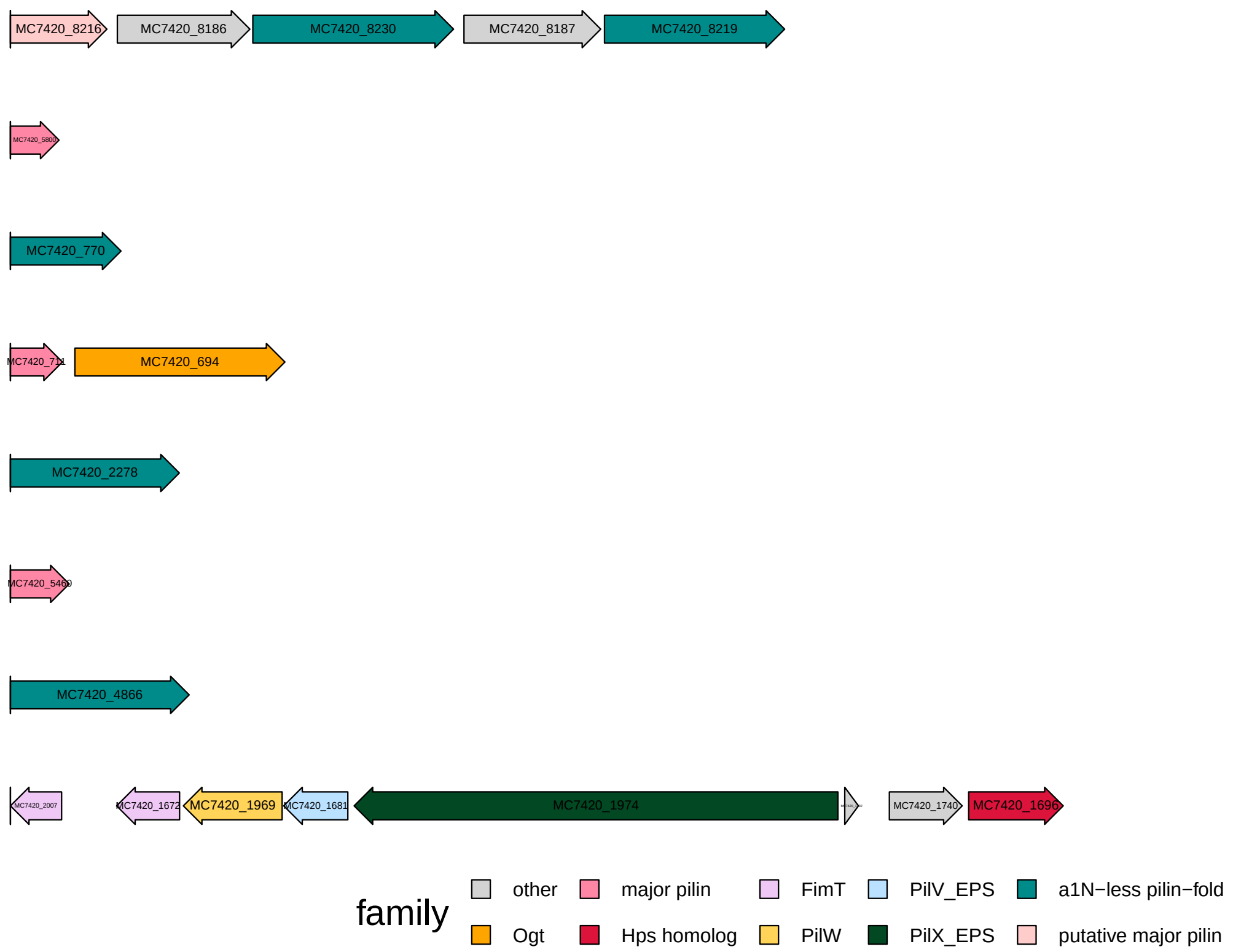

locus	gene	family	start	end
319	MC7420_8219	a1N−less pilin−fold	53935	55854
319	MC7420_8187	other	55894	57351
319	MC7420_8230	a1N−less pilin−fold	57462	59600
319	MC7420_8186	other	59631	61043
319	MC7420_8216	putative major pilin	61154	62182
326	MC7420_5800	major pilin	47174	47692
329	MC7420_770	a1N−less pilin−fold	164827	166005
331	MC7420_694	Ogt	258470	260707
331	MC7420_711	major pilin	260840	261394
333	MC7420_2278	a1N−less pilin−fold	130612	132411
337	MC7420_5460	major pilin	170859	171485
338	MC7420_4866	a1N−less pilin−fold	178101	180005
341	MC7420_1696	Hps homolog	177569	178576
341	MC7420_1740	other	178645	179421
341	MC7420_1702	other	179750	179896
341	MC7420_1974	PilX_EPS	179969	185122
341	MC7420_1681	PilV_EPS	185189	185872
341	MC7420_1969	PilW	185889	186941
341	MC7420_1672	FimT	186981	187652
341	MC7420_2007	FimT	188238	188783

Crinalium epipsammum PCC 9333

GCA_000317495.1

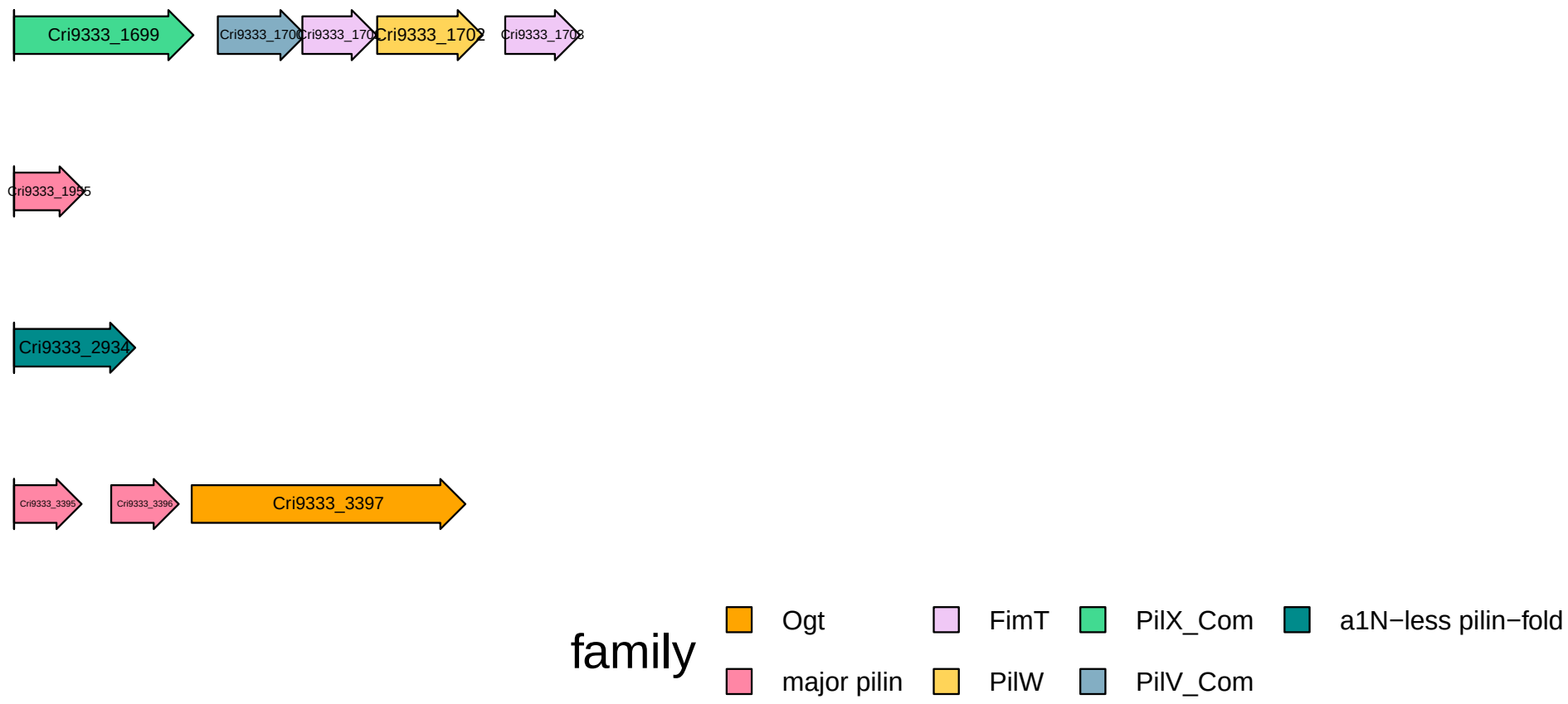

locus	gene	family	start	end
539	Cri9333_1699	PilX_Com	1943648	1945063
539	Cri9333_1700	PilV_Com	1945255	1945947
539	Cri9333_1701	FimT	1945922	1946509
539	Cri9333_1702	PilW	1946512	1947345
539	Cri9333_1703	FimT	1947522	1948112
541	Cri9333_1955	major pilin	2217626	2218183
545	Cri9333_2934	a1N−less pilin−fold	3287623	3288579
546	Cri9333_3395	major pilin	3805962	3806495
546	Cri9333_3396	major pilin	3806728	3807261
546	Cri9333_3397	Ogt	3807363	3809522

Crocosphaera chwakensis CCY0110

GCA_000169335.1

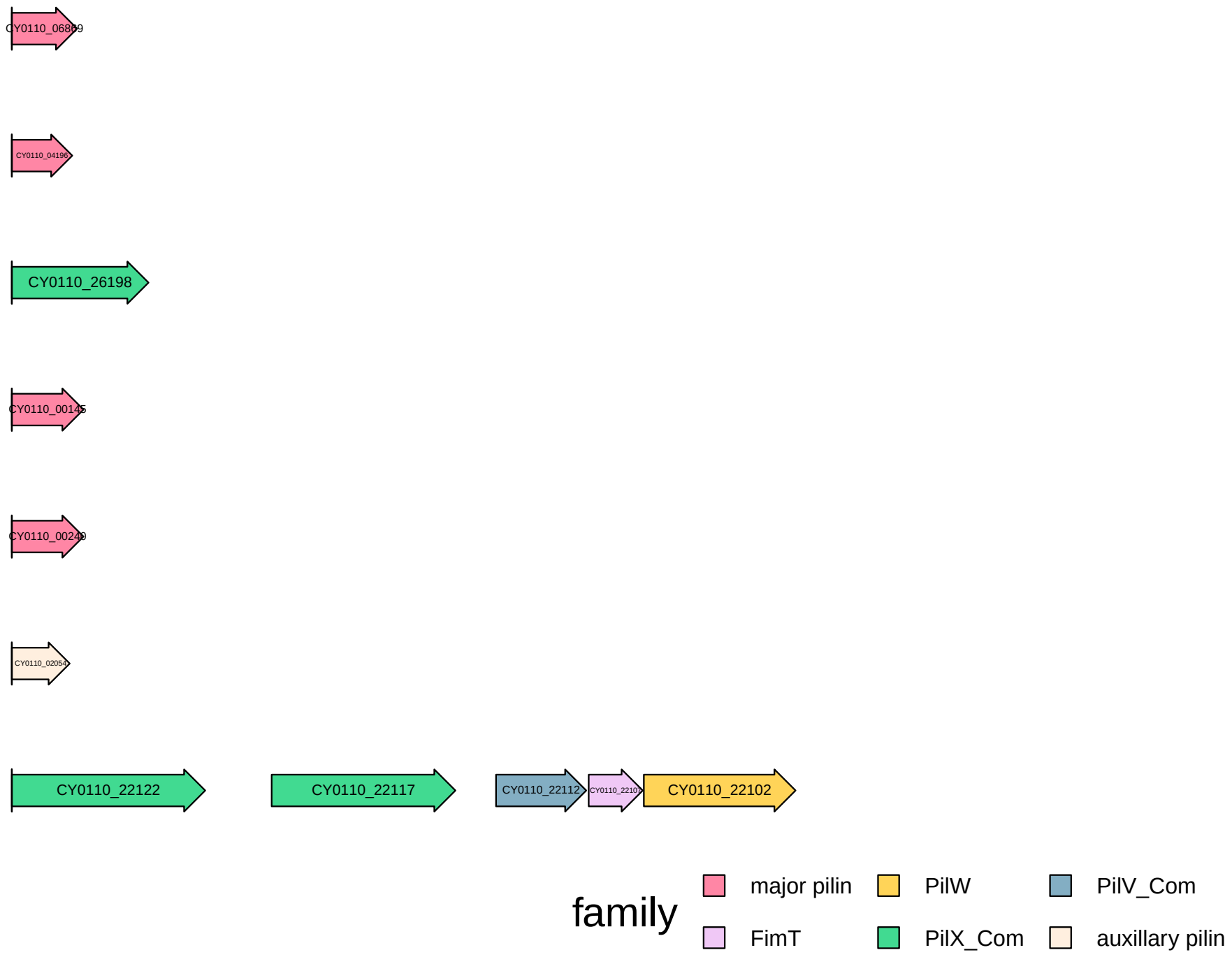

locus	gene	family	start	end
343	CY0110_06869	major pilin	5182	5793
346	CY0110_04196	major pilin	35012	35578
356	CY0110_26198	PilX_Com	79173	80453
357	CY0110_00145	major pilin	27069	27740
358	CY0110_00240	major pilin	41737	42408
361	CY0110_02054	auxillary pilin	89835	90377
362	CY0110_22102	PilW	85766	87187
362	CY0110_22107	FimT	87197	87703
362	CY0110_22112	PilV_Com	87726	88571
362	CY0110_22117	PilX_Com	88951	90672
362	CY0110_22122	PilX_Com	91294	93105

Crocosphaera subtropica (strain ATCC 51142 / BH68) (Cyanothece sp. (strain ATCC 51142))

GCA_000017845.1

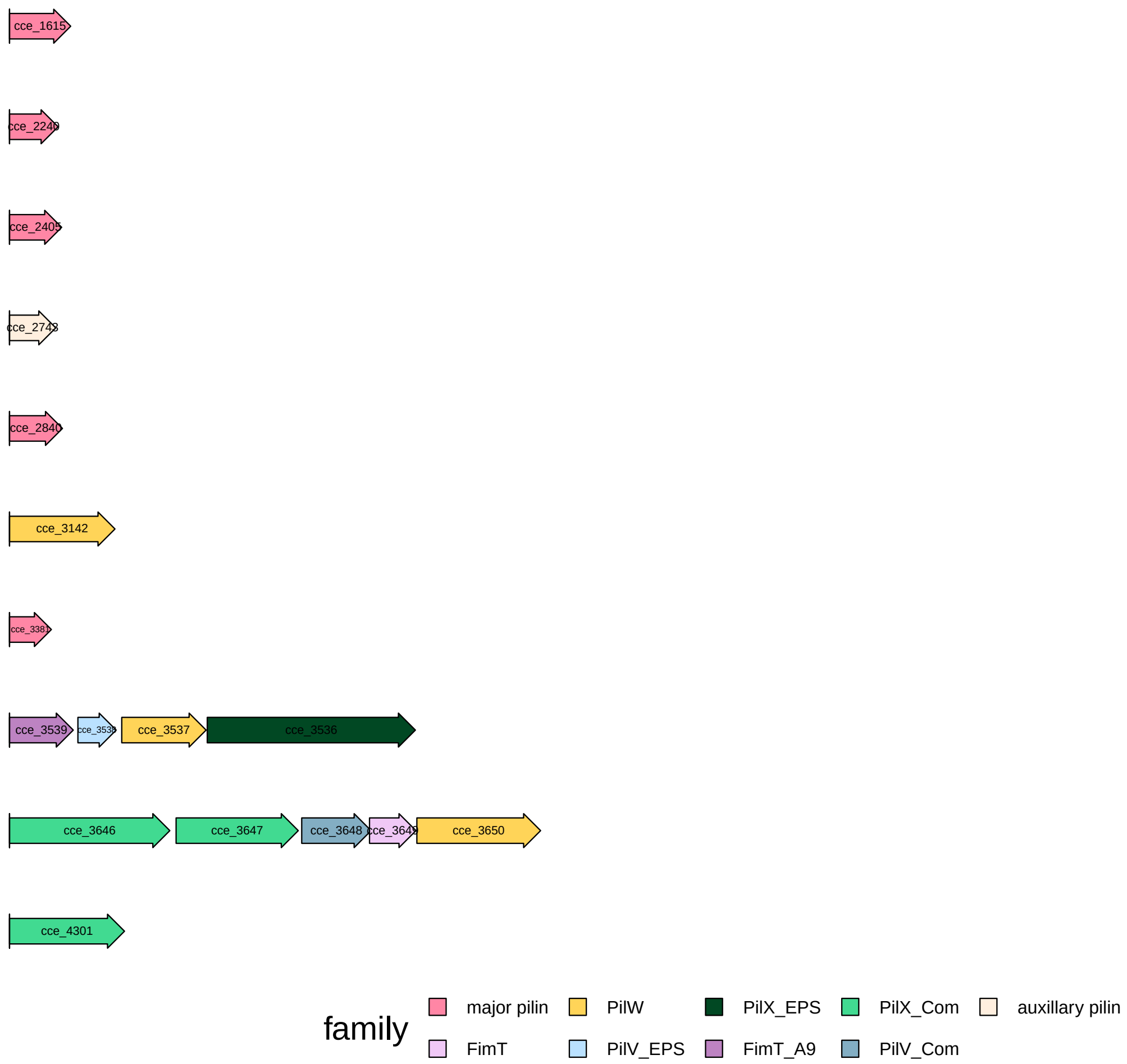

locus	gene	family	start	end
157	cce_1615	major pilin	1626474	1627187
160	cce_2240	major pilin	2283583	2284149
161	cce_2405	major pilin	2459933	2460541
162	cce_2743	auxillary pilin	2802381	2802920
164	cce_2840	major pilin	2891796	2892413
167	cce_3142	PilW	3253624	3254853
168	cce_3381	major pilin	3480200	3480688
169	cce_3536	PilX_EPS	3664073	3666499
169	cce_3537	PilW	3666512	3667495
169	cce_3538	PilV_EPS	3667560	3668006
169	cce_3539	FimT_A9	3668060	3668803
170	cce_3646	PilX_Com	3776344	3778212
170	cce_3647	PilX_Com	3778285	3779709
170	cce_3648	PilV_Com	3779749	3780558
170	cce_3649	FimT	3780539	3781081
170	cce_3650	PilW	3781091	3782533
174	cce_4301	PilX_Com	4428356	4429696

Crocosphaera watsonii WH 0003

GCA_000235665.2

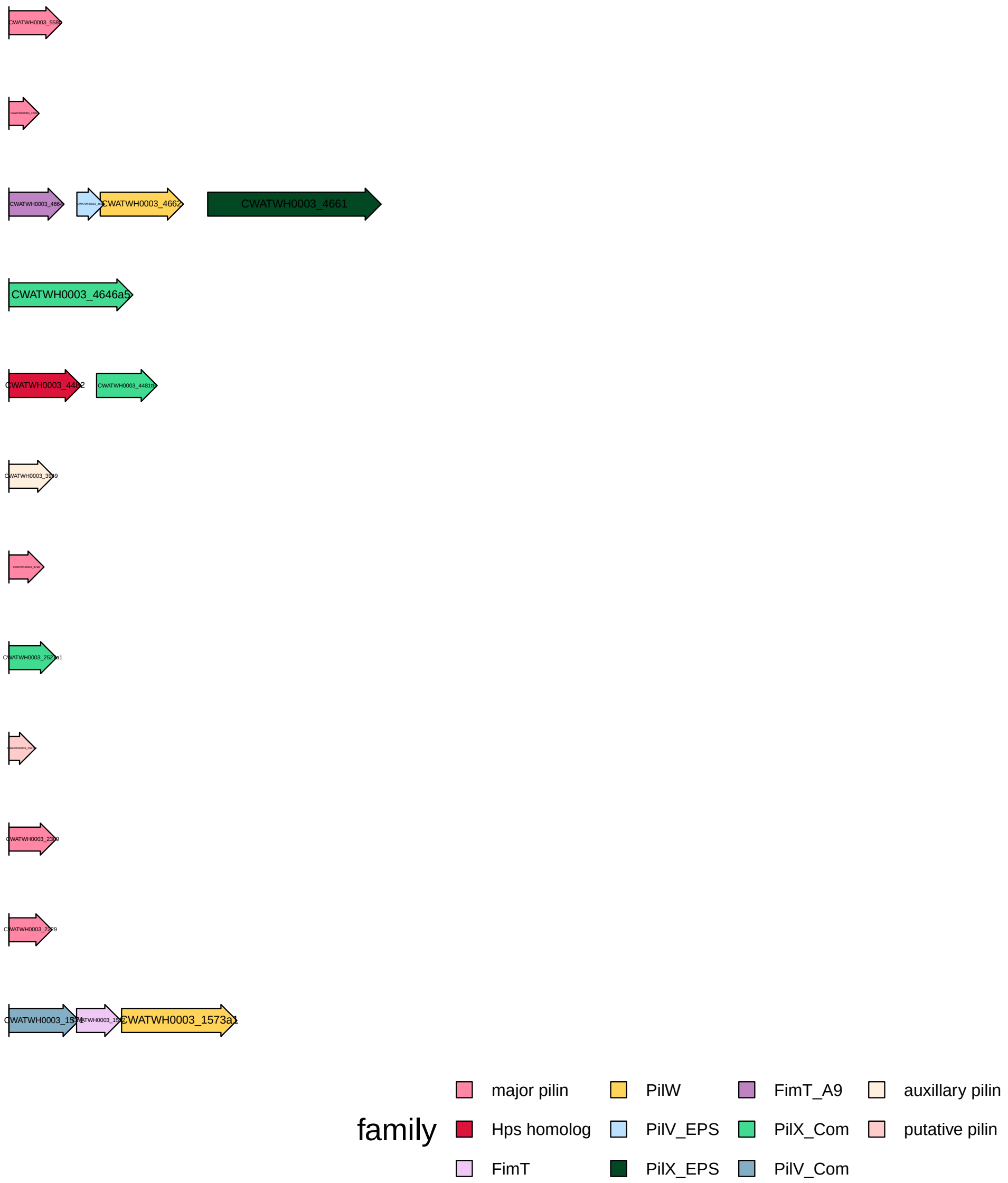

locus	gene	family	start	end
410	CWATWH0003_5580	major pilin	5146	5802
412	CWATWH0003_4703	major pilin	2172	2546
413	CWATWH0003_4661	PilX_EPS	1139	3289
413	CWATWH0003_4662	PilW	3590	4621
413	CWATWH0003_4663	PilV_EPS	4572	4910
413	CWATWH0003_4664	FimT_A9	5069	5752
414	CWATWH0003_4646a5	PilX_Com	1	1537
416	CWATWH0003_4481t6	PilX_Com	1	752
416	CWATWH0003_4482	Hps homolog	942	1838
418	CWATWH0003_3949	auxillary pilin	17134	17688
419	CWATWH0003_3798	major pilin	8452	8886
425	CWATWH0003_2527a1	PilX_Com	1	591
426	CWATWH0003_2527b1	putative pilin	11170	11499
427	CWATWH0003_2309	major pilin	1040	1627
428	CWATWH0003_2229	major pilin	7938	8471
432	CWATWH0003_1571	PilV_Com	237	1106
432	CWATWH0003_1572	FimT	1075	1623
432	CWATWH0003_1573a1	PilW	1633	3062

Cuspidothrix issatschenkoi CHARLIE−1

GCA_002934005.1

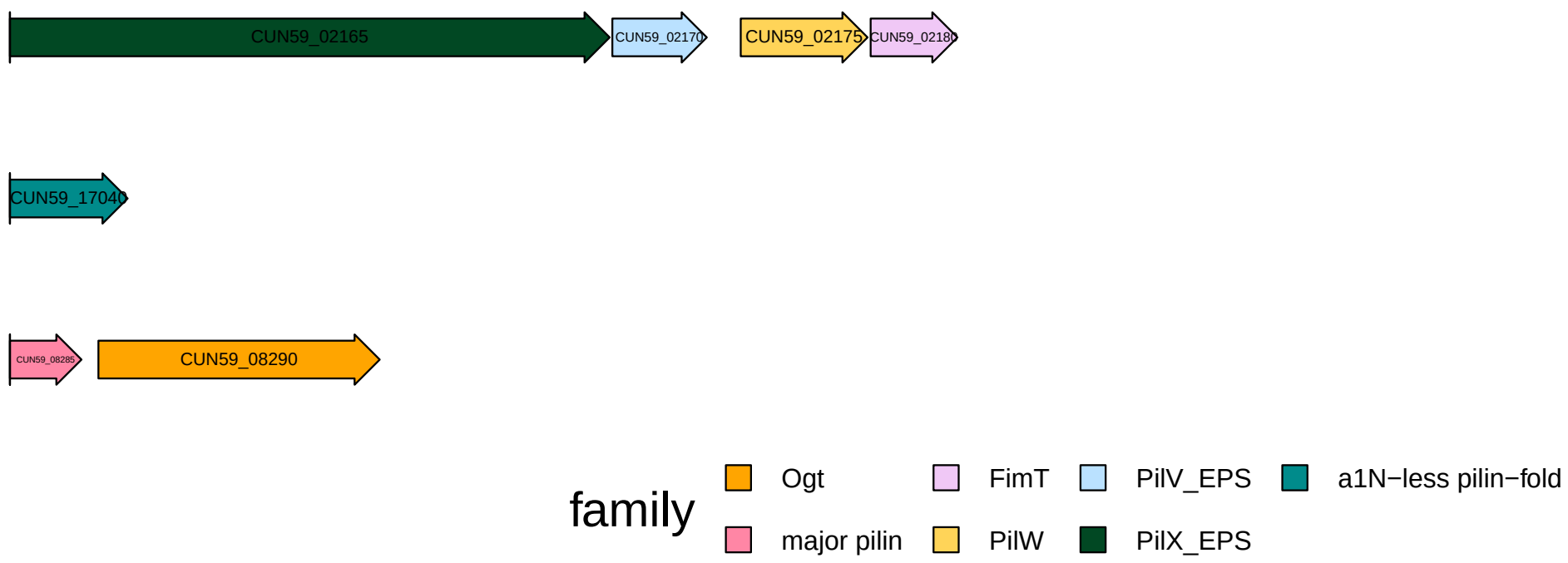

locus	gene	family	start	end
1185	CUN59_02165	PilX_EPS	30532	35250
1185	CUN59_02170	PilV_EPS	35271	36014
1185	CUN59_02175	PilW	36281	37279
1185	CUN59_02180	FimT	37303	37986
1186	CUN59_17040	a1N−less pilin−fold	25523	26449
1194	CUN59_08285	major pilin	37423	37986
1194	CUN59_08290	Ogt	38119	40332

Cyanobacterium aponinum (strain PCC 10605)

GCA_000317675.1

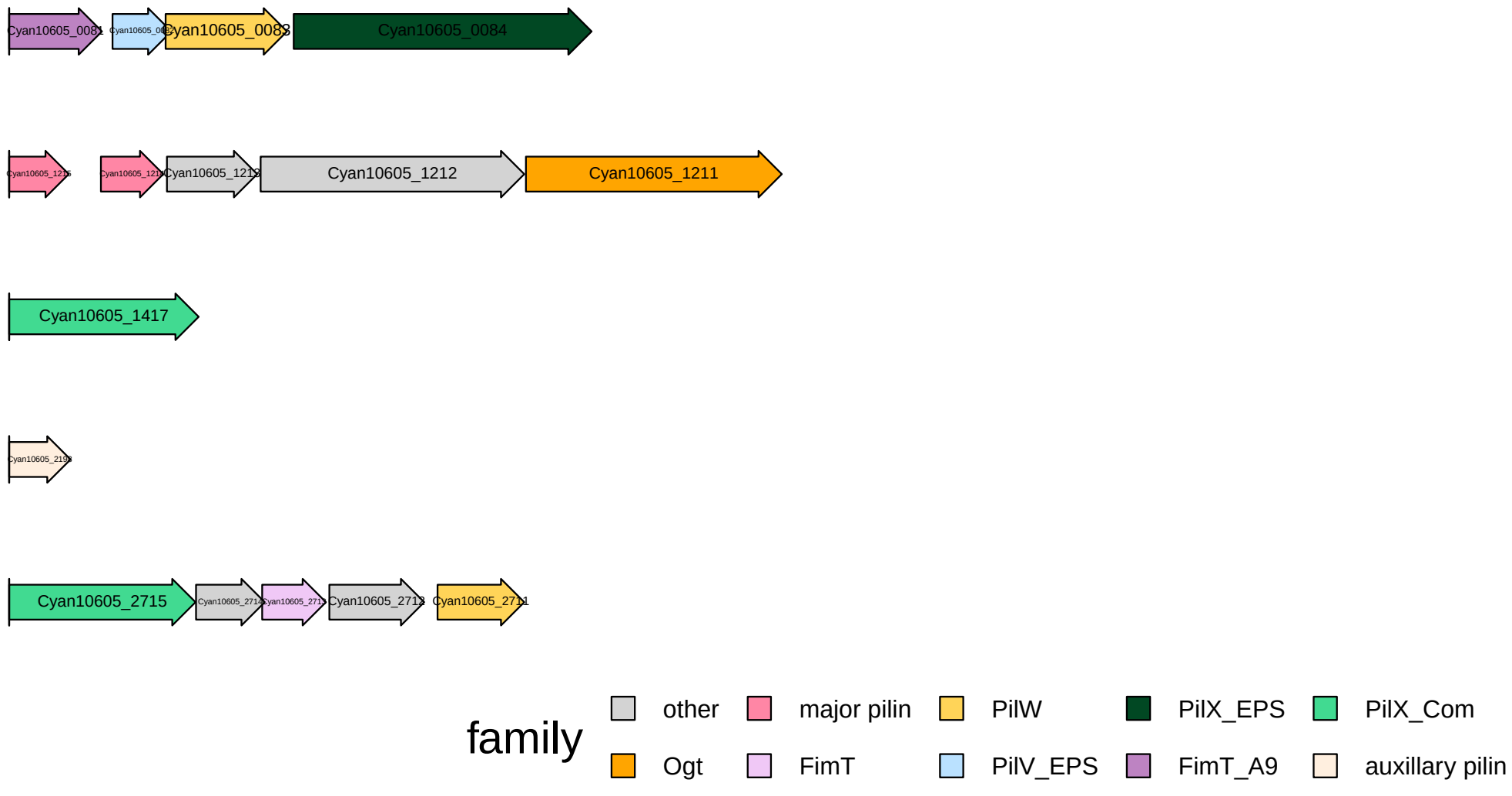

locus	gene	family	start	end
632	Cyan10605_0081	FimT_A9	96879	97667
632	Cyan10605_0082	PilV_EPS	97757	98257
632	Cyan10605_0083	PilW	98208	99239
632	Cyan10605_0084	PilX_EPS	99296	101827
641	Cyan10605_1211	Ogt	1458692	1460866
641	Cyan10605_1212	other	1460880	1463120
641	Cyan10605_1213	other	1463149	1463916
641	Cyan10605_1214	major pilin	1463947	1464477
641	Cyan10605_1215	major pilin	1464750	1465256
643	Cyan10605_1417	PilX_Com	1697424	1699034
646	Cyan10605_2198	auxillary pilin	2639691	2640212
649	Cyan10605_2711	PilW	3223383	3224120
649	Cyan10605_2712	other	3224233	3225039
649	Cyan10605_2713	FimT	3225068	3225610
649	Cyan10605_2714	other	3225588	3226172
649	Cyan10605_2715	PilX_Com	3226176	3227759

Cyanobium gracile (strain ATCC 27147 / PCC 6307)

GCA_000316515.1

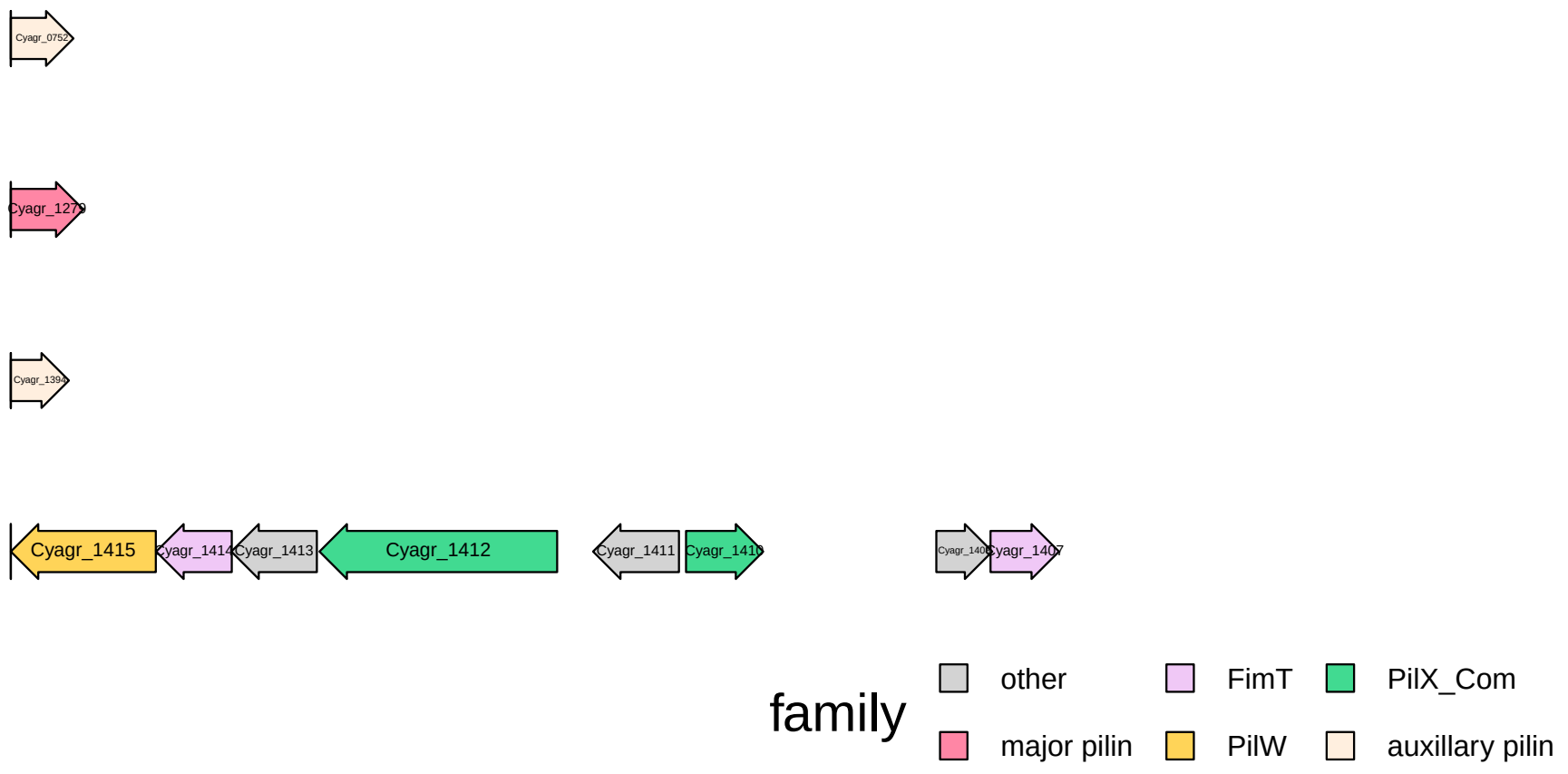

locus	gene	family	start	end
439	Cyagr_0752	auxillary pilin	774264	774716
441	Cyagr_1279	major pilin	1292196	1292720
443	Cyagr_1394	auxillary pilin	1398789	1399208
444	Cyagr_1407	FimT	1411657	1412151
444	Cyagr_1408	other	1412138	1412542
444	Cyagr_1410	PilX_Com	1413791	1414348
444	Cyagr_1411	other	1414399	1415019
444	Cyagr_1412	PilX_Com	1415278	1416993
444	Cyagr_1413	other	1417012	1417629
444	Cyagr_1414	FimT	1417626	1418171
444	Cyagr_1415	PilW	1418174	1419220

Cyanobium usitatum str. Tous

GCA_003011885.1

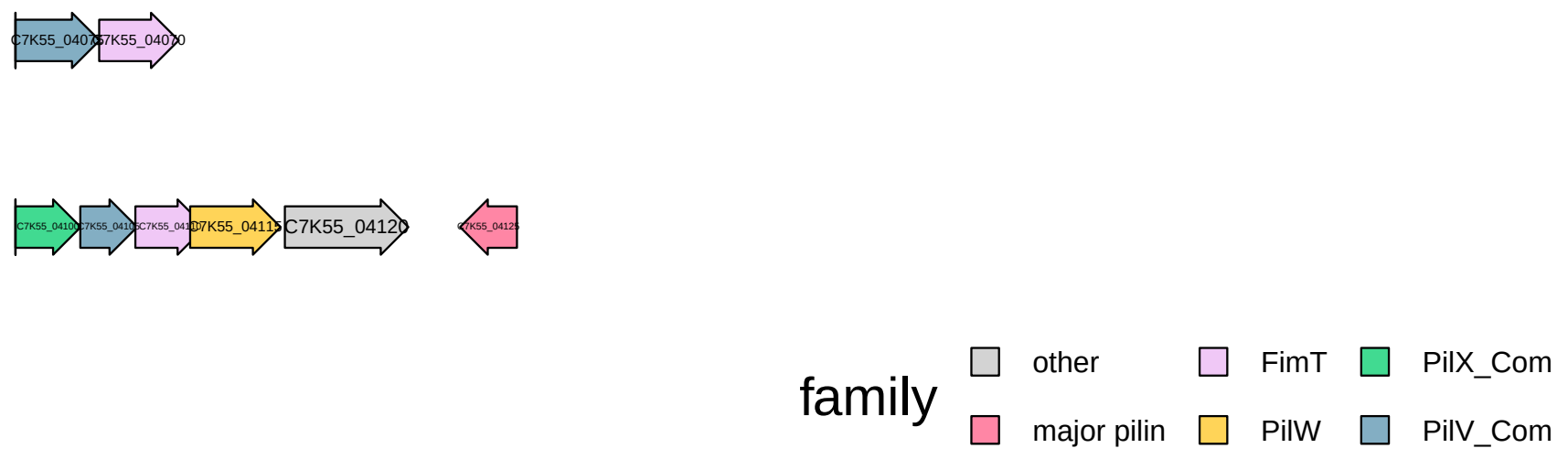

locus	gene	family	start	end
1326	C7K55_04070	FimT	160334	160903
1326	C7K55_04075	PilV_Com	160900	161502
1327	C7K55_04100	PilX_Com	166990	167457
1327	C7K55_04105	PilV_Com	167454	167861
1327	C7K55_04110	FimT	167848	168348
1327	C7K55_04115	PilW	168240	168887
1327	C7K55_04120	other	168918	169802
1327	C7K55_04125	major pilin	170172	170579

Cylindrospermopsis curvispora GIHE−G1

GCA_014489415.1

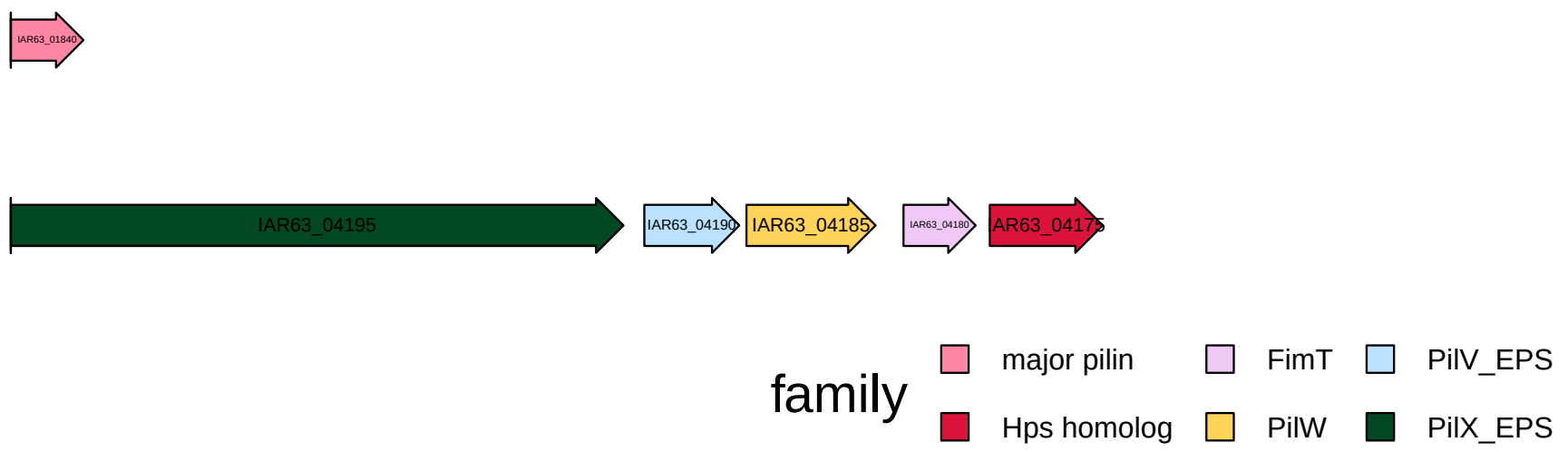

locus	gene	family	start	end
1654	IAR63_01840	major pilin	383166	383690
1655	IAR63_04175	Hps homolog	869838	870653
1655	IAR63_04180	FimT	870755	871276
1655	IAR63_04185	PilW	871477	872409
1655	IAR63_04190	PilV_EPS	872460	873146
1655	IAR63_04195	PilX_EPS	873296	877717

Cylindrospermum stagnale PCC 7417

GCA_000317535.1

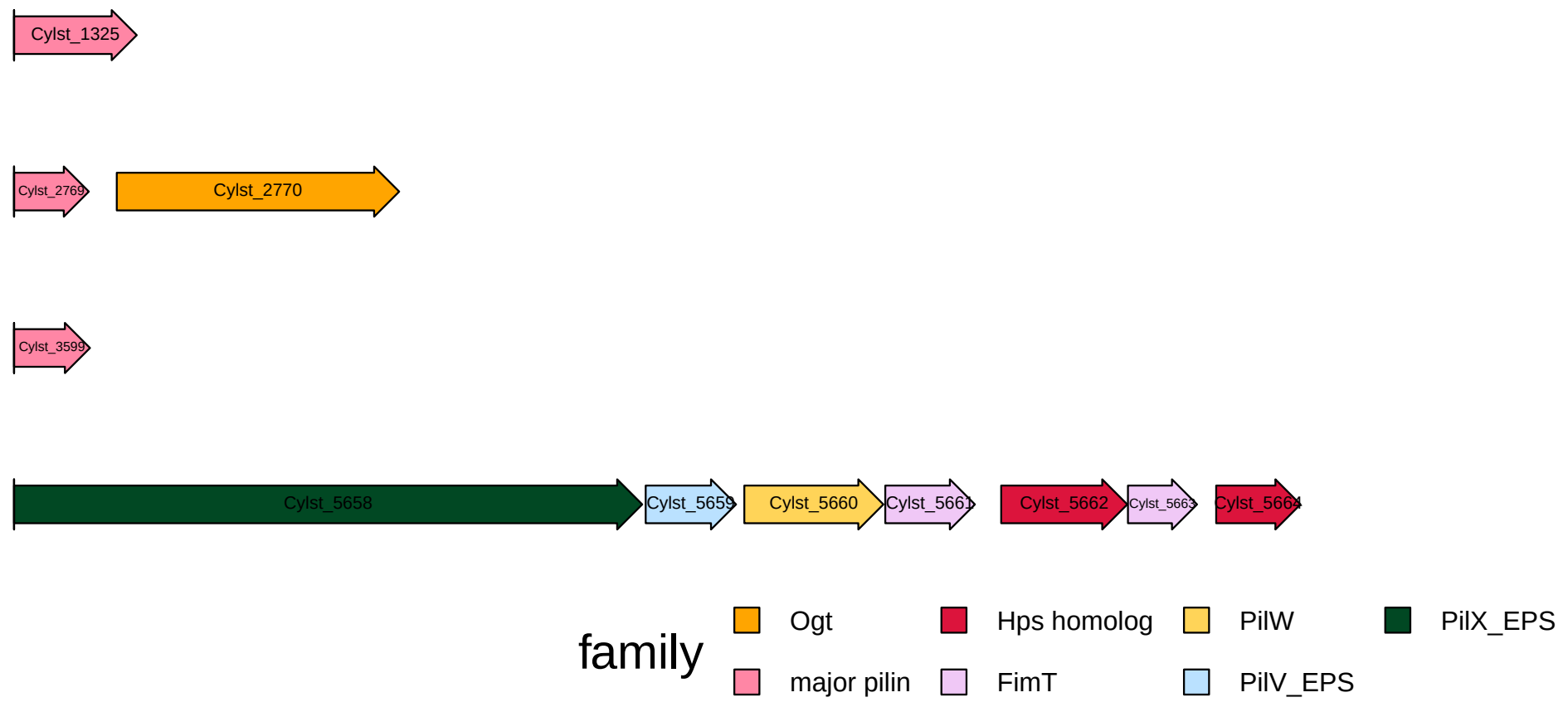

locus	gene	family	start	end
582	Cylst_1325	major pilin	1518031	1518999
586	Cylst_2769	major pilin	3242878	3243468
586	Cylst_2770	Ogt	3243688	3245916
587	Cylst_3599	major pilin	4102155	4102754
591	Cylst_5658	PilX_EPS	6356682	6361637
591	Cylst_5659	PilV_EPS	6361664	6362377
591	Cylst_5660	PilW	6362442	6363539
591	Cylst_5661	FimT	6363554	6364261
591	Cylst_5662	Hps homolog	6364468	6365463
591	Cylst_5663	FimT	6365468	6366013
591	Cylst_5664	Hps homolog	6366164	6366829

Dactylococcopsis salina (strain PCC 8305) (Myxobactron salinum)

GCA_000317615.1

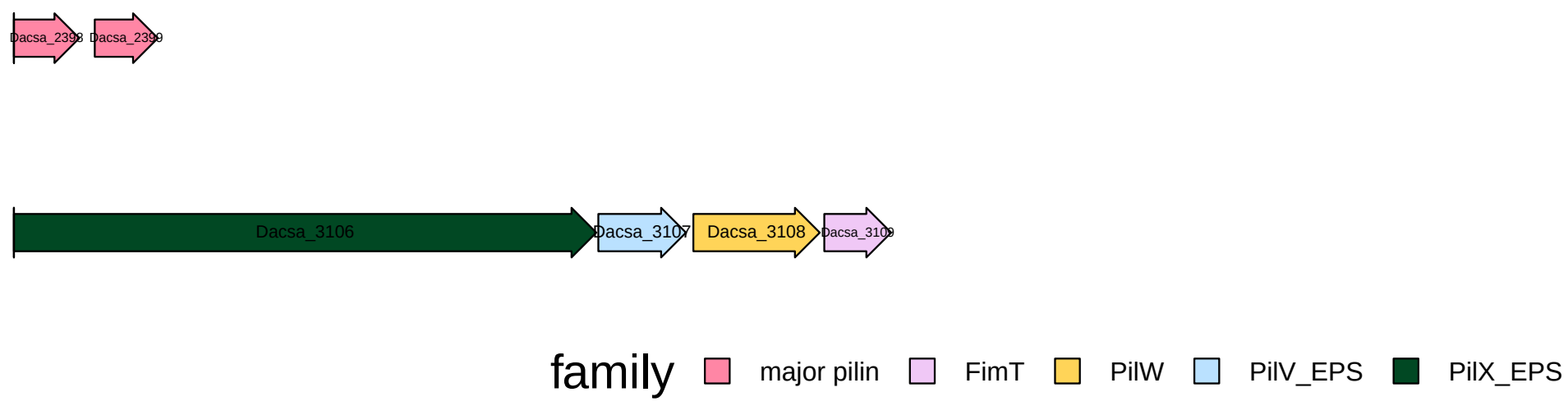

locus	gene	family	start	end
621	Dacsa_2398	major pilin	2436158	2436679
621	Dacsa_2399	major pilin	2436802	2437305
626	Dacsa_3106	PilX_EPS	3186373	3191013
626	Dacsa_3107	PilV_EPS	3191029	3191727
626	Dacsa_3108	PilW	3191786	3192793
626	Dacsa_3109	FimT	3192829	3193362

Desertifilum tharense IPPAS B−1220

GCA_001746915.1

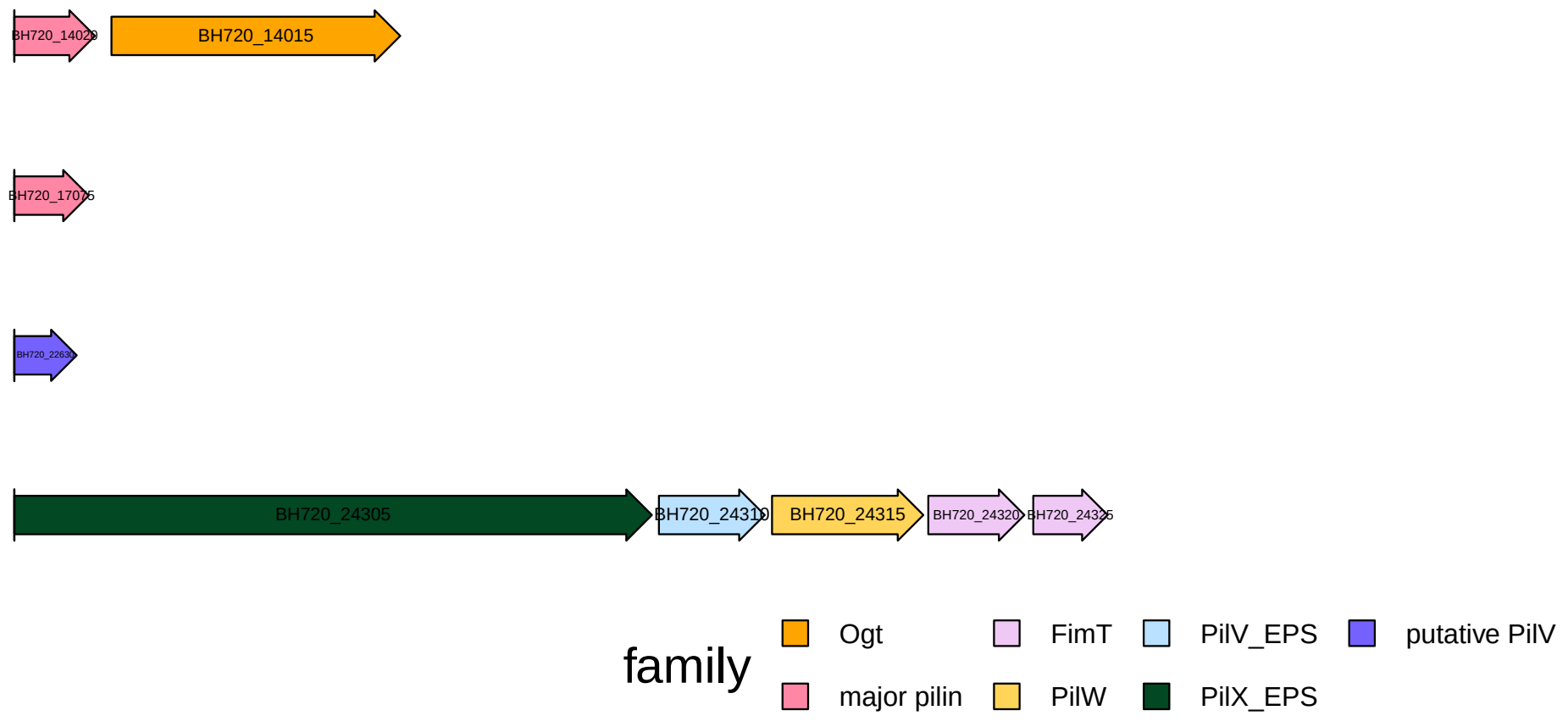

locus	gene	family	start	end
921	BH720_14015	Ogt	49952	52183
921	BH720_14020	major pilin	52310	52933
923	BH720_17075	major pilin	16736	17311
929	BH720_22630	putative PilV	8929	9411
933	BH720_24305	PilX_EPS	126751	131676
933	BH720_24310	PilV_EPS	131730	132548
933	BH720_24315	PilW	132604	133773
933	BH720_24320	FimT	133811	134554
933	BH720_24325	FimT	134622	135197

Dolichospermum flos−aquae CCAP 1403/13F

GCA_012516395.1

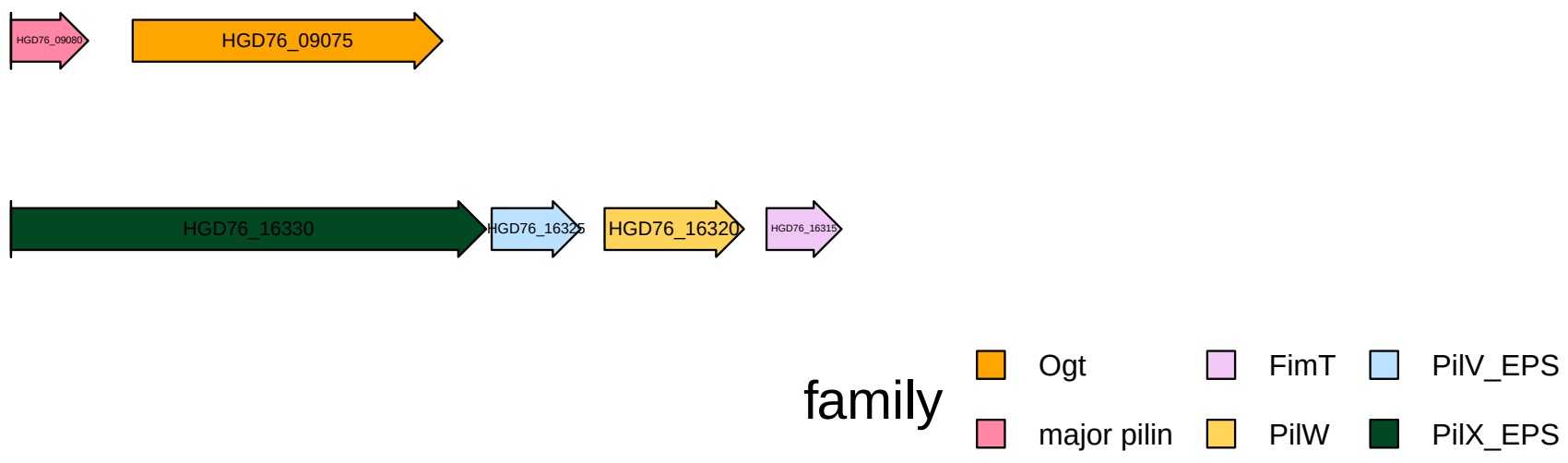

locus	gene	family	start	end
1605	HGD76_09075	Ogt	1858178	1860376
1605	HGD76_09080	major pilin	1860691	1861239
1613	HGD76_16315	FimT	3362835	3363368
1613	HGD76_16320	PilW	3363528	3364517
1613	HGD76_16325	PilV_EPS	3364686	3365318
1613	HGD76_16330	PilX_EPS	3365357	3368728

Dulcicalothrix desertica PCC 7102

GCA_003991905.1

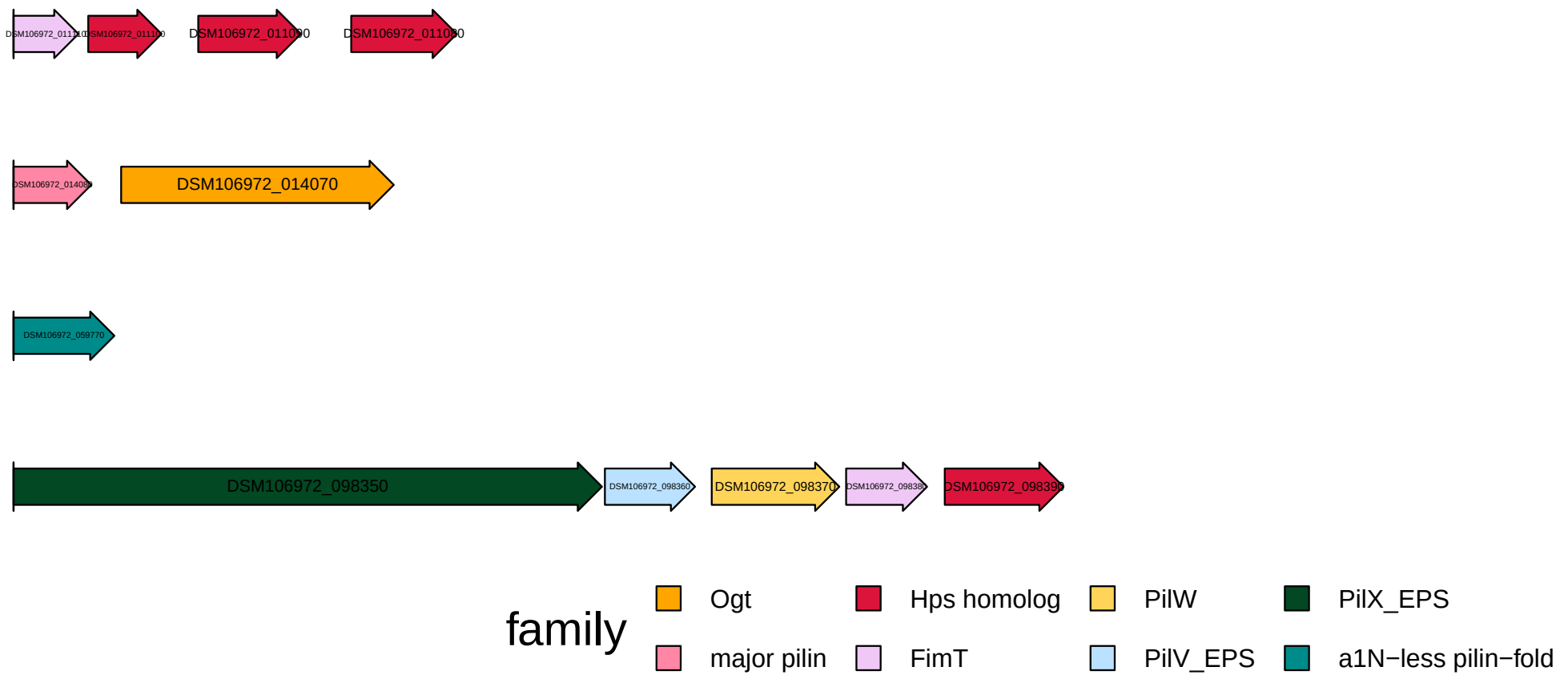

locus	gene	family	start	end
1457	DSM106972_011080	Hps homolog	331931	332794
1457	DSM106972_011090	Hps homolog	333205	334044
1457	DSM106972_011100	Hps homolog	334344	334943
1457	DSM106972_011110	FimT	335023	335553
1459	DSM106972_014070	Ogt	126379	128607
1459	DSM106972_014080	major pilin	128851	129486
1465	DSM106972_059770	a1N−less pilin−fold	163575	164399
1473	DSM106972_098350	PilX_EPS	2083	6891
1473	DSM106972_098360	PilV_EPS	6915	7652
1473	DSM106972_098370	PilW	7788	8831
1473	DSM106972_098380	FimT	8886	9548
1473	DSM106972_098390	Hps homolog	9692	10663

Euhalothece natronophila Z−M001

GCA_007904085.1

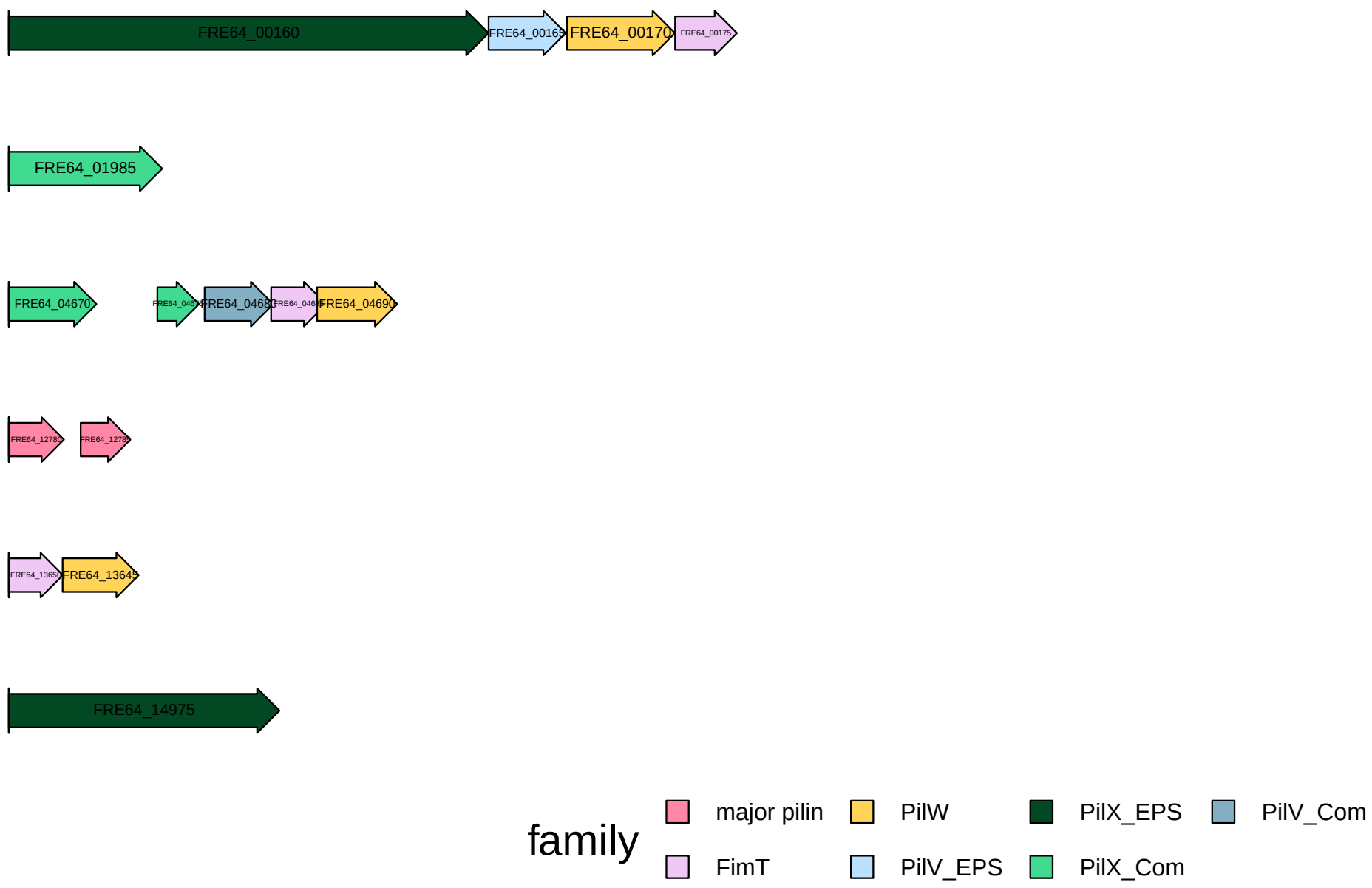

locus	gene	family	start	end
1540	FRE64_00160	PilX_EPS	37646	41893
1540	FRE64_00165	PilV_EPS	41893	42576
1540	FRE64_00170	PilW	42587	43543
1540	FRE64_00175	FimT	43544	44092
1543	FRE64_01985	PilX_Com	440514	441872
1547	FRE64_04670	PilX_Com	991326	992102
1547	FRE64_04675	PilX_Com	992641	993009
1547	FRE64_04680	PilV_Com	993059	993667
1547	FRE64_04685	FimT	993648	994136
1547	FRE64_04690	PilW	994055	994765
1555	FRE64_12780	major pilin	2601295	2601783
1555	FRE64_12785	major pilin	2601931	2602371
1557	FRE64_13645	PilW	2770836	2771510
1557	FRE64_13650	FimT	2771507	2771986
1559	FRE64_14975	PilX_EPS	3040811	3043207

Gloeobacter kilaueensis (strain ATCC BAA−2537 / CCAP 1431/1 / ULC 316 / JS1)

GCA_000484535.1

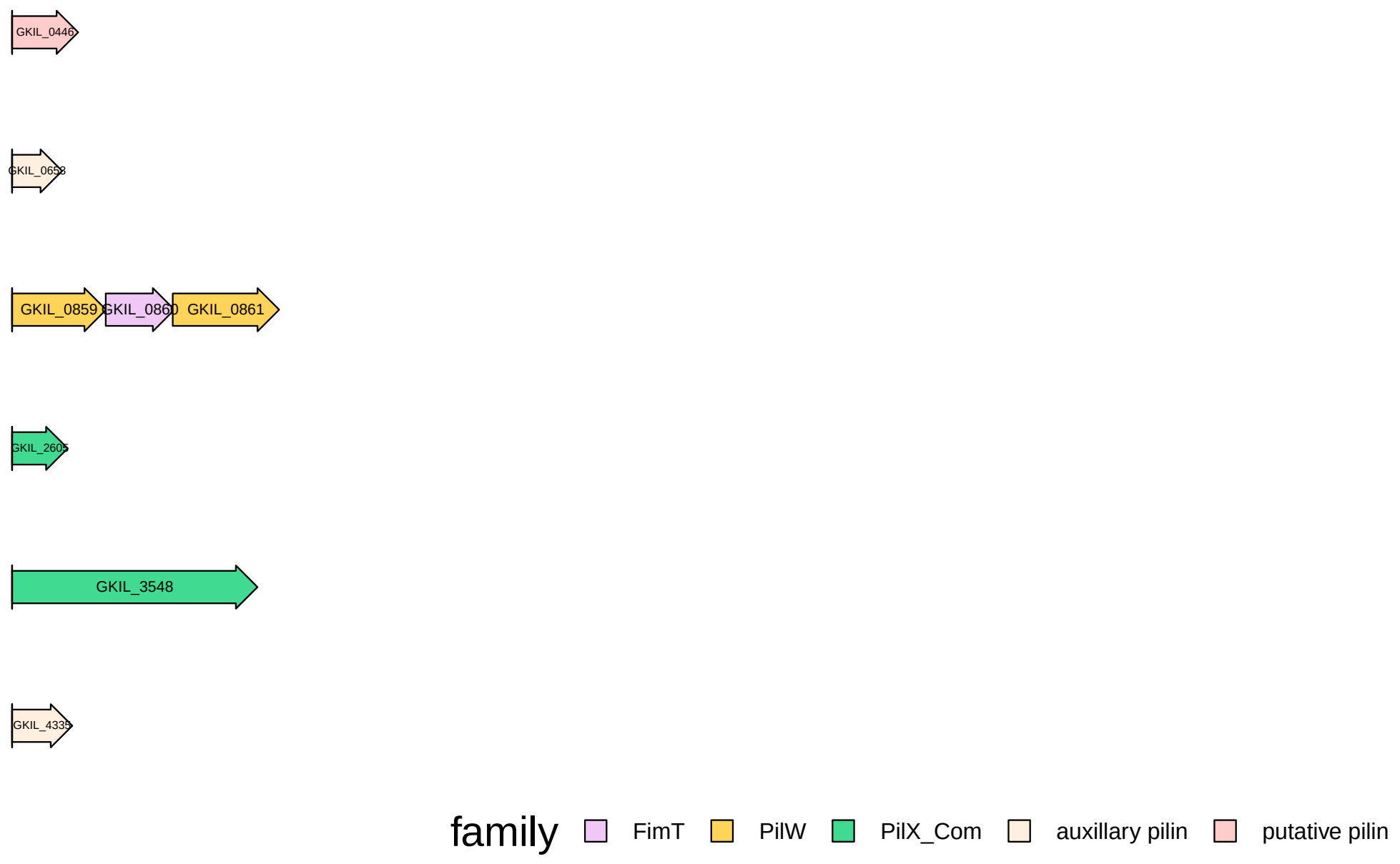

locus	gene	family	start	end
768	GKIL_0446	putative pilin	478316	478921
770	GKIL_0653	auxillary pilin	703396	703854
771	GKIL_0859	PilW	899778	900638
771	GKIL_0860	FimT	900635	901264
771	GKIL_0861	PilW	901248	902222
778	GKIL_2605	PilX_Com	2759162	2759671
781	GKIL_3548	PilX_Com	3724453	3726699
784	GKIL_4335	auxillary pilin	4554715	4555266

Gloeobacter violaceus (strain ATCC 29082 / PCC 7421)

GCA_000011385.1

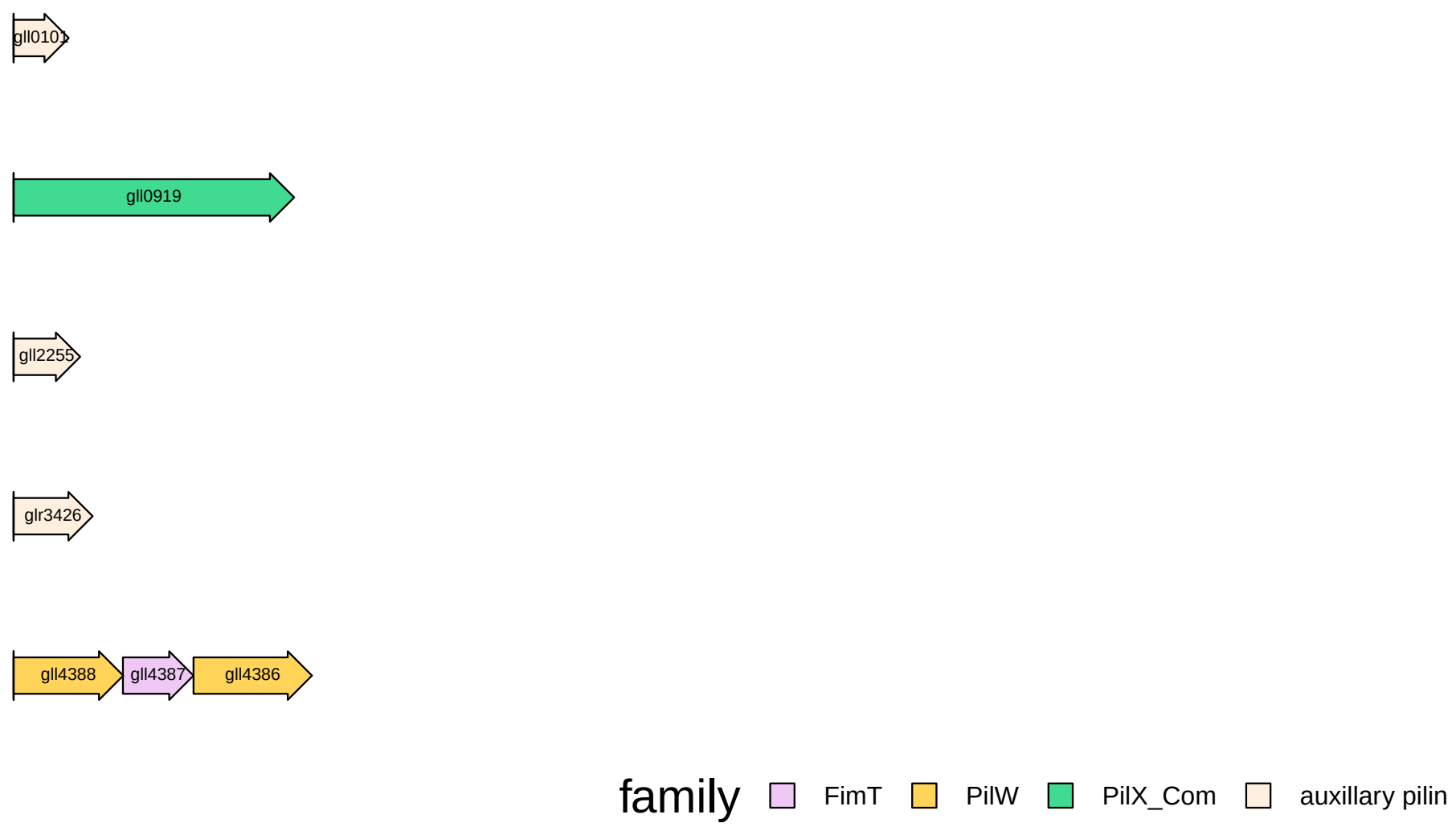

locus	gene	family	start	end
99	gll0101	auxillary pilin	101535	101984
101	gll0919	PilX_Com	973628	975913
106	gll2255	auxillary pilin	2416878	2417420
112	glr3426	auxillary pilin	3625097	3625741
115	gll4386	PilW	4615598	4616563
115	gll4387	FimT	4616563	4617138
115	gll4388	PilW	4617135	4618028

Gloeomargarita lithophora Alchichica−D10

GCA_001870225.1

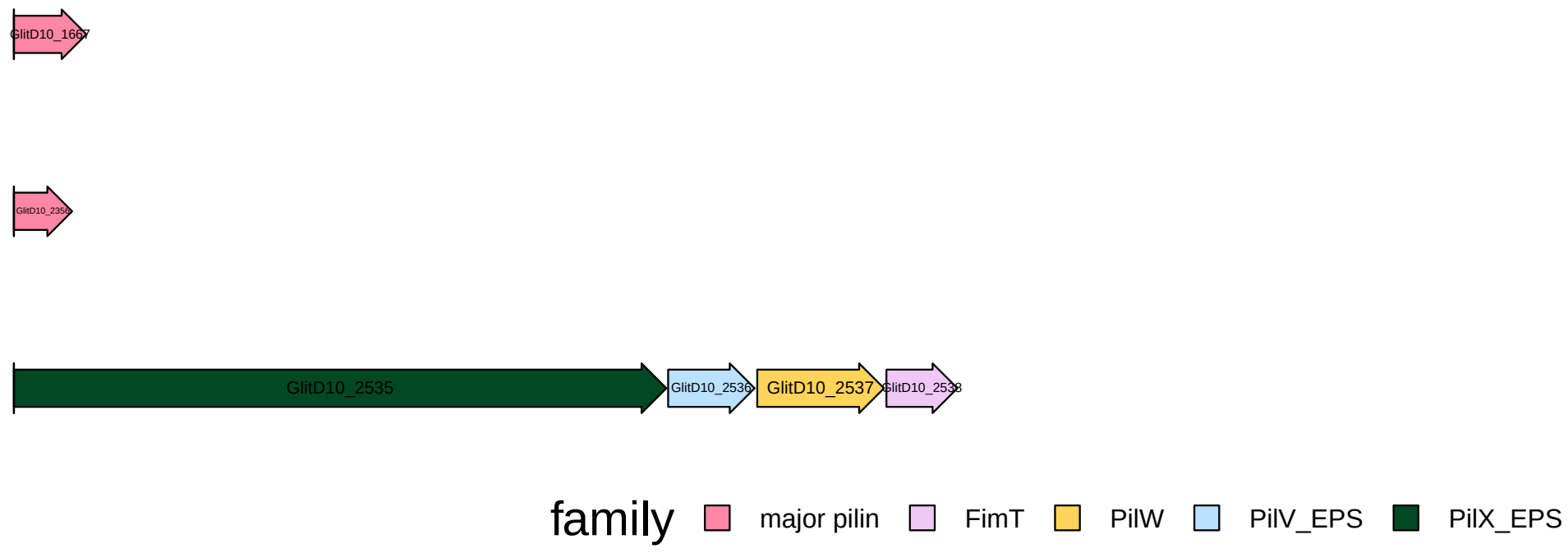

locus	gene	family	start	end
941	GlitD10_1667	major pilin	1704198	1704776
946	GlitD10_2356	major pilin	2413377	2413841
948	GlitD10_2535	PilX_EPS	2590946	2596144
948	GlitD10_2536	PilV_EPS	2596158	2596847
948	GlitD10_2537	PilW	2596868	2597878
948	GlitD10_2538	FimT	2597897	2598463

Gloeothece citriformis (strain PCC 7424) (Cyanothece sp. (strain PCC 7424))

GCA_000021825.1

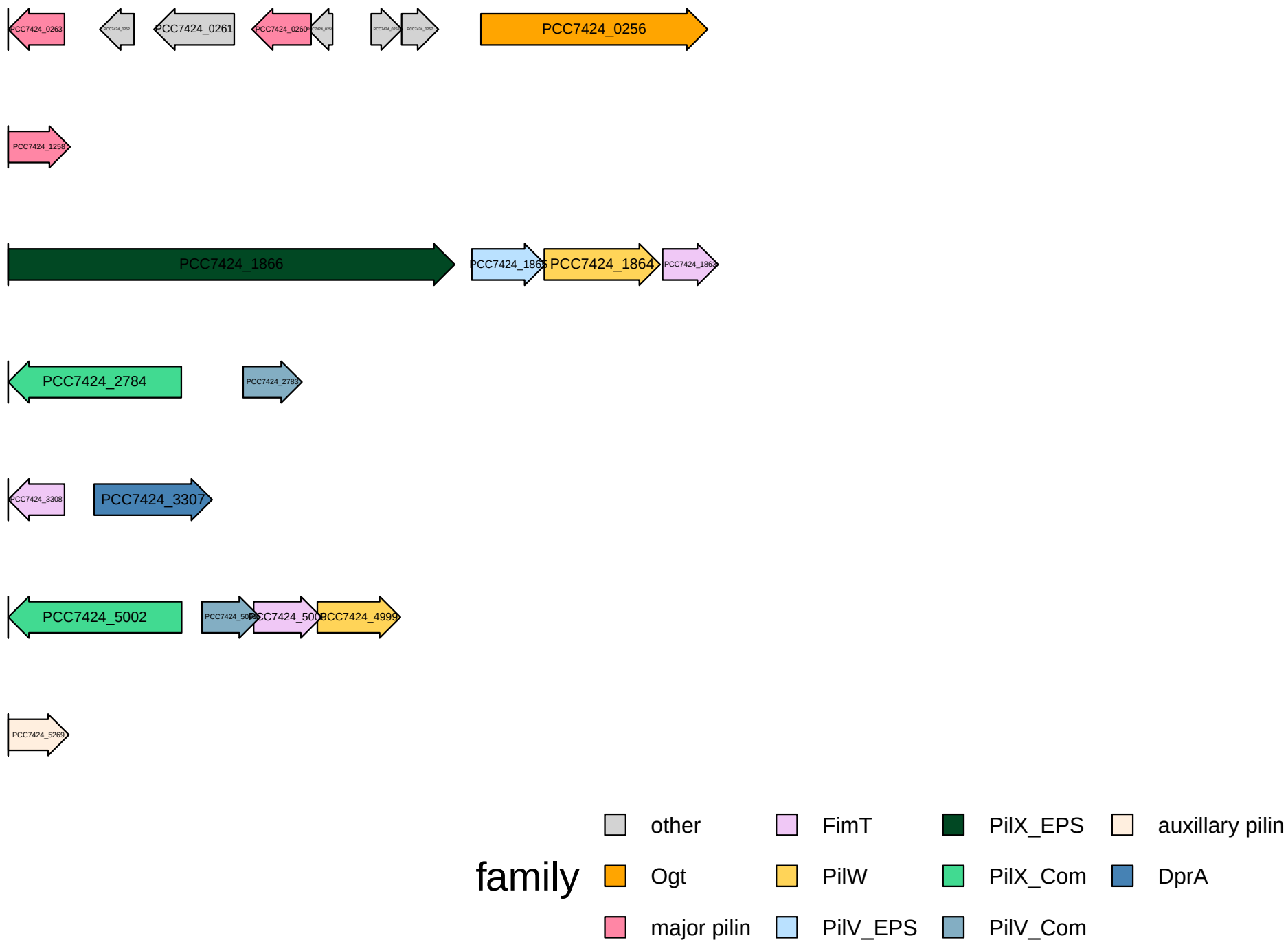

locus	gene	family	start	end
272	PCC7424_0256	Ogt	282473	284632
272	PCC7424_0257	other	285037	285387
272	PCC7424_0258	other	285384	285677
272	PCC7424_0259	other	286045	286281
272	PCC7424_0260	major pilin	286250	286813
272	PCC7424_0261	other	286982	287746
272	PCC7424_0262	other	287936	288262
272	PCC7424_0263	major pilin	288599	289135
274	PCC7424_1258	major pilin	1389780	1390370
276	PCC7424_1863	FimT	2075710	2076240
276	PCC7424_1864	PilW	2076264	2077367
276	PCC7424_1865	PilV_EPS	2077357	2078058
276	PCC7424_1866	PilX_EPS	2078221	2082474
277	PCC7424_2783	PilV_Com	3092144	3092704
277	PCC7424_2784	PilX_Com	3093293	3094942
282	PCC7424_3307	DprA	3704151	3705275
282	PCC7424_3308	FimT	3705558	3706094
287	PCC7424_4999	PilW	5558427	5559218
287	PCC7424_5000	FimT	5559178	5559825
287	PCC7424_5001	PilV_Com	5559764	5560318
287	PCC7424_5002	PilX_Com	5560511	5562163
291	PCC7424_5269	auxillary pilin	5841382	5841960

Gloeothece verrucosa (strain PCC 7822) (Cyanothece sp. (strain PCC 7822))

GCA_000147335.1

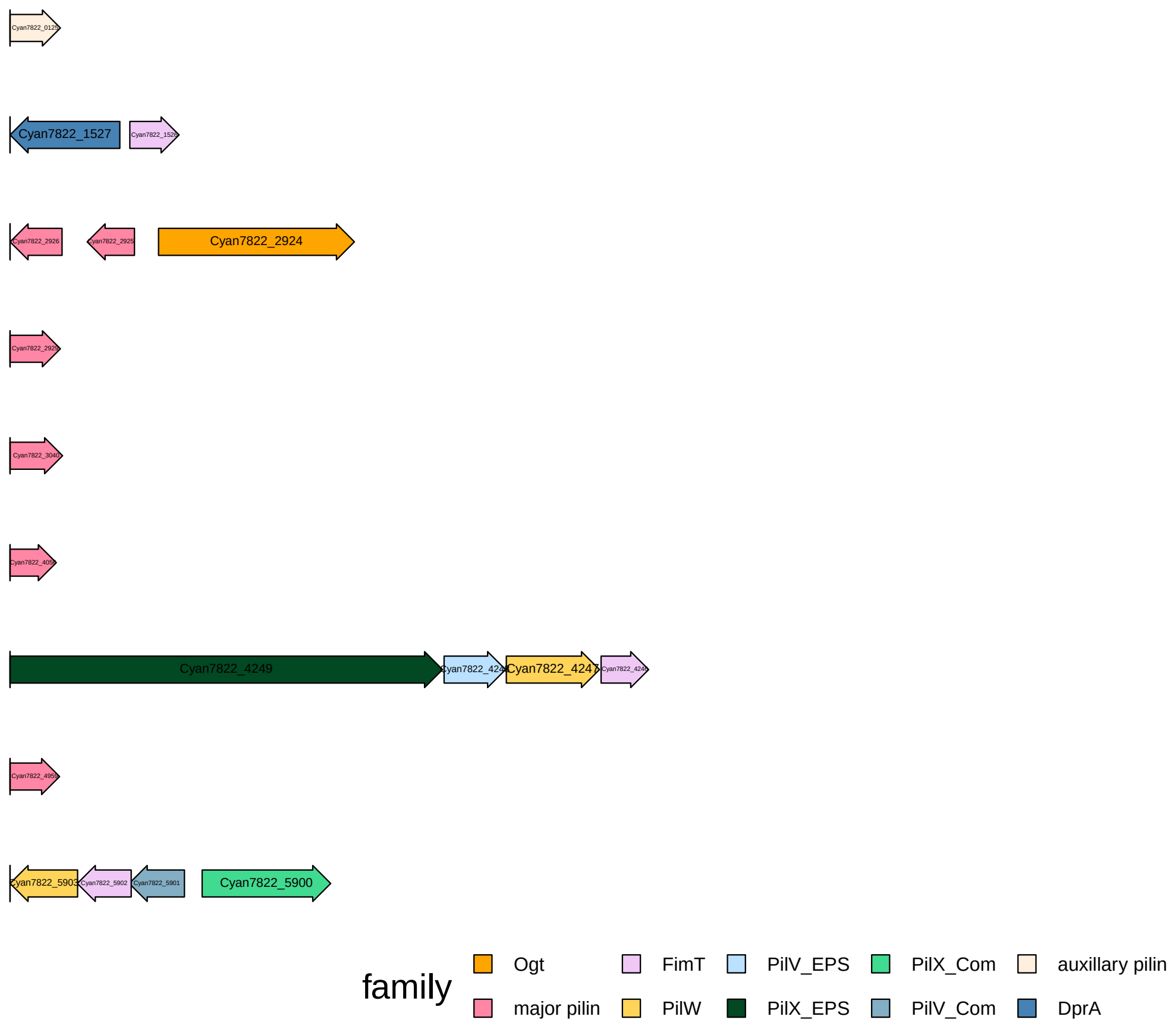

locus	gene	family	start	end
295	Cyan7822_0125	auxillary pilin	135944	136498
300	Cyan7822_1526	FimT	1664059	1664601
300	Cyan7822_1527	DprA	1664711	1665919
304	Cyan7822_2924	Ogt	3251124	3253280
304	Cyan7822_2925	major pilin	3253545	3254066
304	Cyan7822_2926	major pilin	3254342	3254914
305	Cyan7822_2929	major pilin	3258748	3259302
306	Cyan7822_3040	major pilin	3374339	3374917
308	Cyan7822_4056	major pilin	4477427	4477936
310	Cyan7822_4246	FimT	4673826	4674350
310	Cyan7822_4247	PilW	4674368	4675393
310	Cyan7822_4248	PilV_EPS	4675401	4676078
310	Cyan7822_4249	PilX_EPS	4676095	4680855
313	Cyan7822_4959	major pilin	5397990	5398535
317	Cyan7822_5900	PilX_Com	457161	458576
317	Cyan7822_5901	PilV_Com	458771	459379
317	Cyan7822_5902	FimT	459357	459950
317	Cyan7822_5903	PilW	459947	460690

Halomicronema hongdechloris C2206

GCA_002075285.3

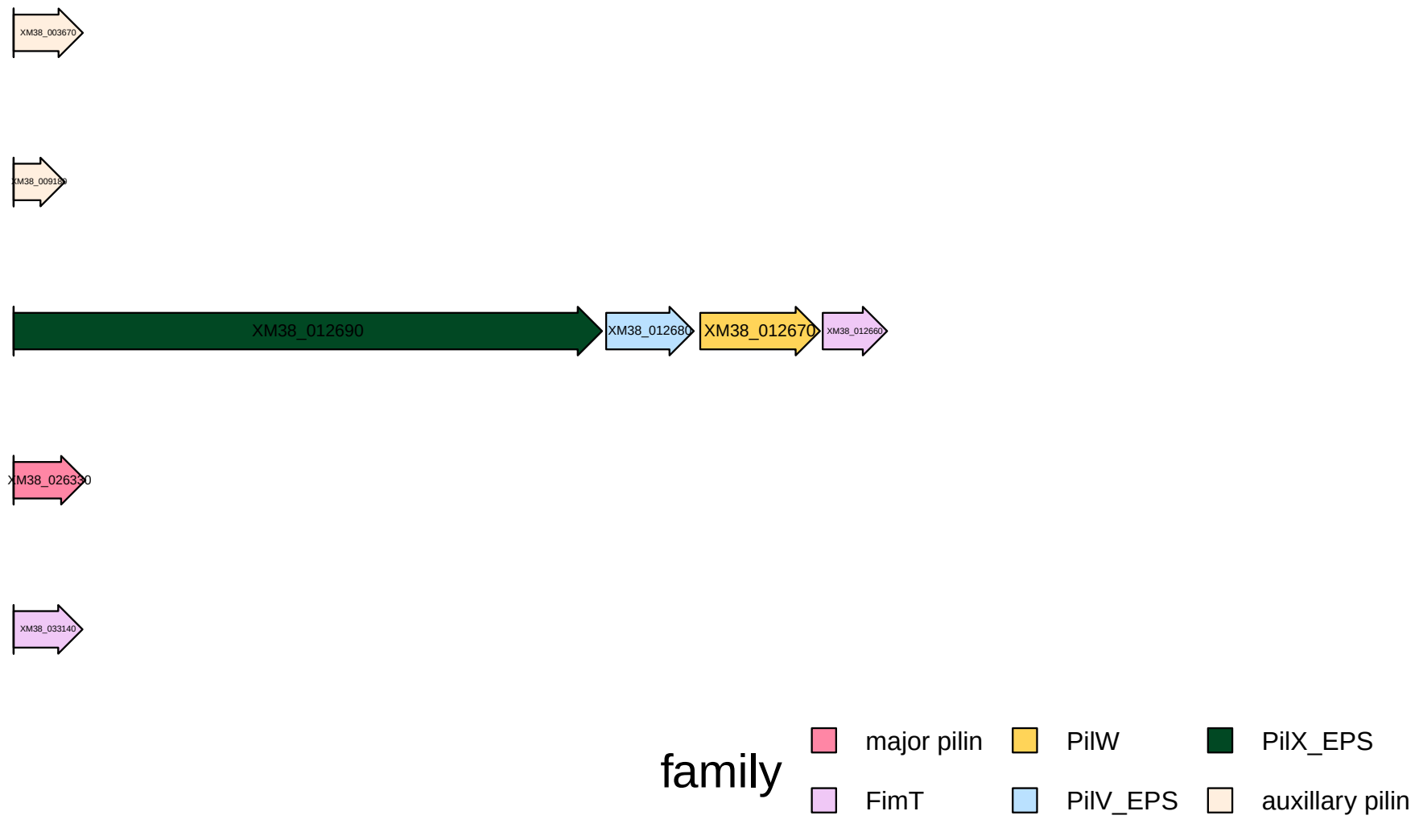

locus	gene	family	start	end
1058	XM38_003670	auxillary pilin	397895	398458
1064	XM38_009180	auxillary pilin	951584	952000
1068	XM38_012660	FimT	1303535	1304059
1068	XM38_012670	PilW	1304081	1305055
1068	XM38_012680	PilV_EPS	1305107	1305820
1068	XM38_012690	PilX_EPS	1305853	1310637
1072	XM38_026330	major pilin	2781750	2782334
1073	XM38_033140	FimT	3491367	3491927

Hassalia byssoidea VB512170

GCA_000817785.2

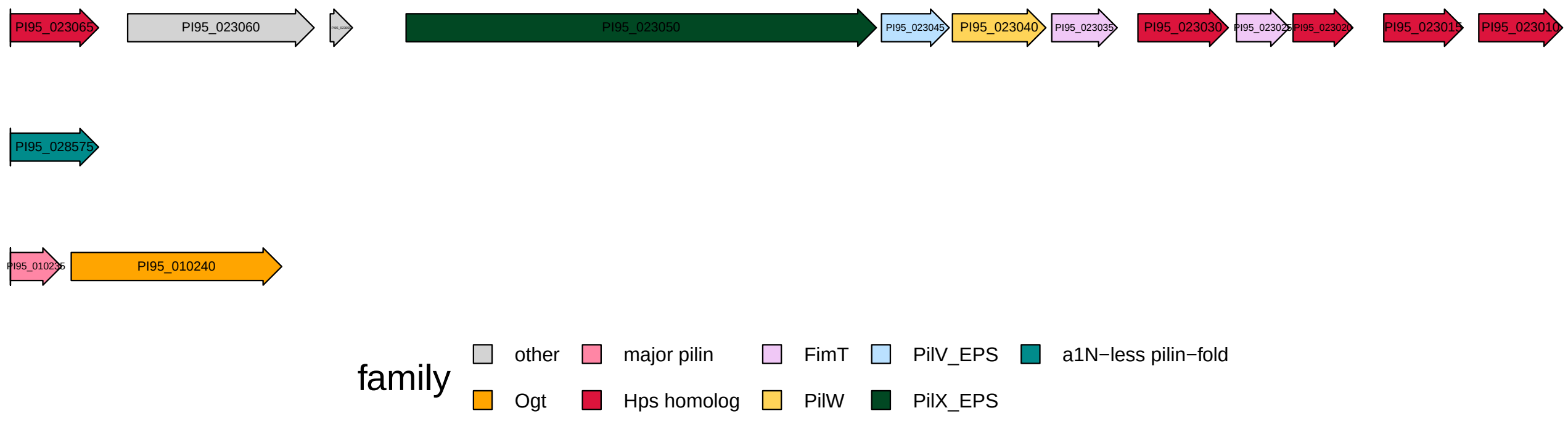

locus	gene	family	start	end
852	PI95_023010	Hps homolog	13221	14111
852	PI95_023015	Hps homolog	14277	15119
852	PI95_023020	Hps homolog	15447	16082
852	PI95_023025	FimT	16120	16683
852	PI95_023030	Hps homolog	16769	17728
852	PI95_023035	FimT	17948	18643
852	PI95_023040	PilW	18705	19697
852	PI95_023045	PilV_EPS	19731	20450
852	PI95_023050	PilX_EPS	20504	25495
852	PI95_023055	other	26064	26300
852	PI95_023060	other	26471	28450
852	PI95_023065	Hps homolog	28759	29694
855	PI95_028575	a1N−less pilin−fold	1540	2475
856	PI95_010235	major pilin	8157	8699
856	PI95_010240	Ogt	8802	11036

Hydrococcus rivularis NIES−593

GCA_001904635.1

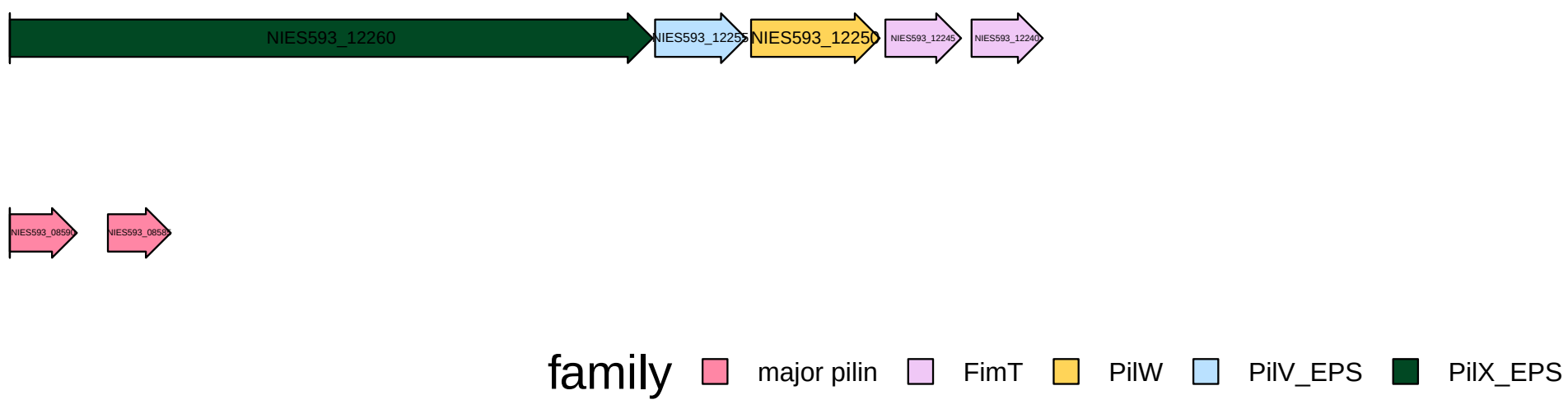

locus	gene	family	start	end
956	NIES593_12240	FimT	51564	52130
956	NIES593_12245	FimT	52213	52815
956	NIES593_12250	PilW	52860	53882
956	NIES593_12255	PilV_EPS	53923	54645
956	NIES593_12260	PilX_EPS	54664	59772
965	NIES593_08585	major pilin	162402	162902
965	NIES593_08590	major pilin	163148	163681

Hyella patelloides LEGE 07179

GCA_900659865.1

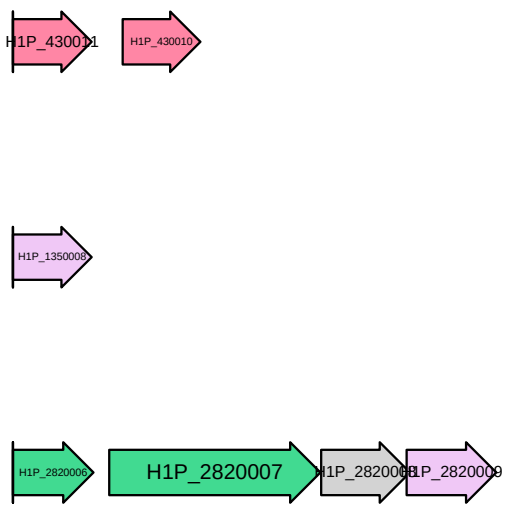

family other major pilin FimT PilX_Com

locus	gene	family	start	end
1711	H1P_430010	major pilin	15530	16039
1711	H1P_430011	major pilin	16242	16757
1719	H1P_1350008	FimT	8856	9371
1724	H1P_2820006	PilX_Com	3383	3910
1724	H1P_2820007	PilX_Com	4013	5395
1724	H1P_2820008	other	5400	5981
1724	H1P_2820009	FimT	5959	6546

Leptolyngbya iicbica LK

GCA_004212215.1

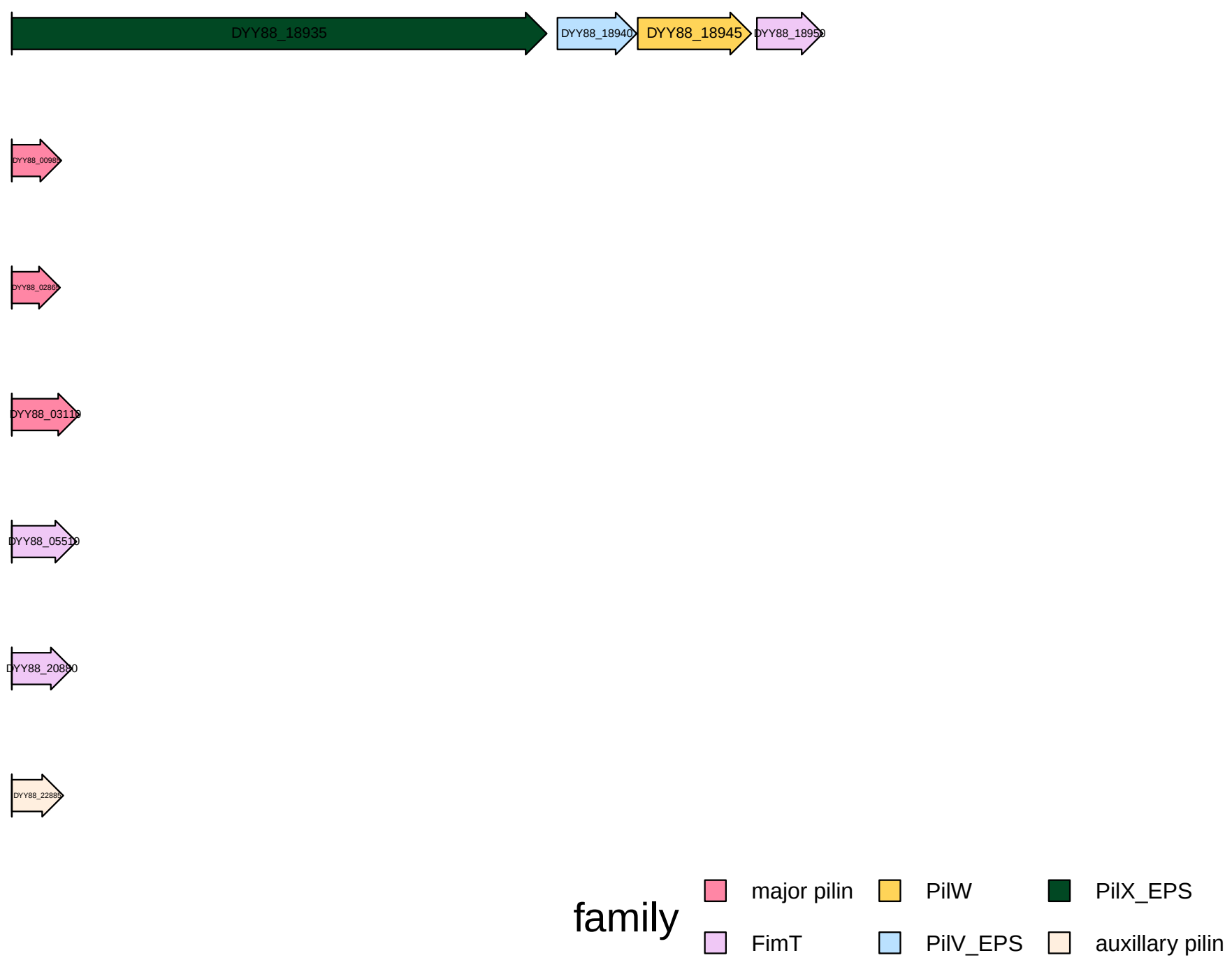

locus	gene	family	start	end
1476	DYY88_18935	PilX_EPS	38965	43974
1476	DYY88_18940	PilV_EPS	44074	44817
1476	DYY88_18945	PilW	44826	45890
1476	DYY88_18950	FimT	45942	46559
1477	DYY88_00985	major pilin	238389	238853
1479	DYY88_02865	major pilin	673363	673815
1480	DYY88_03110	major pilin	725388	726020
1485	DYY88_05510	FimT	1311229	1311834
1488	DYY88_20880	FimT	58997	59560
1496	DYY88_22885	auxillary pilin	68626	69108

Lyngbya aestuarii BL J

GCA_000478195.2

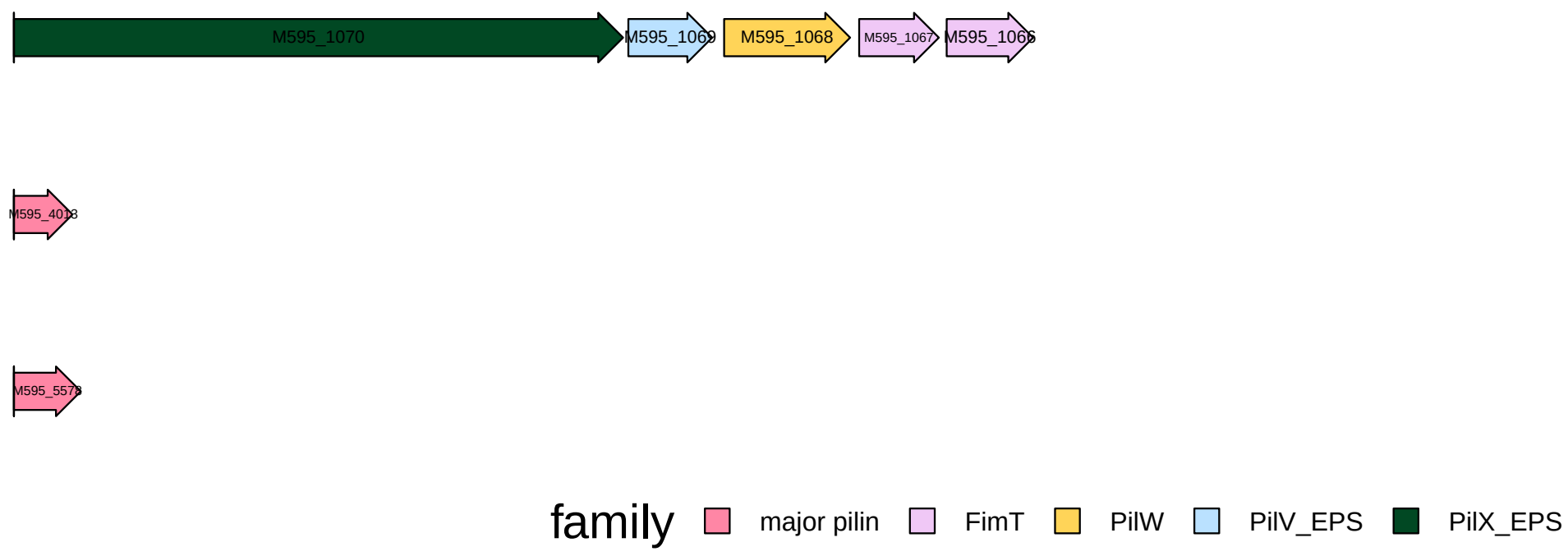

locus	gene	family	start	end
745	M595_1066	FimT	138504	139193
745	M595_1067	FimT	139255	139890
745	M595_1068	PilW	139962	140966
745	M595_1069	PilV_EPS	141061	141729
745	M595_1070	PilX_EPS	141771	146624
753	M595_4013	major pilin	5282	5749
762	M595_5578	major pilin	11836	12369

Lyngbya confervoides BDU141951

GCA_000817775.2

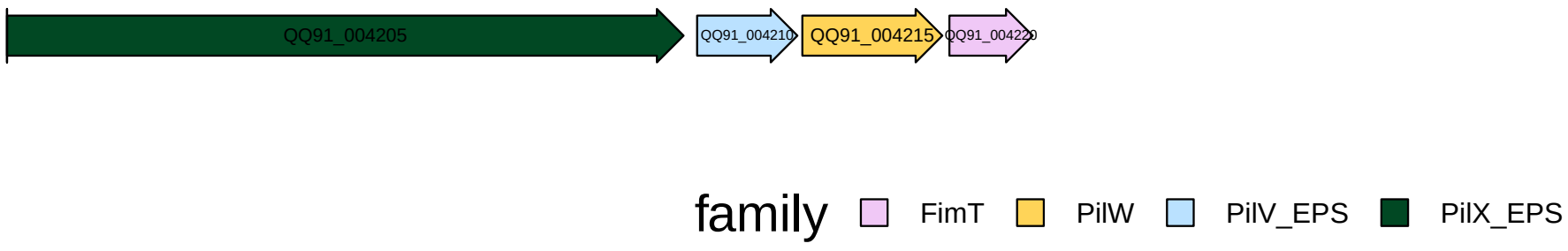

locus	gene	family	start	end
833	QQ91_004205	PilX_EPS	343017	348026
833	QQ91_004210	PilV_EPS	348126	348869
833	QQ91_004215	PilW	348905	349942
833	QQ91_004220	FimT	349994	350611

Mastigocoleus testarum BC008

GCA_001456025.1

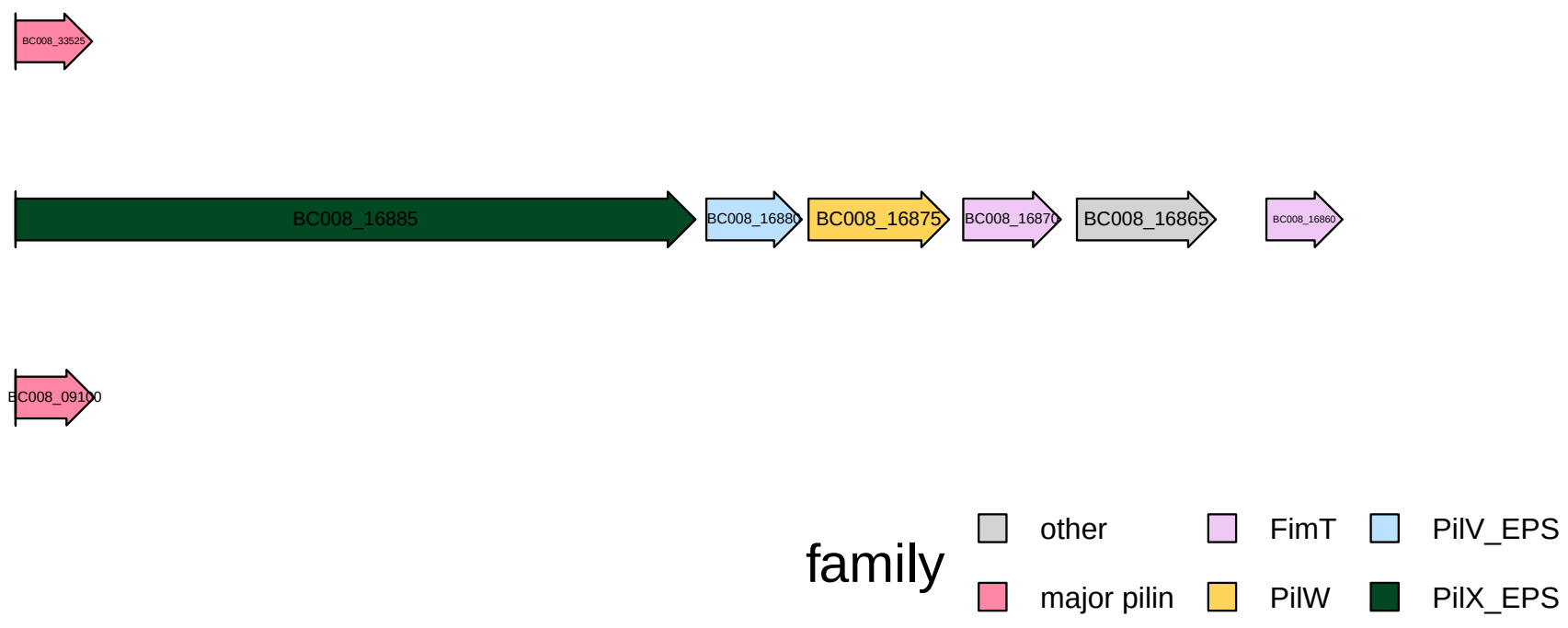

locus	gene	family	start	end
898	BC008_33525	major pilin	45398	45943
908	BC008_16860	FimT	17021	17563
908	BC008_16865	other	17922	18911
908	BC008_16870	FimT	19025	19720
908	BC008_16875	PilW	19820	20821
908	BC008_16880	PilV_EPS	20868	21548
908	BC008_16885	PilX_EPS	21625	26463
912	BC008_09100	major pilin	25043	25603

Merismopedia glauca CCAP 1448/3

GCA_003003775.1

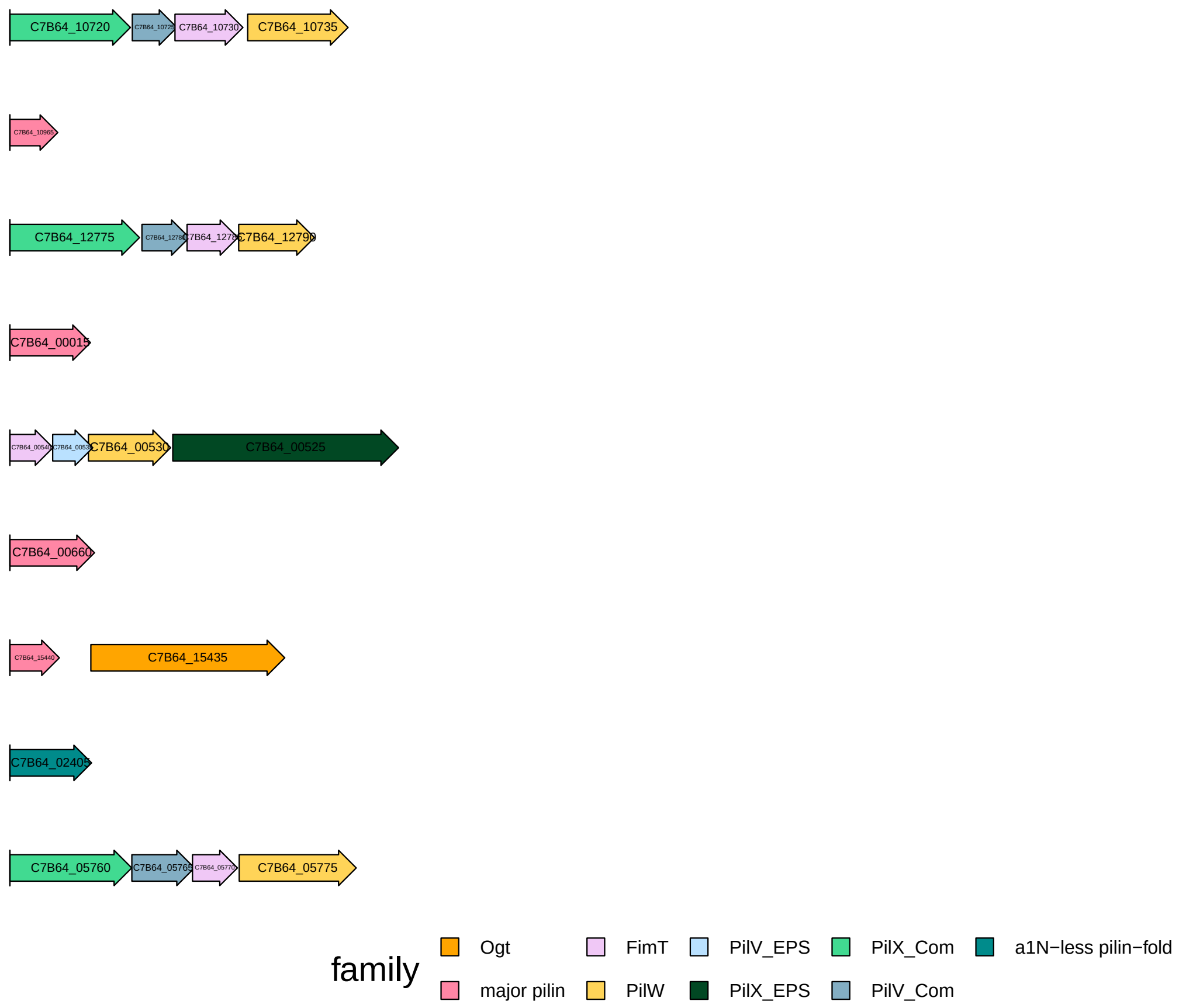

locus	gene	family	start	end
1221	C7B64_10720	PilX_Com	253	1602
1221	C7B64_10725	PilV_Com	1623	2123
1221	C7B64_10730	FimT	2101	2862
1221	C7B64_10735	PilW	2915	4039
1222	C7B64_10965	major pilin	9335	9871
1225	C7B64_12775	PilX_Com	27864	29315
1225	C7B64_12780	PilV_Com	29342	29872
1225	C7B64_12785	FimT	29847	30416
1225	C7B64_12790	PilW	30425	31273
1227	C7B64_00015	major pilin	1585	2487
1229	C7B64_00525	PilX_EPS	24356	26884
1229	C7B64_00530	PilW	26908	27828
1229	C7B64_00535	PilV_EPS	27779	28228
1229	C7B64_00540	FimT	28225	28707
1230	C7B64_00660	major pilin	54390	55337
1231	C7B64_15435	Ogt	10582	12753
1231	C7B64_15440	major pilin	13105	13659
1238	C7B64_02405	a1N−less pilin−fold	52897	53811
1243	C7B64_05760	PilX_Com	32011	33375
1243	C7B64_05765	PilV_Com	33376	34077
1243	C7B64_05770	FimT	34055	34558
1243	C7B64_05775	PilW	34578	35888

Microcystis aeruginosa (strain NIES−843 / IAM M−2473)

GCA_000010625.1

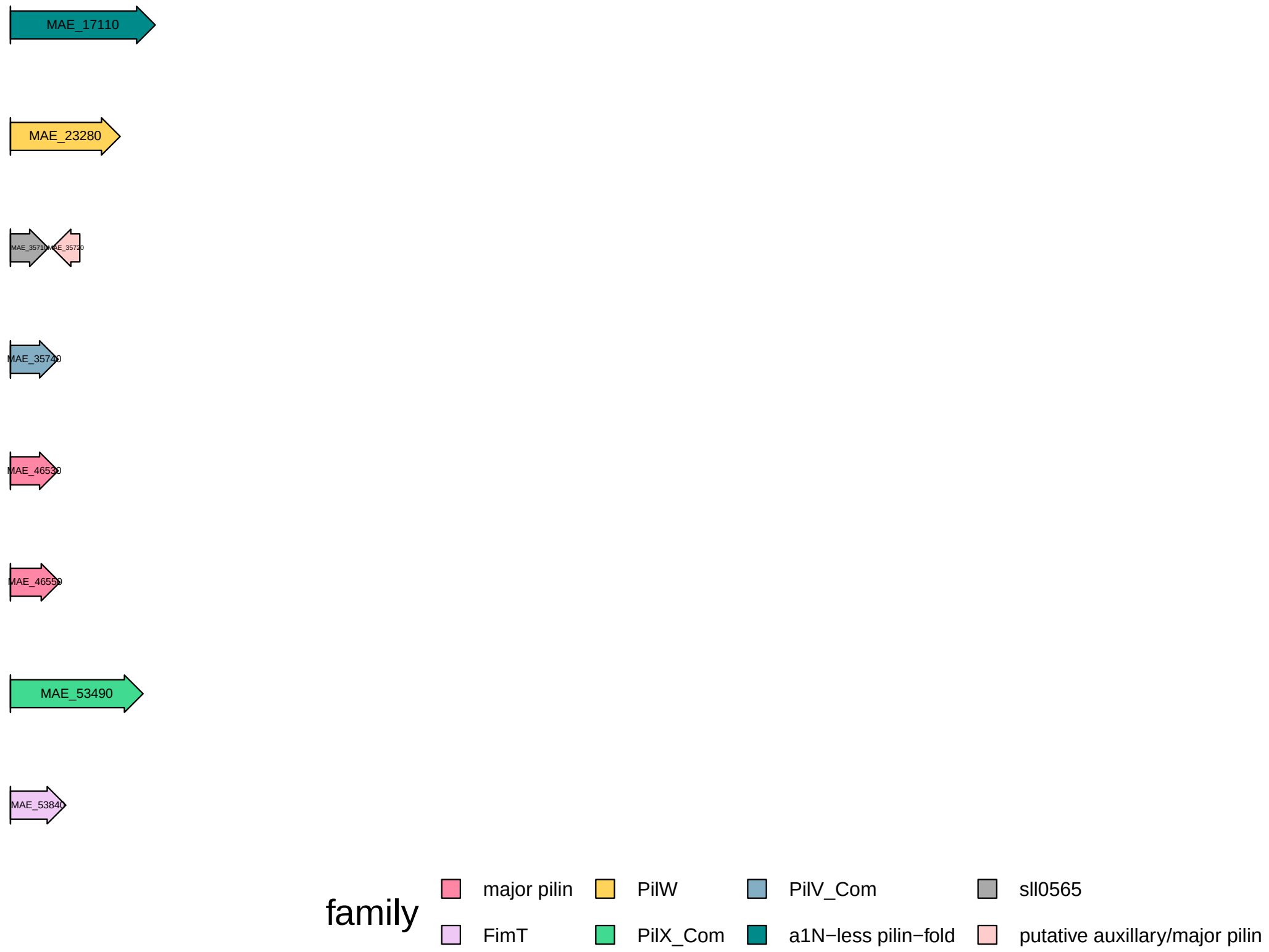

locus	gene	family	start	end
61	MAE_17110	a1N−less pilin−fold	1526900	1528435
63	MAE_23280	PilW	2092201	2093364
67	MAE_35710	sll0565	3225750	3226151
67	MAE_35720	putative auxillary/major pilin	3226192	3226485
68	MAE_35740	PilV_Com	3226799	3227305
71	MAE_46530	major pilin	4279478	4279984
72	MAE_46550	major pilin	4280370	4280894
75	MAE_53490	PilX_Com	4911408	4912814
76	MAE_53840	FimT	4946369	4946956

Moorena bouillonii PNG

GCA_001942495.1

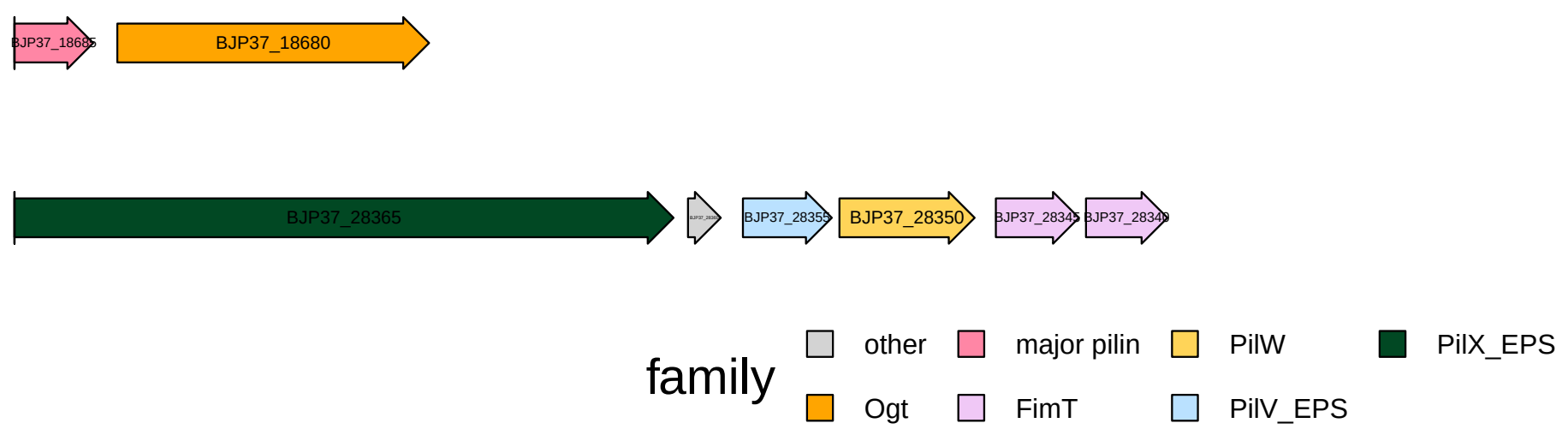

locus	gene	family	start	end
1045	BJP37_18680	Ogt	4818190	4820574
1045	BJP37_18685	major pilin	4820758	4821360
1049	BJP37_28340	FimT	7290515	7291138
1049	BJP37_28345	FimT	7291192	7291827
1049	BJP37_28350	PilW	7291989	7293023
1049	BJP37_28355	PilV_EPS	7293081	7293761
1049	BJP37_28360	other	7293930	7294181
1049	BJP37_28365	PilX_EPS	7294291	7299330

Moorena producens 3L

GCA_000211815.1

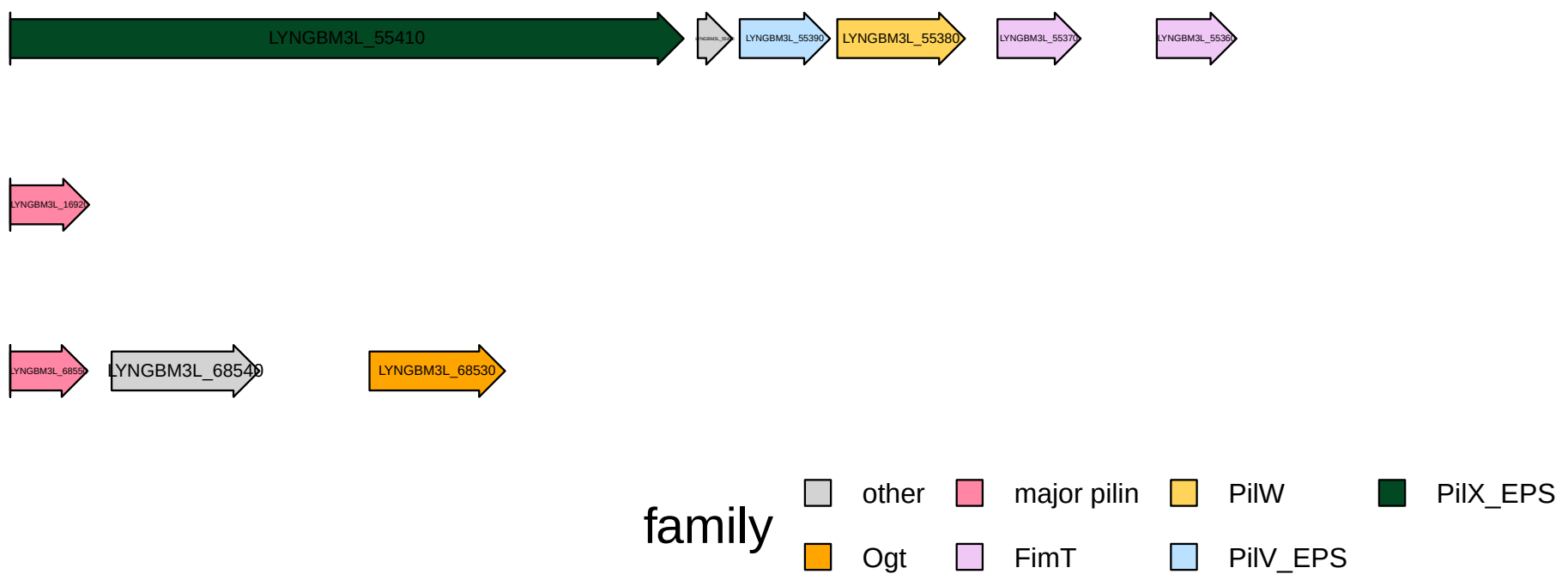

locus	gene	family	start	end
393	LYNGBM3L_55360	FimT	45939	46547
393	LYNGBM3L_55370	FimT	47127	47762
393	LYNGBM3L_55380	PilW	48009	48983
393	LYNGBM3L_55390	PilV_EPS	49038	49727
393	LYNGBM3L_55400	other	49784	50047
393	LYNGBM3L_55410	PilX_EPS	50154	55289
397	LYNGBM3L_16920	major pilin	40597	41199
407	LYNGBM3L_68530	Ogt	214520	215554
407	LYNGBM3L_68540	other	216393	217520
407	LYNGBM3L_68550	major pilin	217703	218293

Neosynechococcus sphagnicola sy1

GCA_000775285.1

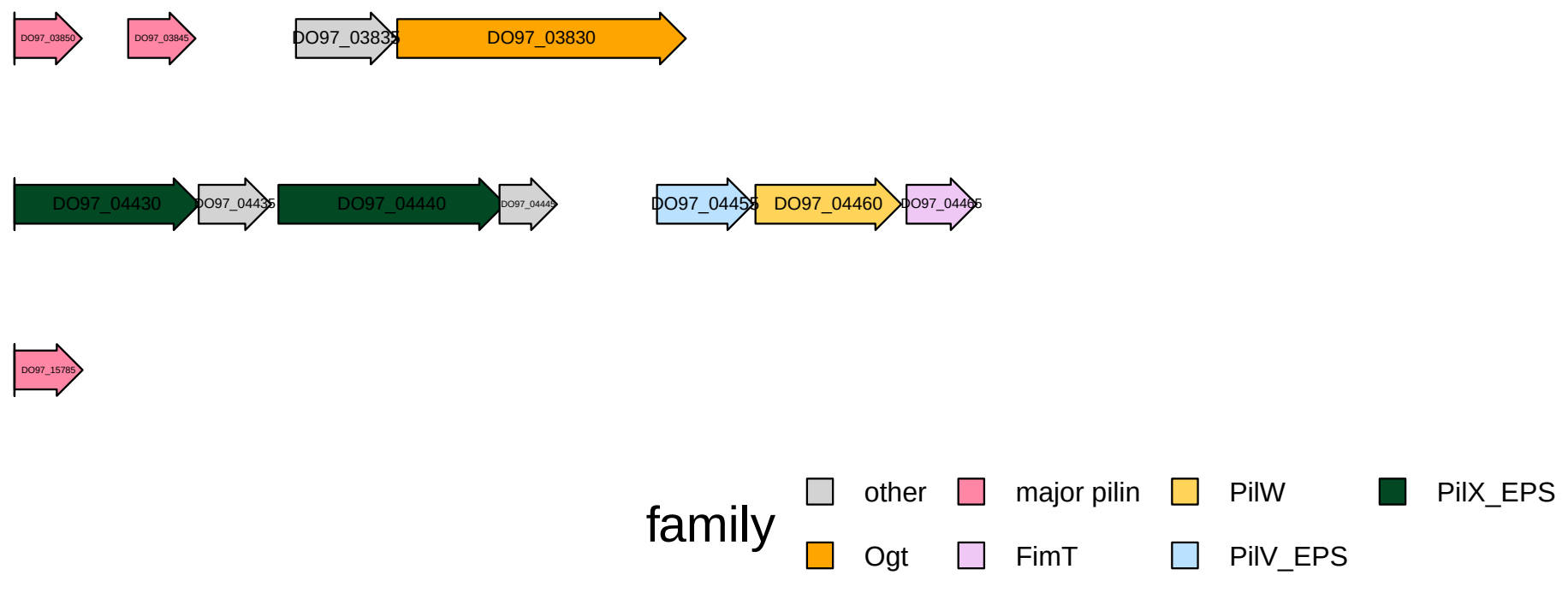

locus	gene	family	start	end
818	DO97_03830	Ogt	71235	73442
818	DO97_03835	other	73446	74216
818	DO97_03845	major pilin	74984	75499
818	DO97_03850	major pilin	75853	76368
819	DO97_04430	PilX_EPS	201010	202425
819	DO97_04435	other	202418	202972
819	DO97_04440	PilX_EPS	203028	204761
819	DO97_04445	other	204718	205158
819	DO97_04455	PilV_EPS	205922	206659
819	DO97_04460	PilW	206675	207790
819	DO97_04465	FimT	207830	208366
825	DO97_15785	major pilin	15511	16032

Nodularia spumigena UHCC 0039

GCA_003054475.1

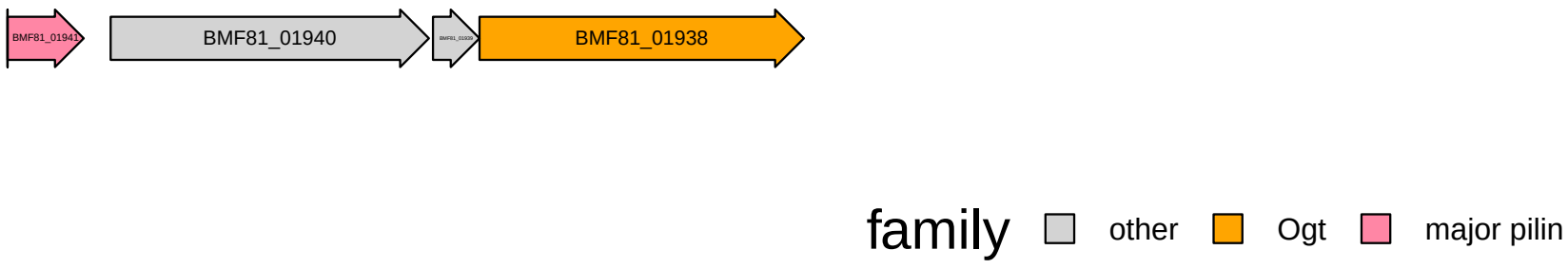

locus	gene	family	start	end
1336	BMF81_01938	Ogt	2218546	2220777
1336	BMF81_01939	other	2220777	2221097
1336	BMF81_01940	other	2221125	2223314
1336	BMF81_01941	major pilin	2223499	2224023

Nostoc azollae (strain 0708) (Anabaena azollae (strain 0708))

GCA_000196515.1

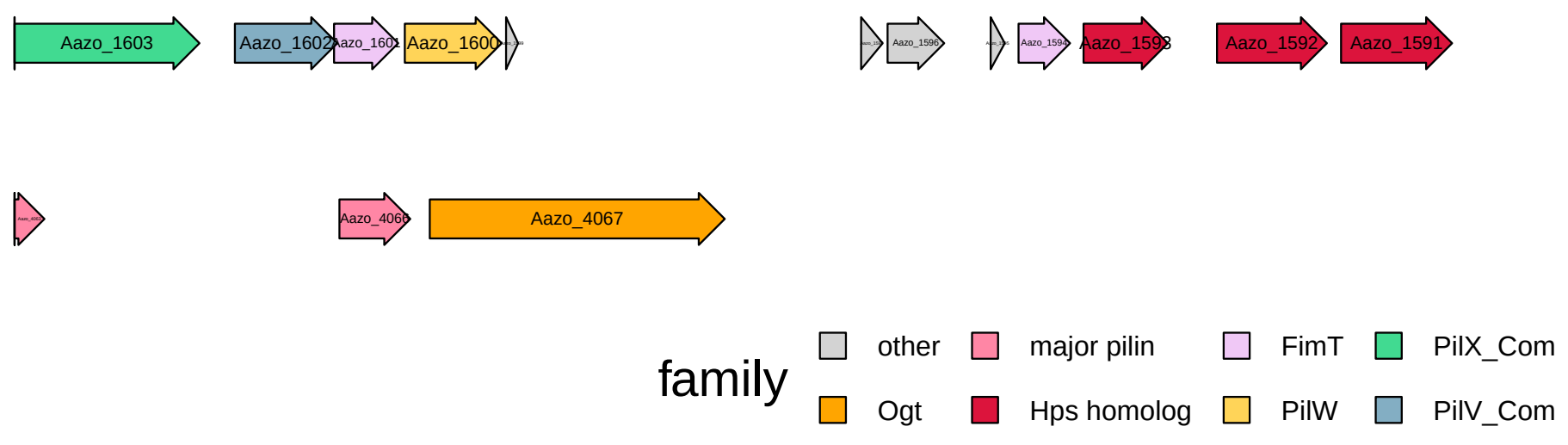

locus	gene	family	start	end
380	Aazo_1591	Hps homolog	1666613	1667458
380	Aazo_1592	Hps homolog	1667565	1668401
380	Aazo_1593	Hps homolog	1668775	1669416
380	Aazo_1594	FimT	1669518	1669910
380	Aazo_1595	other	1670015	1670122
380	Aazo_1596	other	1670475	1670906
380	Aazo_1597	other	1670943	1671107
380	Aazo_1599	other	1673715	1673807
380	Aazo_1600	PilW	1673840	1674577
380	Aazo_1601	FimT	1674627	1675115
380	Aazo_1602	PilV_Com	1675090	1675869
380	Aazo_1603	PilX_Com	1676138	1677544
384	Aazo_4063	major pilin	4124043	4124270
384	Aazo_4066	major pilin	4126513	4127052
384	Aazo_4067	Ogt	4127200	4129443

Nostoc commune NIES−4072

GCA_003113895.1

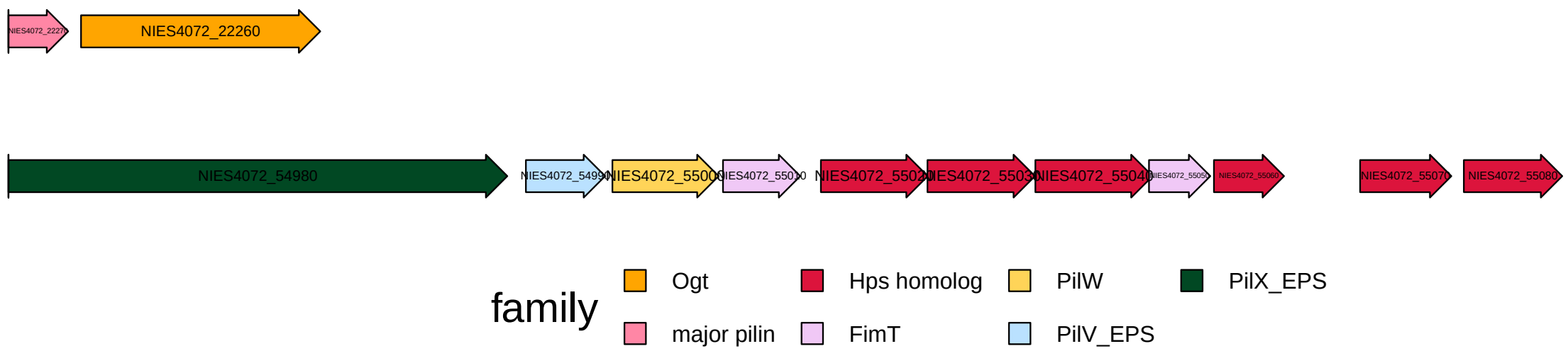

locus	gene	family	start	end
1346	NIES4072_22260	Ogt	2541291	2543498
1346	NIES4072_22270	major pilin	2543616	2544167
1353	NIES4072_54980	PilX_EPS	6223809	6228410
1353	NIES4072_54990	PilV_EPS	6228581	6229312
1353	NIES4072_55000	PilW	6229379	6230359
1353	NIES4072_55010	FimT	6230399	6231112
1353	NIES4072_55020	Hps homolog	6231302	6232288
1353	NIES4072_55030	Hps homolog	6232285	6233277
1353	NIES4072_55040	Hps homolog	6233280	6234362
1353	NIES4072_55050	FimT	6234328	6234894
1353	NIES4072_55060	Hps homolog	6234927	6235574
1353	NIES4072_55070	Hps homolog	6236275	6237117
1353	NIES4072_55080	Hps homolog	6237232	6238140

Nostoc cycadae WK−1

GCA_002897135.1

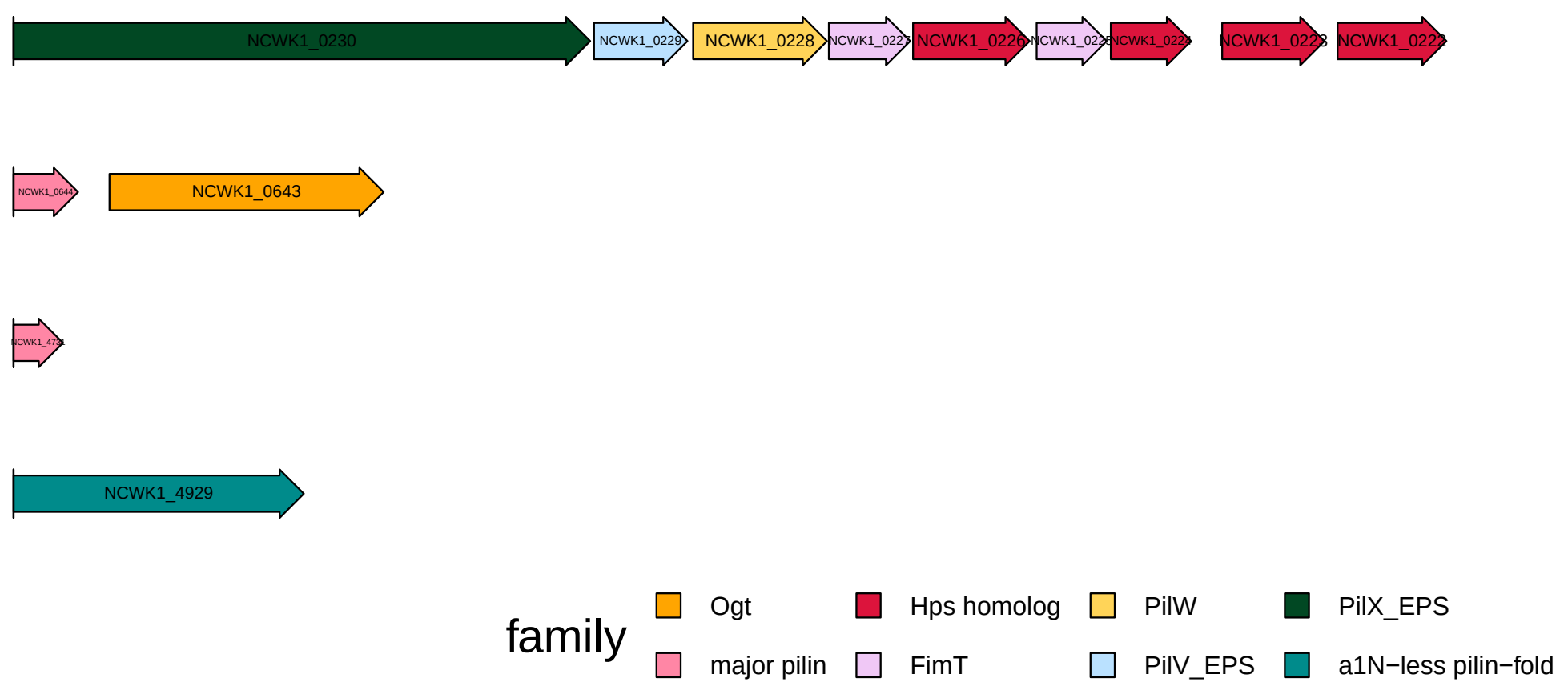

locus	gene	family	start	end
1166	NCWK1_0222	Hps homolog	288829	289719
1166	NCWK1_0223	Hps homolog	289826	290662
1166	NCWK1_0224	Hps homolog	290916	291572
1166	NCWK1_0225	FimT	291606	292178
1166	NCWK1_0226	Hps homolog	292235	293188
1166	NCWK1_0227	FimT	293211	293876
1166	NCWK1_0228	PilW	293891	294985
1166	NCWK1_0229	PilV_EPS	295031	295795
1166	NCWK1_0230	PilX_EPS	295826	300538
1170	NCWK1_0643	Ogt	271298	273538
1170	NCWK1_0644	major pilin	273795	274322
1180	NCWK1_4731	major pilin	46274	46678
1181	NCWK1_4929	a1N−less pilin−fold	93724	96096

Nostoc edaphicum CCNP1411

GCA_014023275.1

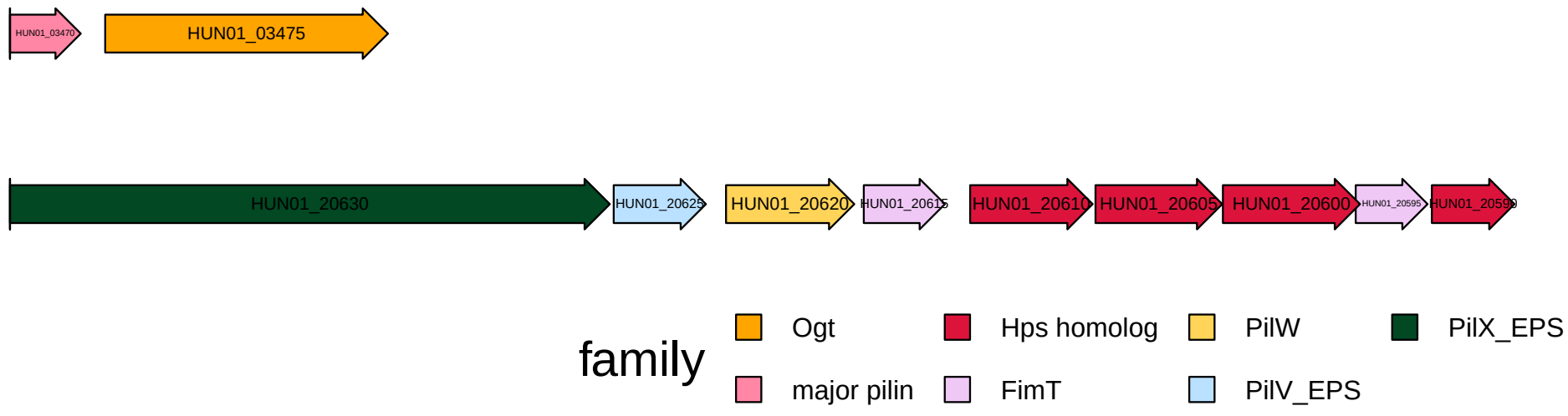

locus	gene	family	start	end
1638	HUN01_03470	major pilin	155626	156180
1638	HUN01_03475	Ogt	156371	158584
1644	HUN01_20590	Hps homolog	4307891	4308538
1644	HUN01_20595	FimT	4308571	4309134
1644	HUN01_20600	Hps homolog	4309100	4310173
1644	HUN01_20605	Hps homolog	4310178	4311170
1644	HUN01_20610	Hps homolog	4311192	4312151
1644	HUN01_20615	FimT	4312348	4312983
1644	HUN01_20620	PilW	4313057	4314061
1644	HUN01_20625	PilV_EPS	4314218	4314940
1644	HUN01_20630	PilX_EPS	4314970	4319664

Nostoc flagelliforme CCNUN1

GCA_002813575.1

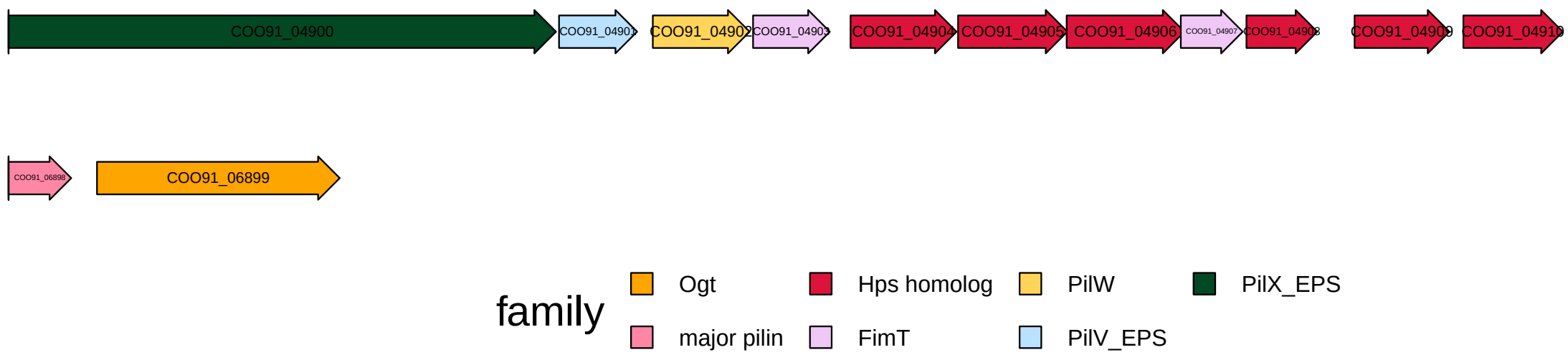

locus	gene	family	start	end
1152	COO91_04900	PilX_EPS	4537734	4542731
1152	COO91_04901	PilV_EPS	4542758	4543471
1152	COO91_04902	PilW	4543614	4544498
1152	COO91_04903	FimT	4544529	4545230
1152	COO91_04904	Hps homolog	4545419	4546387
1152	COO91_04905	Hps homolog	4546400	4547398
1152	COO91_04906	Hps homolog	4547391	4548467
1152	COO91_04907	FimT	4548433	4548999
1152	COO91_04908	Hps homolog	4549032	4549679
1152	COO91_04909	Hps homolog	4550019	4550885
1152	COO91_04910	Hps homolog	4551012	4551920
1161	COO91_06898	major pilin	6415596	6416168
1161	COO91_06899	Ogt	6416403	6418619

Nostoc piscinale CENA21

GCA_001298445.1

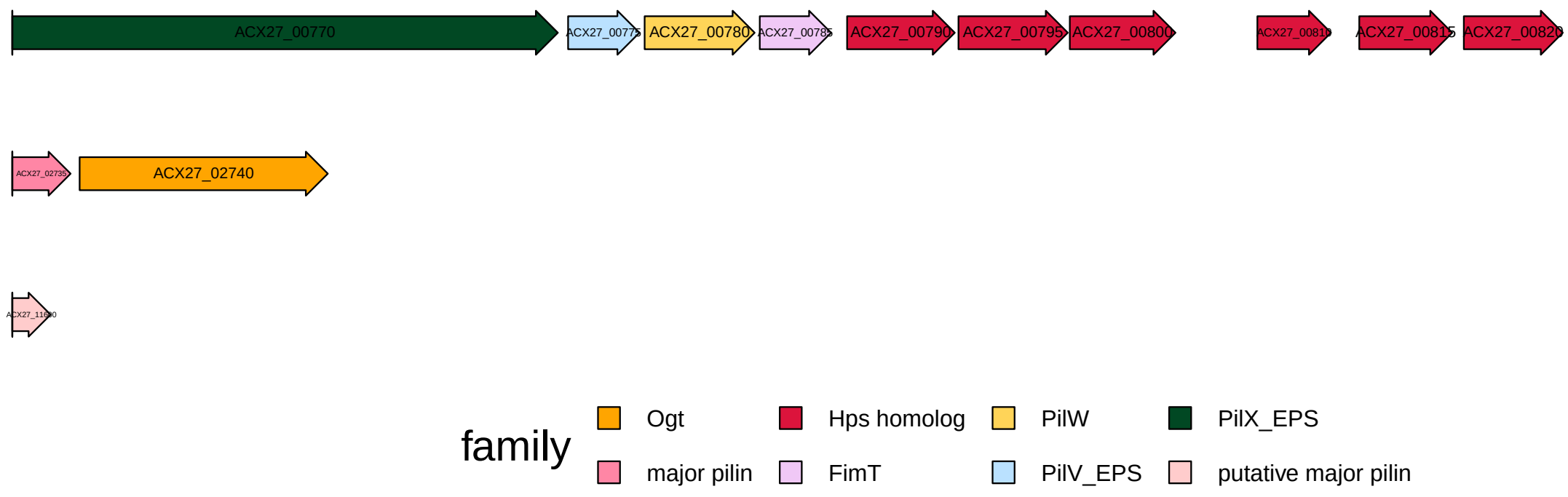

locus	gene	family	start	end
882	ACX27_00770	PilX_EPS	171159	176063
882	ACX27_00775	PilV_EPS	176155	176796
882	ACX27_00780	PilW	176843	177832
882	ACX27_00785	FimT	177878	178519
882	ACX27_00790	Hps homolog	178663	179631
882	ACX27_00795	Hps homolog	179664	180647
882	ACX27_00800	Hps homolog	180665	181615
882	ACX27_00810	Hps homolog	182351	183013
882	ACX27_00815	Hps homolog	183267	184103
882	ACX27_00820	Hps homolog	184207	185097
885	ACX27_02735	major pilin	618757	619281
885	ACX27_02740	Ogt	619362	621593
887	ACX27_11600	putative major pilin	2650645	2650989

Nostoc punctiforme (strain ATCC 29133 / PCC 73102)

GCA_000020025.1

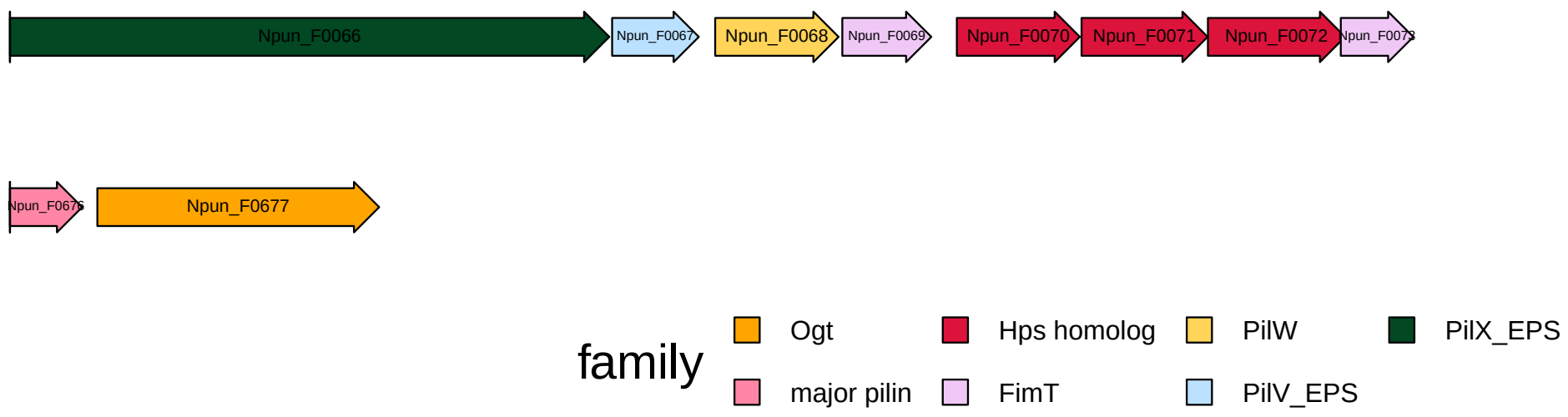

locus	gene	family	start	end
233	Npun_F0066	PilX_EPS	103560	108260
233	Npun_F0067	PilV_EPS	108282	108962
233	Npun_F0068	PilW	109090	110058
233	Npun_F0069	FimT	110087	110785
233	Npun_F0070	Hps homolog	110985	111950
233	Npun_F0071	Hps homolog	111963	112952
233	Npun_F0072	Hps homolog	112954	114030
233	Npun_F0073	FimT	113996	114565
237	Npun_F0676	major pilin	799692	800258
237	Npun_F0677	Ogt	800377	802587

Nostoc sp. (strain PCC 7120 / SAG 25.82 / UTEX 2576)

GCA_000009705.1

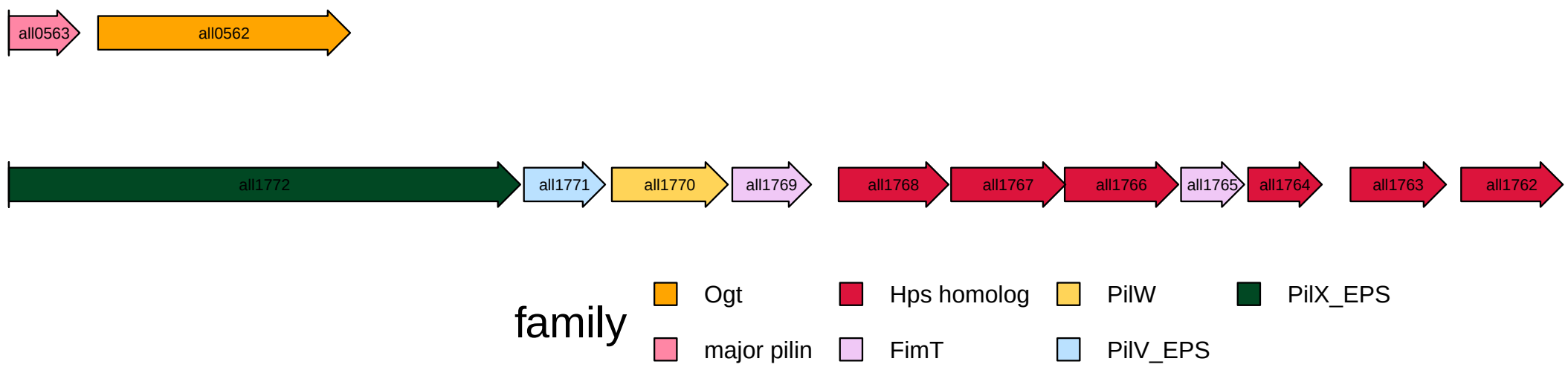

locus	gene	family	start	end
1	all0562	Ogt	654843	657062
1	all0563	major pilin	657224	657847
5	all1762	Hps homolog	2117622	2118518
5	all1763	Hps homolog	2118650	2119492
5	all1764	Hps homolog	2119743	2120393
5	all1765	FimT	2120426	2120983
5	all1766	Hps homolog	2121006	2122007
5	all1767	Hps homolog	2122000	2123007
5	all1768	Hps homolog	2123029	2123997
5	all1769	FimT	2124238	2124933
5	all1770	PilW	2124971	2125993
5	all1771	PilV_EPS	2126051	2126767
5	all1772	PilX_EPS	2126798	2131300

Okeania hirsuta

GCA_003838195.1

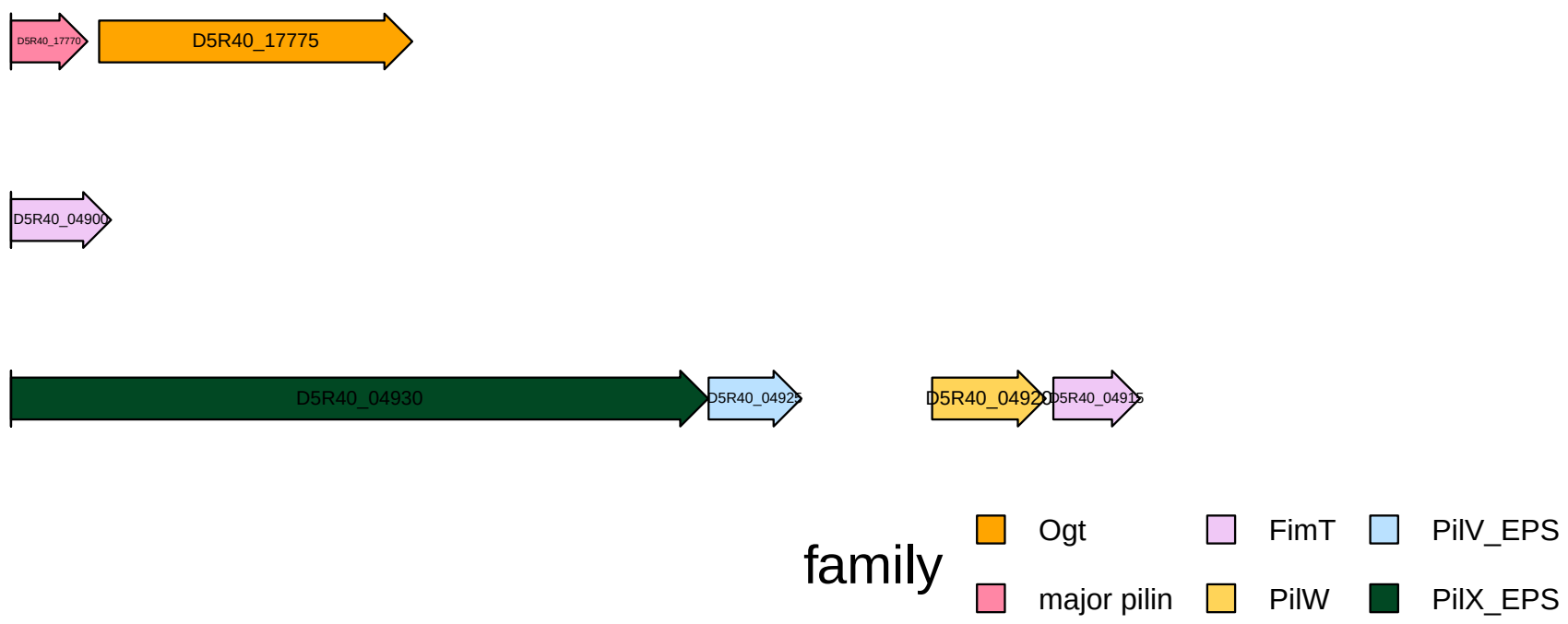

locus	gene	family	start	end
1402	D5R40_17770	major pilin	13603	14145
1402	D5R40_17775	Ogt	14228	16450
1406	D5R40_04900	FimT	19275	19985
1407	D5R40_04915	FimT	22166	22780
1407	D5R40_04920	PilW	22834	23640
1407	D5R40_04925	PilV_EPS	24567	25226
1407	D5R40_04930	PilX_EPS	25229	30175

Oscillatoria acuminata PCC 6304

GCA_000317105.1

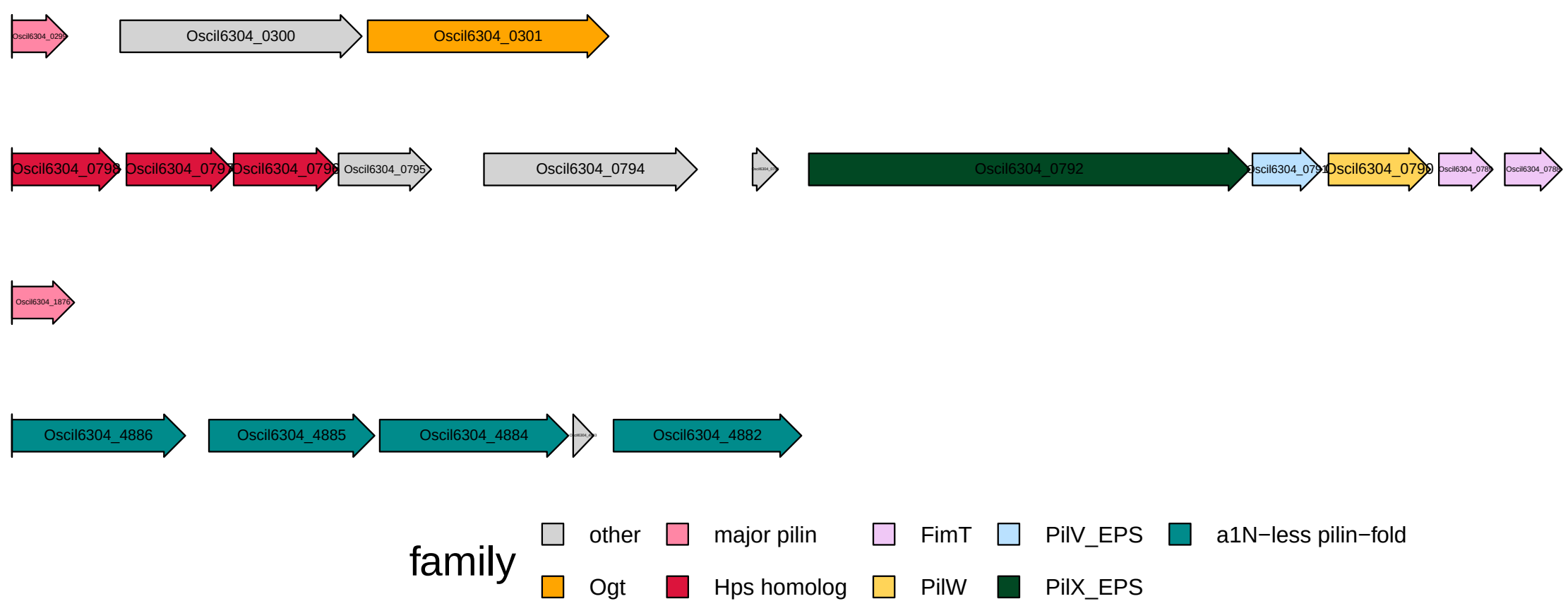

locus	gene	family	start	end
454	Oscil6304_0299	major pilin	353891	354406
454	Oscil6304_0300	other	354894	357134
454	Oscil6304_0301	Ogt	357188	359422
456	Oscil6304_0788	FimT	974692	975222
456	Oscil6304_0789	FimT	975333	975833
456	Oscil6304_0790	PilW	975912	976856
456	Oscil6304_0791	PilV_EPS	976917	977561
456	Oscil6304_0792	PilX_EPS	977585	981673
456	Oscil6304_0793	other	981958	982194
456	Oscil6304_0794	other	982708	984684
456	Oscil6304_0795	other	985172	986032
456	Oscil6304_0796	Hps homolog	986039	987004
456	Oscil6304_0797	Hps homolog	987015	987998
456	Oscil6304_0798	Hps homolog	988052	989059
459	Oscil6304_1876	major pilin	2278455	2279033
470	Oscil6304_4882	a1N−less pilin−fold	6214570	6216312
470	Oscil6304_4883	other	6216498	6216686
470	Oscil6304_4884	a1N−less pilin−fold	6216731	6218479
470	Oscil6304_4885	a1N−less pilin−fold	6218527	6220062
470	Oscil6304_4886	a1N−less pilin−fold	6220281	6221888

Oscillatoria nigro−viridis PCC 7112

GCA_000317475.1

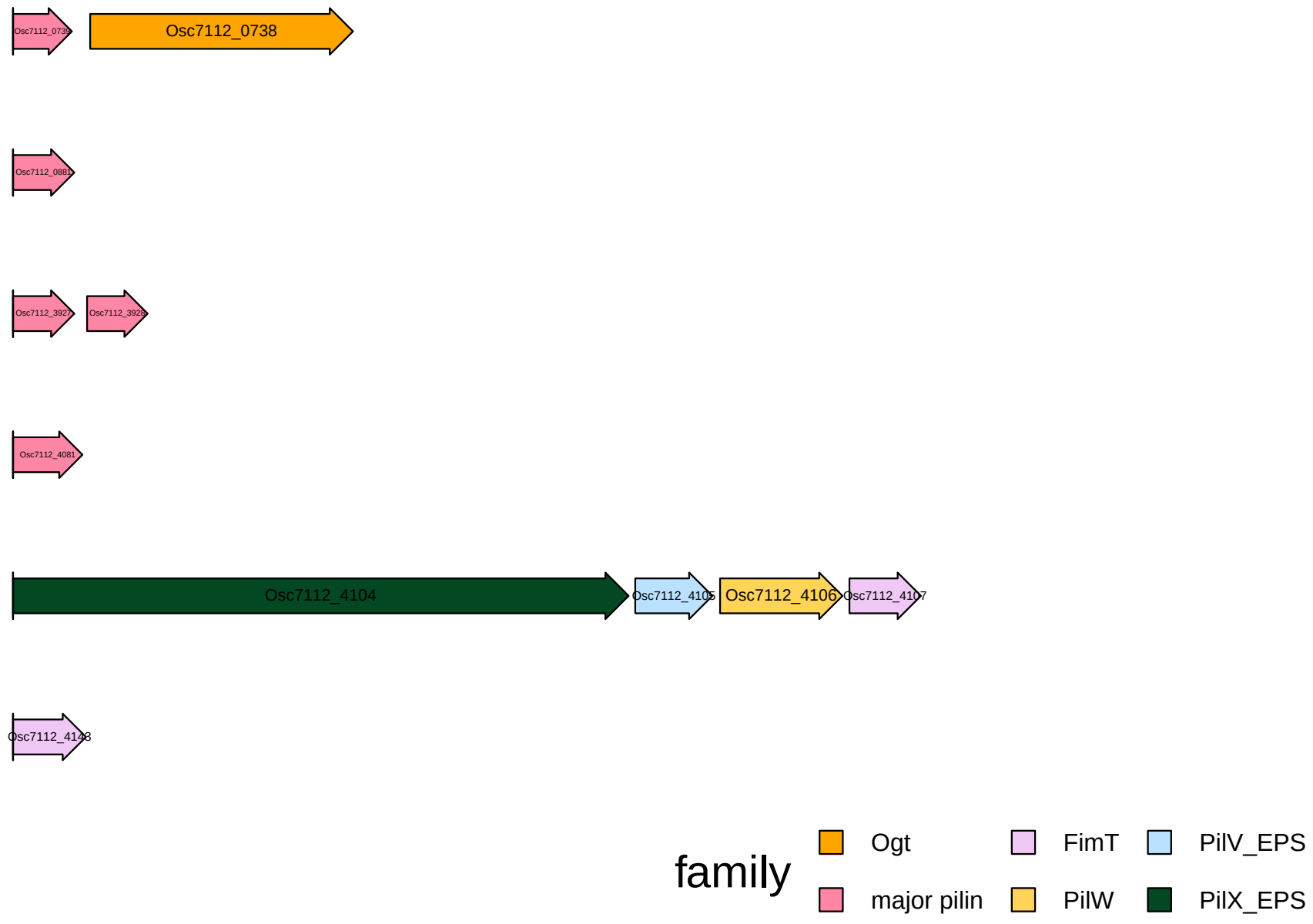

locus	gene	family	start	end
511	Osc7112_0738	Ogt	853921	856155
511	Osc7112_0739	major pilin	856310	856810
513	Osc7112_0881	major pilin	1009774	1010295
526	Osc7112_3927	major pilin	4635103	4635624
526	Osc7112_3928	major pilin	4635732	4636247
527	Osc7112_4081	major pilin	4833651	4834241
528	Osc7112_4104	PilX_EPS	4855189	4860420
528	Osc7112_4105	PilV_EPS	4860476	4861132
528	Osc7112_4106	PilW	4861197	4862237
528	Osc7112_4107	FimT	4862296	4862904
530	Osc7112_4143	FimT	4908027	4908647

Oxynema aestuarii AP17

GCA_012295525.1

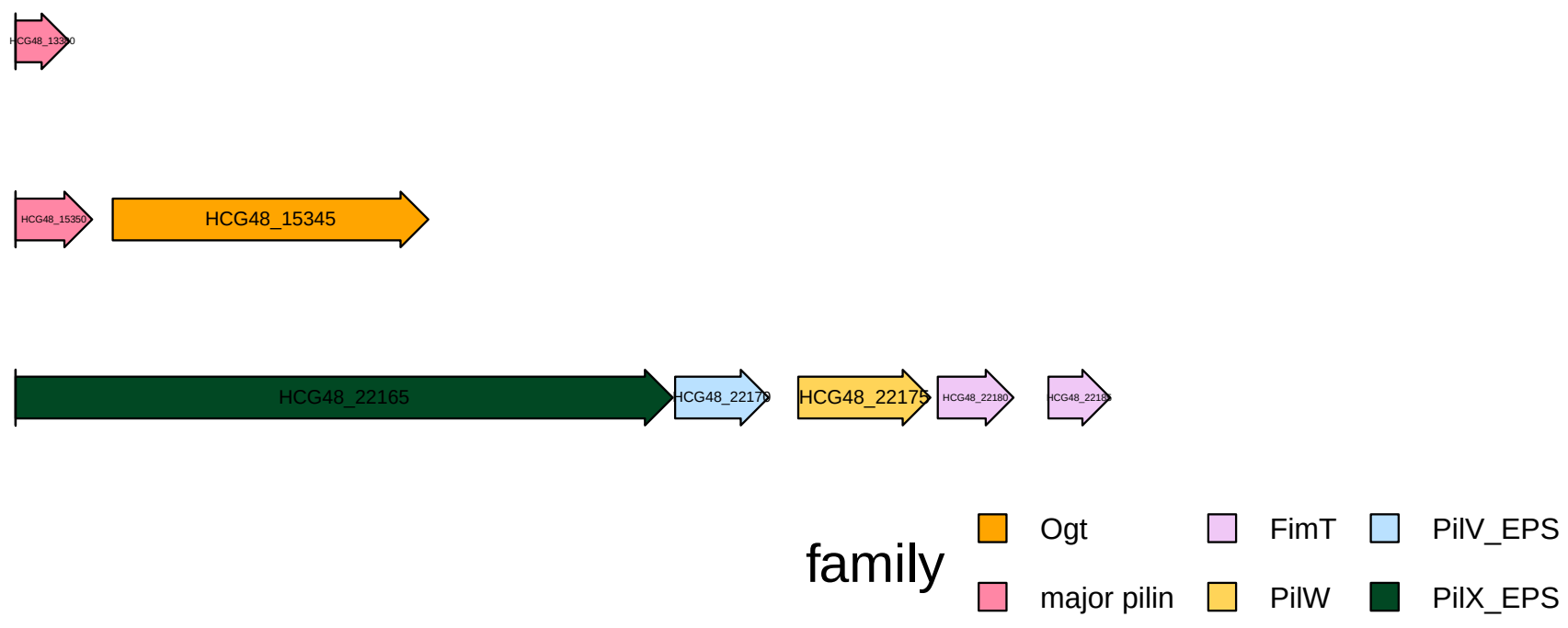

locus	gene	family	start	end
1594	HCG48_13380	major pilin	3283594	3283977
1595	HCG48_15345	Ogt	3778165	3780411
1595	HCG48_15350	major pilin	3780557	3781102
1601	HCG48_22165	PilX_EPS	5551320	5555996
1601	HCG48_22170	PilV_EPS	5556013	5556678
1601	HCG48_22175	PilW	5556889	5557830
1601	HCG48_22180	FimT	5557882	5558421
1601	HCG48_22185	FimT	5558669	5559109

Parasynechococcus marenigrum (strain WH8102)

GCA_000195975.1

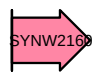

family ■ major pilin

locus	gene	family	start	end
373	SYNW2160	major pilin	2054900	2055412

Phormidesmis priestleyi ULC007

GCA_003003695.1

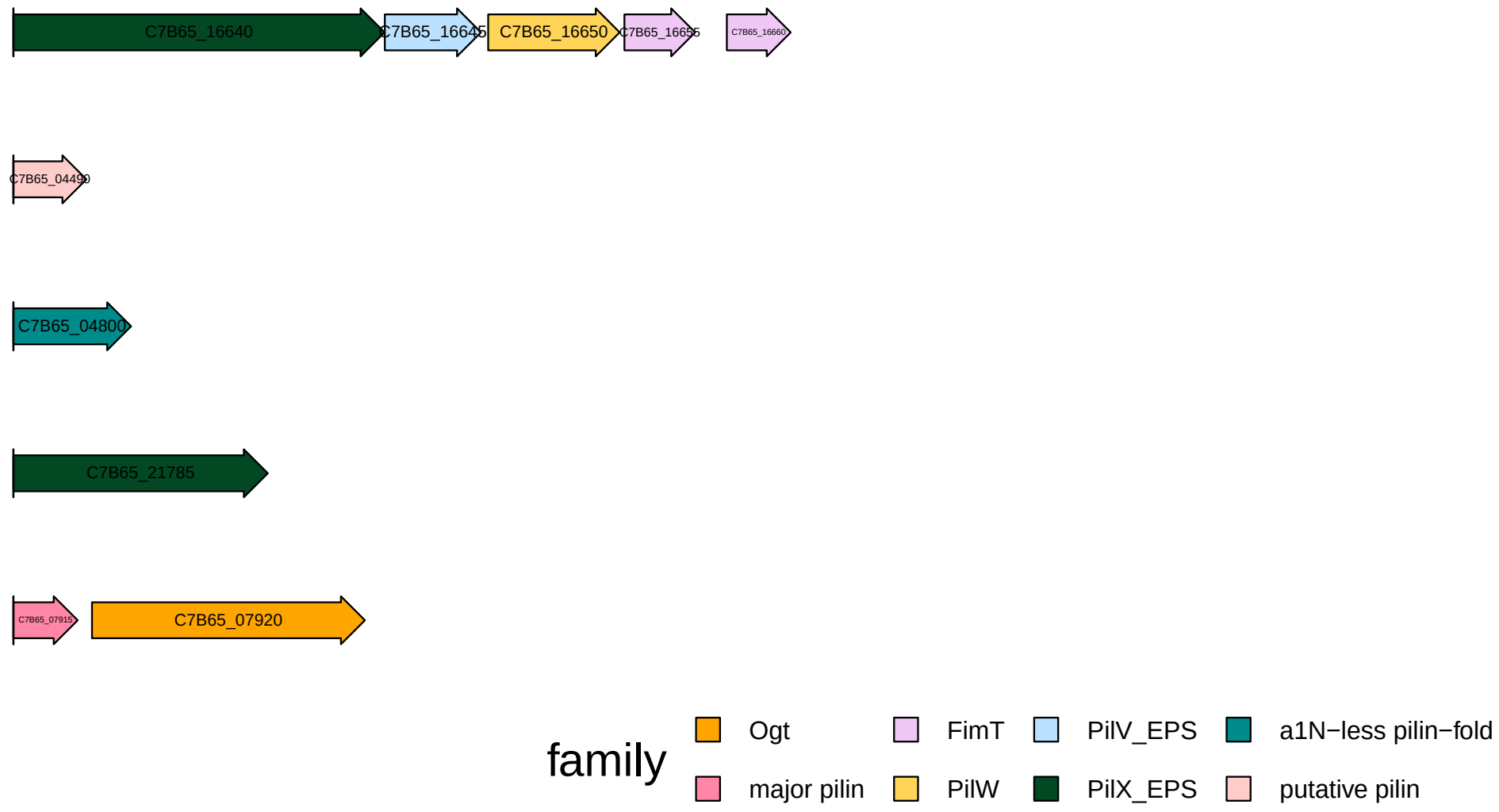

locus	gene	family	start	end
1205	C7B65_16640	PilX_EPS	123	3185
1205	C7B65_16645	PilV_EPS	3187	3981
1205	C7B65_16650	PilW	4040	5125
1205	C7B65_16655	FimT	5164	5748
1205	C7B65_16660	FimT	6009	6536
1208	C7B65_04490	putative pilin	113873	114475
1209	C7B65_04800	a1N−less pilin−fold	177910	178881
1210	C7B65_21785	PilX_EPS	37147	39246
1216	C7B65_07915	major pilin	118940	119470
1216	C7B65_07920	Ogt	119588	121840

Phormidium ambiguum IAM M−71

GCA_001904725.1

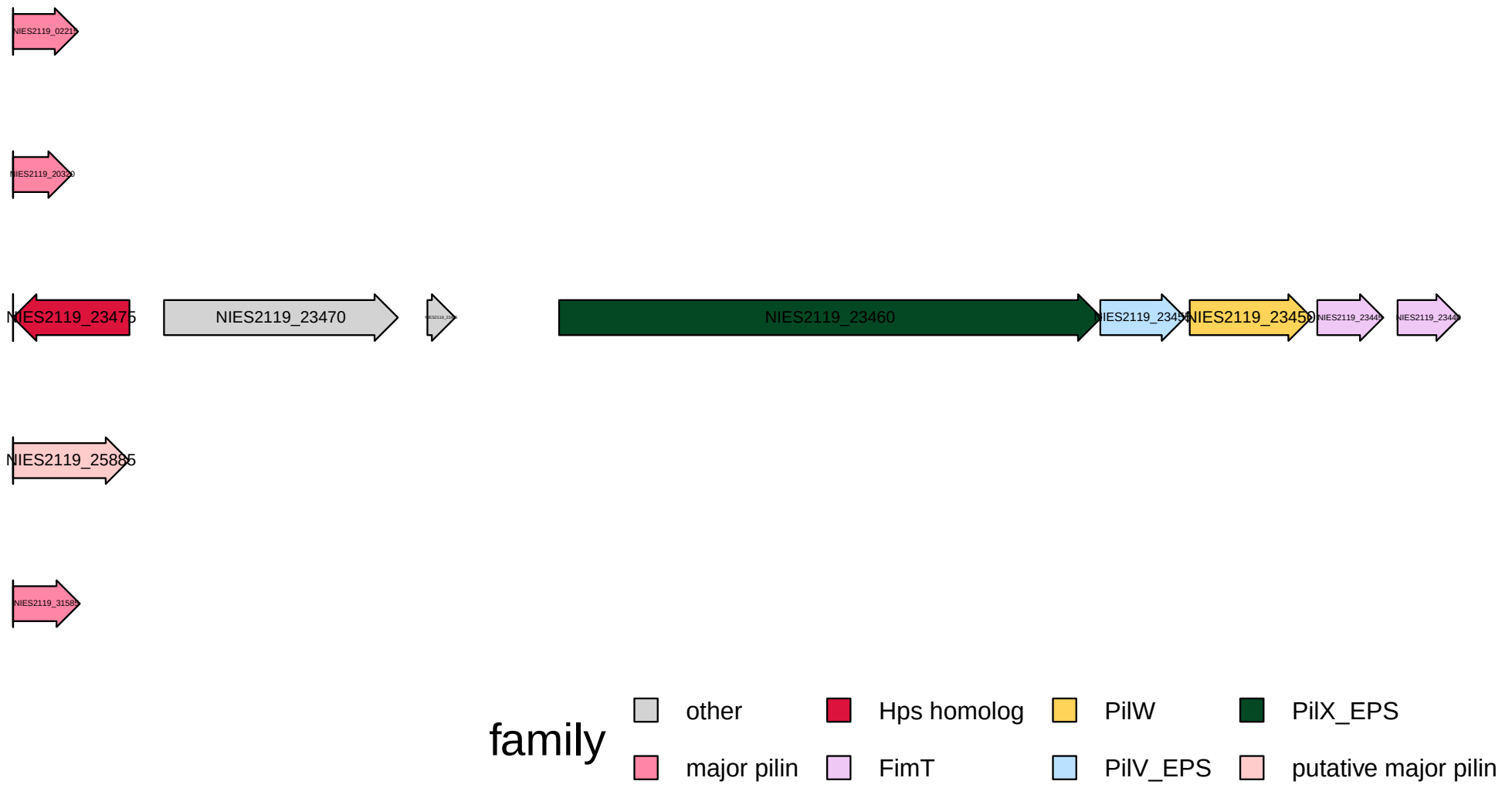

locus	gene	family	start	end
994	NIES2119_02215	major pilin	125781	126332
997	NIES2119_20320	major pilin	4839	5336
998	NIES2119_23440	FimT	33889	34416
998	NIES2119_23445	FimT	34536	35096
998	NIES2119_23450	PilW	35143	36177
998	NIES2119_23455	PilV_EPS	36216	36935
998	NIES2119_23460	PilX_EPS	36926	41521
998	NIES2119_23465	other	42401	42637
998	NIES2119_23470	other	42887	44869
998	NIES2119_23475	Hps homolog	45162	46148
1000	NIES2119_25885	putative major pilin	35497	36480
1007	NIES2119_31585	major pilin	8483	9049

Phormidium tenue NIES−30

GCA_001904775.1

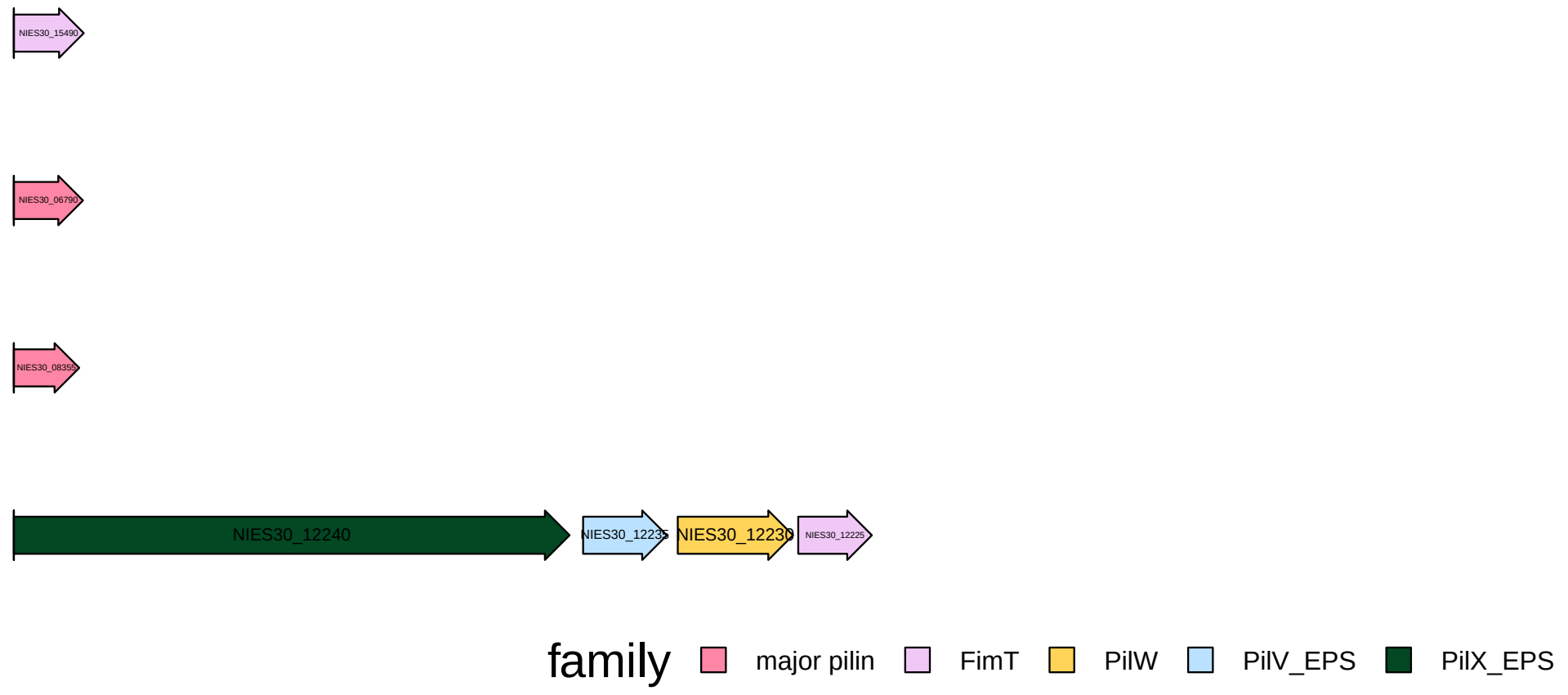

locus	gene	family	start	end
1016	NIES30_15490	FimT	153771	154331
1023	NIES30_06790	major pilin	191419	191973
1025	NIES30_08355	major pilin	247901	248425
1029	NIES30_12225	FimT	94882	95472
1029	NIES30_12230	PilW	95514	96437
1029	NIES30_12235	PilV_EPS	96524	97195
1029	NIES30_12240	PilX_EPS	97303	101754

Picosynechococcus sp. (strain ATCC 27264 / PCC 7002 / PR−6) (Agmenellum quadruplicatum)

GCA_000019485.1

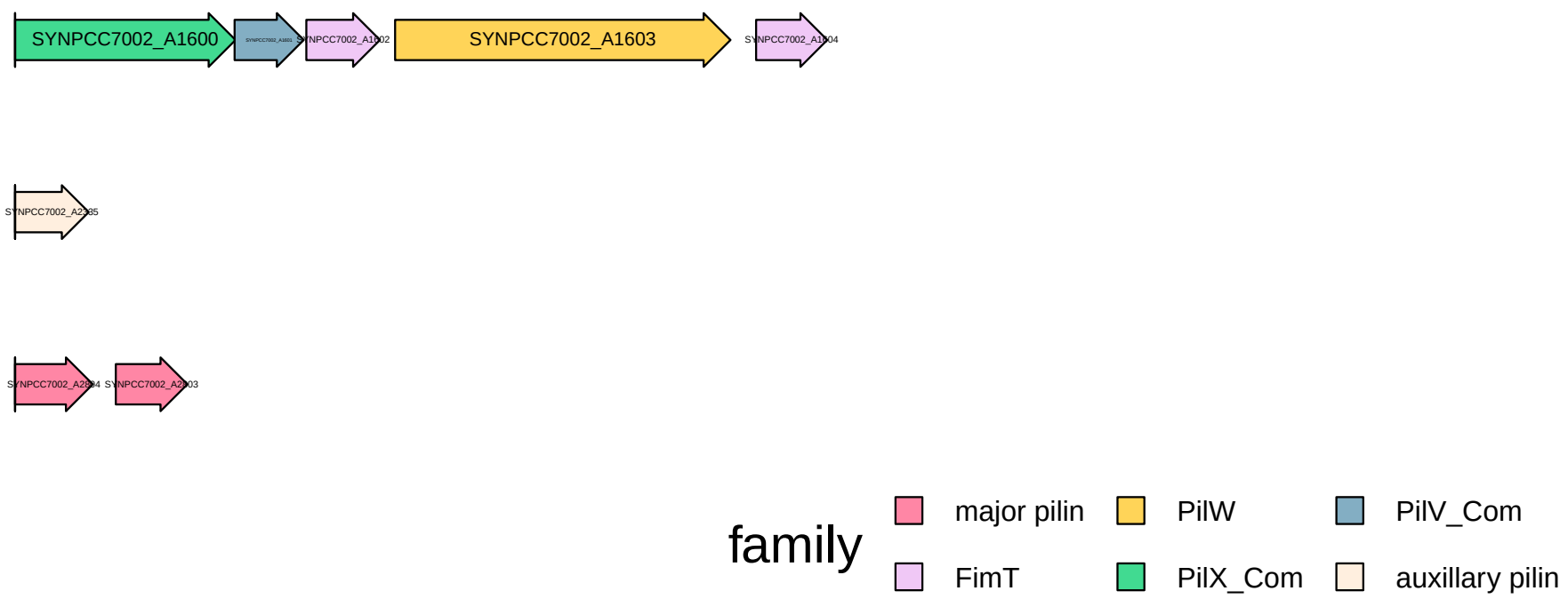

locus	gene	family	start	end
226	SYNPCC7002_A1600	PilX_Com	1678748	1680370
226	SYNPCC7002_A1601	PilV_Com	1680363	1680872
226	SYNPCC7002_A1602	FimT	1680891	1681433
226	SYNPCC7002_A1603	PilW	1681544	1684012
226	SYNPCC7002_A1604	FimT	1684200	1684724
230	SYNPCC7002_A2335	auxillary pilin	2411718	2412260
232	SYNPCC7002_A2803	major pilin	2927084	2927611
232	SYNPCC7002_A2804	major pilin	2927780	2928352

Planktothrix agardhii (Oscillatoria agardhii)

GCA_904830855.1

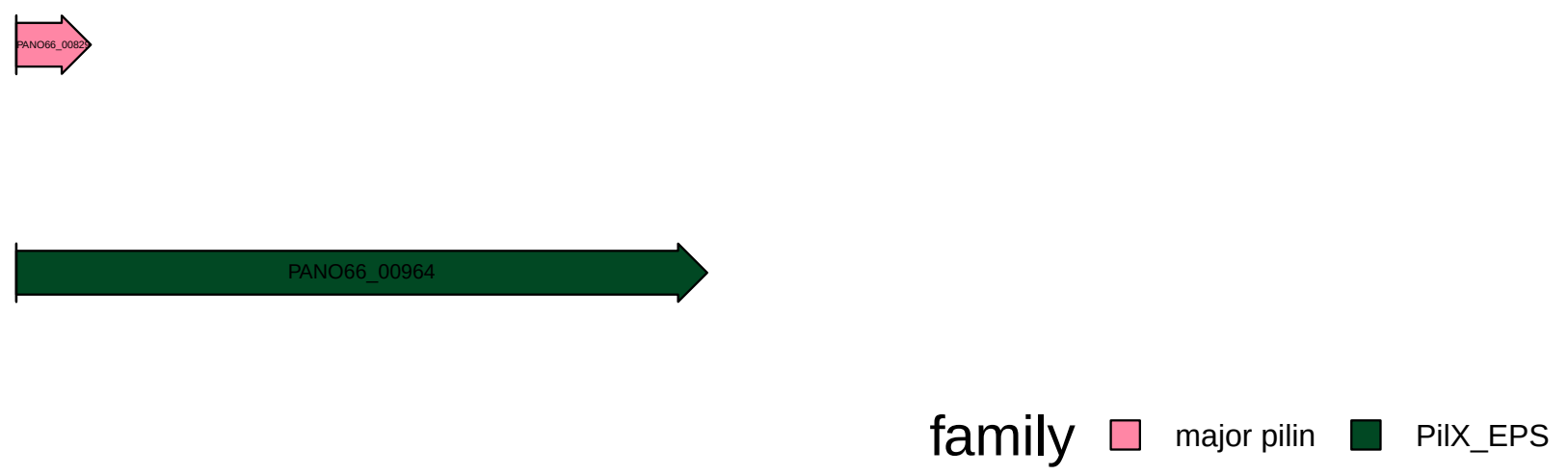

locus	gene	family	start	end
1728	PANO66_00829	major pilin	991662	992168
1729	PANO66_00964	PilX_EPS	1134070	1138761

Planktothrix paucivesiculata PCC 9631

GCA_900009265.2

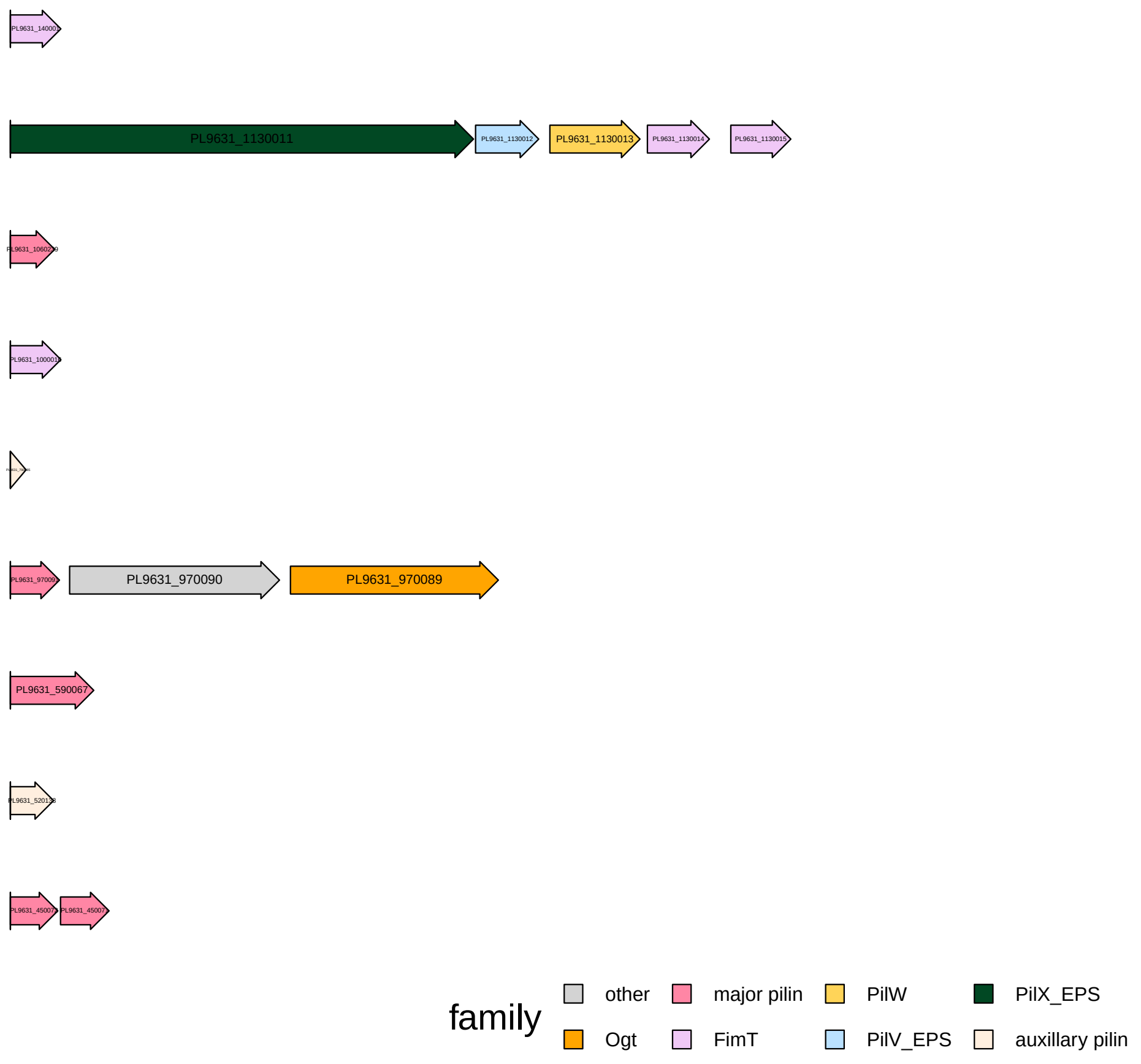

locus	gene	family	start	end
1669	PL9631_140001	FimT	160	699
1670	PL9631_1130011	PilX_EPS	155167	160110
1670	PL9631_1130012	PilV_EPS	160132	160806
1670	PL9631_1130013	PilW	160925	161887
1670	PL9631_1130014	FimT	161966	162628
1670	PL9631_1130015	FimT	162856	163497
1673	PL9631_1060239	major pilin	582056	582529
1675	PL9631_1000016	FimT	83137	83676
1678	PL9631_790085	auxillary pilin	491006	491173
1681	PL9631_970089	Ogt	176944	179163
1681	PL9631_970090	other	179280	181520
1681	PL9631_970091	major pilin	181628	182152
1684	PL9631_590067	major pilin	66551	67441
1687	PL9631_520133	auxillary pilin	140294	140755
1689	PL9631_450071	major pilin	124520	125038
1689	PL9631_450072	major pilin	125067	125573

Planktothrix serta PCC 8927

GCA_900010725.2

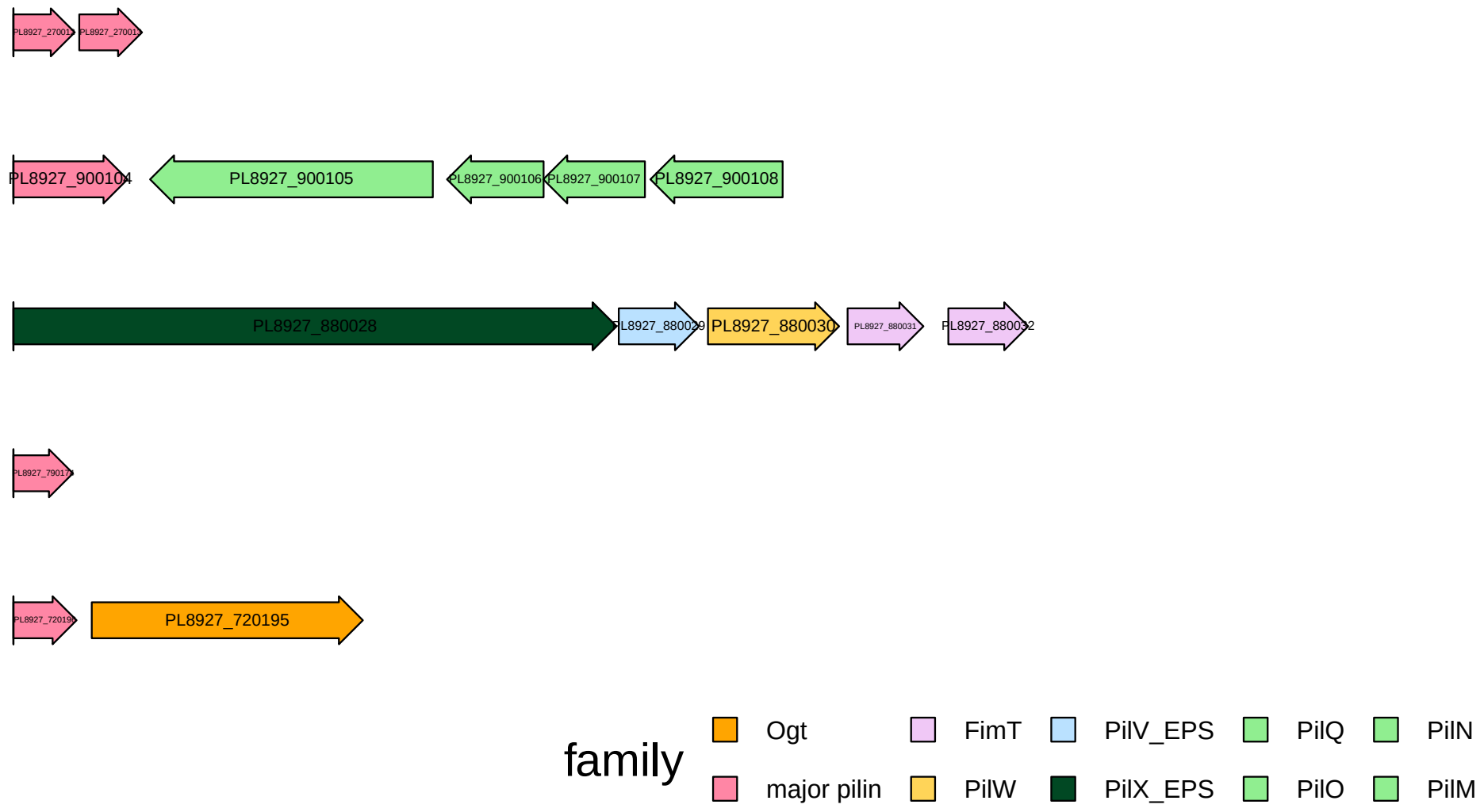

locus	gene	family	start	end
1690	PL8927_270012	major pilin	9304	9810
1690	PL8927_270013	major pilin	9847	10365
1696	PL8927_900104	major pilin	113556	114497
1696	PL8927_900105	PilQ	114683	117016
1696	PL8927_900106	PilO	117133	117930
1696	PL8927_900107	PilN	117927	118766
1696	PL8927_900108	PilM	118811	119902
1700	PL8927_880028	PilX_EPS	41033	46009
1700	PL8927_880029	PilV_EPS	46027	46689
1700	PL8927_880030	PilW	46763	47845
1700	PL8927_880031	FimT	47915	48541
1700	PL8927_880032	FimT	48746	49405
1702	PL8927_790174	major pilin	205066	205557
1704	PL8927_720195	Ogt	222825	225062
1704	PL8927_720196	major pilin	225187	225708

Prochlorothrix hollandica PCC 9006 = CALU 1027

GCA_000341585.2

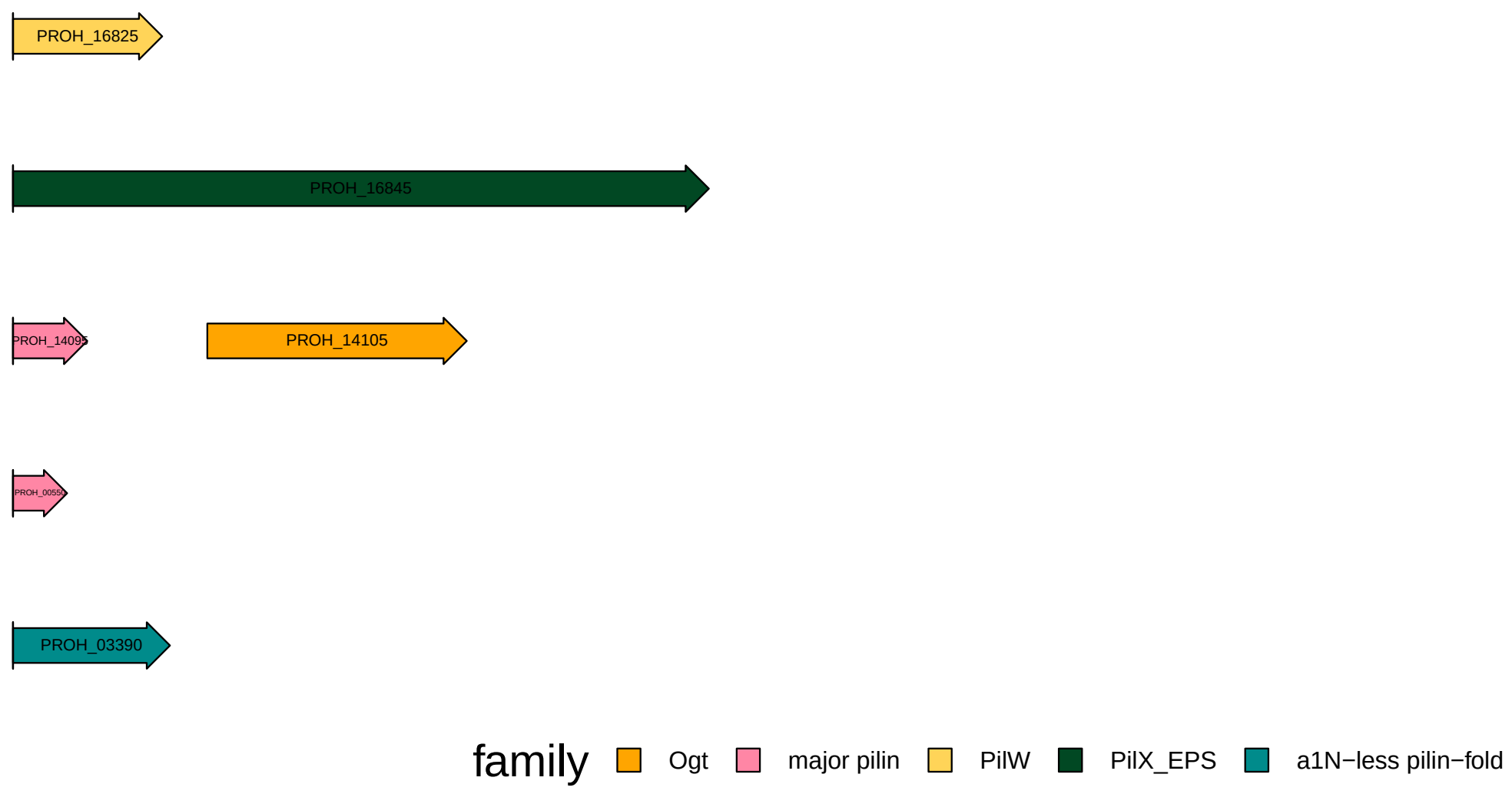

locus	gene	family	start	end
694	PROH_16825	PilW	35966	37237
695	PROH_16845	PilX_EPS	38896	44823
699	PROH_14095	major pilin	205868	206500
699	PROH_14105	Ogt	207523	209733
705	PROH_00550	major pilin	122276	122737
707	PROH_03390	a1N−less pilin−fold	864085	865422

Pseudanabaena biceps PCC 7429

GCA_000332215.1

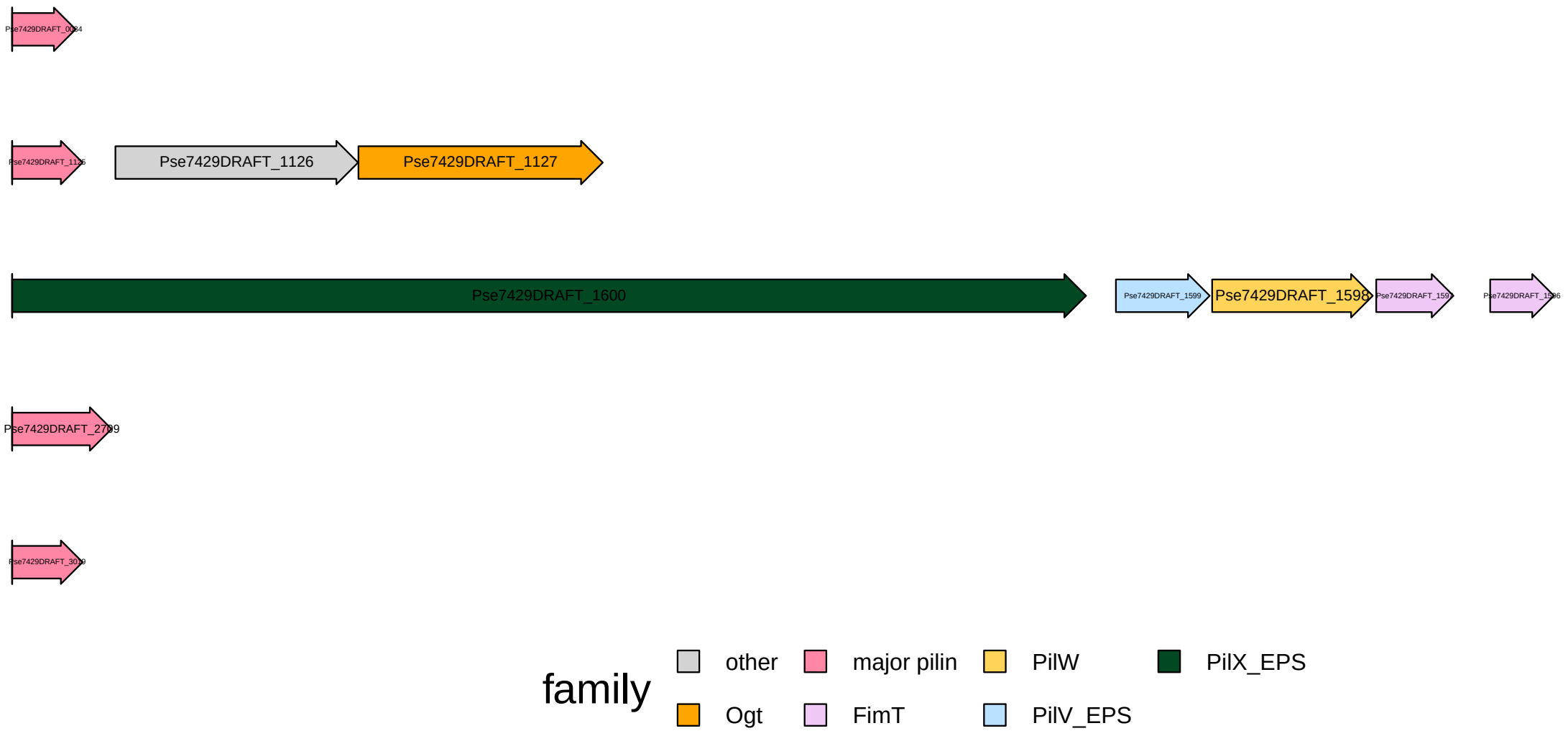

locus	gene	family	start	end
669	Pse7429DRAFT_0084	major pilin	20801	21379
675	Pse7429DRAFT_1125	major pilin	259	900
675	Pse7429DRAFT_1126	other	1199	3409
675	Pse7429DRAFT_1127	Ogt	3412	5637
677	Pse7429DRAFT_1596	FimT	1821	2402
677	Pse7429DRAFT_1597	FimT	2736	3440
677	Pse7429DRAFT_1598	PilW	3471	4934
677	Pse7429DRAFT_1599	PilV_EPS	4956	5810
677	Pse7429DRAFT_1600	PilX_EPS	6082	15861
683	Pse7429DRAFT_2709	major pilin	11222	12127
684	Pse7429DRAFT_3019	major pilin	597	1238

Rippkaea orientalis (strain PCC 8801 / RF−1) (Cyanothece sp. (strain PCC 8801))

GCA_000021805.1

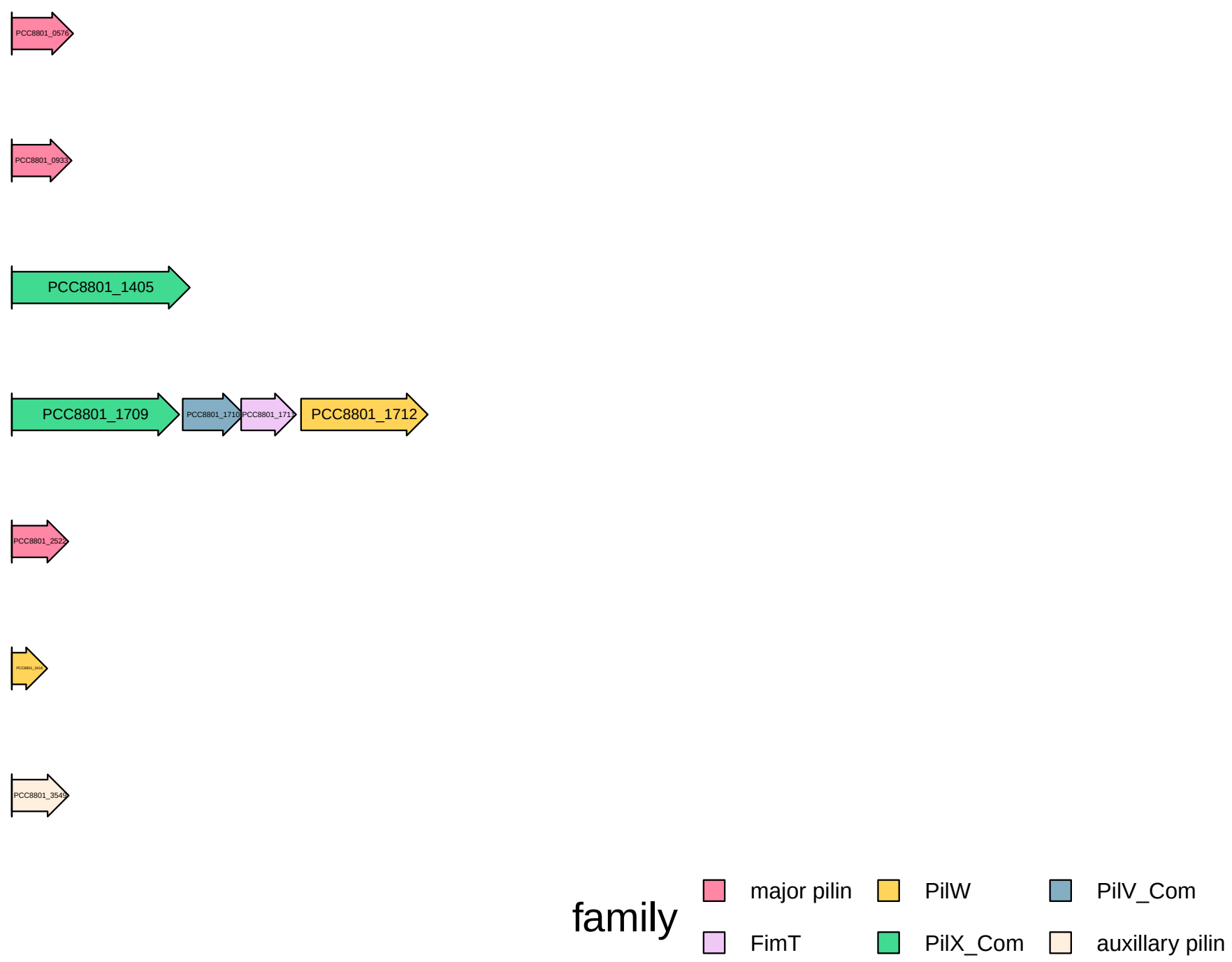

locus	gene	family	start	end
252	PCC8801_0576	major pilin	607219	607794
255	PCC8801_0933	major pilin	983467	984027
256	PCC8801_1405	PilX_Com	1469181	1470848
259	PCC8801_1709	PilX_Com	1794061	1795629
259	PCC8801_1710	PilV_Com	1795661	1796236
259	PCC8801_1711	FimT	1796208	1796723
259	PCC8801_1712	PilW	1796769	1797956
262	PCC8801_2522	major pilin	2615065	2615595
266	PCC8801_3416	PilW	3563247	3563579
268	PCC8801_3549	auxillary pilin	3697639	3698172

Rubidibacter lacunae KORDI 51−2

GCA_000473895.1

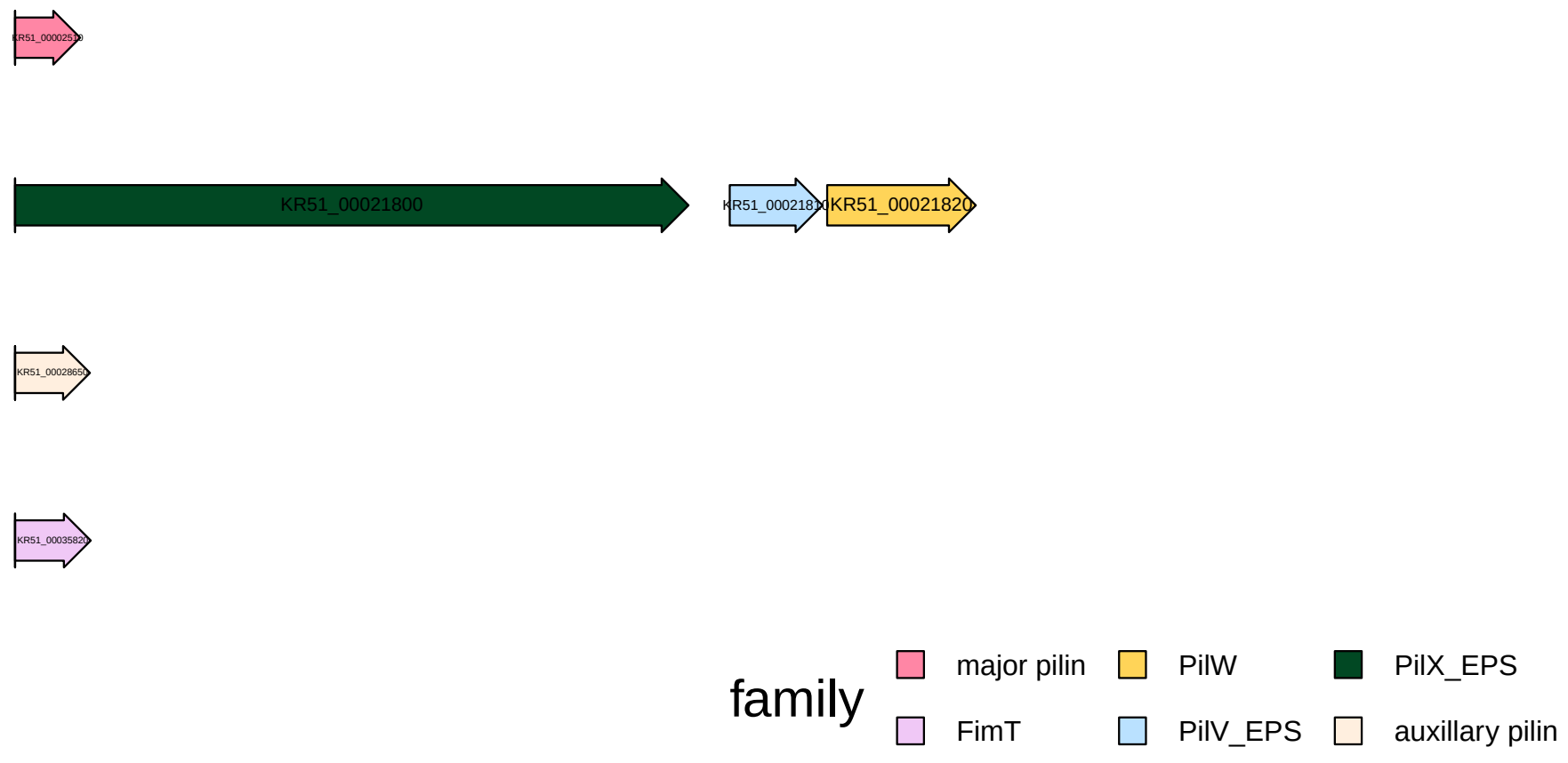

locus	gene	family	start	end
727	KR51_00002510	major pilin	67685	68164
734	KR51_00021800	PilX_EPS	44154	49109
734	KR51_00021810	PilV_EPS	49412	50095
734	KR51_00021820	PilW	50129	51223
738	KR51_00028650	auxillary pilin	32243	32794
743	KR51_00035820	FimT	53787	54344

Scytonema hofmannii PCC 7110

GCA_000346485.2

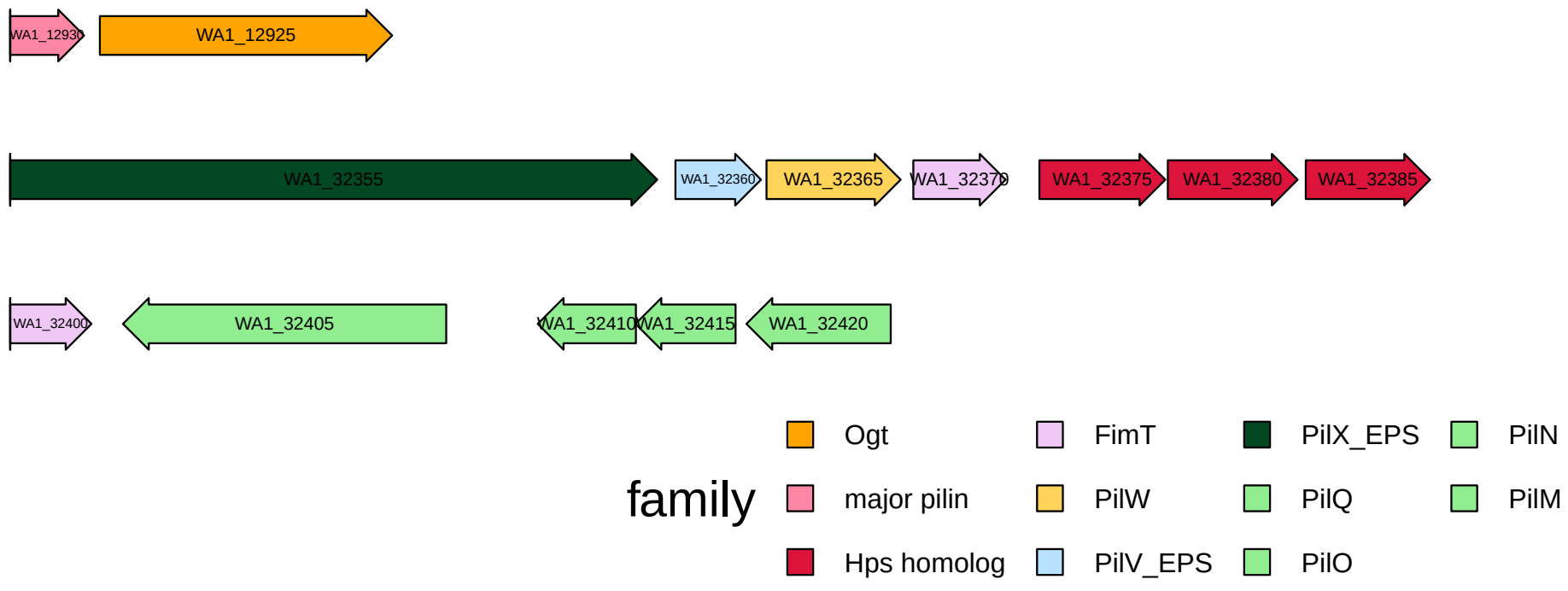

locus	gene	family	start	end
712	WA1_12925	Ogt	3013829	3016069
712	WA1_12930	major pilin	3016190	3016756
721	WA1_32355	PilX_EPS	7694326	7699284
721	WA1_32360	PilV_EPS	7699423	7700079
721	WA1_32365	PilW	7700121	7701149
721	WA1_32370	FimT	7701246	7701953
721	WA1_32375	Hps homolog	7702212	7703177
721	WA1_32380	Hps homolog	7703196	7704191
721	WA1_32385	Hps homolog	7704253	7705206
722	WA1_32400	FimT	7707204	7707827
722	WA1_32405	PilQ	7708068	7710545
722	WA1_32410	PilO	7711246	7711998
722	WA1_32415	PilN	7711995	7712762
722	WA1_32420	PilM	7712845	7713951

Sphaerospermopsis reniformis

GCA_005402885.1

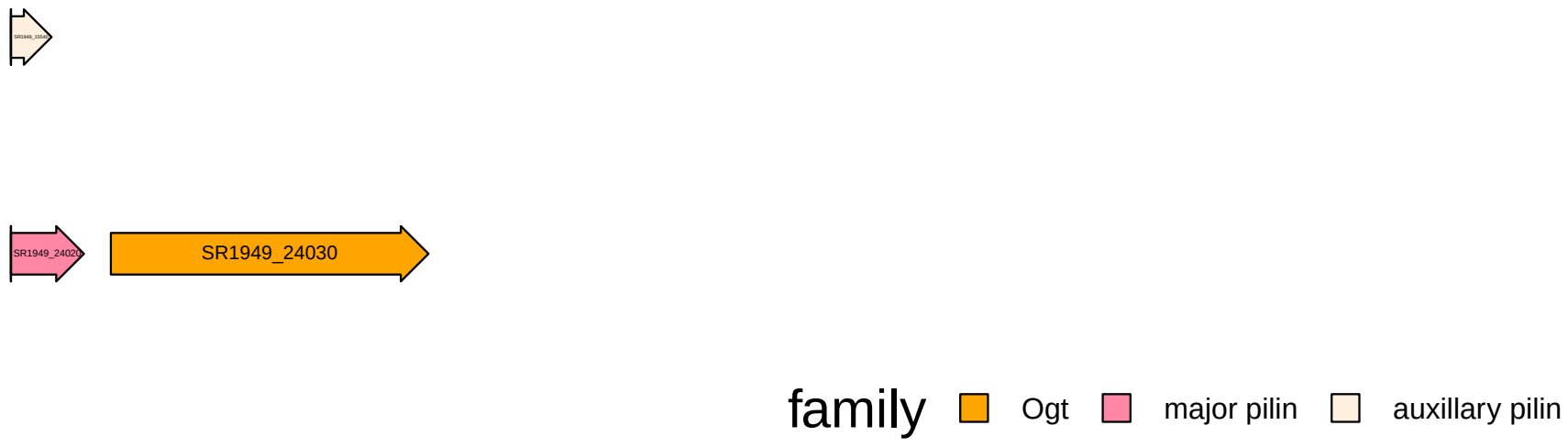

locus	gene	family	start	end
1500	SR1949_15540	auxillary pilin	2449	2739
1504	SR1949_24020	major pilin	2482	3003
1504	SR1949_24030	Ogt	3195	5462

Stanieria cyanosphaera (strain ATCC 29371 / PCC 7437)

GCA_000317575.1

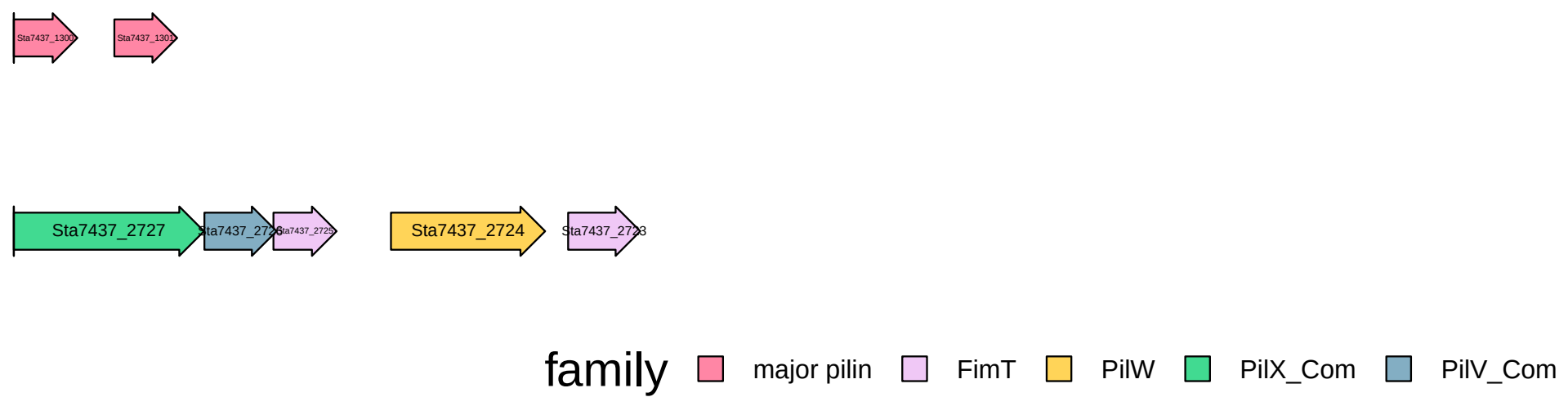

locus	gene	family	start	end
599	Sta7437_1300	major pilin	1453209	1453718
599	Sta7437_1301	major pilin	1454014	1454517
602	Sta7437_2723	FimT	3098499	3099077
602	Sta7437_2724	PilW	3099261	3100496
602	Sta7437_2725	FimT	3100931	3101437
602	Sta7437_2726	PilV_Com	3101412	3101990
602	Sta7437_2727	PilX_Com	3101993	3103516

Stenomitos frigidus ULC18

GCA_003003795.1

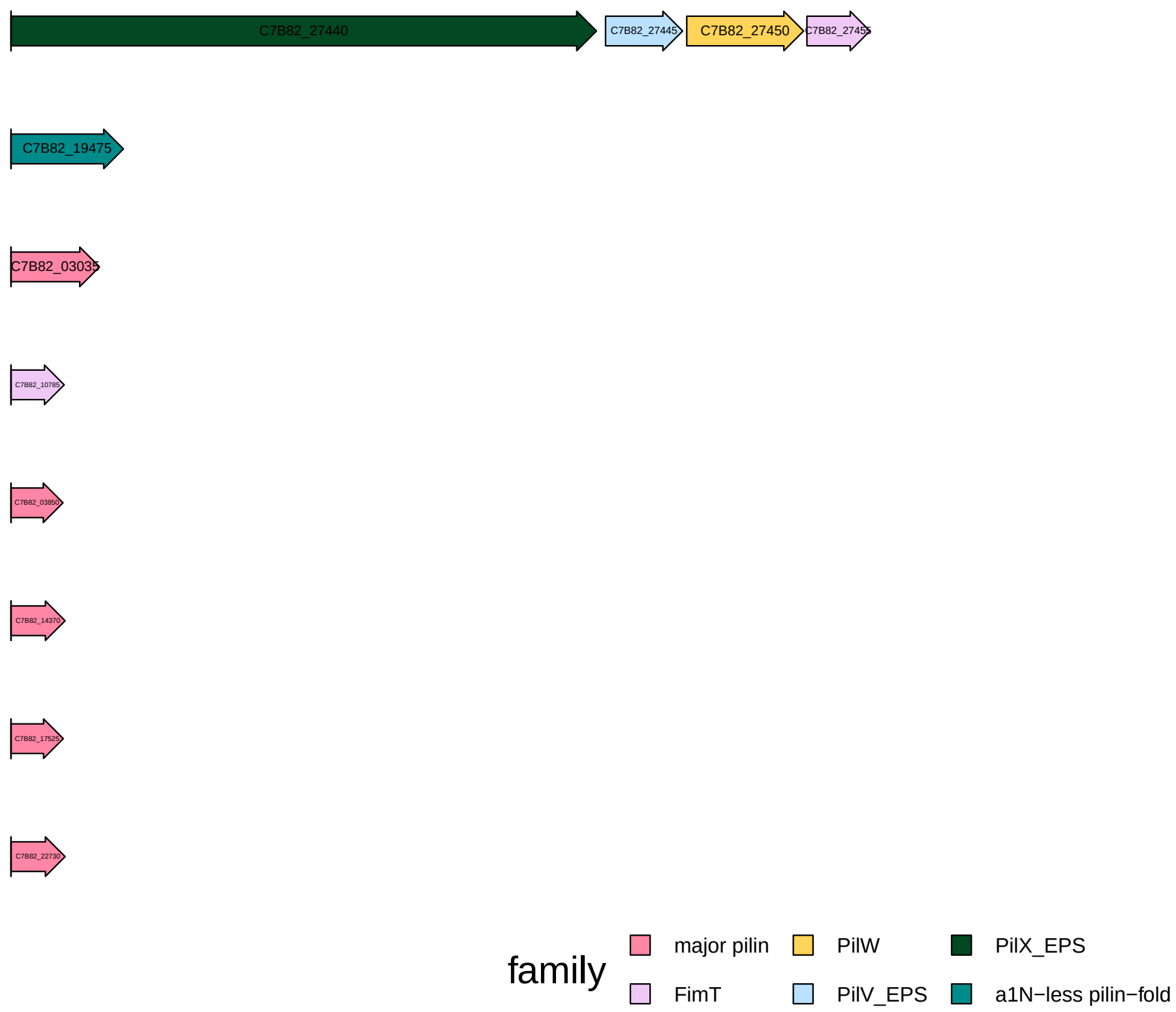

locus	gene	family	start	end
1247	C7B82_27440	PilX_EPS	29228	35098
1247	C7B82_27445	PilV_EPS	35189	35962
1247	C7B82_27450	PilW	36003	37175
1247	C7B82_27455	FimT	37208	37840
1254	C7B82_19475	a1N−less pilin−fold	2505	3632
1256	C7B82_03035	major pilin	242552	243439
1257	C7B82_10785	FimT	11519	12052
1258	C7B82_03850	major pilin	88489	89010
1263	C7B82_14370	major pilin	54671	55213
1265	C7B82_17525	major pilin	18296	18820
1272	C7B82_22730	major pilin	71902	72444

Synechococcus elongatus (strain ATCC 33912 / PCC 7942 / FACHB−805) (Anacystis nidulans R2)

GCA_000012525.1

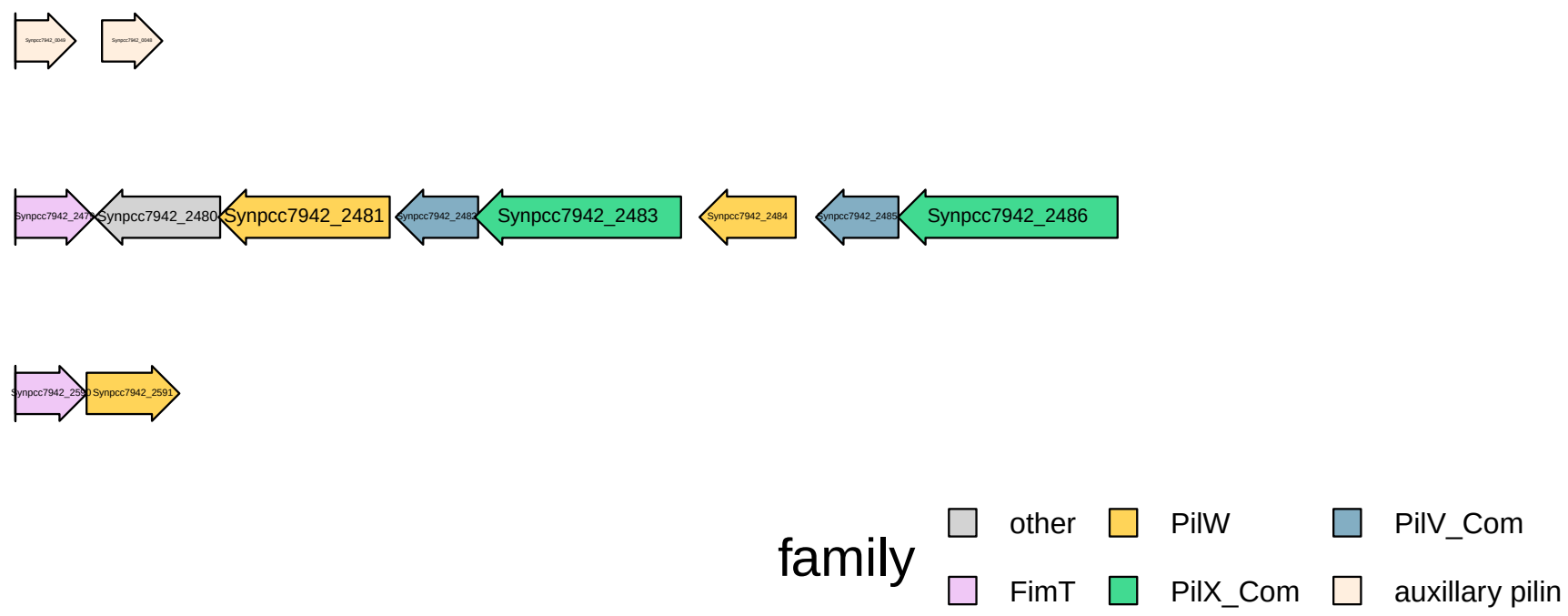

locus	gene	family	start	end
116	Synpcc7942_0048	auxillary pilin	48927	49361
116	Synpcc7942_0049	auxillary pilin	49550	49984
130	Synpcc7942_2479	FimT	2560375	2560941
130	Synpcc7942_2480	other	2560946	2561848
130	Synpcc7942_2481	PilW	2561836	2563068
130	Synpcc7942_2482	PilV_Com	2563111	2563704
130	Synpcc7942_2483	PilX_Com	2563680	2565164
130	Synpcc7942_2484	PilW	2565297	2565989
130	Synpcc7942_2485	PilV_Com	2566135	2566728
130	Synpcc7942_2486	PilX_Com	2566725	2568305
131	Synpcc7942_2590	FimT	2674093	2674608
131	Synpcc7942_2591	PilW	2674605	2675273

Synechococcus sp. (strain ATCC 27144 / PCC 6301 / SAUG 1402/1) (Anacystis nidulans)

GCA_000010065.1

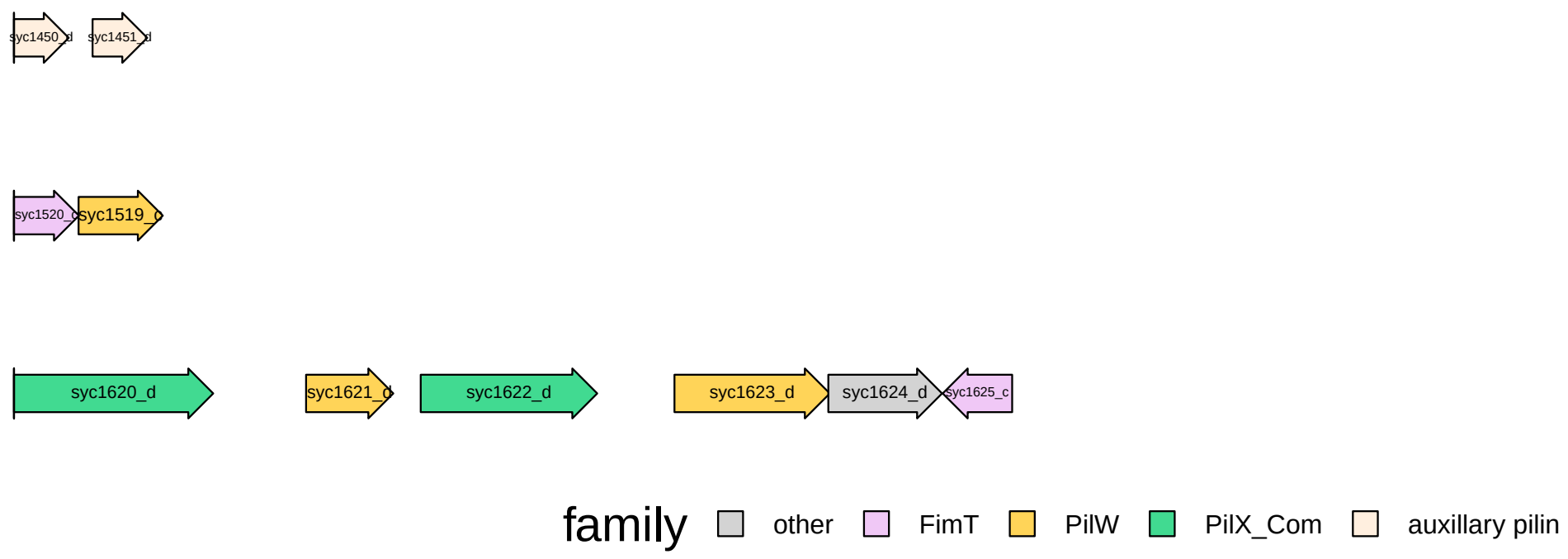

locus	gene	family	start	end
44	syc1450_d	auxillary pilin	1570899	1571333
44	syc1451_d	auxillary pilin	1571522	1571956
45	syc1519_c	PilW	1641513	1642181
45	syc1520_c	FimT	1642178	1642693
46	syc1620_d	PilX_Com	1748479	1750059
46	syc1621_d	PilW	1750795	1751487
46	syc1622_d	PilX_Com	1751704	1753104
46	syc1623_d	PilW	1753716	1754948
46	syc1624_d	other	1754936	1755838
46	syc1625_c	FimT	1755843	1756394

Synechocystis sp. (strain PCC 6803 / Kazusa)

GCA_000009725.1

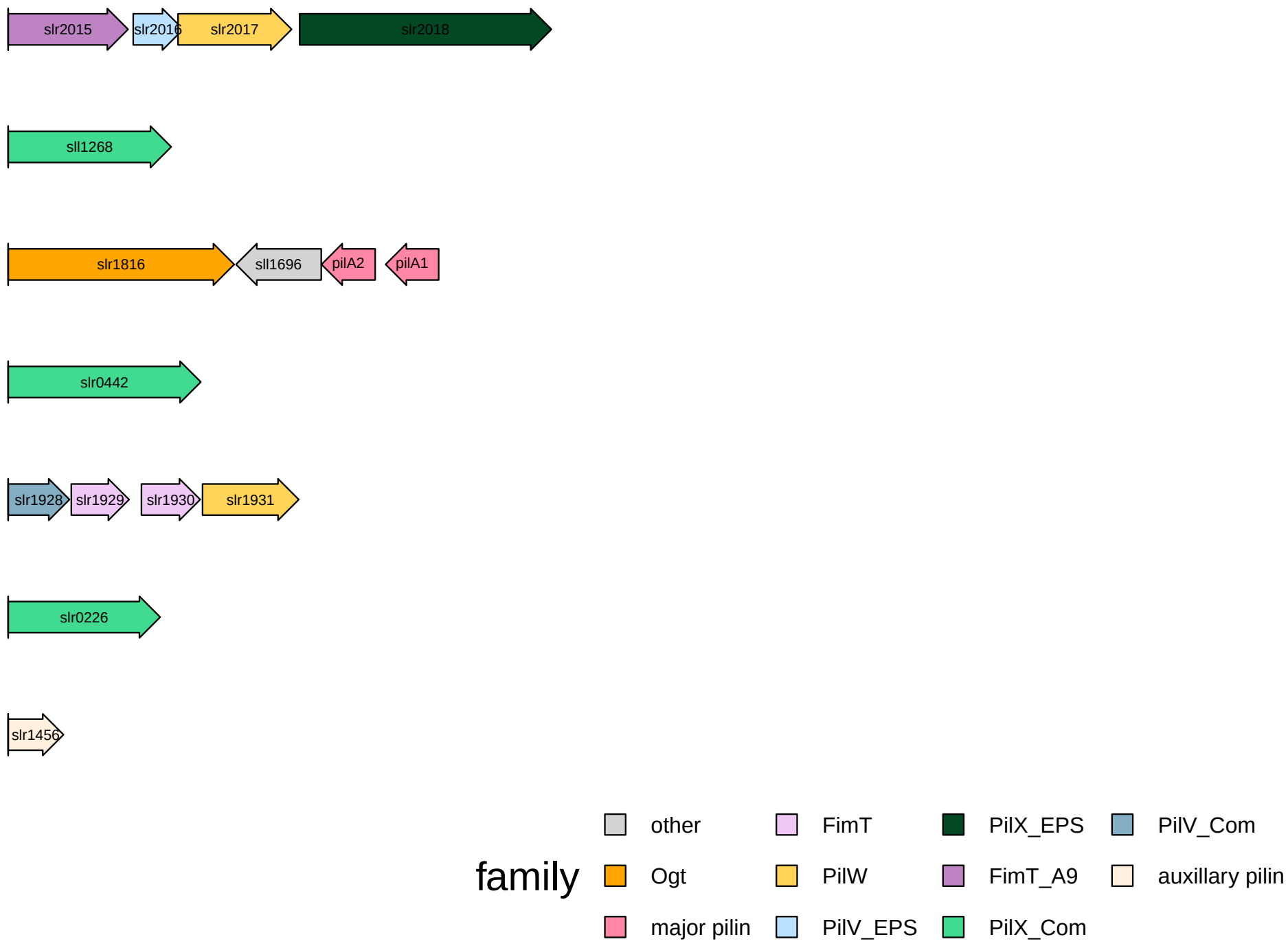

locus	gene	family	start	end
17	slr2015	FimT_A9	756053	757195
17	slr2016	PilV_EPS	757244	757720
17	slr2017	PilW	757671	758753
17	slr2018	PilX_EPS	758829	761228
19	sll1268	PilX_Com	1121627	1123180
21	slr1816	Ogt	1293398	1295551
21	sll1696	other	1295568	1296380
21	pilA2	major pilin	1296382	1296894
21	pilA1	major pilin	1296993	1297499
27	slr0442	PilX_Com	2080885	2082720
29	slr1928	PilV_Com	2221047	2221631
29	slr1929	FimT	2221648	2222199
29	slr1930	FimT	2222316	2222876
29	slr1931	PilW	2222898	2223815
31	slr0226	PilX_Com	2526633	2528081
36	slr1456	auxillary pilin	3302812	3303339

Thermoleptolyngbya sichuanensis A183

GCA_013177315.1

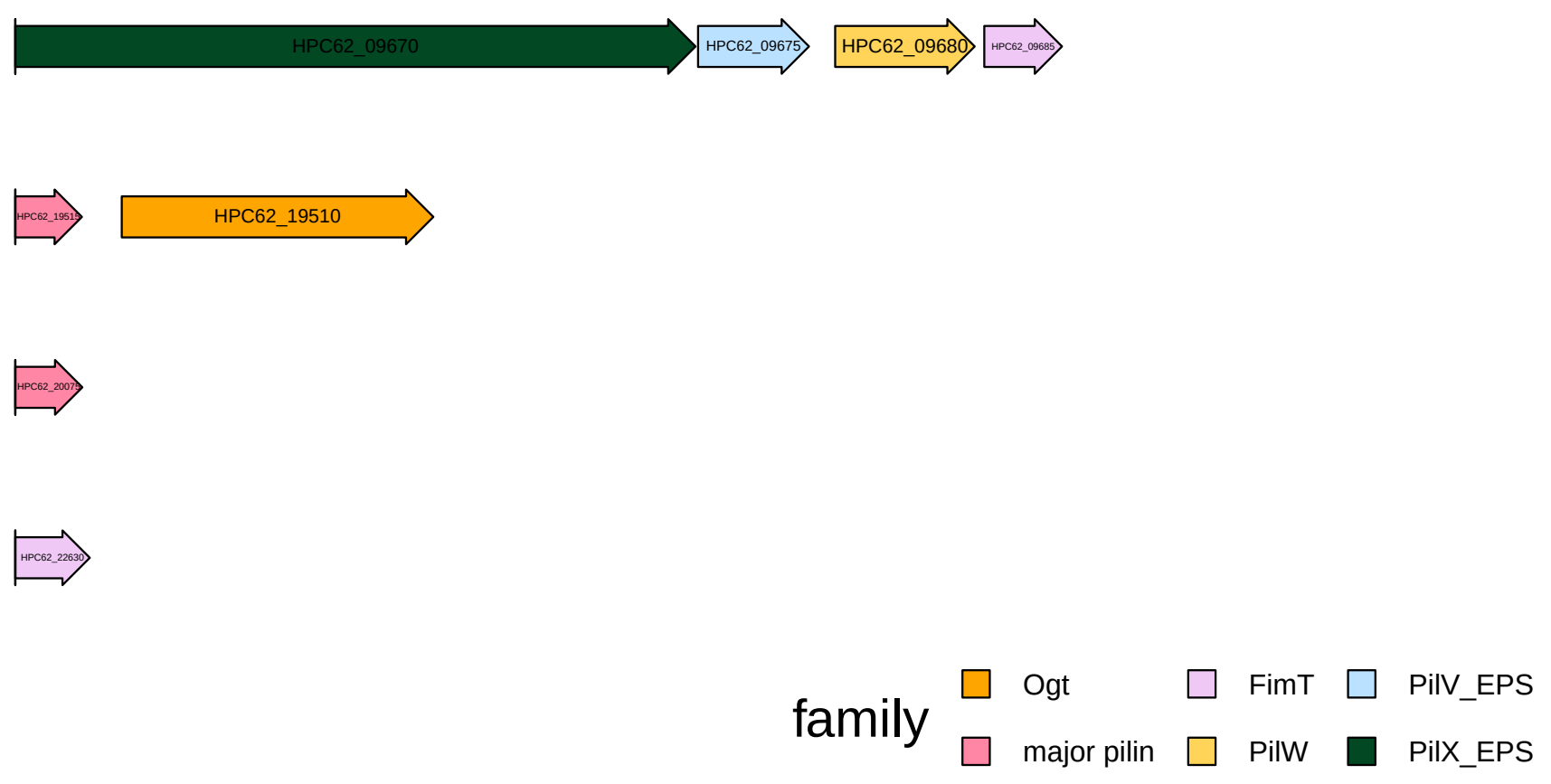

locus	gene	family	start	end
1626	HPC62_09670	PilX_EPS	2293804	2298726
1626	HPC62_09675	PilV_EPS	2298744	2299547
1626	HPC62_09680	PilW	2299736	2300746
1626	HPC62_09685	FimT	2300815	2301378
1632	HPC62_19510	Ogt	4707890	4710145
1632	HPC62_19515	major pilin	4710433	4710915
1634	HPC62_20075	major pilin	4841654	4842139
1637	HPC62_22630	FimT	5440270	5440809

Thermostichus lividus PCC 6715

GCA_002754935.1

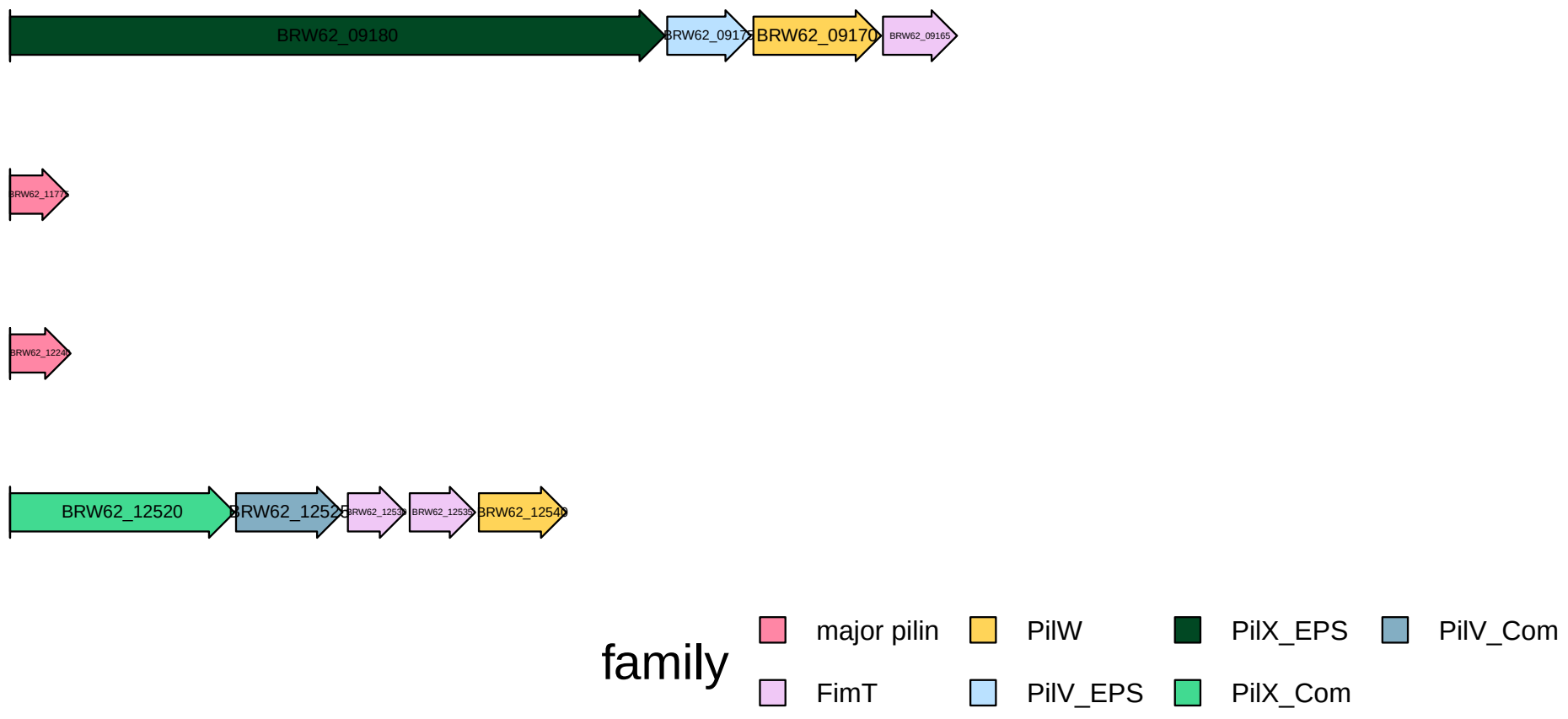

locus	gene	family	start	end
1141	BRW62_09165	FimT	1916693	1917268
1141	BRW62_09170	PilW	1917282	1918274
1141	BRW62_09175	PilV_EPS	1918293	1918943
1141	BRW62_09180	PilX_EPS	1918960	1924044
1144	BRW62_11775	major pilin	2502178	2502627
1145	BRW62_12240	major pilin	2591070	2591540
1146	BRW62_12520	PilX_Com	2647863	2649605
1146	BRW62_12525	PilV_Com	2649617	2650444
1146	BRW62_12530	FimT	2650485	2650931
1146	BRW62_12535	FimT	2650965	2651474
1146	BRW62_12540	PilW	2651502	2652182

Thermosynechococcus sichuanensis E542

GCA_003555505.2

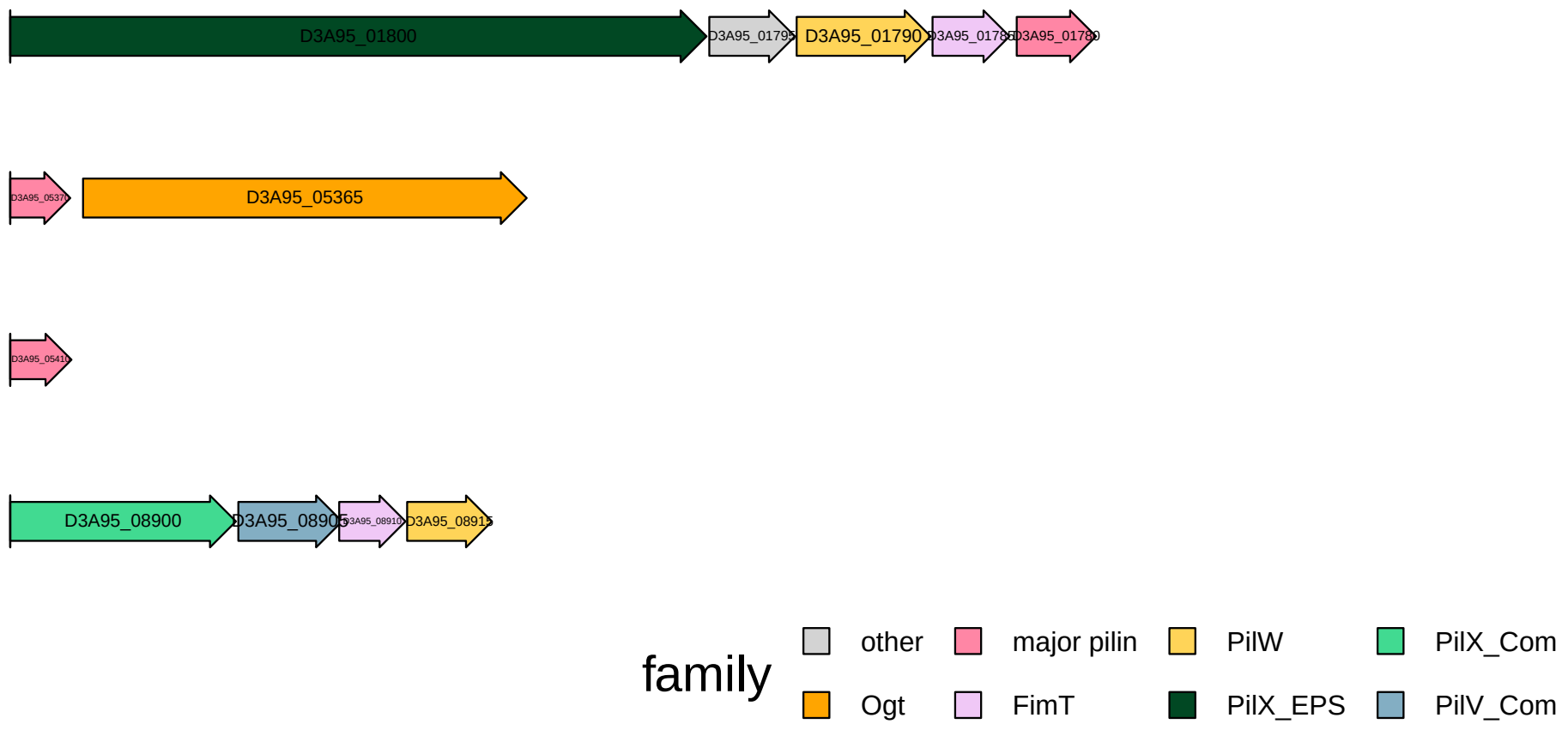

locus	gene	family	start	end
1385	D3A95_01780	major pilin	353949	354554
1385	D3A95_01785	FimT	354609	355196
1385	D3A95_01790	PilW	355210	356232
1385	D3A95_01795	other	356245	356898
1385	D3A95_01800	PilX_EPS	356919	362228
1390	D3A95_05365	Ogt	1093850	1097233
1390	D3A95_05370	major pilin	1097330	1097788
1392	D3A95_05410	major pilin	1101846	1102313
1398	D3A95_08900	PilX_Com	1797553	1799274
1398	D3A95_08905	PilV_Com	1799292	1800083
1398	D3A95_08910	FimT	1800061	1800567
1398	D3A95_08915	PilW	1800579	1801226

Thermosynechococcus vestitus (strain NIES−2133 / IAM M−273 / BP−1)

GCA_000011345.1

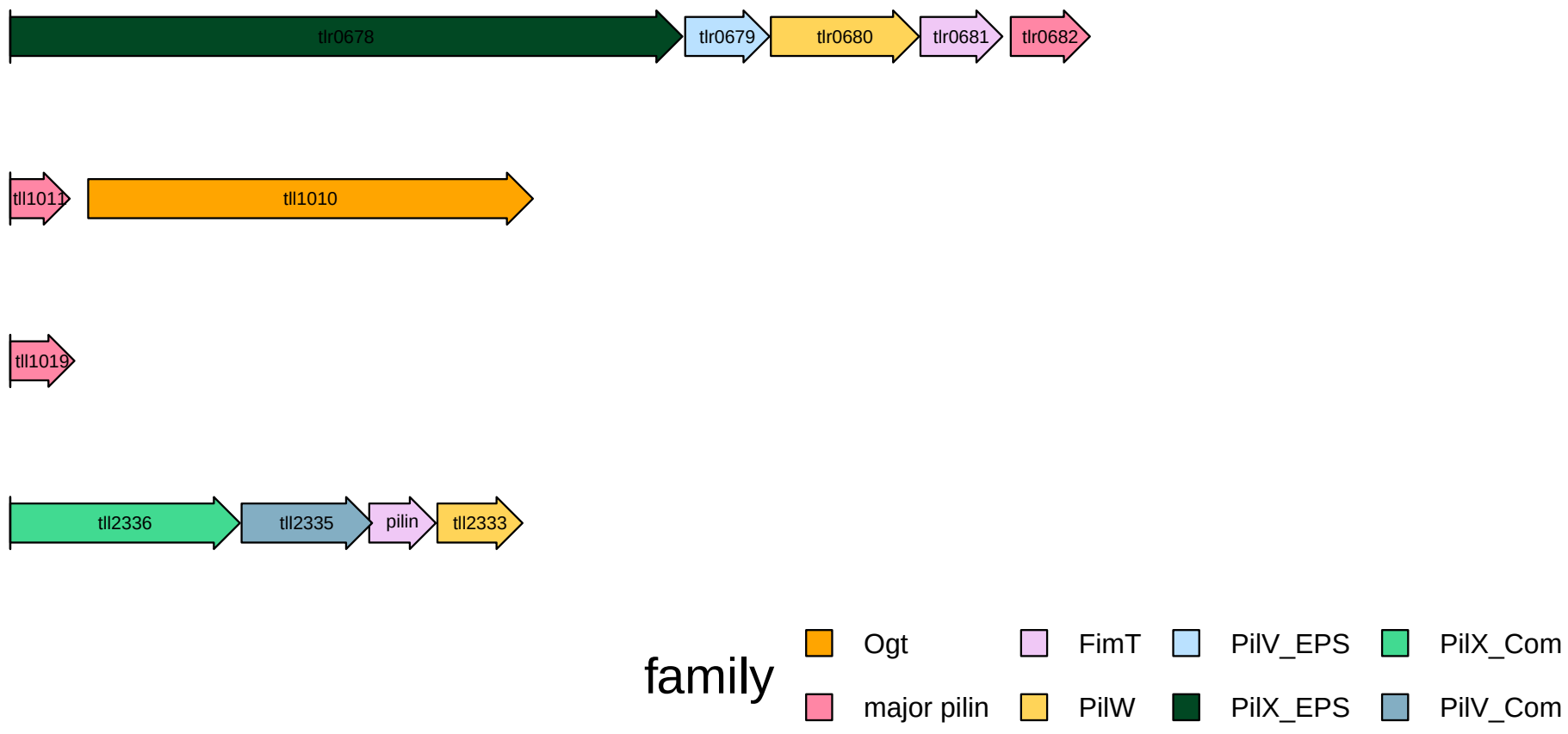

locus	gene	family	start	end
85	tlr0678	PilX_EPS	694822	699930
85	tlr0679	PilV_EPS	699951	700592
85	tlr0680	PilW	700602	701729
85	tlr0681	FimT	701739	702362
85	tlr0682	major pilin	702425	703027
87	tll1010	Ogt	1034604	1037984
87	tll1011	major pilin	1038124	1038576
88	tll1019	major pilin	1044505	1044993
96	tll2333	PilW	2428762	2429409
96	pilin	FimT	2429421	2429927
96	tll2335	PilV_Com	2429905	2430897
96	tll2336	PilX_Com	2430910	2432655

Tolypothrix bouteillei VB521301

GCA_000760695.2

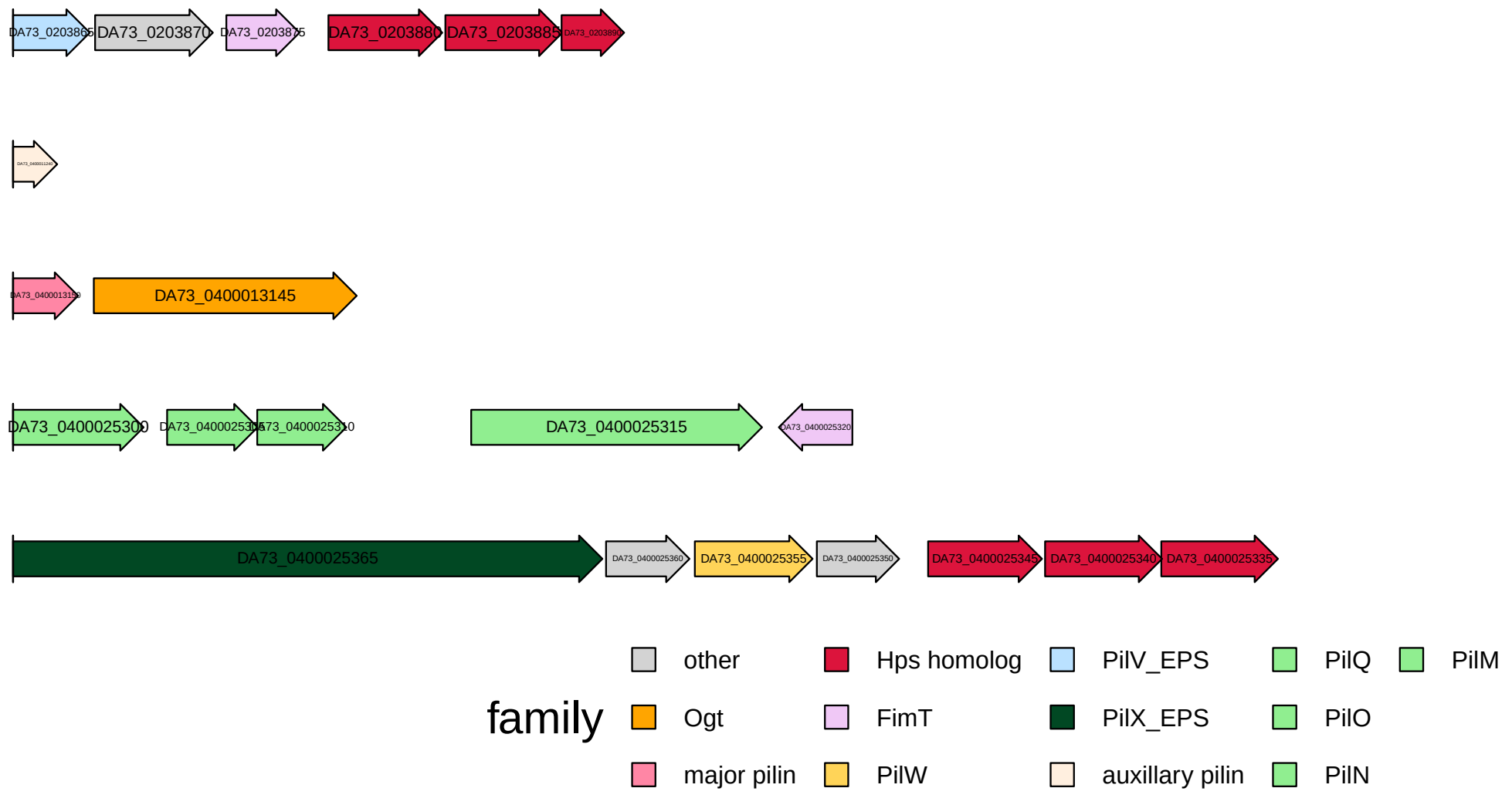

locus	gene	family	start	end
794	DA73_0203865	PilV_EPS	10251	10901
794	DA73_0203870	other	10946	11944
794	DA73_0203875	FimT	12058	12678
794	DA73_0203880	Hps homolog	12923	13888
794	DA73_0203885	Hps homolog	13914	14906
794	DA73_0203890	Hps homolog	14899	15429
803	DA73_0400011240	auxillary pilin	2823968	2824342
804	DA73_0400013145	Ogt	3302647	3304875
804	DA73_0400013150	major pilin	3305008	3305559
809	DA73_0400025300	PilM	6258701	6259807
809	DA73_0400025305	PilN	6260006	6260773
809	DA73_0400025310	PilO	6260770	6261525
809	DA73_0400025315	PilQ	6262583	6265048
809	DA73_0400025320	FimT	6265190	6265813
810	DA73_0400025335	Hps homolog	6266697	6267686
810	DA73_0400025340	Hps homolog	6267679	6268671
810	DA73_0400025345	Hps homolog	6268697	6269662
810	DA73_0400025350	other	6269907	6270605
810	DA73_0400025355	PilW	6270641	6271639
810	DA73_0400025360	other	6271684	6272391
810	DA73_0400025365	PilX_EPS	6272422	6277416

Tolypothrix tenuis PCC 7101

GCA_002368295.1

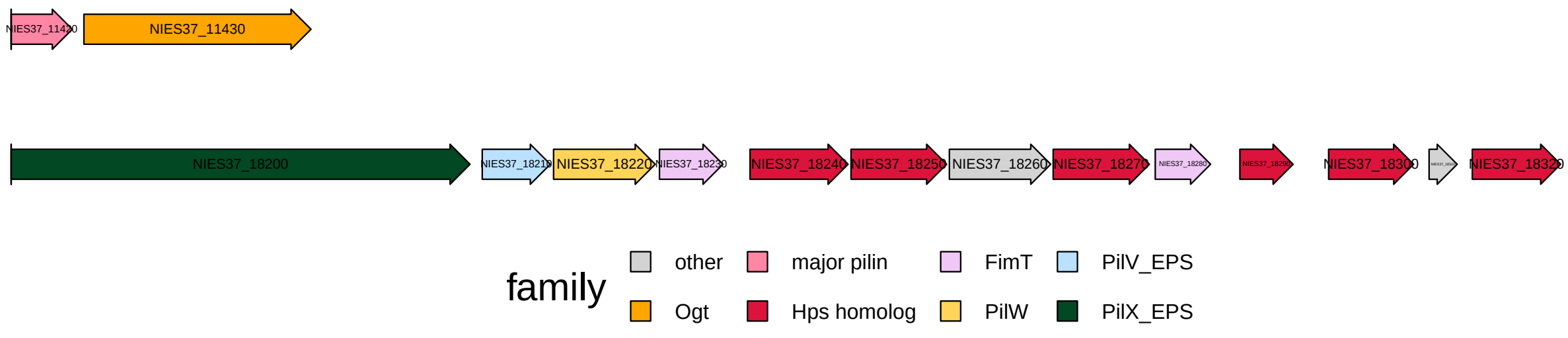

locus	gene	family	start	end
1098	NIES37_11420	major pilin	1414968	1415573
1098	NIES37_11430	Ogt	1415688	1417937
1101	NIES37_18200	PilX_EPS	2208821	2213365
1101	NIES37_18210	PilV_EPS	2213486	2214160
1101	NIES37_18220	PilW	2214192	2215199
1101	NIES37_18230	FimT	2215241	2215870
1101	NIES37_18240	Hps homolog	2216138	2217109
1101	NIES37_18250	Hps homolog	2217138	2218085
1101	NIES37_18260	other	2218112	2219116
1101	NIES37_18270	Hps homolog	2219140	2220093
1101	NIES37_18280	FimT	2220151	2220699
1101	NIES37_18290	Hps homolog	2220989	2221516
1101	NIES37_18300	Hps homolog	2221868	2222710
1101	NIES37_18310	other	2222863	2223141
1101	NIES37_18320	Hps homolog	2223291	2224166

Trichodesmium erythraeum (strain IMS101)

GCA_000014265.1

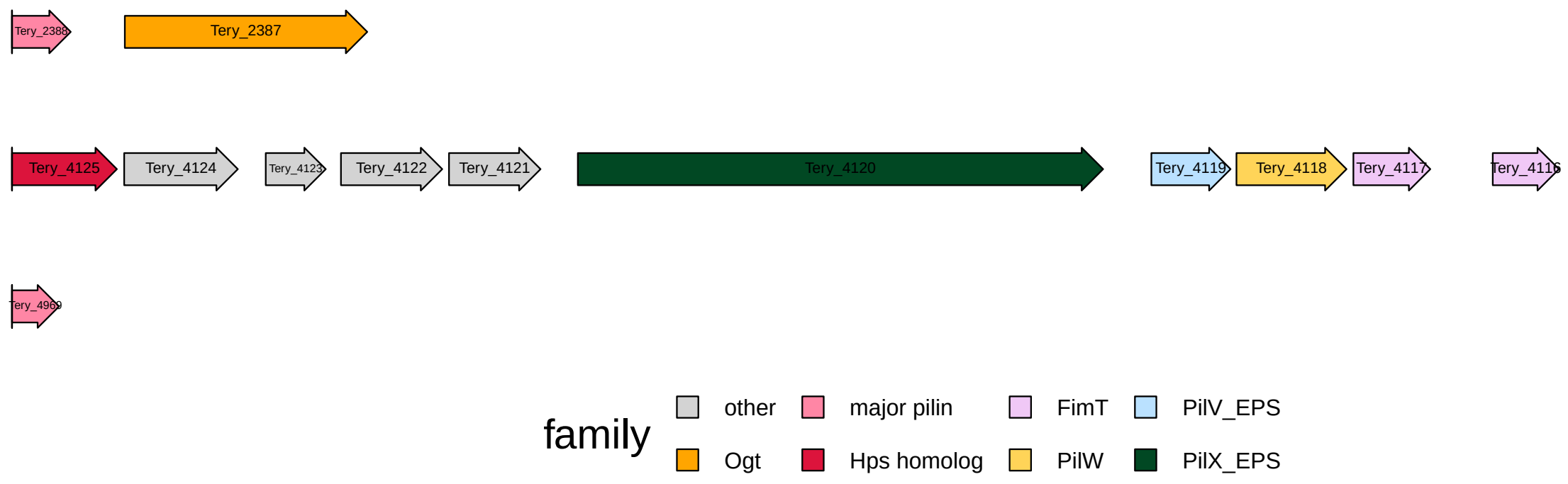

locus	gene	family	start	end
141	Tery_2387	Ogt	3689328	3691565
141	Tery_2388	major pilin	3692064	3692606
147	Tery_4116	FimT	6350462	6351073
147	Tery_4117	FimT	6351649	6352359
147	Tery_4118	PilW	6352422	6353438
147	Tery_4119	PilV_EPS	6353492	6354223
147	Tery_4120	PilX_EPS	6354668	6359515
147	Tery_4121	other	6359859	6360704
147	Tery_4122	other	6360766	6361701
147	Tery_4123	other	6361841	6362395
147	Tery_4124	other	6362652	6363701
147	Tery_4125	Hps homolog	6363768	6364736
150	Tery_4969	major pilin	7580253	7580687

Tychonema bourrellyi FEM_GT703

GCA_002412335.2

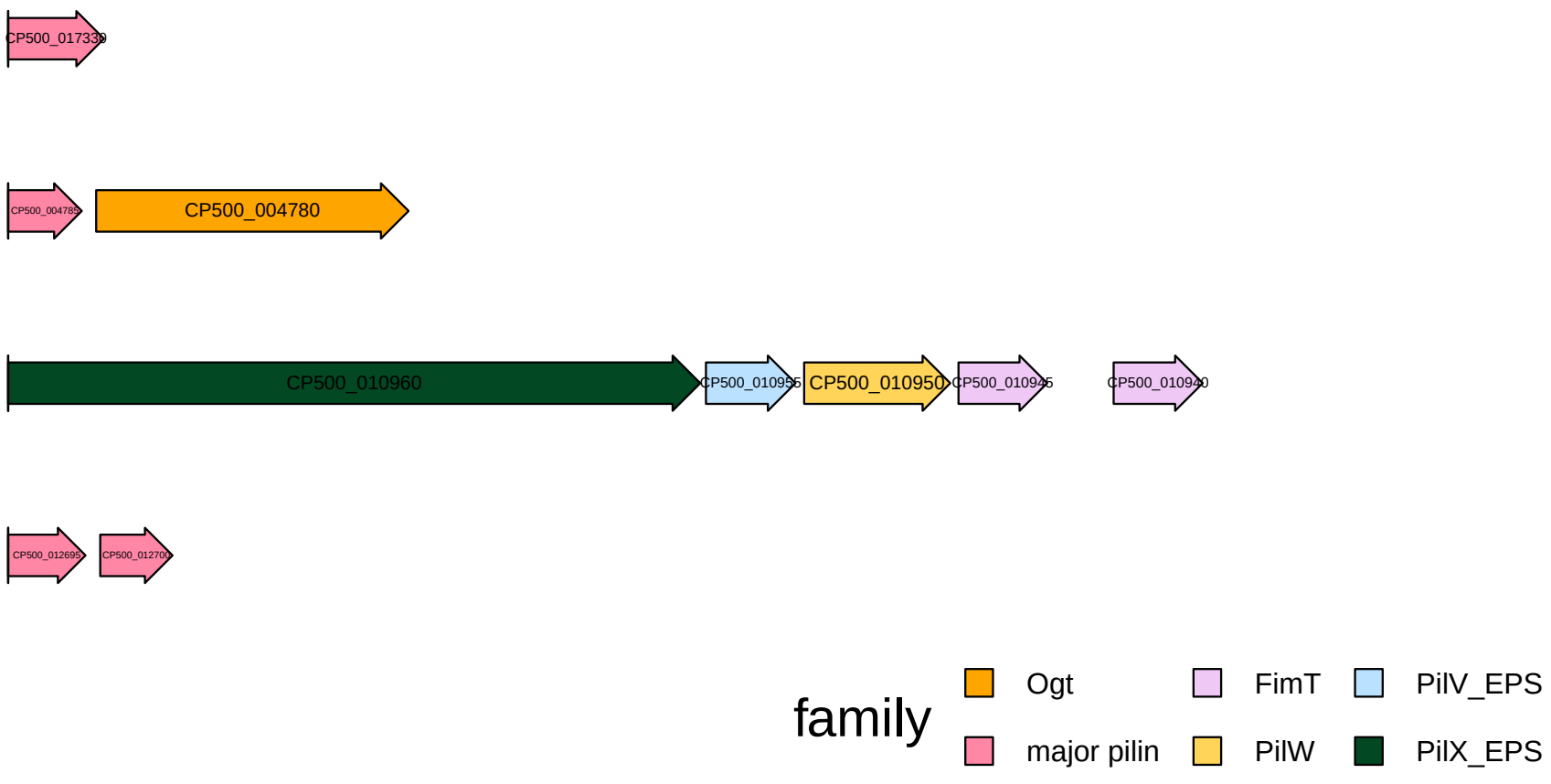

locus	gene	family	start	end
1111	CP500_017330	major pilin	12097	12783
1115	CP500_004780	Ogt	39273	41510
1115	CP500_004785	major pilin	41613	42140
1123	CP500_010940	FimT	9779	10417
1123	CP500_010945	FimT	10893	11528
1123	CP500_010950	PilW	11587	12633
1123	CP500_010955	PilV_EPS	12695	13336
1123	CP500_010960	PilX_EPS	13377	18332
1124	CP500_012695	major pilin	14780	15334
1124	CP500_012700	major pilin	15439	15957
