# Supplementary material for: Distinct core minor pilin complexes prime specialized type IV filaments in cyanobacteria
Source: PLoS Genet. 2026 Oct 26;22(10):e1012337. doi: 10.1371/journal.pgen.1012337 (PMC13759059; doi:10.1371/journal.pgen.1012337)
Supplement: Supplementary file 3 [file pgen.1012337.s025.pdf]

Table S1: Strains used in this study

Strain	Parent	Relevant Genotype	Reference/Plasmid
**Wild types & mutant strains**			
Synechocystis PCC-M	N/A	Wild type	(1)
Δ*pilA5-6*	WT	TU2300::Cm	(2)
Δ*pilA9-12*	WT	TU763::Km	(2)
Thermosynechococcus vulcanus NIES-2134	N/A	Wild type	(3)
***Synechocystis* strains created for this study**			
Δ*pilA1*	WT	*pilA1*::Cm^R	pGEM-Teasy-ΔpilA1-CamR
Δ*pilA2*	WT	*pilA2*::Km^R	pNS112
Δ*pilA4*	WT	*pilA4*::Km^R	pNS061
Δ*pilA7-8*	WT	*pilA7-8*::Km^R	pNS062
Δ*pilA7-8/pilA7*$^+$	Δ*pilA7-8*	*pilA7-8*::Km^R / [P*rnsB*::*pilA7*, Sm^R]	pNS142
Δ*pilA7-8/pilA8*$^+$	Δ*pilA7-8*	*pilA7-8*::Km^R / [P*petJ*::*pilA8*, Sm^R]	pNS132
Δ*pilA7-8/pilA7-8*$^+$	Δ*pilA7-8*	*pilA7-8*::Km^R / [P*petJ*::*pilA7-8*, Sm^R]	pNS131
Δ*pilA9-12/pilA5-6*	Δ*pilA9-12*	TU763::Km^R, TU2300::Cm^R	pJET-ΔTU2300-CmR-rev
Δ*pilA9-12/pilA7-8*	Δ*pilA9-12*	TU763::Km^R, *pilA7-8*::Em^R	pNS133
pilA1-Km^R	WT	*pilA1*:Km^R	pUC-*pilA1*-KmR
pilA1(T77C)	WT	*pilA1*(T77C)-Km^R	pNS074
pilA1(T80C)	WT	*pilA1*(T80C)-Km^R	pNS075
pilA1(T105C)	WT	*pilA1*(T105C)-Km^R	pNS077
pilA1(T134C)	WT	*pilA1*(T134C)-Km^R	pNS087
pilA1(A146C)	WT	*pilA1*(A146C)-Km^R	pNS076
pilA1(T151C)	WT	*pilA1*(T151C)-Km^R	pNS078
WT^{T77C}	WT	*pilA1*(T77C)-Sm^R	pNS121
Δ*pilA5-6*^{T77C}	WT^{T77C}	TU2300::Cm^R, *pilA1*(T77C)-Sm^R	pJET-ΔTU2300-CmR-rev
Δ*pilA7-8*^{T77C}	WT^{T77C}	Δ*pilA7-8*::Km^R, *pilA1*(T77C)-Sm^R	pNS133
Δ*pilA9-12*^{T77C}	Δ*pilA9-12*	TU763::Km^R, *pilA1*(T77C)-Sm^R	pNS121
Δ*pilX1*^{T77C}	WT^{T77C}	*slr0226*::Gm^R, *pilA1*(T77C)-Sm^R	pNS149
Δ*pilX2*^{T77C}	WT^{T77C}	*slr0442*::Km^R, *pilA1*(T77C)-Sm^R	pJET-Δslr0442
Δ*pilX3*^{T77C}	WT^{T77C}	*sll1268*::Em^R, *pilA1*(T77C)-Sm^R	pNS150
***Thermosynechococcus* strains created for this study**			
Δ*tll1019*	WT	Δ*tll1019::Km*^{R}	pUC19-Δtll1019_Kmv2
Δ*tlr0680-82*	WT	Δ*tlr0680-82::Km*^{R}	pUC19-Δtlr0680-82_Kmv2
Δ*tll2333-35*	WT	Δ*tll2333-35::Km*^{R}	pUC19-Δtll2333-35_Kmv2

References

1. D. Trautmann, *et al.*, Microevolution in Cyanobacteria: Re-sequencing a Motile Substrain of Synechocystis sp. PCC 6803. *DNA Res.* **19**, 435–448 (2012).

2. T. Wallner, L. Pedroza, K. Voigt, V. Kaever, A. Wilde, The cyanobacterial phytochrome 2 regulates the expression of motility-related genes through the second messenger cyclic di-GMP. *Photochem. Photobiol. Sci. Off. J. Eur. Photochem. Assoc. Eur. Soc. Photobiol.* **19**, 631–643 (2020).

3. D. Nakane, *et al.*, Thermosynechococcus switches the direction of phototaxis by a c-di-GMP-dependent process with high spatial resolution. *eLife* **11** (2022).
