# Supplementary material for: Distinct core minor pilin complexes prime specialized type IV filaments in cyanobacteria
Source: PLoS Genet. 2026 Oct 26;22(10):e1012337. doi: 10.1371/journal.pgen.1012337 (PMC13759059; doi:10.1371/journal.pgen.1012337)
Supplement: Supplementary file 4 [file pgen.1012337.s026.pdf]

Table S2: Plasmids used in this study

Plasmid	Description / Function	Source / Reference
**Plasmids (this study)**		
pNS05	RSF1010-Based conjugative expression vector with P*petJ*	This study
pNS141	pNS05 derivative with P*rnsB* promoter	This study
pNS142	P*rnsB*-driven expression of *pilA7*	This study
pNS131	P*petJ*-driven expression of *pilA7-pilA8*	This study
pNS132	P*petJ*-driven expression of *pilA8*	This study
pNS062	Knockout construct for *pilA7-pilA8* (*pilA7-8*::Km^R)	This study
pNS133	Knockout construct for *pilA7-pilA8* (*pilA7-8*::Em^R)	This study
pNS112	Knockout construct for *pilA2* (*pilA2*::Km^R)	This study
pNS061	Knockout construct for *pilA4* (*pilA4*::Km^R)	This study
pUC-pilA1-KmR	Construct for Km^R integration between *pilA1* and *pilA2*	This study
pNS074	pUC-pilA1-KmR derivative with *pilA1(T77C)*	This study
pNS075	PUC-pilA1-KmR derivative with *pilA1(T80C)*	This study
pNS077	PUC-pilA1-KmR derivative with *pilA1(T105C)*	This study
pNS087	PUC-pilA1-KmR derivative with *pilA1(T134C)*	This study
pNS076	PUC-pilA1-KmR derivative with *pilA1(A146C)*	This study
pNS078	PUC-pilA1-KmR derivative with *pilA1(T151C)*	This study
pNS121	pNS074 derivative where Km^R is replaced by Sm^R	This study
pNS149	pJET-Δ*slr0226* derivative where Gm^R replaces Km^R	This study
pNS150	pJET-Δ*sll1268* derivative where Km^R is replaced by Em^R	This study
pUC19-Δ*tll1019*_Kmv2	Knockout construct for NIES2134_108990	This study
pUC19-Δ*tlr0680*-82_Kmv2	Knockout construct for NIES2134-105520	This study
pUC19-Δ*tll2333*-35_Kmv2	Knockout construct for NIES2134-105520	This study
**Source plasmids**		
pUR	Source of streptomycin resistance (Sm^R) cassette	(1)
pUC-Δ*pixGH*	Source of gentamycin resistance (Gm^R) cassette	(2)
pUC4K	Source of kanamycin resistance (Km^R) cassette	Amersham
pUC19	Amp^R backbone for cloning knockout constructs	(3)
pGEM-Teasy-ΔpilA1-CamR	Construct for *pilA1* knockout (*pilA1*::Cm^R)	(4)
pJET-Δ*slr0226*	Knockout construct for *slr0226* (*slr0226*::Km^R)	(5)
pJET-Δ*sll1268*	Knockout construct for *sll1268* (*sll1268*::Km^R)	(5)
pJET-Δ*slr0442*	Knockout construct for *slr0442* (*slr0442*::Km^R)	(5)
pJET-ΔTU2300-CmR-rev	Knockout construct for TU2300 (*pilA5-6*::Cm^R)	(6)

References

1. A. Wiegard, A.K. Dörrich, H.T. Deinzer, C. Beck, A. Wilde, J. Holtzendorff, and I.M. Axmann, Biochemical analysis of three putative KaiC clock proteins from *Synechocystis* sp. PCC 6803 suggests their functional divergence. *Microbiology* **159**: 948–958 (2013)

2. Y. Han, A. Jakob, S. Engel, A. Wilde, N. Schuergers, PATAN-domain regulators interact with the Type IV pilus motor to control phototactic orientation in the cyanobacterium Synechocystis. *Mol. Microbiol.* **117**, 790–801 (2022).

3. J. Norrander, T. Kempe, J. Messing, Construction of improved M13 vectors using oligodeoxynucleotide-directed mutagenesis. *Gene* **26**, 101–106 (1983).

4. F. D. Conradi, *et al.*, Factors Controlling Floc Formation and Structure in the Cyanobacterium Synechocystis sp. Strain PCC 6803. *J. Bacteriol.* **201** (2019).

5. S. Oeser, *et al.*, Minor pilins are involved in motility and natural competence in the cyanobacterium Synechocystis sp. PCC 6803. *Mol. Microbiol.* **116**, 743–765 (2021).

6. T. Wallner, L. Pedroza, K. Voigt, V. Kaever, A. Wilde, The cyanobacterial phytochrome 2 regulates the expression of motility-related genes through the second messenger cyclic di-GMP. *Photochem. Photobiol. Sci. Off. J. Eur. Photochem. Assoc. Eur. Soc. Photobiol.* **19**, 631–643 (2020).
