# Supplementary material for: Distinct core minor pilin complexes prime specialized type IV filaments in cyanobacteria
Source: PLoS Genet. 2026 Oct 26;22(10):e1012337. doi: 10.1371/journal.pgen.1012337 (PMC13759059; doi:10.1371/journal.pgen.1012337)
Supplement: Supplementary file 5 [file pgen.1012337.s027.pdf]

Table S1: Oligonucleotides used in this study

Primer Name	Sequence (5' to 3')	Template/Purpose
**pNS141 construction**		
NS310	TCATGATCTTTATAATCCATACCACCTCAAATTGGGAATT	*rnsB* promoter
NS311	CGGTCAAGGTTCTGGACCAGTTCCACCAGCAAAATTCGCA	*rnsB* promoter
NS312	AATTCCCAATTTGAGGTGGTATGGATTATAAAGATCATGATGGCG	pNS05
NS313	TGCGAAATTTTGCTGGTGGAACTGGTCCAGAACCTTGACCG	pNS05
**pNS142 construction**		
NS314	AATTCCCAATTTGAGGTGGTATGGCTTACAAGAATGAACTG	*pilA7*
NS315	GGCAACCGAGCGTTGGATCCCTAATAACCAGTTCTGCATTGC	*pilA7*
NS316	AATGCAGAACTGGTTATTAGGGATCCAACGCTCGGTTGCC	pNS142
NS317	AGTTCATTCTTGTAAGCCATACCACCTCAAATTGGGAATTTGTC	pNS142
**pNS131 construction**		
NS291	AGTTCATTCTTGTAAGCCATATGTTCTCCTTCAAGGATAAAG	pNS05
NS292	GTACAATTAATGCAGTCTTTAATAAGGATCCAACGCTCG	pNS05
NS293	CGAGCGTTGGATCCTTATTAAAGACTGCATTAATTGTACAAC	*pilA7-pilA8*
NS294	TATCCTTGAAAGGAGAACATATGGCTTACAAGAATGAACTG	*pilA7-pilA8*
**pNS132 construction**		
NS295	GTACAATTAATGCAGTCTTTAATAAGGATCCAACGCTCG	pNS05
NS296	AAGTACCATAGAATTTTCACATGTTCTCCTTCAAGGATAAAG	pNS05
NS297	TATCCTTGAAAGGAGAACATGTGAAAATTCTATGGTTACTAATGAC	*pilA8*
NS298	CGAGCGTTGGATCCTTATTAAAGACTGCATTAATTGTACAAC	*pilA8*
**pNS062 construction**		
NS050	GTGCGGGCCTCTTCGCTATTTTTGAGCAGGTTGAAAAACA	*pilA8* flank
NS051	AGATTTTGAGACACAACGTGAGCAAAAATGGTAGCTTAAAATTAT	*pilA8* flank
NS052	TTTAAGCTACCATTTTTGCTCACGTTGTGTCTCAAAATCT	Km^R cassette
NS053	CTTGAACAAGACTTTGCATTGATCCTTCAACTCAGCAAAA	Km^R cassette
NS054	TTTTGCTGAGTTGAAGGATCAATGCAAAGTCTTGTTCAAGATC	*pilA7* flank
NS055	GAGTTAGCTCACTCATTAGGAGTTCGGGCATTAGCTGCAA	*pilA7* flank
NS056	TTGCAGCTAATGCCCGAACTCCTAATGAGTGAGCTAACTCACATT	pUC19 backbone
NS057	TGTTTTTCAACCTGCTCAAAAATAGCGAAGAGGCCCGCAC	pUC19 backbone
**pNS133 construction**		
NS299	AATTAGTATAATTATAGCACGAGCAAAAATGGTAGCTTAAAATTATCT	pNS062
NS300	GTTAAGGGATGCAGGTCGACAATGCAAAGTCTTGTTCAAGA	pNS062
NS301	CTTGAACAAGACTTTGCATTGTCGACGTGCATCCCTTAAC	Em^R cassette
NS302	TTTAAGCTACCATTTTTGCTCGTGCTATAATTATACTAATTTTATAAGGA	Em^R cassette
**pNS112 construction**		
NS174	AAAAAACGACGTAGATCCATCACCGAAACGCGCGAGACGA	pUC19 backbone
NS175	GCCCCAATAAACATATTCTTCACTGACTCGCTGCGCTCGG	pUC19 backbone
NS176	TCGTCTCGCGCGTTTCGGTGATGGATCTACGTCGTTTTTTC	*pilA2* flank
NS177	AGATTTTGAGACACAACGTGAACAATAGTGTGAAAATATTAACCCA	*pilA2* flank
NS178	AATATTTTCACACTATTGTTCACGTTGTGTCTCAAAATCT	Km^R cassette
NS179	AATTCAGCTTAAATACCATATTAGAAAAACTCATCGAGCATC	Km^R cassette
NS180	TGCTCGATGAGTTTTTCTAATATGGTATTTAAGCTGAATTTACACG	*pilA2* flank
NS181	CCGAGCGCAGCGAGTCAGTGAAGAATATGTTTATTGGGGCAC	*pilA2* flank
**pNS061 construction**		
NS042	GTGCGGGCCTCTTCGCTATTAAGTTGCCGTGGTGAATAGT	*pilA4* flank
NS043	AGATTTTGAGACACAACGTGCTCGGTCTTCATAGCTGTGC	*pilA4* flank
NS044	GCACAGCTATGAAGACCGAGCACGTTGTGTCTCAAAATCT	Km^R cassette
NS045	AGTAATCCATCCCTAGAGCCGATCCTTCAACTCAGCAAAA	Km^R cassette
NS046	TTTTGCTGAGTTGAAGGATCGGCTCTAGGGATGGATTACT	*pilA4* flank
NS047	GAGTTAGCTCACTCATTAGGTAAACATTTAACTATTCGACAAAATATTG	*pilA4* flank
NS048	GTCGAATAGTTAAATGTTTACCTAATGAGTGAGCTAACTCACATT	pUC19 backbone
NS049	ACTATTCACCACGGCAACTTAATAGCGAAGAGGCCCGCAC	pUC19 backbone
**pUC-*pilA1*-KmR construction**		
pUC-HR1-fw2	AAAACGACGGCCAGTGAATTCTGAGTTCCATGTGC	*pilA1* flank
pUC-HR1-rv2	AAAACGACGGCCAGTGAATTCTGAGTTCCATGTG	pUC19 backbone
HR1-Km-fw2	AGTAATTAAATAGGACGGGAAAGCCACGTTGTG	Km^R cassette
HR1-Km-rv2	ACAACGTGGCTTTCCCGTCCTATTTAATTACTTCAGC	*pilA1* flank
Km-HR2-fw2	TCACGAGGCAGACCTCCCTATTATGTTTTGAGTG	*pilA2* flank
Km-HR2-rv2	CTCAAAACATAATAGGGAGGTCTGCCTCGTGAAG	Km^R cassette

HR2-pUC-fw2	TTAGTATCCGAGAAAGGATCCTCTAGAGTCGACC	pUC19 backbone
HR2-pUC-rv2	GGTCGACTCTAGAGGATCCTTTCTCGGATACTAA	*pilA2* flank
**Site-directed mutagenesis**		
NS084	TGAGAAGGGTTGTTTTGCAACCGATACG	*pilA1(T77C)*
NS085	GTAAAATAACCTTGTTGG	*pilA1(T77C)*
NS086	ACGTTTGCATGTGATACGGAAACGCTTG	*pilA1(T80C)*
NS087	ACCCTTCTCAGTAAAATAACC	*pilA1(T80C)*
NS088	GCTGACAACTGTGAAGCTATCCAAGAC	*pilA1(T105C)*
NS089	AGTGTTAACTGCAAAAC	*pilA1(T105C)*
NS090	CGATTCAGGCTGTCGCGCCTTCAGTAC	*pilA1(T134C)*
NS091	TAAAACGTTCCACCTGAC	*pilA1(T134C)*
NS092	TCGGGCTGAATGTGGTAGTGAAGATACTC	*pilA1(A146C)*
NS093	CAAACAACAGTACTGAAGG	*pilA1(A146C)*
NS094	TAGTGAAGATTGTCCCCCAACCCCAGG	*pilA1(T151C)*
NS095	CCTGCTTCAGCCCGACAAAC	*pilA1(T151C)*
**pNS121 construction**		
NS210	CGGTCAAGGTTCTGGACCAGGTCCTATTTAATTACTTCAGCACC	pUC-*pilA1*-KmR
NS211	TTTTGGCGCGCCGCTAGCATCCTATTATGTTTTGAGTGGTGC	pUC-*pilA1*-KmR
NS212	ACCACTCAAAACATAATAGGATGCTAGCGGCGCGCAAAA	Sm^R cassette
NS213	CTGAAGTAATTAAATAGGACCTGGICCAGAACCTTGACCGA	Sm^R cassette
**pNS149 construction**		
NS334	CCAATTCGAGCTCGGTACCCTTAGTTGTTTTTCTAGAAGTTTTCTTGCTACC	pJET-Δ*slr0226*
NS335	TCAATTCGAGCTCGGTACCCAGGGAGCGACTTCAGCCCAC	pJET-Δ*slr0226*
NS336	GTGGGCTGAAGTCGCTCCCTGGGTACCGAGCTCGAATTGA	Gm^R cassette
NS337	ACTTCTAGAAAAACAACTAAGGGTACCGAGCTCGAATTGG	Gm^R cassette
**pNS150 construction**		
VR5	ATTTCTTTTTTCGTCGACTTAAAAGCAACCAGCAGG	pJET-Δ*sll1268*
VR6	CCTGCTGGTTGCTTTTAAGTCGACGAAAAAAGAAAT	Em^R cassette
VR7	AAGGATAAAACTGAACTTACTTACTTATTAAATAAT	Em^R cassette
VR8	ATTATTTAATAAGTAAGTAAGTTCAGTTTTATCCTT	pJET-Δ*sll1268*

**pUC19-Δtvtll1019_Kmv2 construction**	
pUC19-45R_tvtll1019	AATTTGGGCAATGGGATACCGAGCTCGAATTCAC
pUC19-46F_tvtll1019	TGCAAACGCTGTGAAATGCAAGCTTGGCGTAATC
tvtll1019-1F_pUC19	GAATTCGAGCTCGGTATCCCATTGCCCAAATTCG
tvtll1019-2R_Kmv2	CCTGAGTGCTTGCGGCTCGATAGAACCAAGGCAG
Km-23F_tvtll1019	GCCTTGGTTCTATCGAGCCGCAAGCACTCAGG
Km-24R_tvtll1019	TATGTACGTGGCGTAGCCTTTCATAGAAGGCGGC
tvtll1019-3F_Kmv2	CGCCTTCTATGAAAGGCTACGCCACGTACATAAC
tvtll1019-4R_pUC19	TTACGCCAAGCTTGCATTTCACAGCGTTTGCAGC
**pUC19-Δtvtlr0680-82_Kmv2 construction**	
pUC19-47R_tvtlr0680	AGTCACACCATCAAAGTACCGAGCTCGAATTCAC
pUC19-48F_tvtlr0680	TCCTTGAGAAAGGTGGTGCAAGCTTGGCGTAATC
tvtlr0680-1F_pUC19	GAATTCGAGCTCGGTACTTTGATGGTGTGACTCC
tvtlr0680-2R_Kmv2	CCTGAGTGCTTGCGGCTCTCCGTCAAGGTAAAGC
Km-25F_tvtlr0680	TTTACCTTGACGGAGAGCCGCAAGCACTCAGG
Km-26R_tvtlr0680	AGATACTCCAGTGGTTCCTTTCATAGAAGGCGGC
tvtlr0680-3F_Kmv2	CGCCTTCTATGAAAGGAACCACTGGAGTATCTGC
tvtlr0680-4R_pUC19	TTACGCCAAGCTTGCACCACCTTTCTCAAGGATC
**pUC19-Δtvtll2333-35_Kmv2 construction**	
pUC19-49R_tvtll2333	AGGGCATTGTGTTTGTTACCGAGCTCGAATTCAC
pUC19-50F_tvtll2333	AGAACTGGAACGCTATTGCAAGCTTGGCGTAATC
tvtll2333-1F_pUC19	GAATTCGAGCTCGGTAACAAACACAATGCCCTCC
tvtll2333-2R_Kmv2	CCTGAGTGCTTGCGGCGGGCTTGTATCCCATTTC
Km-27F_tvtll2333	AATGGGATACAAGCCCGCCGCAAGCACTCAGG
Km-28R_tvtll2333	AAACCACACCCGTTGACCTTTCATAGAAGGCGGC
tvtll2333-3F_Kmv2	CGCCTTCTATGAAAGGTCAACGGGTGTGGTTTAC
tvtll2333-4R_pUC19	TTACGCCAAGCTTGCAATAGCGTTCCAGTTCTCC
