# Supplementary material for: Distinct core minor pilin complexes prime specialized type IV filaments in cyanobacteria
Source: PLoS Genet. 2026 Oct 26;22(10):e1012337. doi: 10.1371/journal.pgen.1012337 (PMC13759059; doi:10.1371/journal.pgen.1012337)
Supplement: Supplementary file 6 [file pgen.1012337.s029.pdf]

We highly appreciate the very helpful comments on our manuscript and wish to thank the reviewers for carefully reading our manuscript. Please find the comments from the reviewers in bold followed by our answers and details on the changes to the manuscript.

Reviewer Comments to the Authors:

Reviewer #1: Distinct core minor pilin complexes prime specialized type IV filaments in cyanobacteria
Nils Schuergers, Lars Wittig, Gen Enomoto, Julia Herz, Shamphavi Sivabalasarma, Katharina Ruoff, Friedel Drepper, Sonja-Verena Albers, Pitter F. Huesgen, Annegret Wilde

Brief summary:
The genome of the model cyanobacterium Synechocystis sp. PCC 6803 encodes a diverse repertoire of Type IV pilus (T4P) components, including one major pilin, PilA1, and at least 13 additional putative minor pilins — highlighting the complexity of its pilus machinery. Through systematic genetic analysis, Schuergers et al. reveal that deletion of pilA7-A8 specifically impairs natural competence (DNA uptake) without affecting T4P formation, phototactic motility, or aggregation. In contrast, deletion of pilA4 has no such effect, underscoring the distinct roles of individual minor pilins. Building on this, the authors apply structural phylogenomics across 90 cyanobacterial genomes, uncovering six conserved pilin families: the major pilin PilA, and four minor pilin families — FimT, PilW, PilV, and PilX — plus a novel auxiliary pilin family. Within PilV and PilW, subgroups are identified, reflecting functional diversification. Based on structural and genomic context, two new subfamilies are proposed: PilXEPS (for PilX subunits associated with EPS motility pathways), and PilVEPS (co-occurring with PilXEPS) and PilVCom/PilXCom (linked to DNA uptake).
Genome-wide analysis reveals that many cyanobacteria possess multiple, co-occurring sets of core minor pilin genes (FimT/U–PilV–PilW–PilX), including both Com-type (competence-associated) and EPS-type (motility-associated) variants. This prediction is experimentally validated in Thermosynechococcus vulcanus, where deletion of specific pilin genes confirms their roles in motility and DNA uptake. A key finding is that individual deletion of either the Com or EPS pilin set in Synechocystis does not disrupt T4P formation, but loss of both sets abolishes pilus assembly entirely — demonstrating functional redundancy and the requirement of at least one pilin set for pilus biogenesis. Using maleimide labeling, the authors visualize morphologically distinct filaments: short, dynamic filaments in strains with only the Com set, and longer, adhesive filaments in those with only the EPS set.
Finally, quantitative proteomics of sheared pilus fractions reveals differential roles among the three PilX3 paralogs, with PilX2 playing a critical role in the accumulation of the PilA9–PilA12 pilin complex — linking specific minor pilins to structural and/or functional specialization.

The key discovery of that paper is that Synechocystis sp. PCC 6803 and probably several other cyanobacteria build two distinct Type IV pilus filaments using a shared core machinery — but with at least two different sets of minor pilins. The paper is filling some important knowledge gaps about the complexity of cyanobacterial T4P systems and is of broad interest for readers interested in the diverse functions of bacterial T4P systems. The paper presents a variety of highly interesting data and is well organized and clearly written. To further strengthen the manuscript, refinements would enhance clarity and consistency: A standardized and consistent naming scheme in text and figures and datasets for major, minor, and auxiliary pilins would improve readability. More detailed descriptions of certain experimental approaches and interpretations would help readers fully appreciate the conclusions.

Major comments:

Line 28: The sentence is misleading. It is the same T4P machine, which is capable to assemble two different type of filaments as written in lines 37 and 38.

We agree that the term 'two specialized nanomachines' could be misinterpreted and now emphasize that while the motor is common to both, the architectural decoupling of the filaments allows the cell to satisfy the conflicting requirements of DNA uptake and surface motility.

Revised Manuscript Sentence (Lines 28-21):

"These findings challenge the concept of a single multipurpose pilus, establishing that cyanobacteria utilize a shared machinery to assemble two specialized filaments, each primed by distinct core minor pilin complexes to meet the conflicting biophysical requirements of DNA uptake and surface motility."

Line 40: Tone down the sticky.

We refined the wording to better describe their longer dwelling times and function in facilitating cell movement.

The revised sentence (Line 41) now reads:

"...long fibers specialized for surface attachment..."

Lines 94-95: Please very briefly summarize the findings for PilA1, PilA2, PilA5-6, PilA9-12, PilX1, PilX2, and PilX3 in regard to T4P formation, phototactic motility, aggregation and DNA uptake as a supplementary figure to ease integration of the new presented data. Since the paper is filling some important knowledge gaps, it would also be suitable to provide a table at the end of the manuscript summarizing involvement of all 14 major, minor and auxiliary pilins in T4P formation, phototactic motility, DNA uptake and aggregation.

Lines 147-148: For clarification you must provide for Synechocystis an additional supplementary figure showing the depicted 14 major, minor and auxiliary pilin encoding genes as in figure 2C, but in which you also provide for every protein the locus tags of the genes (slr...), the trivial of the corresponding gene products (pilA5 .....), and the family type (as in dataset S2). Also provide the length of the genes (instead of start and stop) and size of the corresponding gene products in aa. Just an example PilA1 is mentioned all over in the manuscript, neither Figure 2C nor the dataset S2 are indicating which gene is pilA1. I assume it is one of the hofGs, for which no slr numbers are given.

We agree that navigating the pilin repertoire of *Synechocystis* can be challenging. According to the excellent idea of the reviewer, we have prepared a comprehensive summary table that integrates all the requested information into a single, easy-to-reference format. This new table details all 14 major, minor, and auxiliary pilins, providing their trivial names, locus tags, transcriptional units (TUs), family classifications, and protein sizes. Furthermore, it explicitly summarizes the phenotypic involvement of each pilin in T4P assembly, motility, cell aggregation, and DNA uptake. Furthermore, we have added Supplementary Figures S3 and S4 to illustrate the organization, locus tags, common names, structural family types and protein

structure predictions for all 14 *Synechocystis* pilin genes. To resolve any remaining ambiguities in the nomenclature, we have updated the supplementary datasets S1 and S2 to annotate the respective *hofG* loci by name and/or locus number.

The new summary table has been incorporated into the manuscript and is referenced multiple times in the updated text, especially in the introduction when *Synechocystis* pilins are mentioned for the time (Lines 82-83):

"A comprehensive summary of the complete Synechocystis pilin repertoire is provided in Table 1"

Figure S3 is mentioned explicitly (Lines 150-151):

"The genomic organization, structural classification, and predicted structures of the 14 Synechocystis pilins are detailed in Supplementary Figures S3 and S4."

Lines 154-156. Please describe the homology (protein sequence or structure) between this PilX subfamily and HpsA in more detail.

We agree that detailing the architectural divergence clarifies the functional specialization of these proteins. To directly address this, we now include the structural predictions for the complete *Synechocystis* pilin repertoire (now included as Figure S4) and performed comprehensive, structure-based alignments of the Com and EPS minor pilin subfamilies using FoldMason (now included as Figure S5). Our structural analysis reveals a profound architectural divergence between the two PilX subfamilies. While both retain the canonical pilin fold, the $PilX_{Com}$ variants are relatively compact (mean length ~468 amino acids) and are characterized by a structurally expanded αβ-loop region immediately following the α1N helix. In stark contrast, the $PilX_{EPS}$ subfamily is massively expanded (mean length ~1464 amino acids). This size increase is primarily driven by a highly extended β-solenoid domain interspersed with complex coiled regions, frequently featuring distinct, lineage-specific domains grafted onto the distal tip of the solenoid. These additional domains likely modulate functional fine-tuning and their presence could possibly allow phylogenetic subclassification. However, mapping these terminal subdomains across all cyanobacterial lineages falls beyond the scope of the current manuscript.

Interestingly, the established HpsA hidden Markov model includes both the expanded domains and the PilX core elements (the α1N-helix and β-sheets). Because it reliably detects only the $PilX_{EPS}$ subfamily, it clearly indicates that the structural differences between the Com and EPS core folds are also conserved at the primary sequence level.

The manuscript now includes these findings (Lines 169-177):

"However, structure-based alignments reveal an architectural division (Fig S5). One subfamily is relatively compact, featuring an expanded αβ-loop region. In contrast, the other subfamily exhibits structural expansion driven by a highly elongated β-solenoid and the frequent addition of lineage-specific domains grafted onto the distal end of the solenoid. Similarly, the PilV subfamilies diverge structurally; the PilV co-occurring with the expanded PilX features a significantly elongated, flexible coil region between strands β3 and β4 that is enriched in helix-breaking and polar residues.

Notably, the sequence profile of HpsA, a protein essential for motility and hormogonium polysaccharide (HPS) secretion in Nostoc punctiforme (43), reliably maps only to this enlarged PilX subfamily, demonstrating that the structural divergence is reflected at the sequence level."

Lines 159-161. Please give references for PilA5 and RntB.

We have added references for PilA5 (Oeser et al., 2021) and RntB (Taton et al., 2020) to correctly attribute the characterization of these proteins and their roles in natural competence.

Line 204-211: Why is the analysis of the Thermosynechococcus vulcanus genome not included in the Dataset S2? Which three genes are deleted in the tlr0680-82 mutant and the tll2333-35 mutant? Please clarify.

Our structural analysis utilized 90 NCBI RefSeq genomes, with *T. vestitus* BP-1 (NIES-2133) as the designated reference for the genus. Given the >99 protein identity, BP-1 locus tags serve as high-fidelity references for orthologs in *T. vulcanus* NIES-2134 wich we used for functional assays because the BP-1 laboratory strain is non-motile due to a known *pilC* frameshift mutation.

Revised Manuscript Text (Lines 229-236):

"...we constructed deletion mutants of genes encoding pilins in Thermosynechococcus vulcanus NIES-2134. This strain is highly similar to the NCBI reference strain T. vestitus BP-1 analyzed in Dataset S2, but was preferred for functional assays as it remains fully motile under laboratory conditions. [...] Targeting tlr0680-82 from the predicted EPS/motility-related locus (Figure S3) abolished phototactic movement, whereas inactivation of tll2333-35 from the predicted competence-related locus resulted in a complete loss of natural transformability..."

Lines 222-233: Could the authors try to model the two suggested core minor pilin set using Alphafold

We have now used AlphaFold3 to model the co-assembly of both the putative Com-type (PilA5/A7/A8/PilX1) and EPS-type (PilA9/A10/A11/A12) minor pilin complexes atop a base segment of six PilA1 subunits. These models have been added to the manuscript as the new Figure S7. The Com-type tip complex yielded a favorable interface predicted Template Modeling (ipTM) score of 0.74. While the EPS-type tip complex produced a more modest ipTM score of 0.54, the predicted interactions of the N-terminal α1-helices and the hierarchical assembly order of the subunits align perfectly with the proposed spatial arrangement of the core minor pilins in the priming complex. Therefore, we believe these structural predictions provide a plausible representation of how these distinct core minor pilin sets assemble into specialized pilus tips.

We now refer to the models in the manuscript (Lines 252-254):

"Furthermore, structural predictions of these putative tip complexes support the hypothesis that they can co-assemble atop a PilA1 filament in a hierarchical arrangement consistent with proposed priming complex models (Fig S7)."

Do the authors assume that the PilV PilW proteins of PilA5-A8 can alternatively associate with PilX1, PilX2 or PilX3? Did the authors tried to delete PilX1 and PilX3 together, or evne all three PilX proteins?

We agree that this is an important question. To determine which of the PilX$_{Com}$ paralogs PilX1, PilX2, or PilX3 caps the competence pilus we generated all possible double *pilX* mutants, as well as a triple *pilX1-3* mutant. Our transformation assays revealed that only the triple mutant lost natural competence, demonstrating that any single PilX paralog is sufficient to support DNA uptake, confirming the high degree of functional redundancy and alternative association you hypothesized. We have integrated these genetic findings into the revised proteomics section of the Results to functionally explain why none of the mutants lacking a single PilXcom paralog showed a stark depletion of other com-type pilins in the sheared pilus fraction.

The text now reads (Lines 330-338):

"Moreover, none of the mutants lacking a single PilX$_{Com}$ paralog showed a stark depletion of other Com-type pilins in the sheared pilus fraction. This precluded the assigning of PilX variants to defined complexes based solely on abundance in sheared fractions and suggests a high degree of functional plasticity. To test if PilX$_{Com}$ paralogs can substitute for one another, we evaluated natural competence in all double and triple pilX$_{Com}$ mutant combinations (Fig 4C). Strikingly, while all double mutants retained wild-type transformation efficiencies, the triple ΔpilX1-3 mutant was non-transformable. This demonstrates that any single PilX$_{Com}$ paralog is sufficient to cap the Com-type filament and prime assembly of competence pili."

What might be role of the two FimT proteins in PilA5-A8?

PilA6 and PilA7 share 42% sequence identity and a 0.84 TM-score, suggesting structural interchangeability within the pilus tip complex. Despite this conservation, PilA7 is essential for natural competence, whereas PilA6 is redundant. It remains unclear why these nearly identical proteins have such distinct requirements, as no specific structural features or conserved positions explain why PilA7 cannot be substituted by its homolog.

We now address this by adding (Lines 119-121):

"However, while PilA7 is strictly required for this process, PilA6 is entirely redundant. This functional divergence is unexpected given their 42% sequence identity, indicating that these putative paralogs cannot simply substitute for one another."

Lines 236-245: I highly appreciate the search for a functional PilA1 mutation suitable for maleimide labeling. However the chosen PilA1 T77C mutation seems to produce less pili as the WT, that should be stated.

Regarding the apparent reduction of pili in the PilA1(T77C) mutant shown in the initial screen, we agree that the western blot signal is weaker. However, this difference could also be attributed to altered protein stability, increased pilus fragility during the mechanical harvesting process, or reduced antibody affinity toward the mutated PilA1 variant. Nonetheless, we fully acknowledge that the T77C mutation could result in a reduction in shearable PilA1 relative to the native wild type. To ensure transparency without detracting from the comparative analysis of

the minor pilin mutants, we have added a brief statement to the manuscript acknowledging this (Lines 269-272):

"Fluorescence microscopy of strains expressing a PilA1(T77C) variant which, while possibly exhibiting slightly reduced overall piliation compared to the native WT, successfully retained pilus dynamics, revealed striking morphological differences across the different minor pilin mutant backgrounds that were quantified by semi-automated image analysis (Fig 3 B, C)."

Also, based on the hypothesis the WT should produce both forms of T4P, the long and the short ones. Would the pilus dynamics in WT and mutants differ if in that assay DNA would be included? Has that been tested?

Testing whether extracellular DNA alters the dynamics of Com-type pili would certainly be a valuable addition. Unfortunately, the specific confocal microscope required for our *in vivo* pilus imaging currently has a defective laser. Because adapting this sensitive live-imaging assay to a completely different microscope setup is not easily accomplished, we decided not to conduct these experiments for the revision.

Why was it not possible to create motile deletion strains with the PilA1 T77C mutation, as in the WT?

The exact reason for this observation remains elusive, as the only methodological difference between the strains lies in the specific antibiotic resistance cassettes used for mutagenesis. We speculate that variations in cassette promoter or terminator strengths might cause unintended transcriptional read-through. This could produce an antisense RNA that interferes with the downstream ogt gene—a glycosyltransferase essential for T4P-mediated motility. However, this remains purely speculative.

Why 2% agar pads were used in these assays, while in all other assays (motility, shear-off) 0.5-0.75% agar was used?

We increased the agar concentration to 2% specifically to stabilize the samples during microscopy. When using 0.5–0.75% agar, the pads are prone to microscopic sliding and environmental vibrations (e.g., from air conditioning), and the cells themselves tend to "wiggle" on the softer surface. Because our fluorescence scanning required relatively long exposure times, this movement caused severe image blurring. The 2% agar pads provided the rigid surface necessary to fully immobilize the cells for sharp, high-resolution imaging. However, for long term growing of cultures 0.5–0.75% agar is better suited.

We adjusted the Material and methods to reflect this (Lines 620-622):

“Labeled cells were resuspended in approx. 150 µl BG11 and 3 µl aliquots were spotted onto rigid BG11 agar pads (2% agar, 0.2% glucose, 10 mM TES pH 8, and 0.3% sodium thiosulfate) to immobilize the cells for sharp, high-resolution imaging.”

Line 274-275: It would be interesting to see the different sheared pilus fraction by negative-staining TEM. Are the long and short pili differ in width as reported earlier for Synechocystis and Thermus ? The former should also be mentioned in the discussion.

To address whether the distinct pilus types differ in width, we quantified pilus diameters from negative-staining TEM images of our *Synechocystis* Δ*pilA7-8* mutant, alongside a re-evaluation of Δ*pilA5-6* and Δ*pilA9-12* micrographs from our previous work (Oeser *et al.*, 2021). Our measurements yielded mean diameters of 7.44 ± 0.98 nm (Δ*pilA5-6*), 7.20 ± 0.92 nm (Δ*pilA7-8*), and 6.94 ± 1.13 nm (Δ*pilA9-12*), revealing no statistically significant differences between the strains. While earlier studies (e.g., Hu *et al.*, 2018) reported thinner pili linked to *pilA11* regulation, our evaluation of the minor pilin deletion strains indicates that the Com and EPS pili do not exhibit reliably distinguishable thicknesses under standard TEM. We have added this quantification as a new Supplementary Figure and present these findings in the manuscript (Lines 279-283):

"To determine if the distinct EPS or Com-type pilus assemblies exhibit morphological differences in width, we (re-)evaluated pilus diameters from negative-stain transmission electron microscopy (TEM) micrographs (36). However, no significant differences in thickness were observed between mutants lacking the Com-associated (ΔpilA5-6 = 7.44 ± 1 nm, ΔpilA7-8 = 7.2 ± 0.9 nm) or EPS-associated (ΔpilA9-12 = 6.94 ± 1.1 nm) minor pilins (Fig S9)."

Moreover, we discuss these findings (Lines 393-398):

"However, unlike these species that physically modulate filament width, our TEM quantifications reveal that the distinct Com and EPS pili in Synechocystis maintain a conserved core thickness (~6.9–7.4 nm). While this aligns with observations that minor pilins do not dictate filament diameter (36), it contrasts with a study linking the pilA11 regulator PilR to altered pilus width (53) and we cannot rule out that Synechocystis might assemble T4P with altered diameter under specific conditions."

Line 275: Tone down the sentence: Suggested to cap the Com-type tip complex.

We now write (Line 325):

"... the PilX paralogs hypothesized to associate with the Com-type tip complex"

Figure 2E: Show the structure for all three PilX proteins. I assume PilX1 is slr0226, PilX2 is slr 0442 and PilX3 is slr1268, that is neither evident from your figure (2C) nor the dataset S2. It would be also helpful to add in the Dataset S2 that PilXEPS /slr2018 is PilA12.

We thank the reviewer for highlighting the ambiguity regarding the nomenclature and the need for a broader structural comparison. We agree that the mapping between the locus tags and the assigned pilin names was difficult to follow in the initial draft. To resolve the annotation issues, we now include a summary table (as detailed in our response to an earlier comment). We have also added the new Figure S3, which clearly maps the genetic organization and structural classification of all minor pilin genes in *Synechocystis*.

Regarding the structures of the PilX proteins, we agree that comparing them is highly informative. Rather than expanding Figure 2E, we have introduced a comprehensive new supplementary figure (Figure S4) that displays the AlphaFold predicted structures for the entire *Synechocystis* pilin repertoire, including all PilX variants. Furthermore, in order to demonstrate the structural differences between the PilX subfamilies, we now include the structure-based FoldMason alignments of the Com and EPS minor pilins as the new Figure S5 (again detailed in our response to an earlier comment).

Figure 2F right: Are these representative images of repeated motility and transformation assays. Could the transformation data be quantified as shown in Figure 1F?

As suggested by all reviewers, we have replaced the representative images of the transformation assay with fully quantified CFU data from at least three independent replicates with statistical analyses (Dunnett's post-hoc test) mirroring the format of Figure 1F. This quantitative transformation data is now presented as the new Figure 2G.

Figure 4B: I would be more informative if abundance changes of all 14 pilins would be depicted. Is the abundance of PilA1, PilA2 and PilA4 affected by the different deletions?

We agree that providing a complete overview of the pilin repertoire is much more informative. Accordingly, we have updated the heatmap in Figure 4B to specifically display the abundance changes for all 14 *Synechocystis* pilins. From this updated overview, it is clear that PilA4 abundance is not significantly affected by the different deletions. We do observe that the major pilins PilA1 and PilA2 are more abundant in Δ*pilA5-6* mutant background. However, this increase is notably absent in the Δ*pilA7-8* mutant. Because both of these mutants exhibit the same distinct EPS-type pilus morphology, the specific fluctuation of PilA1 and PilA2 in the Δ*pilA5-6* strain cannot be the driving factor behind this shared pilus morphology. We assume the reviewer's interest in these pilins relates to the broader question of whether the ratio of major to minor pilins affects the observed phenotypes. To address this, we point the reviewer to our newly acquired quantitative LC-MS/MS proteomic data (new Figure S10). As detailed in our subsequent responses regarding pilin stoichiometry, we analyzed these new data to evaluate the intracellular and surface-sheared major-to-minor pilin ratios, confirming that the distinct filament morphologies are not artifacts of drastically altered PilA1 levels.

Minor comments:

Figure S1: Please explain why the WT or corresponding parent strains of the mutants was not used for TEM.

While a wild-type sample was included during our TEM imaging sessions, the resulting micrographs were unfortunately of suboptimal technical quality. Instead, we opted to use the Δ*sll1835* strain which exhibits no T4P-related phenotypes and harbors the exact same antibiotic resistance cassette used to generate the imaged minor pilin mutants. We reasoned that this makes it a highly adequate methodological control, as it accounts for any potential secondary morphological effects caused by the expression of the resistance cassette itself. We have updated the legend for Figure S1 to clarify this.

Revised Figure S1 Legend Sentence:

“A Δsll1835 knockout strain, which has no observable T4P-related phenotypes but harbors the same antibiotic resistance cassette as the minor pilin mutants, served as a methodological control.”

Figure S3B: Decreased levels of T4P formation are seen for one, two or three clones of T151C, T77C, T80C and T105C.

We agree that the screening blot shows varying, and sometimes decreased, levels of T4P formation for these clones. As detailed in our previous response regarding the PilA1(T77C) variant, these variations in the initial unquantified screen likely stem from altered protein stability, differences in pilus fragility during mechanical shearing, or reduced antibody affinity. We added a clarifying statement to the main text acknowledging the potentially reduced piliation levels associated with these mutations (see comment above & Lines 269-272).

Figure S3C: Functional motility for T77C and T105C in 2 out of three clones is obvious but not for T80C.

The presence of some non-motile clones for T77C and T105C is an expected technical artifact of the strain generation process. In *Synechocystis*, it is highly common for random, spontaneous secondary mutations during standard colony selection to abolish motility—typically affecting 10–50% of all picked clones for any given transformation. Regarding the T80C variant, we do not claim in the manuscript that it supports functional motility, which aligns with the absence of any motile clones observed in this assay.

Reviewer #2: Summary:

The major goal of this manuscript was to tackle the challenging question of how functional specialization of T4P is achieved in Cyanobacteria species that contain multiple minor pilin operons. The authors show that deletion of specific minor pilin operons affects distinct T4P-dependent phenotypes (pilA2 and pilA4 are dispensable for motility, transformation, and aggregation, while pilA7-8 are dispensable for aggregation and motility but essential for transformation). The authors use computational analyses to phylogenetically characterize pilins across different species of cyanobacteria, resulting in a clustering of related minor pilins into distinct subclades that may be attributed to their function. The authors then use fluorescence microscopy to visualize T4P dynamics in different minor pilin mutants, followed by mass spectrometry analysis of sheared T4P fractions in different minor pilin mutant backgrounds. While figures 1 and 2 provide interesting and more compelling data, figure 3 lacks control information that is crucial for accurate interpretation of T4P properties. Figure 4 also shows sheared fraction proteomics data that are overinterpreted – without knowing total expression profiles for different minor pilins in each mutant, it is difficult to conclude if changes in certain pilin abundance in sheared fractions is directly due to changes in incorporation or indirectly due to changes in expression. Figure 4 results were also used to draw conclusions about intracellular signaling networks without validating data (such as genetic data or whole cell proteomics data) required for these conclusions.

Thank you for the constructive comments. In the revised version of our manuscript we addressed the criticism.

Major comments:

• The authors' measured data in 1B and the text indicate the ΔpilA7/8 strain has significant aggregation compared to the WT, but the images don't really reflect this – can the authors include a supplemental figure with the replicate data used for quantification to display the hyperaggregation phenotype more clearly?

To better visualize the hyperaggregation phenotype, we have added a new Supplementary Figure S2, which displays the macroscopic cultures and their respective aggregation scores immediately following inoculation. In these images, it is clearly visible that the Δ*pilA7-8* strain flocculates even under conditions where the wild-type strain remains evenly suspended as planktonic single cells. We believe that this clear visual phenotype, combined with the statistically significant increase in the quantitative flocculation score shown in Figure 1B, justifies our use of the term hyperaggregation to describe this mutant.

• Are all of these pilins expressed at the same levels? One simple model for how different minor pilins could determine function is differential expression under different conditions

We completely agree with the reviewer's model. Differential expression under changing environmental conditions is exactly how we hypothesize *Synechocystis* dynamically switches between motility and DNA uptake modes. We explicitly address this regulatory switching in the Discussion (Lines 420-428), noting that the inverse accumulation of Com- and EPS-related proteins mirrors their divergent transcriptional regulation by cAMP and c-di-GMP.

To verify that the minor pilins are expressed at consistent levels under our standard experimental conditions, we screened their expression via qPCR (see below) which demonstrates that the deletion of one set of minor pilins does not significantly alter the baseline transcription of the remaining minor pilin genes:

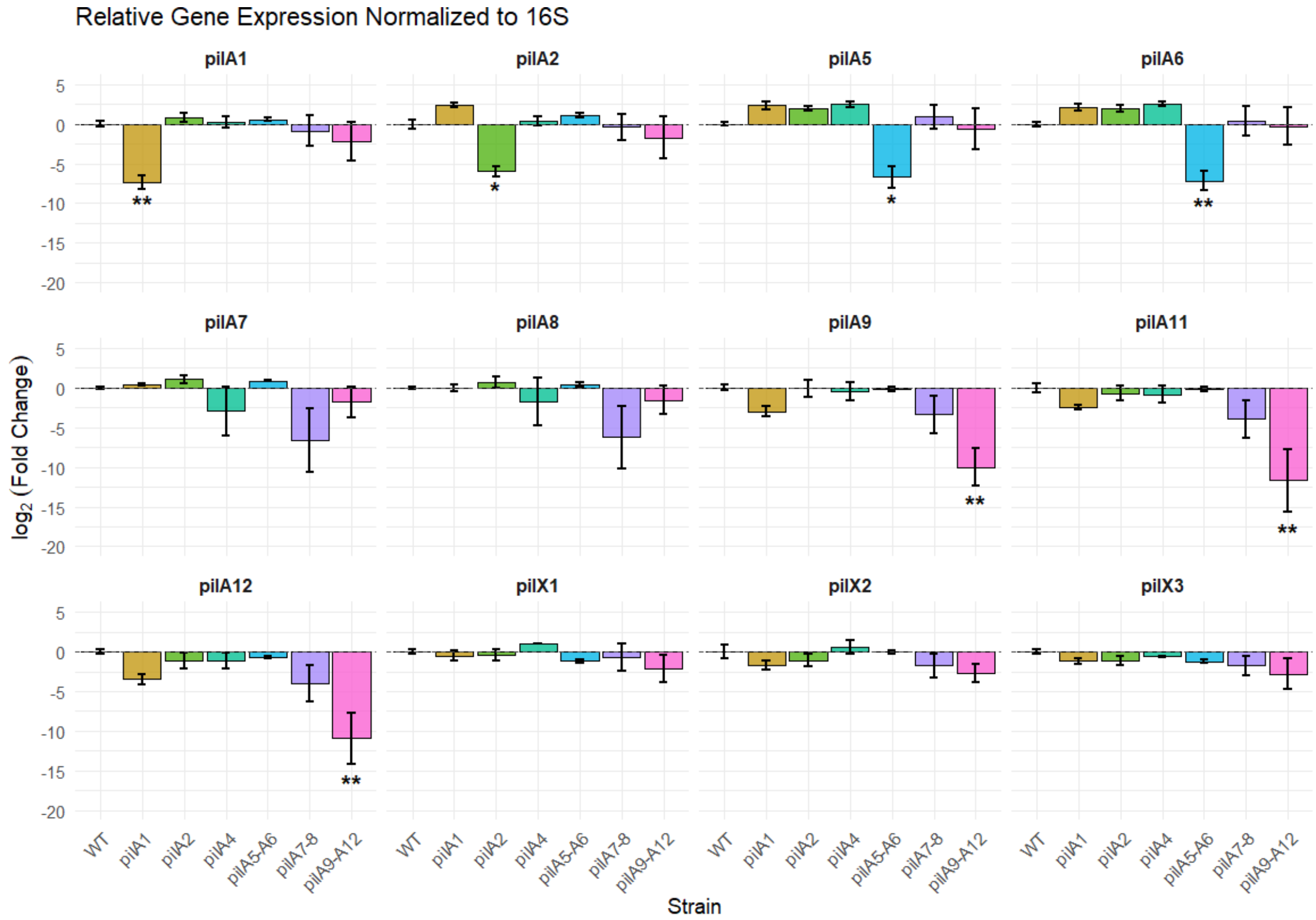

Supporting Figure: Relative expression of pilin genes across Type IV pili mutant strains. Transcript levels were quantified via RT-qPCR and normalized to the 16S rRNA housekeeping gene. Bars represent the mean log2 fold change (-ΔΔCt) relative to the Wild Type (WT) strain. Error bars indicate the standard error of the mean (SEM) from three biological replicates. Statistical significance was assessed using an ANOVA followed by Dunnett's multiple comparisons test, comparing each mutant directly to the WT (* $p < 0.05$, $p < 0.01$, *** $p < 0.001$).

However, we acknowledge that a comprehensive evaluation must extend beyond transcription to confirm actual protein-level stoichiometry and interdependency. As this point is raised in a subsequent comment about the recent study by Christman et al. (2026), we performed a new quantitative LC-MS/MS proteomic experiment. The results, along with our corresponding additions to the manuscript (including the new Figure S10 and Discussion text), are detailed in our response to that subsequent comment below.

• Where do the different pilins relevant to this manuscript fall in the phylogenetic tree? Arrows indicating their position in Fig 2B would be useful

We thank the reviewer for this helpful suggestion. We agree that providing a clear way for readers to navigate the *Synechocystis* pilin repertoire is crucial. While we considered adding arrows to the dendrogram in Figure 2B, we concluded that a more comprehensive standalone overview would better serve the reader. As detailed in our response to Reviewer 1, we have addressed this need by introducing a new summary table (Table 1) and a new Supplementary Figure S3. Together, these additions explicitly map out the genomic organization, structural family classifications, locus tags, and phenotypic roles for all 14 major, minor, and auxiliary

pilins. We believe this approach provides a much more robust and accessible reference guide than annotations on the phylogenetic tree alone.

• The authors should provide quantification for the transformation assays in 2F – the plate images do not clearly depict phenotypes

The reviewer is correct that relying solely on representative plate images can make it difficult to fully assess the phenotypes. To resolve this, and as mentioned in our response above, we have quantified the transformation assays. The exact transformation frequencies for all tested mutants are now presented in the newly added Figure 2G.

• For figure S3, are these 3 lanes for each strain different clones? Clearer labeling is needed to interpret these results

We thank the reviewer for pointing this out. Yes, the three lanes shown for each strain represent three independent biological clones. We have updated the figure legend (now Figure S8) to explicitly state this, ensuring the results are easily interpretable.

• The cysteine knock in strain used by the authors seems to significantly reduce PilA1 surface levels, but the authors do not comment on this or how it might affect their phenotypes. Are reduced surface levels of PilA1 reflective of intracellular PilA1 levels? The answer to this could influence the interpretation of the pilus phenotype results.

(see below)

• Also, is expression of any of the minor pilins dependent on PilA1 levels or on each other? If so, the phenotypes presented and quantified in Fig 3 may not accurately represent the deletion phenotypes. Given the recent preprint showing that the ratio between minor and major pilin levels can significantly affect dynamics (https://www.biorxiv.org/content/10.64898/2026.01.17.700090v1), I am not confident in the interpretation of these data that incorporation of specific minor pilins per se influences these properties. This draws to question the interpretation that T4P made with different minor pilins are morphologically distinct. Expression data for the minor pilins in the cysteine background strain and in relation to deletions of each other would help strengthen this claim.

We thank the reviewer for this insightful comment and for directing us to the highly relevant recent paper by Christman et al., 2026. To address the valid concerns regarding intracellular versus surface pilin levels and their stoichiometric ratios, we have performed a new quantitative proteomic experiment evaluating both the cellular and surface-sheared fractions of the WT and minor pilin mutant strains in the PilA1(T77C) background. These new data are now included in the manuscript as Figure S10 and dataset S4.

Our LC-MS/MS analysis shows that, despite a slight but statistically significant reduction of intracellular PilA1 in the *pilA5-6* mutant background, the protein remains highly abundant overall, and levels are unchanged in the *pilA7-8* and *pilA9-12* backgrounds compared to the WT. Importantly, the cellular stoichiometry of minor-to-major pilins across all mutants is not

significantly altered. We do observe a slight (non-significant) reduction in the cellular minor-to-major ratio in the *pilA5-6* and *pilA7-8* mutant backgrounds. According to the mechanism described by Christman et al., in which a lower ratio reduces pilus initiation frequency, this cellular reduction could be the underlying cause for the reduced number of pili observed in these specific strains.

In the surface-sheared fraction of the *pilA9-12* mutant, the minor-to-major ratio is indeed elevated. While this aligns with the high number of short pili observed in our fluorescence imaging, we cannot definitively disentangle cause from effect here; this elevated ratio may simply reflect the compositional outcome of assembling short pili rather than driving their dynamics.

Crucially, Christman et al. explicitly demonstrated that while pilin stoichiometry alters initiation frequency, it did not affect pilus length in their model. While we cannot definitively rule out that stoichiometry has some effect on length in *Synechocystis*, this study strongly supports our interpretation that the morphological differences (length) between the Com- and EPS-type filaments are intrinsically determined by the specific minor pilin complexes incorporated.

We incorporated these results in the main text (Lines 312-321):

"To verify that the mutant phenotypes were not artifacts of perturbed global expression, we performed stoichiometric profiling of the intracellular and surface-sheared fractions (Fig S10). Although intracellular PilA1 levels were slightly decreased in the ΔpilA5-6 mutant background compared with the WT, the protein remained highly expressed overall, and levels were unchanged in the ΔpilA7-8 and ΔpilA9-12 backgrounds. Moreover, minor-to-major pilin ratios were not significantly altered compared to the WT, though slight reductions in the intracellular ratio occurred in ΔpilA5-6 and ΔpilA7-8 while the minor-to-major pilin ratio was elevated in the sheared pili fraction of the ΔpilA9-12 mutant background. This extracellular shift supports the observed phenotype, in which an abundance of short Com-type filaments leads to a higher ratio of tip-localized minor pilins to major pilins."

Additional we added a paragraph to the discussion (Lines 399-408):

"Recent findings in Vibrio cholerae established that the stoichiometric ratio of minor to major pilins dictates the frequency of T4aP dynamic activity (54). This mechanism offers a possible explanation for the altered pilus numbers in the cyanobacterial minor pilin mutants. The slight reduction in the cellular minor-to-major pilin ratio in the ΔpilA5-6 and ΔpilA7-8 strains might cause the observed reduction in pili numbers (Fig 3). Importantly, altered pilin stoichiometry in V. cholerae does not influence pilus length. Hence, the difference in length between Com- and EPS-type filaments is unlikely to be a mere artifact of varying pilin ratios, and these distinct morphologies are likely intrinsically dictated by the specific core priming complexes utilized. Together, the observed differences in length, abundance, and dynamics are consistent with ..."

• In figure 4, the authors draw conclusions about changes in signaling networks based on changes to the abundance of intracellular proteins found in shear fractions. While this is an interesting observation, it requires significant experimental data to validate. Additionally, was total protein abundance across these strains the same? This can affect mass spectrometry detection limits and quantification accuracy.

The reviewer is right that, while the Label-Free Quantification (LFQ, using log10-transformed values) of our original dataset mathematically accounts for loading variations within the sheared fraction itself, this extracellular measurement cannot account for potential major fluctuations in total intracellular protein abundance across the different mutant strains. We agree that drawing firm conclusions about intracellular signaling solely from an extracellular proxy requires further validation. To directly address this concern, we point to our new, independent quantitative LC-MS/MS dataset of the PilA1(T77C) strains (detailed in our earlier responses). Crucially, this experiment analyzed both the intracellular (whole-cell) and surface-sheared fractions from independent clones. This new whole-cell dataset successfully validates our initial findings. As shown in the volcano plots of the new Figure S10C, we observe the same significant differential regulation of the proteins highlighted in our text in the Δ*pilA9-12* mutant background. However, because the validation of the proteins directly within the intracellular fraction of Δ*pilA9-12* mutant so clearly corroborates the protein abundance trends we initially observed in the sheared fraction, we are highly confident that the original approach in Figure 4 reflects genuine biological shifts in the signaling networks rather than artifacts stemming from varying intracellular protein pools.

We added the following sentence to corroborate our findings (Lines 349-350):

"The differential regulation of these proteins was independently validated in cellular and sheared extracts of the ΔpilA9-12/pilA1(T77C) mutant (Fig S10C)."

Also why isn't pilA7 shown in the heat map?

• What are each of the additional proteins shown in the heat map in fig 4 and why are they relevant?

We agree that the inclusion of various non-pilin proteins in the original heatmap lacked sufficient context, as their specific roles were not detailed further in the manuscript. To resolve this and provide a more focused analysis, we have completely revised Figure 4B. As mentioned in our response to a previous comment, the updated heatmap now exclusively displays the abundance changes for all 14 *Synechocystis* pilins, removing the unrelated proteins entirely.

Minor comments:

• Line 37 "a" should be removed from "a membrane-spanning T4P-machinery"

To our knowledge, singular count nouns require an article, and "a membrane-spanning T4P-machinery" should be correct. But we will happily remove the "a" from the proofs if this is flagged again.

• The authors should include a WT image of the aggregation phenotype in 1E as they do for the mutants for comparison purposes

The decision to omit the wild-type image from Figure 1E was driven primarily by space and layout constraints. Adding an additional panel would break the current symmetry of the figure and require reducing the size of all aggregation images, making differences in chlorophyll distribution harder to see. Given that the baseline wild-type aggregation is already depicted in Figure 1B, we prioritized a larger, uncrowded layout for the mutant comparison in panel 1E. Nevertheless, if the reviewer considers it essential, we are happy to adjust the figure.

• Why is there a 10-fold difference in transformation rates in the WT between 1C and 1F? The error bars look pretty small on the WT in both panels, so it seems strange to have this amount of variability between experiments; also looks like there is significant variability in ΔpilA2 strain in 1F – can the authors comment on this?

The difference in absolute wild-type transformation rates between independent experiments (Fig. 1C vs. 1F) reflects the inherent variability of this assay. We suspect that absolute efficiencies are heavily influenced by subtle, hard-to-control factors, such as agar surface dryness during plating and exact antibiotic diffusion rates. Because of this high inter-assay variability, we ensure that all strains and replicates for a given experiment are plated simultaneously on a single, fresh batch of plates.

Regarding the variance in the Δ*pilA2* strain: unlike the wild type, our mutants are independently propagated biological clones. This independent propagation naturally introduces an additional level of biological variance (e.g., due to slight differences in culture adaptation or undetected secondary mutations), which, when combined with the inherent sensitivity of the assay, likely leads to the large variability observed in the mutant. Despite this, the data clearly show that PilA2 is neither crucial for motility nor required for DNA uptake.

Reviewer #3:

The manuscript by Schuergers et al. describes their characterization of pilins in cyanobacteria by phylogenic/structural analysis and by assessing phenotypes of putative pilin gene mutants. They find that pilins cluster into distinct families, primarily PilA (major pilin) and minor pilins PilV, PilW, PilX and FimT, which have been characterized in other systems. They show that the minor pilins of Synechocysis and Thermosynechococcus vulcanus are involved in discrete functions of transformation (Com) or motility (EPS) while for the major PilA1 is associated with both functions, at least for Synechocystis. From an analysis of pilus length, piliation levels and pilus dynamics they propose that the different minor pilins form morphologically distinct pili. The results are interesting and nicely described in the Author Summary ("We discovered that cyanobacteria assemble two distinct types of filaments using the same basic machinery." However the description of these filaments as morphologically distinct in the Abstract and throughout the text is an overinterpretation of the results of the morphological analysis shown in Fig. 3. With no statistical analysis these data do not support the existence in Synechocystis of morphologically distinct filaments. Differences in the priming complexes may alter the efficiency of pilus assembly and retraction (or, more likely of "re-assembly"), as well as adhesion, all of which could impact pilus dynamics, but this is not the same as having morphologically distinct pili. Overall, the manuscript reads to some degree like an internal document that assums deep knowledge of past results and publication and gene targets. A table summarizing the genes, proteins, deletion phenotypes, classifications and organism would have been helpful, as would more such information on each figure. More specific comments are provided below, presented as the issues appear in the manuscript.

We thank the reviewer for the insightful comments. According to the comments of both other reviewers, we now include a detailed table summarizing annotations and phenotypes.

Line 26 (Abstract) and elsewhere - What is meant by "tip plug"? Though this term has been used elsewhere it is not clear why the tip must be plugged - suggests that something may otherwise leak out. Rephrase.

In the T4P literature, it has been suggested that PilY1 sits as a "champagne-cork-shaped density" within the outer membrane secretin pore that effectively blocks the secretin channel. But to our knowledge, there is no experimental verification for this plugging function and we agree that it might be better to rephrase this term. Therefore, we now write that PilX/PilY-homologs are capping the distal end of the pilus filament.

Line 86 paragraph (Intro) – At the end of the Introduction the authors pose the question "whether cyanobacteria possess a single T4P machine incorporating specialized accessory pilins or assemble multiple, distinct filaments" but it is not clear what the answer is. They seem to suggest the latter, but it appears to be the former - a single T4P formed from the major pilin PilA1 (at least for Synechocystis), but having one or the other minor pilin priming complex at its tip.

We agree that the original question posed at the end of the Introduction set up a confusing dichotomy. As noted in our response to a similar comment from another reviewer, we have revised this language here and throughout the manuscript to eliminate the implication of separate basal motors. Please also see our response to this reviewer's related comment below.

Line 86 paragraph (Intro) – They distinguish between the "machinery" required for motility and DNA uptake, suggesting there are two distinct machines, but in fact there appears to be a single T4P that differs only in its minor pilins at the pilus tip. In the T4P field, the term machinery" is typically used to describe the components that assemble the pilus, i.e. assembly ATPase, platform protein, secretin channel etc., none of which are discussed here. Are there distinct sets of assembly ATPase, platform protein, secretin channel etc. or a single set? What about PilY1?

We agree with the reviewer that 'machinery' should refer strictly to the core basal assembly apparatus (ATPases, platform, secretin, etc.), of which *Synechocystis* encodes only a single set. To correct this, we have revised the final paragraph of the Introduction. The text now explicitly acknowledges the single assembly apparatus and clarifies that the functional specialization is achieved by producing morphologically distinct filaments, rather than distinct machineries.

Regarding the reviewer's question about PilY1: because this protein has not been described in cyanobacteria, we chose not to mention it in this context. Instead, we address this in the Discussion, where we argue that the large domains grafted onto cyanobacterial PilX and other bacterial homologs like ComZ serve a functional role analogous to PilY1.

The main text now reads (Lines 88-90):

"This raises the question of whether the single T4P assembly machinery typically found in cyanobacteria (39), secretes a single, multi-purpose filament incorporating specialized accessory pilins or assembles multiple, distinct filaments."

Line 100 paragraph (Results) – Little is said about PilA1. Remind the reader that PilA1 is a major pilin. Is it the only major pilin in this strain? Two are shown in Fig. 2C and Supp Data Set 2. Which of these corresponds to the PilA1 gene. Was the second major pilin gene deleted and if so, what was the phenotype?

We apologize for the confusion regarding the major pilins. The reviewer is completely correct to point this out; the ambiguity arose because our original figures and datasets listed the two major pilin genes (*pilA1* and *pilA2*) under their automated annotations (*hofG*), making them difficult to distinguish. To directly answer the reviewer's questions: PilA1 is indeed the primary major pilin. The second major pilin homolog shown is PilA2. We did delete *pilA2*, and as shown in our phenotypic assays (Figure 1), the mutant is fully capable of both phototactic motility and natural transformation. This indicates that PilA2 is neither crucial for T4P assembly nor for these specific functions under the conditions tested. To prevent similar confusion for the reader, we have – as outlined in our response to a previous comment - added a new summary table (Table 1) and a new Supplementary Figure S3 that explicitly map all 14 major, minor, and auxiliary pilins—clearly linking their trivial names (PilA1, PilA2) to their specific locus tags, family classifications, and phenotypic roles. Additionally, we have updated Supplementary Datasets S1 and S2 to replace the generic *hofG* labels with the exact *pilA1* and *pilA2* annotations.

Line 100 paragraph (Results) – The authors state that the aggregation results for the pilA1 deletion do not agree with previous reports but do not elaborate. What were the previous results and did they use the same strain? Could a second major pilin have been present? How is aggregation scored? Emphasize here and elsewhere that PilA1 appears to be required for competence, motility and aggregation, which would suggest that it is pili formed by this major pili that confers all these functions.

The previous study (Conradi et al., 2019) used a similar experimental setup in another laboratory and the identical scoring algorithm to measure aggregation, but employed a different wild-type background. Because aggregation strictly depends on active pilus extension (as evidenced by the lack of aggregation in Δ*pilB* or Δ*pilC* assembly mutants), and because PilA1 is indispensable for any observable T4P filament formation or function (Bhaya et al., 1999, 2000; Yoshihara et al., 2001; this study), we are confident in our finding that a Δ*pilA1* mutant cannot aggregate. We hypothesize that the mutant analyzed in the prior report was likely not fully segregated, leaving sufficient functional PilA1 to support aggregation. However, because we cannot definitively prove this and it is not the main focus of our current study, we opted not to elaborate on this hypothesis in the main text. Instead, to maximize clarity without over-explaining, we adjusted the sentence in the manuscript to explicitly state what the previous study observed and moved the citation directly to that claim.

The text now reads (Lines 109-112):

"Notably, the lack of aggregation in our ΔpilA1 mutant contrasts with previous reports showing flocculation of a ΔpilA1 strain (34), yet is fully consistent with the dependence of flocculation on pilus assembly by the PilB1 extension motor."

Moreover, we completely agree with the reviewer that PilA1's essentiality for competence, motility, and aggregation should be explicitly stated to emphasize that a single major pilin forms the foundation for all these functions. We have updated the Introduction to reflect this (Lines 80-82):

"This functional complexity is reflected in a genome encoding, apart from the major pilin PilA1, which is indispensable for filament assembly and T4P-mediated functions, at least 13 additional putative pilins."

Line 135 section - Bioinformatic analysis on 90 cyanobacterial species was performed to identify putative pilin genes, and their structures were predicted and compared, resulting in clustering into 6 different families, but primarily PilA (major pilin) and minor pilins PilV, PilW, PilX and FimT. The structural features of the PilX family were discussed but it wasn't clear what features resulted in the PilV, PilW and FimT minor pilin classifications. Show characteristic structures for all classifications and compare these to empirically determined structures. What features cause the split in PilX and FimT families? Do all PilX proteins have a hydrophobic residue at position 5 of the mature protein? What about the other pilins?

The reviewer is right that a comprehensive structural comparison to clarify the defining features of all minor pilin families would strengthen the manuscript. To address this, we have introduced a new supplementary figure (Figure S4) displaying the AlphaFold-predicted structures for the entire *Synechocystis* pilin repertoire, and we now provide structure-based FoldMason alignments of the complete minor pilin subfamilies (Figure X).

The defining structural features of our cyanobacterial pilin families strongly align with empirically determined structures of minor (pseudo-)pilins from other bacteria. For PilW, cyanobacterial variants exhibit an exceptionally long αβ-loop region that accommodates up to five additional antiparallel β-strands, perfectly mirroring known structures like *Thermus thermophilus* Tt1219 (Karuppiah *et al.*, 2016). The cyanobacterial FimT homologs strictly conserve the central β-strand swap characteristic of experimentally determined FimT/FimU/GspH structures from heterotrophic models (Nguyen *et al.*, 2015, Braus *et al.*, 2022). This topology folds into two distinct antiparallel β-sheets connected non-sequentially, where strands β1, β2, β3, and β5 form the first sheet, and strands β4, β6, β7, and β8 form the second. The PilA9-like FimT variants are distinguished by the insertion of a distinct extra domain (CATH Superfamily 2.40.30.20) between the second and third β-strands.

The comparison of the PilX and PilV subfamily alignments revealed the following. $PilX_{Com}$ variants exhibit a compact fold with an expanded αβ-loop, whereas the $PilX_{EPS}$ subfamily is massively expanded by a highly elongated β-solenoid and grafted distal domains (see our answer to the earlier question on PilX for further details). Furthermore, the PilV family divergence is characterized by the expansion of loop regions within the core fold. Specifically, the $PilV_{EPS}$ subfamily features a significantly elongated sequence between strands β3 and β4 that packs loosely atop the core β-sheet. This region is highly enriched in helix-breaking and polar residues (e.g., Pro, Gly, Ser, Thr), characteristic of a highly flexible, solvent-exposed extended coil rather than a rigid structural domain.

Finally, a sequence alignment (Oeser *et al.*, 2021) supports the capping function of PilX. All *Synechocystis* PilX variants indeed feature a hydrophobic residue (L/M) at position +5, while all other minor pilins retain the canonical glutamate (E) at this position.

We now incorporate these findings in the results section (Lines 144-183):

"... we could consolidate [the cyanobacterial pilin proteins] into six major families: the major pilin PilA family, a related auxiliary pilin family, and families with homology to the four core minor pilins, that is, the FimT, PilW, PilV, and PilX families (Fig 2B, Datasets S1-S2). ***The genomic organization, structural classification, and predicted structures of the 14 Synechocystis pilins are detailed in Supplementary Figures S3 and S4.***

The PilA family constitutes the canonical major pilin cluster. A divergent family of 'auxiliary pilins' was also identified, though with weak statistical support. We chose this specific nomenclature because these proteins are likely unnecessary for assembly, as seen with PilA4, despite their structural similarity to the major pilin. ***Structure-based alignments (Fig S5) revealed that the core minor pilins share defining features with empirically determined structures from other bacteria. For example, cyanobacterial PilW homologs exhibit an exceptionally long αβ-loop region accommodating additional antiparallel β-strands, mirroring known structures such as Thermus thermophilus Tt1219 (41). Similarly, the cyanobacterial FimT family strictly conserves the central β-strand swap characteristic of FimT/FimU homologs (20, 42).*** *The FimT family split clearly into a major cluster and a minor group containing Synechocystis PilA9,* ***which is structurally distinguished by the insertion of an extra domain between the second and third β-strands****.*

Crucially, the clustering analysis revealed a deep split within the PilV and PilX families, which clearly defined two subfamilies. Consistent with this divergence, a strict co-occurrence pattern was observed between the PilX and PilV subfamilies, even when the genes were not colocalized, suggesting a functional linkage (Fig 2C, D). All cyanobacterial PilX homologs feature a canonical pilin fold interrupted by a bulky, triangular β-solenoid domain that resembles ice-binding proteins and other adhesive domains inserted between the penultimate and C-terminal β-strands (Fig 2E). ***However, structure-based alignments reveal an architectural division (Fig S5). One subfamily is relatively compact, featuring an expanded αβ-loop region. In contrast, the other subfamily exhibits structural expansion driven by a highly elongated β-solenoid and the frequent addition of lineage-specific domains grafted onto the distal end of the solenoid. Similarly, the PilV subfamilies diverge structurally; the PilV co-occurring with the expanded PilX features a significantly elongated, flexible coil region between strands β3 and β4 that is enriched in helix-breaking and polar residues.***

Notably, the sequence profile of HpsA, a protein essential for motility and hormogonium polysaccharide (HPS) secretion in Nostoc punctiforme (43), reliably maps only to this enlarged PilX subfamily, demonstrating that the structural divergence is reflected at the sequence level. *Although only a few cyanobacterial strains differentiate hormogonia, extracellular polysaccharides (EPS) are likely universally required for T4P-mediated motility in cyanobacteria. Consequently, we designated this subfamily as $PilX_{EPS}$ and the co-occurring PilV clade as $PilV_{EPS}$. The remaining PilX variants are associated with the PilV subfamily that includes competence-associated pilins such as Synechocystis PilA5* ***(36)*** *and S. elongatus RntB* ***(37)****. Consequently, we designated these subfamilies as $PilX_{Com}$ and $PilV_{Com}$."*

Line 204 section (Results) - Following the structural classification, deletions were made in putative pilin genes/gene clusters in Thermosynechococcus vulcanus. Why this cyanobacteria? What is its pilin gene organization? It is not shown in Fig. 3C nor in the Supplementary Materials. Do the authors mean T. vestitus?

The reviewer is right to call this out and we admit that the unexplained jump between the two strain names was undeniably confusing. As detailed in our response to an earlier comment, we intentionally used *T. vulcanus* NIES-2134 for the functional assays, even though our structural analysis (Fig. 3C, Dataset S2) utilized the NCBI reference genome *T. vestitus* BP-1. We had to make this switch because the *T. vestitus* laboratory strain harbors a known *pilC* frameshift mutation, rendering it unsuitable for assaying T4P functions. Because the two strains share >99% protein identity, their pilin gene organization is functionally identical. This allowed us to confidently target the homologous *T. vulcanus* loci (*tlr0680-82* and *tll2333-35*) based on the *T. vestitus* predictions. We have updated the Results section (Lines 229-236) to explicitly clarify this strain substitution and added the genomic organization of *T. vestitus* in Figure S3.

Line 204 section and Fig. 2F (Results) – The transformation results are not visible and not quantified. Please present as for Fig. 1.

As detailed in our response to an earlier comment raising this point, we have updated the manuscript and the respective figure to explicitly quantify and display the transformation results, matching the presentation format used in Figure 1.

Line 204 section and Fig. 2F (Results) – Deletion of the second major pilin homolog tll1019 resulted in reduction of motility. What about the first major pilin homolog – what is its phenotype? To show that the system shown for Synechocystis, a single pilus with two distinct tips imparting unique functions, is universal for cyanobacteria, the authors should show that T. vulcanus also uses a single major pilin.

We agree that acknowledging the reliance on a primary major pilin provides helpful context. We did not independently knock out the first major pilin homolog (*tll1011*) in this study because its crucial role for pilus function has already been established in the literature (Nakane *et al.*, 2022). While it has not been verified that *tll1011* is strictly required for T4P assembly, imaging confirms Tll1011 is present along the entire length of the pilus.

Because defining the exact mechanistic roles and the interplay between the primary and secondary major pilins (e.g. PilA1 vs PilA2 or Tll1011 vs. Tll1019) falls outside the core focus of our study, we opted not to elaborate further in the manuscript. To provide the necessary background without shifting the paper's focus, we have added a brief sentence referencing the role of tll1011:

"Previous work has demonstrated that the major pilin homolog Tll1011 (PilA1), is essential for T4P-dependent functions (66)."

Line 234 section and Fig. 3 (Results) – Since the study started with Synechocystis then went to T. vulcanus and then back to Synechocystis, please clearly state which organism is associated with which gene cluster in the text and all figures. Describe the genome organization for the Synechocystis pilins, including the three pilX genes, before describing the experiments. What is the hypothesis re. the pilX genes? They are classified as Com but what are their phenotypes re. motility and competence? Why was pilX_EPS not examined?

We thank the reviewer for highlighting these points. To ensure clarity about the organisms, we have revised the text to explicitly state when we refer to *Synechocystis*. For example, we have updated the transitions to explicitly name *Synechocystis* in discussions of single-operon mutants, AlphaFold predictions, and in vivo imaging

(Lines 250-251):

"The functional differentiation and persistence of surface pili in single-operon mutants of Synechocystis (Table 1) suggest that the two core minor pilin sets prime separate pilus types."

(Lines 200-201):

"AlphaFold predictions of Synechocystis PilA12 (AF-P73238) and PilX3 (AF-P73549)."

(Line 267):

"To visualize potential differences in Synechocystis pilus morphology and dynamics in vivo..."

To provide the reader with a clear overview of the genomic organization and phenotypes of the *Synechocystis* pilins prior to these experiments, we have introduced the comprehensive summary table (Table 1) and the new Supplementary Figure S3 as described in the earlier comments.

Regarding the *pilX* genes: our working hypothesis was that these proteins likely cap the Com-type pilus tip. Because *Synechocystis* encodes multiple $PilX_{Com}$ homologs (PilX1-3), we assumed potential functional redundancy. This explains why single *pilX* knockouts retain natural competence, hinting at a shared function at the tip. To keep the manuscript focused, we have kept this assumption brief in the main text, while their specific motility and competence phenotypes are now explicitly cataloged in the new Table 1.

The Results now state (Lines 275-278):

"In contrast, differences in the pilX mutants were less pronounced. Because we hypothesize that these multiple PilX homologs function redundantly to cap the Com-type pilus tip, the loss of a single pilX gene is likely compensated by the others. Nevertheless, the ΔpilX1 mutant showed ..."

Finally, we apologize for the confusion surrounding pilX_EPS. This misunderstanding stemmed directly from our initially suboptimal naming scheme. We hope that our new Table 1 guides the reader by clearly mapping all structural classifications to their corresponding trivial names and locus tags. We did indeed examine $PilX_{EPS}$, which is actually PilA12 (slr2018), but we analyzed it as part of the complete deletion of the *pilA9-12* transcriptional unit rather than as a single-gene knockout.

Line 234 section and Fig. 4 (Results) – Despite several claims of "statistically significant differences", stats are not provided for most comparisons. Line 239 reports "striking morphological differences" in pilus dynamics among the mutants, which are not apparent to this reviewer given the large error bars. The same can be said for reported differences in piliation levels and lengths. Yet these data lead the authors to the major conclusion of the manuscript, that there are two morphologically distinct pilus filaments, each with a different function.

We completely agree the reviewer that explicitly showing the statistical significance in Figure 3 (which we assume is the figure in question) is crucial for supporting our major conclusions regarding the distinct pilus morphologies.

Prompted by this comment, we completely reassessed our statistical approach to provide the most rigorous analysis possible. We transitioned from basic non-parametric tests (Wilcoxon) to (Poisson Generalized) Linear Mixed-Effects Models (GLMMs/LMMs) with Dunnett's post-hoc correction. This upgrade explicitly avoids pseudoreplication by treating imaged clones (plates), and acquisition dates as random effects, providing superior statistical power. This analysis confirms our original conclusion: the morphological differences are indeed statistically significant. Interestingly, this improved statistical stringency revealed that the minor increase in pilus length we previously reported for the *ΔpilX3* mutant is actually statistically insignificant, which aligns with our hypothesis regarding PilX functional redundancy. To ensure total transparency, we have updated Figure 3 to clearly indicate all statistical significance using standard asterisk notation. We have also updated the corresponding figure legend and the Materials & Methods section to explicitly detail the use of these mixed-effects models (Lines 632-636):

"Analyses were conducted in R using mixed-effects models with imaged clones and acquisition date as random effects to avoid pseudoreplication. Log-transformed pilus length was analyzed using a Linear Mixed Model. Pilus count and dynamic events were analyzed using a Poisson Generalized Linear Mixed Model. All mutant-to-WT comparisons used Dunnett's post-hoc adjustment."

283-284 (Results) - What is meant by "enrichment of one coincides with depletion of the other? Enriched how? By knocking out the other genes? More accurately, "depletion of one results in enrichment of others".

We agree that flipping the sentence to focus first on the depletion is much clearer. However, we have deliberately chosen not to use the phrase "results in" to avoid implying a direct, proven mechanistic or regulatory causality.

Instead, to accommodate the reviewer's request for clearer directionality, without overstating the underlying mechanism, we have revised the text to state that (Lines 339-340):

"...we observed a reciprocal accumulation pattern: the depletion of EPS pilins (PilA9-12) was accompanied by an enrichment of Com pilins (PilA5-8)..."

Line 322 paragraph (Discussion): "The Com filament primed by PilA5-8 is short, abundant, and hyper-dynamic. We hypothesize that this morphology is biophysically optimized for DNA scavenging, where rapid extension and retraction cycles increase the sampling frequency required to capture DNA molecules. Conversely, the EPS filament (primed by PilA9-12) is long, sparse, and persistently attached." These statements, along with claims made in the title, abstract and elsewhere, are not supported by the data. From the data presented, it appears that the two pilus types differ only in their minor pilin priming complexes (which could affect their dynamics).

We agree that our original terminology (e.g., 'specialized nanomachines') may have

inadvertently implied the existence of entirely separate basal motors or distinct major pilins. We completely agree that *Synechocystis* relies on a shared basal apparatus and a common major pilin (PilA1) shaft. We have revised the Abstract, Introduction, and Discussion to explicitly state that a single shared machinery assembles filaments primed by distinct core minor complexes.

However, we respectfully disagree that these filaments are not 'morphologically distinct.' Morphology encompasses physical form and dimensions, not protein composition. Our quantifiable data demonstrates profound, statistically significant differences in pilus length and abundance depending on the incorporated priming complex. A remarkably long, sparse filament is morphologically distinct from a short, highly abundant one, even if their shaft composition is identical.

While recent studies in heterotrophic models are beginning to link specific (minor) pilin proteins to distinct functions, demonstrating that specialized core minor pilin sets intrinsically dictate the physical dimensions, dynamics, and function of the resulting appendage represents a significant conceptual advance. This is particularly critical in cyanobacteria, where the identification and unified annotation of these clusters - let alone their functional decoupling - has remained largely unresolved until now. We are confident that our revised text now clearly conveys this model: a single basal apparatus and major pilin that, when primed by distinct minor pilin sets, assemble two morphologically and functionally distinct filaments
