# Supplementary material for: Distinct core minor pilin complexes prime specialized type IV filaments in cyanobacteria
Source: PLoS Genet. 2026 Oct 26;22(10):e1012337. doi: 10.1371/journal.pgen.1012337 (PMC13759059; doi:10.1371/journal.pgen.1012337)
Supplement: Supplementary file 7 [file pgen.1012337.s030.pdf]

We are pleased that the previous revisions were well received and would like to thank the reviewers again for their support and constructive feedback.

Reviewers' comments:

Reviewer's Responses to Questions

Comments to the Authors:
Please note here if the review is uploaded as an attachment.

Reviewer #1: The authors have done a very good job of addressing all my comments and suggestions as well as those of the other reviewers.
The authors improved the manuscript significantly and the new table and supplementary figures are very helpful.
This paper will be of broad interest for scientist in the field of Type 4 pili biology, biofilm formation and DNA transformation.

Reviewer #2: The new version of the manuscript is significantly improved and is much clearer. The authors have largely addressed my concerns, although there are a few minor points that remain:

Minor comments:

Please provide PAE plots for AlphaFold-predicted structures. For example, Fig S6 shows two predicted protein structures, but the large disordered loops are likely not accurate. It would be valuable to know which parts of the proteins are confidently predicted. For structures pulled from AlphaFold database, this info is already available and doesn't need to be provided

The structures shown in Figure 2 and Supplementary Figure S6 were retrieved directly from the public AlphaFold Database. To ensure readers can easily locate the confidence metric, we have updated the figure legends for both Figure 2 and Figure S6 to explicitly provide the specific AlphaFold model identifiers and the database version used. For the de novo AlphaFold multimeric tip complexes shown in Supplementary Figure S7, we have now included the corresponding Predicted Aligned Error (PAE) plots of the minor pilin complex alongside the structural models, with subunit boundaries clearly indicated along the axes to visualize the confidence of the predicted protein-protein interactions.

I am not fan of referring to pili made of the same major pilin as different types of filaments since there are species that make different pili with different major pilins. Specifically, lines 90-96, confuses this point. I suggest the authors reword this section to make it clear that the pili are made of the same major pilin. I suggest avoiding using terms like morphologically distinct in relation to the filament, and instead emphasize that the differences are in the tip complex.

We agree with the reviewer that it is crucial to avoid confusion with bacterial species that utilize entirely different major pilins to build different pili. However, because our data demonstrate significant differences in filament length and dynamics, 'morphologically distinct' effectively captures this physical divergence. To address the reviewer's concern, we have revised the text to explicitly define this term. We now clarify that these morphological differences refer strictly to the

macroscopic lengths and dynamics imparted by specialized tip priming complexes onto a shared major pilin backbone, rather than any variation in major subunit composition.

The text now reads (Lines 91-96):
Here, we combined structural phylogenomics with functional genetics to demonstrate that Synechocystis utilizes distinct sets of core minor pilins to assemble specialized tip complexes that prime polymerization of a shared major pilin. By conferring specific lengths and retraction dynamics to this common structural backbone, these complexes prime morphologically distinct pili, thereby distinguishing the machinery required for motility from that involved in DNA uptake.

The authors have provided a reasonable explanation to the large discrepancy in transformation rate differences between experiments. For clarity for the reader, can this information be added to the transformation section of the methods?

We agree that including this context is helpful for the reader. We updated the Materials and Methods to explicitly mention the inherent inter-assay variability and clarified that, to control for this, we plated all comparative experiments simultaneously on a single batch of plates using independently propagated biological clones:

The text now reads (Lines 521-524):

Because absolute transformation efficiencies vary between experiments due to subtle differences in assay conditions (e.g., agar dryness and antibiotic diffusion), all strains and biological replicates for a given comparison were processed simultaneously on a single, fresh batch of plates. Mutants were assayed as independently propagated biological clones.

Reviewer #3: The authors have done an impressive job of addressing reviewer comments.

The only point I would add is that although the term "plug" was removed from the text it remains in the Abstract and should similarly be replaced with "cap".

We thank the reviewer for catching this oversight and apologize for the confusion. Although we updated the abstract in the manuscript file during the previous revision, in order to change the terminology from "plug" to "cap", we inadvertently did not apply this change to the separate abstract text field in the journal's online submission portal. We have now updated the submission form to perfectly match the revised manuscript file, ensuring the term “plug” is replaced everywhere.
