# Supplementary material for: Distinct core minor pilin complexes prime specialized type IV filaments in cyanobacteria
Source: PLoS Genet. 2026 Oct 26;22(10):e1012337. doi: 10.1371/journal.pgen.1012337 (PMC13759059; doi:10.1371/journal.pgen.1012337)
Supplement: Peer review history

# Peer review: Decision Letter 0

**DOI:** 10.1371/journal.pgen.1012337.r001

**Authors:** Sean Crosson, Ankur Dalia

25 Mar 2026

PGENETICS-D-26-00203

Distinct core minor pilin complexes prime specialized type IV filaments in cyanobacteria

PLOS Genetics

Dear Dr. Schuergers,

Thank you for submitting your manuscript to PLOS Genetics. After careful consideration, we feel that it has merit but does not fully meet PLOS Genetics's publication criteria as it currently stands. Therefore, we invite you to submit a revised version of the manuscript that addresses the points raised during the review process.

Please submit your revised manuscript by May 24 2026 11:59PM. If you will need more time than this to complete your revisions, please reply to this message or contact the journal office at plosgenetics@plos.org. When you're ready to submit your revision, log on to https://www.editorialmanager.com/pgenetics/ and select the 'Submissions Needing Revision' folder to locate your manuscript file.

Please include the following items when submitting your revised manuscript:

* A letter that responds to each point raised by the editor and reviewer(s). You should upload this letter as a separate file labeled 'Response to Reviewers'. This file does not need to include responses to any formatting updates and technical items listed in the 'Journal Requirements' section below.

* A marked-up copy of your manuscript that highlights changes made to the original version. You should upload this as a separate file labeled 'Revised Manuscript with Track Changes'.

* An unmarked version of your revised paper without tracked changes. You should upload this as a separate file labeled 'Manuscript'.

If you would like to make changes to your financial disclosure, competing interests statement, or data availability statement, please make these updates within the submission form at the time of resubmission. Guidelines for resubmitting your figure files are available below the reviewer comments at the end of this letter.

We look forward to receiving your revised manuscript.

Kind regards,

Ankur B. Dalia

Academic Editor

PLOS Genetics

Sean Crosson

Section Editor

PLOS Genetics

Aimée Dudley

Editor-in-Chief

PLOS Genetics

Anne Goriely

Editor-in-Chief

PLOS Genetics

**Additional Editor Comments:**

As you will see in the reviewer reports below, all three reviewers have a substantial number of concerns with the manuscript in its current form. These include (1) a number of issues related to the clarity and consistency of the text, (2) a lack of quantitative rigor in the way some data are presented or statistically analyzed, (3) issues related to overinterpretation of the conclusions drawn from the data collected, and (4) a lack of appropriate control experiments. Each reviewer provides excellent suggestions on how to address each of these concerns. This will require substantial changes to the text to make the manuscript more accessible to a broader readership, quantification and/or reanalysis of existing data, appropriately qualifying the claims based on the data presented, and by performing a substantial number of additional experiments to support the model presented.

**Journal Requirements:**

1) Please ensure that the CRediT author contributions listed for every co-author are completed accurately and in full.

At this stage, the following Authors/Authors require contributions: Nils Schuergers, Lars Wittig, Gen Enomoto, Julia Herz, Shamphavi Sivabalasarma, Katharina Ruoff, Friedel Drepper, Sonja-Verena Albers, Pitter F. Huesgen, and Annegret Wilde. Please ensure that the full contributions of each author are acknowledged in the "Add/Edit/Remove Authors" section of our submission form.

The list of CRediT author contributions may be found here: https://journals.plos.org/plosgenetics/s/authorship#loc-author-contributions

2) We ask that a manuscript source file is provided at Revision. Please upload your manuscript file as a .doc, .docx, .rtf or .tex. If you are providing a .tex file, please upload it under the item type u2018LaTeX Source Fileu2019 and leave your .pdf version as the item type u2018Manuscriptu2019.

3) Please upload all main figures as separate Figure files in .tif or .eps format. For more information about how to convert and format your figure files please see our guidelines:

https://journals.plos.org/plosgenetics/s/figures

4) We have noticed that you have uploaded Supporting Information files, but you have not included a list of legends. Please add a full list of legends for your Supporting Information files after the references list.

5) Please amend your detailed Financial Disclosure statement. This is published with the article. It must therefore be completed in full sentences and contain the exact wording you wish to be published.

State what role the funders took in the study. If the funders had no role in your study, please state: "The funders had no role in study design, data collection and analysis, decision to publish, or preparation of the manuscript.".

If you did not receive any funding for this study, please simply state: u201cThe authors received no specific funding for this work.u201d

**Reviewers' comments:**

Reviewer's Responses to Questions

**Comments to the Authors:**

**Please note here if the review is uploaded as an attachment.**

Reviewer #1: Distinct core minor pilin complexes prime specialized type IV filaments in cyanobacteria

Nils Schuergers, Lars Wittig, Gen Enomoto, Julia Herz, Shamphavi Sivabalasarma, Katharina

Ruoff, Friedel Drepper, Sonja-Verena Albers, Pitter F. Huesgen, Annegret Wilde

Brief summary:

The genome of the model cyanobacterium Synechocystis sp. PCC 6803 encodes a diverse repertoire of Type IV pilus (T4P) components, including one major pilin, PilA1, and at least 13 additional putative minor pilins — highlighting the complexity of its pilus machinery. Through systematic genetic analysis, Schuergers et al. reveal that deletion of pilA7-A8 specifically impairs natural competence (DNA uptake) without affecting T4P formation, phototactic motility, or aggregation. In contrast, deletion of pilA4 has no such effect, underscoring the distinct roles of individual minor pilins. Building on this, the authors apply structural phylogenomics across 90 cyanobacterial genomes, uncovering six conserved pilin families: the major pilin PilA, and four minor pilin families — FimT, PilW, PilV, and PilX — plus a novel auxiliary pilin family. Within PilV and PilW, subgroups are identified, reflecting functional diversification. Based on structural and genomic context, two new subfamilies are proposed: PilXEPS (for PilX subunits associated with EPS motility pathways), and PilVEPS (co-occurring with PilXEPS ) and PilVCom/PilXCom (linked to DNA uptake).

Genome-wide analysis reveals that many cyanobacteria possess multiple, co-occurring sets of core minor pilin genes (FimT/U–PilV–PilW–PilX), including both Com-type (competence-associated) and EPS-type (motility-associated) variants. This prediction is experimentally validated in Thermosynechococcus vulcanus, where deletion of specific pilin genes confirms their roles in motility and DNA uptake. A key finding is that individual deletion of either the Com or EPS pilin set in Synechocystis does not disrupt T4P formation, but loss of both sets abolishes pilus assembly entirely — demonstrating functional redundancy and the requirement of at least one pilin set for pilus biogenesis. Using maleimide labeling, the authors visualize morphologically distinct filaments: short, dynamic filaments in strains with only the Com set, and longer, adhesive filaments in those with only the EPS set.

Finally, quantitative proteomics of sheared pilus fractions reveals differential roles among the three PilX3 paralogs, with PilX2 playing a critical role in the accumulation of the PilA9–PilA12 pilin complex — linking specific minor pilins to structural and/or functional specialization.

The key discovery of that paper is that Synechocystis sp. PCC 6803 and probably several other cyanobacteria build two distinct Type IV pilus filaments using a shared core machinery — but with at least two different sets of minor pilins. The paper is filling some important knowledge gaps about the complexity of cyanobacterial T4P systems and is of broad interest for readers interested in the diverse functions of bacterial T4P systems. The paper presents a variety of highly interesting data and is well organized and clearly written. To further strengthen the manuscript, refinements would enhance clarity and consistency: A standardized and consistent naming scheme in text and figures and datasets for major, minor, and auxiliary pilins would improve readability. More detailed descriptions of certain experimental approaches and interpretations would help readers fully appreciate the conclusions.

Major comments:

Line 28: The sentence is misleading. It is the same T4P machine, which is capable to assemble two different type of filaments as written in lines 37 and 38.

Line 40: Tone down the sticky.

Lines 94-95: Please very briefly summarize the findings for PilA1, PilA2, PilA5-6, PilA9-12, PilX1, PilX2, and PilX3 in regard to T4P formation, phototactic motility, aggregation and DNA uptake as a supplementary figure to ease integration of the new presented data. Since the paper is filling some important knowledge gaps, it would also be suitable to provide a table at the end of the manuscript summarizing involvement of all 14 major, minor and auxiliary pilins in T4P formation, phototactic motility, DNA uptake and aggregation.

Lines 147-148: For clarification you must provide for Synechocystis an additional supplementary figure showing the depicted 14 major, minor and auxiliary pilin encoding genes as in figure 2C, but in which you also provide for every protein the locus tags of the genes (slr…), the trivial of the corresponding gene products (pilA5 …..), and the family type (as in dataset S2). Also provide the length of the genes (instead of start and stop) and size of the corresponding gene products in aa. Just an example PilA1 is mentioned all over in the manuscript, neither Figure 2C nor the dataset S2 are indicating which gene is pilA1. I assume it is one of the hofGs, for which no slr numbers are given.

Lines 154-156. Please describe the homology (protein sequence or structure) between this PilX subfamily and HpsA in more detail.

Lines 159-161. Please give references for PilA5 and RntB.

Line 204-211: Why is the analysis of the Thermosynechococcus vulcanus genome not included in the Dataset S2? Which three genes are deleted in the tlr0680-82 mutant and the tll2333-35 mutant? Please clarify.

Lines 222-233: Could the authors try to model the two suggested core minor pilin set using Alphafold? Do the authors assume that the PilV PilW proteins of PilA5-A8 can alternatively associate with PilX1, PilX2 or PilX3? Did the authors tried to delete PilX1 and PilX3 together, or evne all three PilX proteins? What might be role of the two FimT proteins in PilA5-A8?

Lines 236-245: I highly appreciate the search for a functional PilA1 mutation suitable for maleimide labeling. However the chosen PilA1 T77C mutation seems to produce less pili as the WT, that should be stated. Also, based on the hypothesis the WT should produce both forms of T4P, the long and the short ones. Would the pilus dynamics in WT and mutants differ if in that assay DNA would be included? Has that been tested?

Why was it not possible to create motile deletion strains with the PilA1 T77C mutation, as in the WT? Why 2% agar pads were used in these assays, while in all other assays (motility, shear-off) 0.5-0.75% agar was used?

Line 274-275: It would be interesting to see the different sheared pilus fraction by negative-staining TEM. Are the long and short pili differ in width as reported earlier for Synechocystis and Thermus ? The former should also be mentioned in the discussion.

Line 275: Tone down the sentence: Suggested to cap the Com-type tip complex.

Figure 2E: Show the structure for all three PilX proteins. I assume PilX1 is slr0226, PilX2 is slr 0442 and PilX3 is slr1268, that is neither evident from your figure (2C) nor the dataset S2. It would be also helpful to add in the Dataset S2 that PilXEPS /slr2018 is PilA12.

Figure 2F right: Are these representative images of repeated motility and transformation assays. Could the transformation data be quantified as shown in Figure 1F?

Figure 4B: I would be more informative if abundance changes of all 14 pilins would be depicted. Is the abundance of PilA1, PilA2 and PilA4 affected by the different deletions?

Minor comments:

Figure S1: Please explain why the WT or corresponding parent strains of the mutants was not used for TEM.

Figure S3B: Decreased levels of T4P formation are seen for one, two or three clones of T151C, T77C, T80C and T105C.

Figure S3C: Functional motility for T77C and T105C in 2 out of three clones is obvious but not for T80C.

Reviewer #2: Summary:

The major goal of this manuscript was to tackle the challenging question of how functional specialization of T4P is achieved in Cyanobacteria species that contain multiple minor pilin operons. The authors show that deletion of specific minor pilin operons affects distinct T4P-dependent phenotypes (pilA2 and pilA4 are dispensable for motility, transformation, and aggregation, while pilA7-8 are dispensable for aggregation and motility but essential for transformation). The authors use computational analyses to phylogenetically characterize pilins across different species of cyanobacteria, resulting in a clustering of related minor pilins into distinct subclades that may be attributed to their function. The authors then use fluorescence microscopy to visualize T4P dynamics in different minor pilin mutants, followed by mass spectrometry analysis of sheared T4P fractions in different minor pilin mutant backgrounds. While figures 1 and 2 provide interesting and more compelling data, figure 3 lacks control information that is crucial for accurate interpretation of T4P properties. Figure 4 also shows sheared fraction proteomics data that are overinterpreted – without knowing total expression profiles for different minor pilins in each mutant, it is difficult to conclude if changes in certain pilin abundance in sheared fractions is directly due to changes in incorporation or indirectly due to changes in expression. Figure 4 results were also used to draw conclusions about intracellular signaling networks without validating data (such as genetic data or whole cell proteomics data) required for these conclusions.

Major comments:

• The authors’ measured data in 1B and the text indicate the ∆pilA7/8 strain has significant aggregation compared to the WT, but the images don’t really reflect this – can the authors include a supplemental figure with the replicate data used for quantification to display the hyperaggregation phenotype more clearly?

• Are all of these pilins expressed at the same levels? One simple model for how different minor pilins could determine function is differential expression under different conditions

• Where do the different pilins relevant to this manuscript fall in the phylogenetic tree? Arrows indicating their position in Fig 2B would be useful

• The authors should provide quantification for the transformation assays in 2F – the plate images do not clearly depict phenotypes

• For figure S3, are these 3 lanes for each strain different clones? Clearer labeling is needed to interpret these results

• The cysteine knock in strain used by the authors seems to significantly reduce PilA1 surface levels, but the authors do not comment on this or how it might affect their phenotypes. Are reduced surface levels of PilA1 reflective of intracellular PilA1 levels? The answer to this could influence the interpretation of the pilus phenotype results.

• Also, is expression of any of the minor pilins dependent on PilA1 levels or on each other? If so, the phenotypes presented and quantified in Fig 3 may not accurately represent the deletion phenotypes. Given the recent preprint showing that the ratio between minor and major pilin levels can significantly affect dynamics (https://www.biorxiv.org/content/10.64898/2026.01.17.700090v1), I am not confident in the interpretation of these data that incorporation of specific minor pilins per se influences these properties. This draws to question the interpretation that T4P made with different minor pilins are morphologically distinct. Expression data for the minor pilins in the cysteine background strain and in relation to deletions of each other would help strengthen this claim.

• In figure 4, the authors draw conclusions about changes in signaling networks based on changes to the abundance of intracellular proteins found in shear fractions. While this is an interesting observation, it requires significant experimental data to validate. Additionally, was total protein abundance across these strains the same? This can affect mass spectrometry detection limits and quantification accuracy. Also why isn’t pilA7 shown in the heat map?

• What are each of the additional proteins shown in the heat map in fig 4 and why are they relevant?

Minor comments:

• Line 37 “a” should be removed from “a membrane-spanning T4P-machinery”

• The authors should include a WT image of the aggregation phenotype in 1E as they do for the mutants for comparison purposes

• Why is there a 10-fold difference in transformation rates in the WT between 1C and 1F? The error bars look pretty small on the WT in both panels, so it seems strange to have this amount of variability between experiments; also looks like there is significant variability in ∆pilA2 strain in 1F – can the authors comment on this?

Reviewer #3: The manuscript by Schuergers et al. describes their characterization of pilins in cyanobacteria by phylogenic/structural analysis and by assessing phenotypes of putative pilin gene mutants. They find that pilins cluster into distinct families, primarily PilA (major pilin) and minor pilins PilV, PilW, PilX and FimT, which have been characterized in other systems. They show that the minor pilins of Synechocysis and Thermosynechococcus vulcanus are involved in discrete functions of transformation (Com) or motility (EPS) while for the major PilA1 is associated with both functions, at least for Synechocystis. From an analysis of pilus length, piliation levels and pilus dynamics they propose that the different minor pilins form morphologically distinct pili. The results are interesting and nicely described in the Author Summary (“We discovered that cyanobacteria assemble two distinct types of filaments using the same basic machinery.” However the description of these filaments as morphologically distinct in the Abstract and throughout the text is an overinterpretation of the results of the morphological analysis shown in Fig. 3. With no statistical analysis these data do not support the existence in Synechocystis of morphologically distinct filaments. Differences in the priming complexes may alter the efficiency of pilus assembly and retraction (or, more likely of “re-assembly”), as well as adhesion, all of which could impact pilus dynamics, but this is not the same as having morphologically distinct pili. Overall, the manuscript reads to some degree like an internal document that assums deep knowledge of past results and publication and gene targets. A table summarizing the genes, proteins, deletion phenotypes, classifications and organism would have been helpful, as would more such information on each figure. More specific comments are provided below, presented as the issues appear in the manuscript.

Line 26 (Abstract) and elsewhere - What is meant by “tip plug”? Though this term has been used elsewhere it is not clear why the tip must be plugged - suggests that something may otherwise leak out. Rephrase.

Line 86 paragraph (Intro) – At the end of the Introduction the authors pose the question “whether cyanobacteria possess a single T4P machine incorporating specialized accessory pilins or assemble multiple, distinct filaments” but it is not clear what the answer is. They seem to suggest the latter, but it appears to be the former - a single T4P formed from the major pilin PilA1 (at least for Synechocystis), but having one or the other minor pilin priming complex at its tip.

Line 86 paragraph (Intro) – They distinguish between the “machinery” required for motility and DNA uptake, suggesting there are two distinct machines, but in fact there appears to be a single T4P that differs only in its minor pilins at the pilus tip. In the T4P field, the term machinery” is typically used to describe the components that assemble the pilus, i.e. assembly ATPase, platform protein, secretin channel etc., none of which are discussed here. Are there distinct sets of assembly ATPase, platform protein, secretin channel etc. or a single set? What about PilY1?

Line 100 paragraph (Results) – Little is said about PilA1. Remind the reader that PilA1 is a major pilin. Is it the only major pilin in this strain? Two are shown in Fig. 2C and Supp Data Set 2. Which of these corresponds to the PilA1 gene. Was the second major pilin gene deleted and if so, what was the phenotype?

Line 100 paragraph (Results) – The authors state that the aggregation results for the pilA1 deletion do not agree with previous reports but do not elaborate. What were the previous results and did they use the same strain? Could a second major pilin have been present? How is aggregation scored? Emphasize here and elsewhere that PilA1 appears to be required for competence, motility and aggregation, which would suggest that it is pili formed by this major pili that confers all these functions.

Line 135 section - Bioinformatic analysis on 90 cyanobacterial species was performed to identify putative pilin genes, and their structures were predicted and compared, resulting in clustering into 6 different families, but primarily PilA (major pilin) and minor pilins PilV, PilW, PilX and FimT. The structural features of the PilX family were discussed but it wasn’t clear what features resulted in the PilV, PilW and FimT minor pilin classifications. Show characteristic structures for all classifications and compare these to empirically determined structures. What features cause the split in PilX and FimT families? Do all PilX proteins have a hydrophobic residue at position 5 of the mature protein? What about the other pilins?

Line 204 section (Results) - Following the structural classification, deletions were made in putative pilin genes/gene clusters in Thermosynechococcus vulcanus. Why this cyanobacteria? What is its pilin gene organization? It is not shown in Fig. 3C nor in the Supplementary Materials. Do the authors mean T. vestitus?

Line 204 section and Fig. 2F (Results) – The transformation results are not visible and not quantified. Please present as for Fig. 1.

Line 204 section and Fig. 2F (Results) – Deletion of the second major pilin homolog tll1019 resulted in reduction of motility. What about the first major pilin homolog – what is its phenotype? To show that the system shown for Synechocystis, a single pilus with two distinct tips imparting unique functions, is universal for cyanobacteria, the authors should show that T. vulcanus also uses a single major pilin.

Line 234 section and Fig. 3 (Results) – Since the study started with Synechocystis then went to T. vulcanus and then back to Synechocystis, please clearly state which organism is associated with which gene cluster in the text and all figures. Describe the genome organization for the Synechocystis pilins, including the three pilX genes, before describing the experiments. What is the hypothesis re. the pilX genes? They are classified as Com but what are their phenotypes re. motility and competence? Why was pilX_EPS not examined?

Line 234 section and Fig. 4 (Results) – Despite several claims of “statistically significant differences”, stats are not provided for most comparisons. Line 239 reports “striking morphological differences” in pilus dynamics among the mutants, which are not apparent to this reviewer given the large error bars. The same can be said for reported differences in piliation levels and lengths. Yet these data lead the authors to the major conclusion of the manuscript, that there are two morphologically distinct pilus filaments, each with a different function.

283-284 (Results) - What is meant by “enrichment of one coincides with depletion of the other? Enriched how? By knocking out the other genes? More accurately, “depletion of one results in enrichment of others”.

Line 322 paragraph (Discussion): “The Com filament primed by PilA5-8 is short, abundant, and hyper-dynamic. We hypothesize that this morphology is biophysically optimized for DNA scavenging, where rapid extension and retraction cycles increase the sampling frequency required to capture DNA molecules. Conversely, the EPS filament (primed by PilA9-12) is long, sparse, and persistently attached.” These statements, along with claims made in the title, abstract and elsewhere, are not supported by the data. From the data presented, it appears that the two pilus types differ only in their minor pilin priming complexes (which could affect their dynamics).

**********

**Have all data underlying the figures and results presented in the manuscript been provided?**

Large-scale datasets should be made available via a public repository as described in the *PLOS Genetics*  data availability policy, and numerical data that underlies graphs or summary statistics should be provided in spreadsheet form as supporting information.

Reviewer #1: Yes

Reviewer #2: Yes

Reviewer #3: Yes

**********

PLOS authors have the option to publish the peer review history of their article (what does this mean?). If published, this will include your full peer review and any attached files.

If you choose “no”, your identity will remain anonymous but your review may still be made public.

**Do you want your identity to be public for this peer review?**  For information about this choice, including consent withdrawal, please see our Privacy Policy.

Reviewer #1: **Yes:**  Anke Treuner-Lange

Reviewer #2: No

Reviewer #3: No

[NOTE: If reviewer comments were submitted as an attachment file, they will be attached to this email and accessible via the submission site. Please log into your account, locate the manuscript record, and check for the action link "View Attachments". If this link does not appear, there are no attachment files.]

**Figure resubmission:**

While revising your submission, we strongly recommend that you use PLOS’s NAAS tool (https://ngplosjournals.pagemajik.ai/artanalysis) to test your figure files. NAAS can convert your figure files to the TIFF file type and meet basic requirements (such as print size, resolution), or provide you with a report on issues that do not meet our requirements and that NAAS cannot fix.

After uploading your figures to PLOS’s NAAS tool - https://ngplosjournals.pagemajik.ai/artanalysis, NAAS will process the files provided and display the results in the "Uploaded Files" section of the page as the processing is complete. If the uploaded figures meet our requirements (or NAAS is able to fix the files to meet our requirements), the figure will be marked as "fixed" above. If NAAS is unable to fix the files, a red "failed" label will appear above. When NAAS has confirmed that the figure files meet our requirements, please download the file via the download option, and include these NAAS processed figure files when submitting your revised manuscript.

**Reproducibility:**

To enhance the reproducibility of your results, we recommend that authors of applicable studies deposit laboratory protocols in protocols.io, where a protocol can be assigned its own identifier (DOI) such that it can be cited independently in the future. Additionally, PLOS ONE offers an option to publish peer-reviewed clinical study protocols. Read more information on sharing protocols at https://plos.org/protocols?utm_medium=editorial-email&utm_source=authorletters&utm_campaign=protocols

# Author comment: Author response to Decision Letter 1

**DOI:** 10.1371/journal.pgen.1012337.r002

12 Aug 2026

Attachment Submitted filename: Response to Reviewers.pdf

# Peer review: Decision Letter 1

**DOI:** 10.1371/journal.pgen.1012337.r003

**Authors:** Sean Crosson, Ankur Dalia

26 Aug 2026

PGENETICS-D-26-00203R1

Distinct core minor pilin complexes prime specialized type IV filaments in cyanobacteria

PLOS Genetics

Dear Dr. Schuergers,

Thank you for submitting your manuscript to PLOS Genetics. After careful consideration, we feel that it has merit but does not fully meet PLOS Genetics's publication criteria as it currently stands. Therefore, we invite you to submit a revised version of the manuscript that addresses the points raised during the review process.

Please submit your revised manuscript within by Sep 25 2026 11:59PM. If you will need more time than this to complete your revisions, please reply to this message or contact the journal office at plosgenetics@plos.org. When you're ready to submit your revision, log on to https://www.editorialmanager.com/pgenetics/ and select the 'Submissions Needing Revision' folder to locate your manuscript file.

Please include the following items when submitting your revised manuscript:

* A letter that responds to each point raised by the editor and reviewer(s). You should upload this letter as a separate file labeled 'Response to Reviewers'. This file does not need to include responses to formatting updates and technical items listed in the 'Journal Requirements' section below.

* A marked-up copy of your manuscript that highlights changes made to the original version. You should upload this as a separate file labeled 'Revised Manuscript with Track Changes'.

* An unmarked version of your revised paper without tracked changes. You should upload this as a separate file labeled 'Manuscript'.

If you would like to make changes to your financial disclosure, competing interests statement, or data availability statement, please make these updates within the submission form at the time of resubmission. Guidelines for resubmitting your figure files are available below the reviewer comments at the end of this letter.

As the corresponding author, your ORCID iD is verified in the submission system and will appear in the published article. PLOS supports the use of ORCID, and we encourage all coauthors to register for an ORCID iD and use it as well. Please encourage your coauthors to verify their ORCID iD within the submission system before final acceptance, as unverified ORCID iDs will not appear in the published article. Only the individual author can complete the verification step; PLOS staff cannot verify ORCID iDs on behalf of authors.

We look forward to receiving your revised manuscript.

Kind regards,

Ankur B. Dalia

Academic Editor

PLOS Genetics

Sean Crosson

Section Editor

PLOS Genetics

Aimée Dudley

Editor-in-Chief

PLOS Genetics

Anne Goriely

Editor-in-Chief

PLOS Genetics

**Additional Editor Comments (if provided):**

As you will see below, all three authors are enthusiastically supportive of the manuscript. Reviewer 2, however, brings up a few minor points that should be addressed in a revised manuscript.

**Journal Requirements:**

If the reviewer comments include a recommendation to cite specific previously published works, please review and evaluate these publications to determine whether they are relevant and should be cited. There is no requirement to cite these works unless the editor has indicated otherwise.

1) Thank you for stating the Data availability statement. However, we were unable to access the data (The mass spectrometry proteomics data have been deposited to the ProteomeXchange Consortium via the PRIDE [1] partner repository with the dataset identifier PXD074444 and PXD081507) using the information provided. Please review and update the Data Availability Statement to ensure that the underlying data are publicly accessible and that all accession numbers, links, or repository details are correct and functional.

**Reviewers' comments:**

Reviewer's Responses to Questions

**Comments to the Authors:**

**Please note here if the review is uploaded as an attachment.**

Reviewer #1: The authors have done a very good job of addressing all my comments and suggestions as well as those of the other reviewers.

The authors improved the manuscript significantly and the new table and supplementary figures are very helpful.

This paper will be of broad interest for scientist in the field of Type 4 pili biology, biofilm formation and DNA transformation.

Reviewer #2: The new version of the manuscript is significantly improved and is much clearer. The authors have largely addressed my concerns, although there are a few minor points that remain:

Minor comments:

• Please provide PAE plots for AlphaFold-predicted structures. For example, Fig S6 shows two predicted protein structures, but the large disordered loops are likely not accurate. It would be valuable to know which parts of the proteins are confidently predicted. For structures pulled from AlphaFold database, this info is already available and doesn’t need to be provided

• I am not fan of referring to pili made of the same major pilin as different types of filaments since there are species that make different pili with different major pilins. Specifically, lines 90-96, confuses this point. I suggest the authors reword this section to make it clear that the pili are made of the same major pilin. I suggest avoiding using terms like morphologically distinct in relation to the filament, and instead emphasize that the differences are in the tip complex.

• The authors have provided a reasonable explanation to the large discrepancy in transformation rate differences between experiments. For clarity for the reader, can this information be added to the transformation section of the methods?

Reviewer #3: The authors have done an impressive job of addressing reviewer comments. The only point I would add is that although the term "plug" was removed from the text it remains in the Abstract and should similarly be replaced with "cap".

**********

**Have all data underlying the figures and results presented in the manuscript been provided?**

Large-scale datasets should be made available via a public repository as described in the *PLOS Genetics*  data availability policy, and numerical data that underlies graphs or summary statistics should be provided in spreadsheet form as supporting information.

Reviewer #1: Yes

Reviewer #2: Yes

Reviewer #3: Yes

**********

PLOS authors have the option to publish the peer review history of their article (what does this mean?). If published, this will include your full peer review and any attached files.

If you choose “no”, your identity will remain anonymous but your review may still be made public.

**Do you want your identity to be public for this peer review?**  For information about this choice, including consent withdrawal, please see our Privacy Policy.

Reviewer #1: **Yes:**  Anke Treuner-Lange

Reviewer #2: No

Reviewer #3: No

[NOTE: If reviewer comments were submitted as an attachment file, they will be attached to this email and accessible via the submission site. Please log into your account, locate the manuscript record, and check for the action link "View Attachments". If this link does not appear, there are no attachment files.]

**Figure resubmission:**

While revising your submission, we strongly recommend that you use PLOS’s NAAS tool (https://ngplosjournals.pagemajik.ai/artanalysis) to test your figure files. NAAS can convert your figure files to the TIFF file type and meet basic requirements (such as print size, resolution), or provide you with a report on issues that do not meet our requirements and that NAAS cannot fix.

After uploading your figures to PLOS’s NAAS tool - https://ngplosjournals.pagemajik.ai/artanalysis, NAAS will process the files provided and display the results in the "Uploaded Files" section of the page as the processing is complete. If the uploaded figures meet our requirements (or NAAS is able to fix the files to meet our requirements), the figure will be marked as "fixed" above. If NAAS is unable to fix the files, a red "failed" label will appear above. When NAAS has confirmed that the figure files meet our requirements, please download the file via the download option, and include these NAAS processed figure files when submitting your revised manuscript.

**Reproducibility:**

To enhance the reproducibility of your results, we recommend that authors deposit laboratory protocols in protocols.io, where a protocol can be assigned its own identifier (DOI) such that it can be cited independently in the future. Additionally, PLOS ONE offers an option to publish peer-reviewed clinical study protocols. Read more information on sharing protocols at https://plos.org/protocols?utm_medium=editorial-email&utm_source=authorletters&utm_campaign=protocols

# Author comment: Author response to Decision Letter 2

**DOI:** 10.1371/journal.pgen.1012337.r004

25 Sep 2026

Attachment Submitted filename: 2026_09_21_response_to_reviewers.pdf

# Editor report: Decision Letter 2

**DOI:** 10.1371/journal.pgen.1012337.r005

**Authors:** Sean Crosson, Ankur Dalia

29 Sep 2026

Dear Dr Schuergers,

We are pleased to inform you that your manuscript entitled "Distinct core minor pilin complexes prime specialized type IV filaments in cyanobacteria" has been editorially accepted for publication in PLOS Genetics. Congratulations!

Before your submission can be formally accepted and sent to production you will need to complete our formatting changes, which you will receive in a follow up email. Please be aware that it may take several days for you to receive this email; during this time no action is required by you. Please note: the accept date on your published article will reflect the date of this provisional acceptance, but your manuscript will not be scheduled for publication until the required changes have been made.

Once your paper is formally accepted, an uncorrected proof of your manuscript will be published online ahead of the final version, unless you’ve already opted out via the online submission form. If, for any reason, you do not want an earlier version of your manuscript published online or are unsure if you have already indicated as such, please let the journal staff know immediately at plosgenetics@plos.org.

In the meantime, please log into Editorial Manager at https://www.editorialmanager.com/pgenetics/, click the "Update My Information" link at the top of the page, and update your user information to ensure an efficient production and billing process. Note that PLOS requires an ORCID iD for all corresponding authors. Therefore, please ensure that you have an ORCID iD and that it is validated in Editorial Manager. To do this, go to ‘Update my Information’ (in the upper left-hand corner of the main menu), and click on the Fetch/Validate link next to the ORCID field.  This will take you to the ORCID site and allow you to create a new iD or authenticate a pre-existing iD in Editorial Manager.

If you have a press-related query, or would like to know about making your underlying data available (as you will be aware, this is required for publication), please see the end of this email. If your institution or institutions have a press office, please notify them about your upcoming article at this point, to enable them to help maximise its impact. Inform journal staff as soon as possible if you are preparing a press release for your article and need a publication date.

Thank you again for supporting open-access publishing; we are looking forward to publishing your work in PLOS Genetics!

Yours sincerely,

Ankur B. Dalia

Academic Editor

PLOS Genetics

Sean Crosson

Section Editor

PLOS Genetics

Aimée Dudley

Editor-in-Chief

PLOS Genetics

Anne Goriely

Editor-in-Chief

PLOS Genetics

www.plosgenetics.org

BlueSky: @plos.bsky.social

----------------------------------------------------

Comments from the reviewers (if applicable):

----------------------------------------------------

**Data Deposition**

If you have submitted a Research Article or Front Matter that has associated data that are not suitable for deposition in a subject-specific public repository (such as GenBank or ArrayExpress), one way to make that data available is to deposit it in the Dryad Digital Repository. As you may recall, we ask all authors to agree to make data available; this is one way to achieve that. A full list of recommended repositories can be found on our website.

The following link will take you to the Dryad record for your article, so you won't have to re‐enter its bibliographic information, and can upload your files directly:

http://datadryad.org/submit?journalID=pgenetics&manu=PGENETICS-D-26-00203R2

More information about depositing data in Dryad is available at http://www.datadryad.org/depositing. If you experience any difficulties in submitting your data, please contact help@datadryad.org for support.

Additionally, please be aware that our data availability policy requires that all numerical data underlying display items are included with the submission, and you will need to provide this before we can formally accept your manuscript, if not already present.

----------------------------------------------------

**Press Queries**

If you or your institution will be preparing press materials for this manuscript, or if you need to know your paper's publication date for media purposes, please inform the journal staff as soon as possible so that your submission can be scheduled accordingly. Your manuscript will remain under a strict press embargo until the publication date and time. This means an early version of your manuscript will not be published ahead of your final version. PLOS Genetics may also choose to issue a press release for your article. If there's anything the journal should know or you'd like more information, please get in touch via plosgenetics@plos.org.

# Editor report: Acceptance letter

**DOI:** 10.1371/journal.pgen.1012337.r006

**Authors:** Sean Crosson, Ankur Dalia

PGENETICS-D-26-00203R2

Distinct core minor pilin complexes prime specialized type IV filaments in cyanobacteria

Dear Dr Schuergers,

We are pleased to inform you that your manuscript entitled "Distinct core minor pilin complexes prime specialized type IV filaments in cyanobacteria" has been formally accepted for publication in PLOS Genetics! Your manuscript is now with our production department and you will be notified of the publication date in due course.

The corresponding author will soon be receiving a typeset proof for review, to ensure errors have not been introduced during production. Please review the PDF proof of your manuscript carefully, as this is the last chance to correct any errors. Please note that major changes, or those which affect the scientific understanding of the work, will likely cause delays to the publication date of your manuscript.

Soon after your final files are uploaded, unless you have opted out or your manuscript is a front-matter piece, the early version of your manuscript will be published online. The date of the early version will be your article's publication date. The final article will be published to the same URL, and all versions of the paper will be accessible to readers.

For Research Articles, you will receive an invoice from PLOS for your publication fee after your manuscript has reached the completed accept phase. If you receive an email requesting payment before acceptance or for any other service, this may be a phishing scheme. Learn how to identify phishing emails and protect your accounts at https://explore.plos.org/phishing.

Thank you again for supporting PLOS Genetics and open-access publishing. We are looking forward to publishing your work!

With kind regards,

Sharmila Kamatchi

PLOS Genetics

On behalf of:

The PLOS Genetics Team

Carlyle House, Carlyle Road, Cambridge CB4 3DN | United Kingdom

plosgenetics@plos.org | +44 (0) 1223-442823

plosgenetics.org | Twitter: @PLOSGenetics
